# Large-scale genome-wide analyses of stuttering

**DOI:** 10.1038/s41588-025-02267-2

**Published:** 2025-07-28

**Authors:** Hannah G. Polikowsky, Alyssa C. Scartozzi, Douglas M. Shaw, Dillon G. Pruett, Hung-Hsin Chen, Lauren E. Petty, Alexander S. Petty, Emily J. Lowther, Shu-Hsien Cho, Yao Yu, 23andMe Research Team, Stella Aslibekyan, Adam Auton, Elizabeth Babalola, Robert K. Bell, Jessica Bielenberg, Katarzyna Bryc, Emily Bullis, Daniella Coker, Gabriel Cuellar Partida, Devika Dhamija, Sayantan Das, Sarah L. Elson, Teresa Filshtein, Kipper Fletez-Brant, Pierre Fontanillas, Will Freyman, Pooja M. Gandhi, Karl Heilbron, Barry Hicks, David A. Hinds, Ethan M. Jewett, Yunxuan Jiang, Katelyn Kukar, Keng-Han Lin, Maya Lowe, Jey McCreight, Matthew H. McIntyre, Steven J. Micheletti, Meghan E. Moreno, Joanna L. Mountain, Priyanka Nandakumar, Elizabeth S. Noblin, Jared O’Connell, Aaron A. Petrakovitz, G. David Poznik, Morgan Schumacher, Anjali J. Shastri, Janie F. Shelton, Jingchunzi Shi, Suyash Shringarpure, Vinh Tran, Joyce Y. Tung, Xin Wang, Wei Wang, Catherine H. Weldon, Peter Wilton, Alejandro Hernandez, Corinna Wong, Christophe Toukam Tchakouté, Sahar Mozaffari, Christy L. Avery, Kathleen Mullan Harris, Reyna L. Gordon, Janet M. Beilby, Kathryn Z. Viljoen, Robin M. Jones, Chad D. Huff, Heather M. Highland, Shelly Jo Kraft, Jennifer E. Below

**Affiliations:** 1 Vanderbilt Genetics Institute, Vanderbilt University Medical Center, Nashville, TN USA; 2 Hearing and Speech Sciences, Vanderbilt University, Nashville, TN USA; 3 Curtin School of Allied Health, Curtin University, Perth, Australia; 4 Department of Epidemiology, University of Texas MD Anderson Cancer Center, Houston, TX USA; 5 Maze Therapeutics, San Francisco, CA USA; 6 Department of Epidemiology, Gillings School of Global Public Health, University of North Carolina at Chapel Hill, Chapel Hill, NC USA; 7 Carolina Population Center, University of North Carolina at Chapel Hill, Chapel Hill, NC USA; 8 Department of Sociology, University of North Carolina at Chapel Hill, Chapel Hill, NC USA; 9 Department of Otolaryngology, Vanderbilt University Medical Center, Nashville, TN USA; 10 Department of Hearing and Speech, Vanderbilt University Medical Center, Nashville, TN USA; 11 Communication Sciences and Disorders, Wayne State University, Detroit, MI USA; 12 23andMe, Inc, Sunnyvale, CA USA

**Keywords:** Genome-wide association studies, Neurodevelopmental disorders

## Abstract

Developmental stuttering is a highly heritable, common speech condition characterized by prolongations, blocks and repetitions of speech. Although stuttering is highly heritable and enriched within families, the genetic architecture is largely understudied. We reasoned that there are both shared and distinct genetic variants impacting stuttering risk within sex and ancestry groups. To test this idea, we performed eight primary genome-wide association analyses of self-reported stuttering that were stratified by sex and ancestry, as well as secondary meta-analyses of more than one million individuals (99,776 cases and 1,023,243 controls), identifying 57 unique loci. We validated the genetic risk of self-reported stuttering in two independent datasets. We further show genetic similarity of stuttering with autism, depression and impaired musical rhythm across sexes, with follow-up analyses highlighting potentially causal relationships among these traits. Our findings provide well-powered insights into genetic factors underlying stuttering.

## Abstract (web summary)

Genome-wide analyses in over one million self-reported cases and controls identify genetic variants associated with stuttering and find genetic correlations with autism, depression and impaired musical rhythm, supporting a potential neurological basis for stuttering.

## Main

Developmental stuttering is the most common fluency disorder, with more than 400 million people affected worldwide and a lifetime prevalence of 5–8% among global populations^1^. Stuttering is characterized, in part, by syllable and word repetitions, sound prolongations and involuntary breaks between words, called blocks, that disrupt the forward movement of speech^2^. The onset of developmental stuttering typically occurs during childhood between ages 2 and 5, and an estimated 80% of children who stutter will spontaneously recover, with or without the aid of speech therapy^3–5^. Although many individuals who stutter seek therapies, including speech interventions, behavior modification, cognitive interventions and technology-based feedback interventions to manage various aspects of stuttering^6^, outside of spontaneous recovery, stuttering does not have a known cure.

For those who experience persistent stuttering into adolescence and adulthood, the impact can be profound and life-long. People who stutter often describe negative perceptions of identity and self-worth, and reduced overall quality of life^7^. Young people who stutter experience increased bullying and decreased classroom participation, and they report a more negative educational experience; stuttering is associated with depression and suicidal ideation in this population^7–9^. For adults, stuttering can negatively impact employability, perceived job performance, socioeconomic status and mental and social wellbeing^7,8,10,11^.

Developmental stuttering is also sexually dimorphic. At stuttering onset, the male-to-female ratio is approximately even (between 1:1 and 2:1)^4^, but stuttering is substantially more common in males than females in adolescent and adult populations (approximately 4:1)^1,12^ owing to differences in the rate of recovery by sex^13,14^. However, the mechanisms leading to differences in prevalence and the rate of recovery by sex are not known.

Studies of stuttering within families, twins and population isolates provide overwhelming evidence for a strong genetic influence on stuttering risk, with heritability estimates ranging from 0.42 to 0.84 (refs. ^15–26^). To date, family studies have identified six candidate causal stuttering genes: *GNPTAB*, *GNPTG* and *NAGPA*^19,27^; *DRD2* (ref. ^20^); *AP4E1* (ref. ^24^); and *CYP17A1* (ref. ^22^); however, these findings have not replicated in other families and explain little of the genetic heritability of stuttering in populations^28,29^. Two prior studies identified two genome-wide significant loci that confer stuttering risk at the population level^30,31^. These previous investigations, leveraging both family data and global outbred populations, demonstrated that stuttering genetic risk factors are complex and involve both rare, familial and common variation. However, larger sample sizes are needed to elucidate ancestry-specific genetic risk factors for this common complex trait, especially to examine sexual dimorphism. Furthermore, models that leverage genetic risk markers (that is, polygenic risk scores (PRS), genetic correlation analysis and causal inference models) may illuminate the broader clinical impact of the genetic risk of stuttering.

Here, to identify both shared and distinct signals impacting stuttering risk, we report the results of eight primary ancestry-specific and sex-specific genome-wide association studies (GWAS) of stuttering and secondary meta-analyses in samples totaling more than 1.1 million individuals (99,776 cases). These analyses are well-powered to detect stuttering risk alleles with modest effect size and explore shared and distinct genetic effects across genetic ancestry-specific and sex-specific groups through cross-strata look-ups and meta-analyses.

Overall, this study reveals the complex genetic architecture of stuttering, identifying 24 signals in the primary ancestry-specific and sex-specific analyses and 63 signals in secondary meta-analyses for self-reported stuttering, mapping to 57 distinct loci. These loci met a two-tiered multiple test correction threshold. First, we applied a false discovery rate threshold of 5% across all eight primary analyses, and second, a traditional *P* value threshold of 5 × 10^−8^. We validated the observed genetic effects in two independent datasets, including an international clinically ascertained stuttering cohort called the International Stuttering Project (ISP)^30^ and a cohort of self-reported stuttering in the National Longitudinal Study of Adolescent to Adult Health (Add Health)^32^. We then leveraged our results to explore genetic correlations between stuttering and its comorbidities. Together, these advances inform our understanding of the molecular etiology of stuttering and lay groundwork for the future of precision care in developmental speech disorders.

## Results

### Study overview

**Extended Data Fig. 1:**
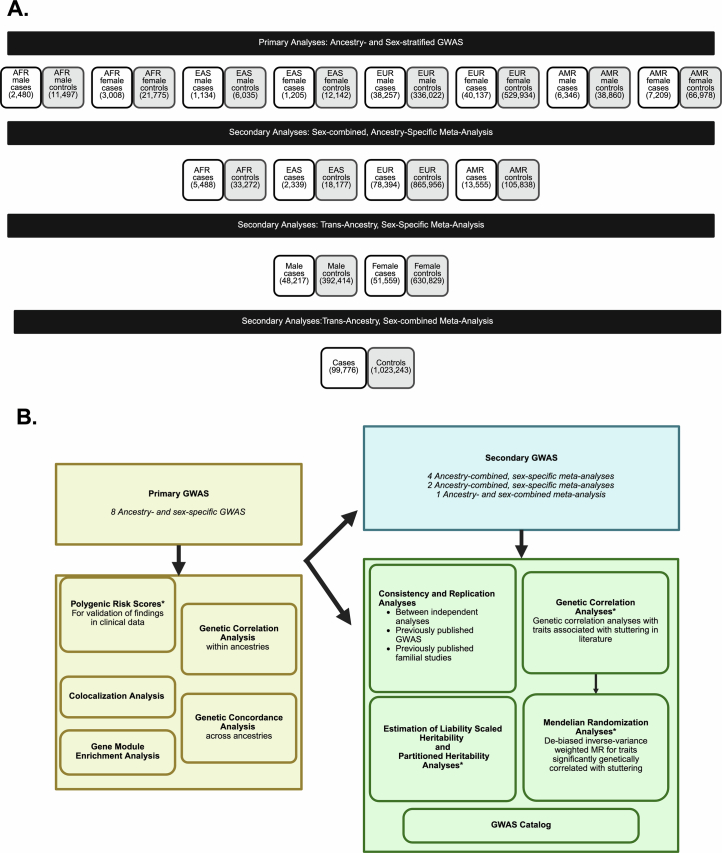
Study design. **a**, Overview of primary and secondary GWAS analyses. **b**, Flow chart of study design and downstream analyses. Primary analyses are indicated in yellow. Secondary analyses are indicated in blue. Green boxes represent analyses that consist of using data from both the primary and secondary analyses. ‘*’ indicate analyses performed in European ancestry only.

We performed eight primary non-overlapping GWAS of self-reported stuttering that were stratified by sex and genetic ancestry in samples from 23andMe, Inc. Stuttering status was based on 23andMe participant responses to the survey question ‘Have you ever had a stammer or stutter?’ The decision to stratify by sex and genetic ancestry group was driven by several factors (see the Supplementary Note for a detailed description of how genetic ancestry and sex groups were defined). In addition to these primary analyses, we report secondary results of meta-analyses across sex by ancestry, meta-analyses across ancestry by sex and a meta-analysis of all eight primary GWAS (see Extended Data Fig. 1 for an overview of analyses).

### GWAS design

In aggregate, the datasets included 99,776 participants responding ‘yes’ to the question ‘Have you ever had a stammer or stutter?’ (cases, 48,217 males; 51,559 females) and 1,023,243 participants responding ‘no’ (controls, 392,414 males; 630,829 females; Table 1). Overall, the study cohort is 60.76% female. Male stuttering prevalence in this dataset is 12.29% and female stuttering prevalence is 8.17%. Distribution by age group is shown in Supplementary Table 1, with age defined as the current age at the time the analysis was conducted. We divided our sample broadly into four continental ancestry groups: African ancestry (AFR), East Asian ancestry (EAS), European ancestry (EUR) and Latino/Admixed American ancestry (AMR), which were defined through genetic analysis^33^ (see Supplementary Table 1 for sample sizes by ancestries and Supplementary Note for description of genetic ancestry determination).

**Table 1:** Demographics of study participants

	Cases (Yes)	Controls (No)
**23andMe**		
Total	99,776	1,023,243
Males	48,217	392,414
Females	51,559	630,829
**Add Health**		
Total	812	8,569
Males	469	3,951
Females	343	4,618
**International Stuttering Project**		
Total	957	6,450
Males	699	4,572
Females	258	1,878

23andMe case status was determined using the survey question, ‘Have you ever had a stammer or stutter?’ Cases represent individuals who answered ‘yes’ to this question; controls represent individuals who answered ‘no’. Add Health case status was determined by the survey question, ‘Do you have a problem with stuttering or stammering?’ Cases represent individuals who at any point answered ‘yes’ to this question; controls always answered ‘no’. Case status in the ISP cohort was determined by speech–language pathologists with expertise in stuttering and fluency disorders.

### Genetic correlations across ancestry-specific and sex-specific GWAS

In the primary analyses of each ancestry-specific and sex-specific GWAS (eight total), we considered autosomal and X chromosome variants that were successfully imputed across all platforms and reached our quality control thresholds (see Methods). We estimated the genetic correlation between the EUR male and EUR female analyses and EAS male and EAS female analyses using linkage disequilibrium score regression (LDSC)^34,35^ (Supplementary Table 2). Statistically significant genetic correlation between sexes was only observed for the EUR population (*r*_g_ = 0.8952, *P* < 10^−50^). Despite this finding, we observed no overlap in genome-wide significant hits between the EUR male and EUR female analyses (Table 2). Comparisons of results across ancestry through concordance analysis can be found in the Supplementary Note.

**Table 2:** Sentinel loci from primary ancestry- and sex-specific GWAS

rsID	Study	Chr	pos_b37	EA	NEA	EAF	OR [95% CI]	*P* value	Functional gene(s)	Location	*q* value
rs35609938	EUR male	2	58,756,729	T	C	0.501	0.95 [0.93–0.96]	5.840 × 10^−12^	*VRK2*	*FANCL*–[]	1.39 × 10^−4^
rs1040225	EUR male	2	58,139,593	G	A	0.598	1.05 [1.04–1.07]	1.820 × 10^−11^	*VRK2*	[*VRK2*]	1.39 × 10^−4^
rs34394051	EUR male	1	6,853,091	G	A	0.157	1.07 [1.04–1.09]	1.510 × 10^−9^	*CAMTA1*	[*CAMTA1*]	0.001
rs545889942*	EUR male	2	104,116,510	I	D	0.445	1.05 [1.03–1.06]	5.070 × 10^−9^	NA	*TMEM182*–[]	0.003
rs72664949	EUR male	13	109,280,508	G	A	0.245	1.06 [1.04–1.07]	7.420 × 10^−9^	*MYO16*	[*MYO16*]	0.003
rs10850379	EUR male	12	110,002,777	T	C	0.445	1.04 [1.03–1.06]	1.770 × 10^−8^	*MMAB*	[*MMAB*]	0.006
rs62337988	EUR male	5	12,031,700	T	A	0.317	1.05 [1.03–1.07]	2.040 × 10^−8^	*CTNND2*	*CTNND2–*[]	0.007
rs11353659	EUR male	15	48,059,138	I	D	0.637	0.96 [0.94–0.97]	2.560 × 10^−8^	*SEMA6D*	[*SEMA6D*]	0.008
rs58120907	EUR male	13	110,413,514	G	A	0.666	0.96 [0.94–0.97]	4.810 × 10^−8^	*IRS2*	[*IRS2*]	0.013
rs558002155	EUR male	8	121,159,409	G	A	0.999	3.51 [2.09–5.89]	4.990 × 10^−8^	*COL14A1*	[*COL14A1*]	0.013
rs13107325	EUR female	4	103,188,709	T	C	0.0813	1.12 [1.09–1.15]	3.810 × 10^−16^	*SLC39A8*	[*SLC39A8*]	7.12 × 10^−8^
rs572319557*	EUR female	18	50,846,441	I	D	0.551	1.05 [1.03–1.06]	2.950 × 10^−10^	*DCC*	[*DCC*]	3.46 × 10^−4^
rs3801279*	EUR female	7	104,904,868	T	C	0.52	0.96 [0.94–0.97]	3.030 × 10^−9^	*SRPK2*	[*SRPK2*]	0.002
15:29934686	EUR female	15	29,934,686	T	C	7.37 × 10^−4^	0.23 [0.13–0.42]	7.530 × 10^−9^	NA	*FAM189A1*–[]–*TJP1*	0.003
rs535503154	EUR female	5	151,965,756	I	D	0.271	0.95 [0.94–0.97]	2.780 × 10^−8^	*NMUR2*	*NMUR2*–[]–*GRIA1*	0.009
rs968163	EUR female	20	51,037,935	G	A	0.258	0.95 [0.94–0.97]	3.810 × 10^−8^	*TSHZ2*	*ZFP64*–[]–*TSHZ2*	0.012
rs529593131	EUR female	17	68,255,397	T	C	2.81 × 10^−4^	0.04 [0.01–0.23]	3.840 × 10^−8^	NA	*KCNJ2*–[]	0.012
rs779897701	EUR female	4	12,449,797	G	C	3.63 × 10^−4^	0.10 [0.03–0.35]	4.050 × 10^−8^	NA	[]–RAB28	0.012
rs62252182	EUR female	3	69,881,433	G	A	0.206	1.05 [1.03–1.07]	4.510 × 10^−8^	*MITF*	[*MITF*]	0.013
rs192857772	AFR male	22	37,824,152	G	A	0.998	0.16 [0.09–0.30]	2.240 × 10^−8^	*CYTH4*	*ELFN2*[]–*MFNG*	0.008
rs7333000	AFR male	13	26,535,079	G	A	0.915	1.40 [1.24–1.59]	4.290 × 10^−8^	*SHISA2*	[*ATP8A2*]	0.012
rs541395135	AFR male	12	80,825,417	I	D	0.945	0.65 [0.56–0.76]	4.360 × 10^−8^	*PTPRQ*	[*PTPRQ*]	0.013
rs35713684	AMR female	10	109,112,494	G	A	0.993	2.23 [1.62–3.06]	4.580 × 10^−8^	*SORCS1*	*SORCS1*–[]	0.013
rs556601931	AMR male	13	41,980,338	T	C	6.76 × 10^−4^	6.84 [3.65–12.84]	2.240 × 10^−8^	*RGCC*	*NAA16*–[]–*RGCC*	0.008

The functional gene(s) represents the variant-to-gene predicted by the Open Targets V2G pipeline (see Methods for details). NA (not available) reported for variants in which Open Targets did not identify a gene. Base pair positions are listed according to human genome reference build 37. Dashes in the location column indicate distance, where [] is contained within the exons of the specified gene; () <1 kb; (-) <10 kb; (–) <100 kb; (—) <1,000 kb either upstream or downstream of the gene. The *q* value represents the false discovery rate-adjusted *P* value when correcting for all autosomal and X chromosome variants in the eight ancestry-specific and sex-specific GWAS. ‘*’ denotes sentinel variants that were within SNP windows for loci previously associated with sex (see ref. ^38^). The ‘Study’ column represents ancestry-specific and sex-specific GWAS; all ancestries represent genetic ancestry. Chr, chromosome; pos_b37, position in build 37; EA, effect allele; NEA, non-effect allele; OR, odds ratio; I, insertion; D, deletion.

### Ancestry- and sex-specific GWAS

**Fig. 1:**
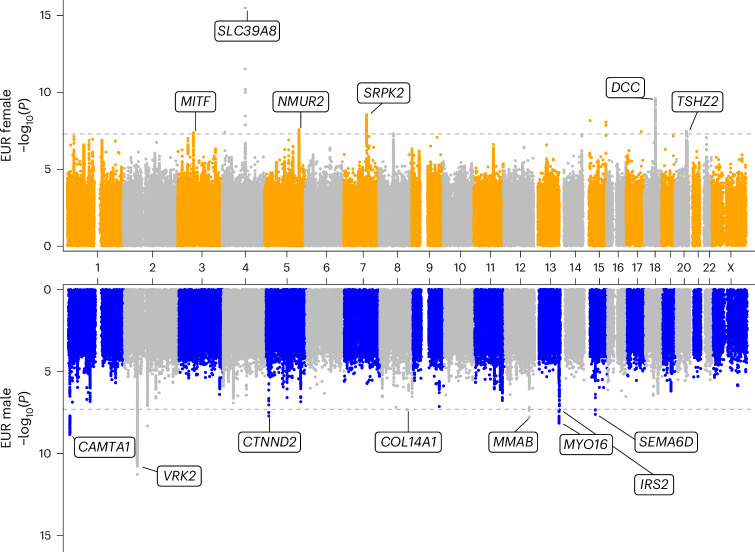
Miami plot of EUR female and EUR male GWAS. The EUR female association study (top panel) included 570,071 total samples (40,137 self-reported stuttering cases) and 29,449,463 autosomal variants. Nine loci reached genome-wide significance (dotted line, *P* < 5.00 × 10^−8^) through logistic regression (see Methods). The EUR male association study (bottom panel) included 374,279 total samples (38,257 self-reported stuttering cases) and 29,409,446 autosomal variants. Ten loci reached genome-wide significance (dotted line, *P* < 5.00 × 10^−8^) through logistic regression (see Methods). The *x* axis represents chromosome base pair coordinates in human genome build 37, and the *y* axis represents observed −log_10_(*P*) for each analysis. Annotated genes for each GWAS are the predicted functional gene for each locus (when available) according to the Open Targets Genetics V2G pipeline (see Methods).

**Extended Data Fig. 2:**
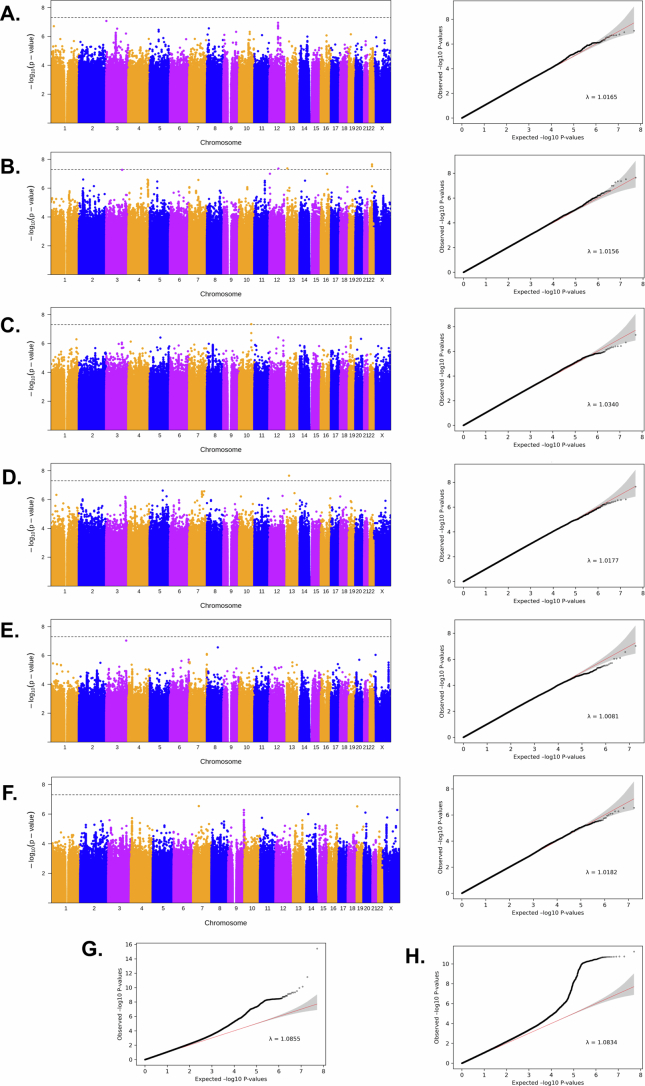
Manhattan and Q-Q plots for primary genome-wide association analyses. Manhattan plots for genome-wide association analyses performed via logistic regression (see Methods). The dotted line represents genome-wide significance (*P* < 5 × 10^−8^). Q-Q plot *x*-axes represent expected −log_10_(*P*-value), and the *y*-axes represent observed −log_10_(*P*-value) with 95% CI indicated by shaded region. **a**, The AFR female association study included 24,783 total samples (3,008 self-reported stuttering cases) and 29,555,214 autosomal variants. No loci reached genome-wide significance (dotted line *P* < 5 × 10^−8^). λ_gc_ = 1.0165. **b**, The AFR male association study included 13,977 total samples (2,480 self-reported stuttering cases) and 29,443,778 autosomal variants. Three loci reached genome-wide significance (dotted line *P* < 5 × 10^−8^). λ_gc_ = 1.0156. **c**, The AMR female association study included 74,187 total samples (7,209 self-reported stuttering cases) and 29,814,878 autosomal variants. One locus reached genome-wide significance (dotted line *P* < 5 × 10^−8^). λ_gc_ = 1.0340. **d**, The AMR male association study included 45,206 total samples (6,346 self-reported stuttering cases) and 29,759,321 autosomal variants. One locus reached genome-wide significance (dotted line *P* < 5 × 10^−8^). λ_gc_ = 1.0177. **e**, The EAS female association study included 13,347 total samples (1,205 self-reported stuttering cases) and 10,595,969 autosomal variants. No loci reached genome-wide significance (dotted line *P* < 5 × 10^−8^). λ_gc_ = 1.0081. **f**, The EAS male association study included 7,169 total samples (1,134 self-reported stuttering cases) and 10,586,108 autosomal variants. No loci reached genome-wide significance (dotted line *P* < 5 × 10^−8^). λ_gc_ = 1.0182. **g**, Q-Q plot for the EUR female GWAS; λ_gc_ = 1.0855. **h**, Q-Q plot for the EUR male GWAS; λ_gc_ = 1.0834.

**Extended Data Fig. 3:**
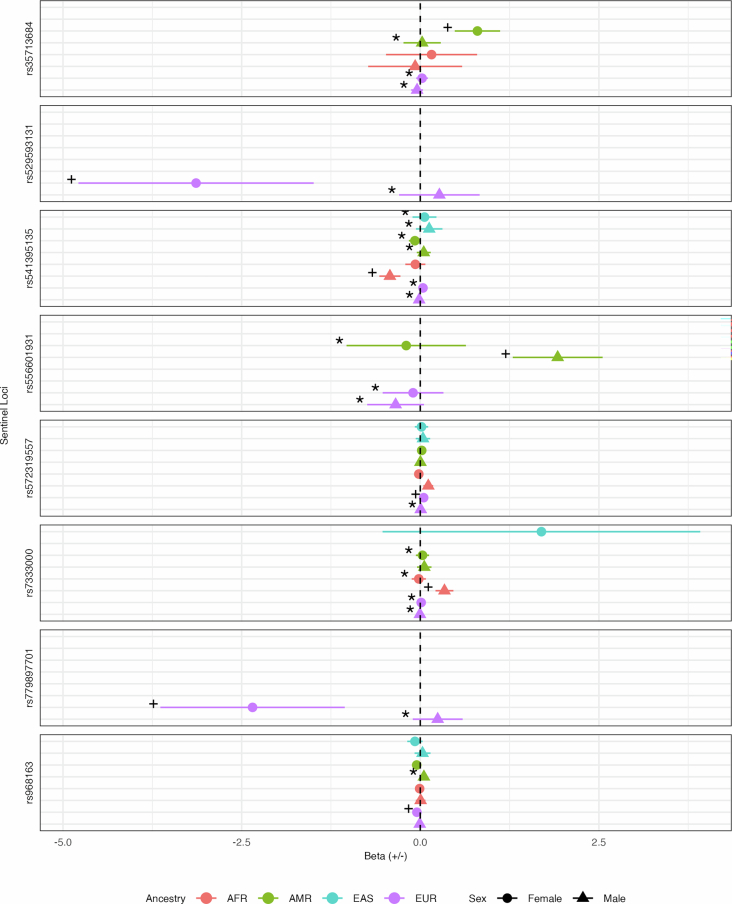
Forest plots showing significant differences in effect size heterogeneity. Wald tests were performed to test for heterogeneity of effect estimates for the 24 sentinel variants that reached genome-wide significance in the eight ancestry- and sex-specific GWAS compared to the other seven primary GWAS. Plot displays the eight sentinel variants that demonstrated significant differences in effect size by ancestry and/or sex. Data points represent effect size +/− standard error. Each genetic ancestry is color coded. Female-specific effect estimates are shown using circles. Male-specific effect estimates are shown using triangles. Effect estimates that are not shown indicate that the variant was not found. The symbol ‘+’ represents the effect of the result in the Discovery GWAS. The symbol ‘*’ denotes significant differences in effect size estimates compared to the Discovery GWAS after Bonferroni correction. Full GWAS samples sizes can be found in Table 1.

From the eight genetic ancestry-specific and sex-specific GWAS, we identified 24 loci (Supplementary Figs. 1–24) associated with stuttering that surpassed a false discovery rate threshold of 5% across all eight primary analyses as well as a conventional genome-wide significance threshold of *P* < 5 × 10^−8^. The identified loci included nine loci from the EUR female study, ten loci from the EUR male study, three loci from the AFR male study and one locus each from the AMR female and AMR male studies (Fig. 1, Table 2 and Extended Data Fig. 2a–h). No loci reached genome-wide significance in the AFR female GWAS, the EAS female GWAS or the EAS male GWAS. Sentinel hits from the EUR male GWAS implicated *VRK2*, *CAMTA1*, *MYO16*, *MMAB*, *CTNND2*, *SEMA6D*, *IRS2* and *COL14A1* as the most likely impacted functional genes (Table 2 and Supplementary Figs. 1–10). Sentinel hits from the EUR female GWAS implicated *SLC39A8*, *DCC*, *SRPK2*, *NMUR2*, *TSHZ2* and *MITF* as the most likely impacted functional genes (Table 2 and Supplementary Figs. 11–19). Sentinel hits from the AFR male GWAS implicated *PTPRQ*, *SHISA2* and *CYTH4* as likely functional genes; AMR female GWAS implicated *SORCS1*; and AMR male GWAS implicated *RGCC* (Table 2 and Supplementary Figs. 20–24). Across all eight ancestry-specific and sex-specific GWAS, no loci on the X chromosome surpassed genome-wide significance. We also present findings from secondary sex-combined meta-analyses, ancestry-combined meta-analyses and a meta-analysis of all sex and ancestry groups (see Extended Data Fig. 1 for study overview and Extended Data Fig. 3, Methods and Supplementary Note for heterogeneity of effect size estimates in the eight primary GWAS).

### Sex-combined, ancestry-specific meta-analyses

To better understand ancestry-specific effects across sexes, we performed sex-combined, ancestry-specific meta-analyses in METAL^36^. We identified 36 loci associated with stuttering at the conventional significance threshold of *P* < 5 × 10^−8^ (Supplementary Figs. 25–60). Specifically, 28 loci were associated with stuttering in the sex-combined EUR analysis (Table 3, Extended Data Fig. 4a and Supplementary Figs. 25–52), seven loci were associated with stuttering in the sex-combined AMR analysis (Table 3, Extended Data Fig. 4b and Supplementary Figs. 53–59) and one locus was associated with stuttering in the sex-combined EAS analysis (Table 3, Extended Data Fig. 4c and Supplementary Fig. 60). No loci reached the conventional significance threshold in the sex-combined AFR analysis (Extended Data Fig. 4d).

**Table 3:** Sentinel loci from secondary sex-combined, ancestry-specific meta-analysis

rsID	Study	Chr	pos_b37	EA	NEA	EAF	OR [95% CI]	*P* value	Functional gene(s)	Location	*P* value heterogeneity
rs13107325	Sex-combined EUR	4	103,188,709	T	C	0.0826	1.09 [1.07–1.12]	1.150 × 10^−19^	*SLC39A8*	[*SLC39A8*]	0.036
rs72808425	Sex-combined EUR	2	58,124,453	T	C	0.7027	0.96 [0.95–0.97]	1.250 × 10^−12^	*VRK2*	[]-*VRK2*	0.273
rs12035477	Sex-combined EUR	1	6,906,171	G	C	0.1567	0.95 [0.94–0.96]	2.160 × 10^−12^	*CAMTA1*	[*CAMTA1*]	0.176
rs11895946	Sex-combined EUR	2	141,004,513	T	C	0.6667	0.96 [0.95–0.97]	2.930 × 10^−11^	*LRP1B*	[*LRP1B*]	0.844
rs12040559	Sex-combined EUR	1	96,337,925	T	G	0.9010	1.06 [1.04–1.08]	5.650 × 10^−11^	*PTBP2*	*RWDD3*–[]–*PTBP2*	0.870
rs34749698	Sex-combined EUR	11	88,799,731	T	C	0.7464	0.96 [0.95–0.97]	1.940 × 10^−10^	*GRM5*	*GRM5* []–*TYR*	0.754
rs4331897	Sex-combined EUR	5	12,019,375	G	A	0.3161	0.96 [0.95–0.97]	3.940 × 10^−10^	*CTNND2*	*CTNND2*–[]	0.159
rs11264408	Sex-combined EUR	1	153,765,007	G	A	0.3712	1.04 [1.03–1.05]	4.420 × 10^−10^	*CREB3L4*	[*GATAB2D*]	0.601
rs11353659*	Sex-combined EUR	15	48,059,138	I	D	0.6374	1.04 [1.02–1.05]	6.770 × 10^−10^	*SEMA6D*	[*SEMA6D*]	0.119
rs6606725	Sex-combined EUR	12	109,905,368	C	A	0.5432	1.03 [1.02–1.04]	2.080 × 10^−9^	*KCTD10*	[*KCTD10*]	0.114
rs10109510	Sex-combined EUR	8	64,882,069	T	C	0.6330	0.97 [0.96–0.98]	2.110 × 10^−9^	NA	*YTHDF3*–[]–*BHLHE22*	0.195
rs559830614	Sex-combined EUR	6	139,031,221	T	C	0.9999	0.25 [0.16–0.39]	2.330 × 10^−9^	NA	*NHSL1*-[]–*CCDC28A*	0.518
rs745532	Sex-combined EUR	9	33,927,325	G	A	0.6261	0.97 [0.96–0.98]	4.820 × 10^−9^	*UBAP2*	[*UBAP2*]	0.577
rs1836999	Sex-combined EUR	5	104,117,403	T	A	0.4733	1.03 [1.02–1.04]	6.060 × 10^−9^	NA	*NUDT12*–[]	0.705
rs147393141	Sex-combined EUR	15	100,368,551	T	C	0.0001	3.76 [2.41–5.88]	6.090 × 10^−9^	*LYSMD4*	*LYSMD4*–[]–*ADAMTS17*	0.009
rs59183769	Sex-combined EUR	17	67,505,113	G	A	0.9999	3.56 [2.31–5.47]	7.530 × 10^−9^	*MAP2K6*	[*MAP2K6*]	0.921
rs9384679	Sex-combined EUR	6	108,864,419	T	C	0.3715	0.97 [0.96–0.98]	9.430 × 10^−9^	*ARMC2*	*AGFL1*–[]-*FOXO3*	0.279
rs7139049	Sex-combined EUR	12	24,273,754	T	G	0.6771	0.97 [0.96–0.98]	9.460 × 10^−9^	*SOX5*	[*SOX5*]	0.196
rs8100340	Sex-combined EUR	19	36,999,682	T	C	0.2328	1.04 [1.02–1.05]	1.460 × 10^−8^	*ZNF567*	*ZNF566*-[] *ZNF260*	0.243
rs182518401	Sex-combined EUR	3	19,429,428	T	G	0.0001	3.93 [2.45–6.32]	1.470 × 10^−8^	*KCNH8*	[*KCNH8*]	0.105
rs537077603	Sex-combined EUR	9	84,321,488	I	D	0.4739	1.03 [1.02–1.04]	1.970 × 10^−8^	NA	*TLE1*-[]–*SPATA31D4*	0.195
rs11931307	Sex-combined EUR	4	62,241,650	T	C	0.2584	0.96 [0.95–0.98]	2.750 × 10^−8^	*ADGRL3*	[*ADGRL3*]	0.494
rs235426	Sex-combined EUR	8	133,813,884	G	A	0.3246	1.03 [1.02–1.05]	3.230 × 10^−8^	*TMEM71*	[*PHF20L1*]	0.293
rs11167732	Sex-combined EUR	5	154,970,396	G	A	0.6195	1.03 [1.02–1.04]	3.370 × 10^−8^	*SGCD*	*KIF4AB*–[]–*SGCD*	0.424
rs758717390	Sex-combined EUR	7	116,332,923	T	G	0.9997	0.45 [0.34–0.60]	3.760 × 10^−8^	*MET*	[*MET*]	0.023
5:115941955	Sex-combined EUR	5	115,941,955	T	C	0.0002	2.69 [1.89–3.84]	4.080 × 10^−8^	NA	*SEMA6A*–[]	0.512
rs35026344	Sex-combined EUR	10	95,466,614	I	D	0.4772	1.03 [1.02–1.04]	4.180 × 10^−8^	NA	*FRA10AC1*-[]–*LGI1*	0.623
rs112352911	Sex-combined EUR	7	104,985,287	I	D	0.4793	1.03 [1.02–1.05]	4.450 × 10^−8^	*SRPK2*	[*SRPK2*]	0.085
rs141986240	Sex-combined AMR	8	92,110,308	G	A	0.0016	0.38 [0.28–0.52]	1.360 × 10^−9^	*LRRC69*	*OTUD6N*-[]-*LRRC69*	0.912
rs146784668	Sex-combined AMR	4	20,903,635	G	A	0.9954	1.64 [1.38–1.95]	1.560 × 10^−8^	*PACRGL*	[*KCNIP4*]	0.944
rs559419877:GA	Sex-combined AMR	4	71,289,806	I	D	0.0035	0.51 [0.40–0.64]	1.590 × 10^−8^	NA	*OPRPN*-[]-*MUC7*	0.849
rs188750450	Sex-combined AMR	15	41,340,355	G	C	0.9992	2.71 [1.91–3.84]	2.010 × 10^−8^	NA	[*INO80*]	0.689
rs537943976	Sex-combined AMR	5	165,853,802	T	C	0.0010	2.51 [1.81–3.48]	2.890 × 10^−8^	NA	[]–*TENM2*	0.776
rs578220221	Sex-combined AMR	4	17,383,583	G	C	0.9994	4.53 [2.65–7.75]	3.590 × 10^−8^	*CLRN2*	[]–[*QDPR*]	0.741
rs556819764	Sex-combined AMR	16	87,027,400	T	C	0.0003	5.49 [2.99–10.07]	4.050 × 10^−8^	*C16orf95*	[]–*C16orf95*	0.833
rs140279480	Sex-combined EAS	4	123,158,809	T	C	0.0025	5.05 [2.83–9.00]	4.110 × 10^−8^	*ADCY5*	[*ADCY5*]	0.433

The functional gene(s) represents the variant-to-gene predicted by the Open Targets V2G pipeline (see Methods for details). NA reported for variants in which Open Targets did not identify a gene. Base pair positions are listed according to human genome reference build 37. Dashes in the location column indicate distance, where [] is contained within the exons of the specified gene: () <1 kb; (-) <10 kb; (–) <100 kb; (—) <1,000 kb either upstream or downstream of the gene. ‘*’ denotes sentinel variants that were within SNP windows for loci previously associated with sex (see ref. ^38^). The ‘Study’ column represents sex-combined, ancestry-specific meta-analyses; all ancestries represent genetic ancestry. Chr, chromosome; pos_b37, position in build 37; EA, effect allele; NEA, non-effect allele; OR, odds ratio; I, insertion; D, deletion.

Sentinel hits from the sex-combined EUR meta-analysis implicated *SLC39A8*, *VRK2*, *CAMTA1*, *LRP1B*, *PTBP2*, *GRM5*, *CTNND2*, *CREB3L4*, *SEMA6D*, *KCTD10*, *UBAP2*, *LYSMD4*, *MAP2K6*, *ARMC2*, *SOX5*, *ZNF567*, *KCNH8*, *ADGRL3*, *TMEM71*, *SGCD*, *MET* and *SRPK2* as the most likely impacted functional genes (Table 3). Sentinel hits from the sex-combined AMR meta-analysis implicated *LRRC69*, *PACRGL*, *CLRN2* and *C16orf95* as the most likely impacted functional genes (Table 3). Sentinel hits from the sex-combined EAS meta-analysis implicated *ADCY5* as a likely functional gene (Table 3).

### Ancestry-combined, sex-specific meta-analyses

**Extended Data Fig. 5:**
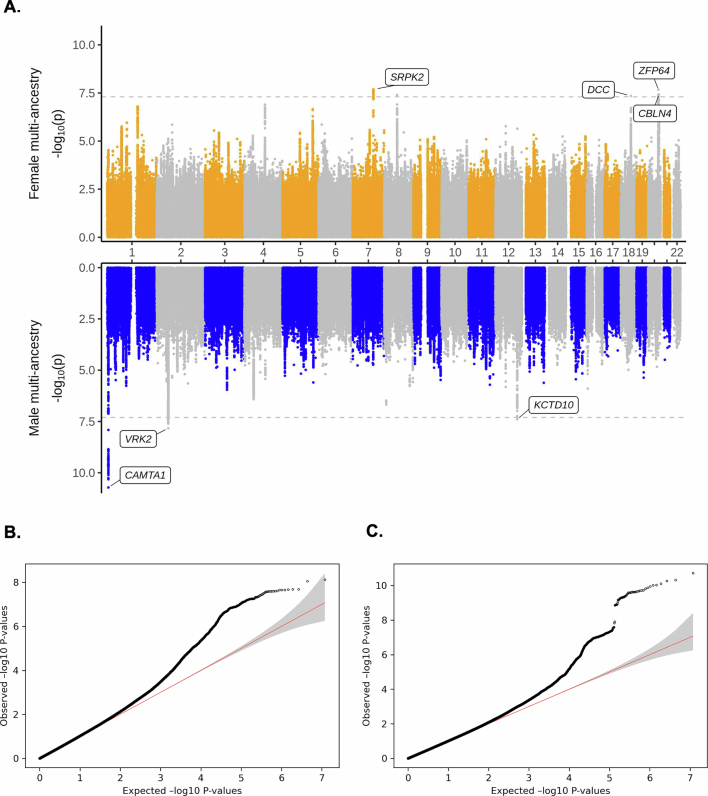
Miami plot for female-specific meta-analysis and male-specific meta-analysis. Ancestry-combined, sex-specific meta-analyses were performed using multi-ancestry meta-regression via MR-MEGA (see Methods). **a**, The multi-ancestry female meta-analysis (top panel in orange) included 682,388 total samples (51,559 self-reported stuttering cases) and 41,960,804 autosomal variants (see Methods). Five loci reached genome-wide significance (dotted line *P* < 5 × 10^−8^). The multi-ancestry male meta-analysis (bottom panel in blue) included 440,631 total samples (48,217 self-reported stuttering cases) and 41,937,718 autosomal variants (see Methods). Three loci reached genome-wide significance (dotted line *P* < 5 × 10^−8^). The x-axis represents chromosome base pair coordinates in human genome build 37, and the y-axis represents observed −log_10_(*P*-value) for each GWAS. Annotated genes for each GWAS includes the predicted functional gene for each loci (when available) according to Open Targets V2G pipeline. **b**, Q-Q plot for the female-specific meta-analysis. **c**, Q-Q plot for the male-specific meta-analysis. Q-Q plot *x*-axes represent expected −log_10_(*P*-value), and *y*-axes represent observed −log_10_(*P*-value), with 95% CI indicated by shaded region. Note: *P*-values were adjusted for genomic control.

We also aggregated association summary statistics by sex across ancestry groups by multi-ancestry meta-regression, using MR-MEGA^37^, and identified five loci in our female-specific meta-analysis (Table 4, Extended Data Fig. 5a,b and Supplementary Figs. 61–65) and three loci in our male-specific meta-analysis (Table 4, Extended Data Fig. 5a,c and Supplementary Figs. 66–68).

**Table 4:** Sentinel loci from secondary ancestry-combined, sex-specific meta-analyses and ancestry-combined and sex-combined meta-analysis

rsID	Study	Chr	pos_b37	EA	NEA	EAF	Beta	SE	*P* value	Functional gene(s)	Location	*P* value heterogeneity
rs10109510	Female-specific meta-analysis	8	64,882,069	T	C	0.6430	−0.043	0.010	7.460 × 10^−9^	NA	*YTHDF3*–[]–*BHLHE22*	0.422
rs3801279	Female-specific meta-analysis	7	104,904,868	T	C	0.5000	−0.051	0.007	2.050 × 10^−8^	*SRPK2*	[*SRPK2*]	0.744
rs13043844	Female-specific meta-analysis	20	50,959,453	G	C	0.1610	−0.070	0.011	2.080 × 10^−8^	*ZFP64*	*ZFP64*–[]–*TSHZ2*	0.605
rs36075020	Female-specific meta-analysis	20	54,698,252	I	D	0.7500	0.019	0.003	3.750 × 10^−8^	*CBLN4*	*CBLN4*–[]–*MC3R*	0.943
rs6508220	Female-specific meta-analysis	18	50,831,176	G	A	0.4940	−0.037	0.011	4.490 × 10^−8^	*DCC*	[*DCC*]	0.421
rs950493	Male-specific meta-analysis	1	6,944,804	T	C	0.8400	−0.077	0.016	1.920 × 10^−11^	*CAMTA1*	[*CAMTA1*]	0.212
rs966721	Male-specific meta-analysis	2	58,197,305	T	A	0.3790	−0.056	0.022	1.480 × 10^−8^	*VRK2*	[*VRK2*]	0.036
rs6606725	Male-specific meta-analysis	12	109,905,368	C	A	0.5380	−0.038	0.005	4.050 × 10^−8^	*KCTD10*	[*KCTD10*]	0.836
rs13107325	Ancestry and sex-combined meta-analysis	4	103,188,709	T	C	7.611	4.324	4.324	1.910 × 10^−17^	*SLC39A8*	[*SLC39A8*]	0.162
rs950493	Ancestry and sex-combined meta-analysis	1	6,944,804	T	C	−0.061	0.019	0.019	4.650 × 10^−14^	*CAMTA1*	[*CAMTA1*]	0.038
rs13392894	Ancestry and sex-combined meta-analysis	2	58,124,471	T	C	0.025	0.011	0.011	6.320 × 10^−11^	*VRK2*	[]–*VRK2*	0.412
rs2841108	Ancestry and sex-combined meta-analysis	1	154,013,220	T	G	0.135	1.401	1.401	1.430 × 10^−10^	*CREB3L4*	[*NUP210L*]	0.433
rs3217162	Ancestry and sex-combined meta-analysis	12	109,888,159	I	D	−0.032	0.012	0.012	8.260 × 10^−10^	*KCTD10*	[*KCTD10*]	0.258
rs669480	Ancestry and sex-combined meta-analysis	11	88,677,665	T	C	−0.022	0.009	0.009	1.290 × 10^−9^	*CTSC*	[*GRM5*]	0.593
rs4624326	Ancestry and sex-combined meta-analysis	2	140,976,973	T	G	0.016	0.011	0.011	1.680 × 10^−9^	NA	[]–*LRP1B*	0.592
rs10842270*	Ancestry and sex-combined meta-analysis	12	24,232,279	C	A	−0.007	0.013	0.013	1.700 × 10^−9^	*SOX5*	*SOX5*–[]–*BCAT1*	0.308
rs11495450	Ancestry and sex-combined meta-analysis	12	52,135,563	T	C	−0.524	0.879	0.879	1.820 × 10^−9^	*SCN8A*	[*SCN8A*]	0.751
rs7806413*	Ancestry and sex-combined meta-analysis	7	69,501,025	C	A	−0.124	0.010	0.010	2.900 × 10^−9^	*AUTS2*	[*AUTS2*]	0.917
rs650891	Ancestry and sex-combined meta-analysis	9	103,842,199	T	A	−0.054	0.010	0.010	3.420 × 10^−9^	*PLPPR1*	[*PLPPR1*]	0.729
rs10109510	Ancestry and sex-combined meta-analysis	8	64,882,069	T	C	−0.034	0.015	0.015	3.910 × 10^−9^	NA	*YTHDF3*–[]–*BHLHE22*	0.128
rs4331897	Ancestry and sex-combined meta-analysis	5	12,019,375	G	A	−1.915	2.486	2.486	6.570 × 10^−9^	*CTNND2*	*CTNND2*–[]	0.125
rs12040559	Ancestry and sex-combined meta-analysis	1	96,337,925	T	G	0.024	0.006	0.006	9.090 × 10^−9^	*PTBP2*	*RWDD3*–[]–*PTBP2*	0.976
rs56090583	Ancestry and sex-combined meta-analysis	13	62,745,302	I	D	−0.038	0.010	0.010	1.530 × 10^−8^	NA	AL592490.1–[]	0.452
rs7228924	Ancestry and sex-combined meta-analysis	18	51,302,255	G	C	3.261	0.538	0.538	1.720 × 10^−8^	*POLI*	*DCC*–[]	0.969
rs143169712	Ancestry and sex-combined meta-analysis	4	95,765,215	C	A	12.829	19.047	19.047	3.850 × 10^−8^	*BMPR1B*	[*BMPR1B*]	0.065
rs73032465	Ancestry and sex-combined meta-analysis	19	37,000,172	T	C	3.815	1.487	1.487	4.130 × 10^−8^	*ZNF567*	*ZNF566-*[]-*ZNF567*	0.738
rs141986240	Ancestry and sex-combined meta-analysis	8	92,110,308	G	A	−2.128	6.623	6.623	4.800 × 10^−8^	*LRRC69*	*OTUD68*-[]-*LRRC69*	0.882

The functional gene(s) represents the variant-to-gene predicted by the Open Targets V2G pipeline (see Methods for details). NA is reported for variants in which Open Targets did not identify a gene. Base pair positions are listed according to human genome reference build 37. Dashes in the location column indicate distance, where [] is contained within the exons of the specified gene: () <1 kb; (-) <10 kb; (–) <100 kb; (—) <1,000 kb either upstream or downstream of the gene. ‘*’ denotes sentinel variants that were within SNP windows for loci previously associated with sex (see ref. ^38^). Chr, chromosome; pos_b37, position in build 37; EA, effect allele; NEA, non-effect allele; Beta (beta_0), the effect of intercept meta-regression; SE (se_0), standard error of intercept meta-regression; I, insertion; D, deletion.

Sentinel hits from the female-specific meta-analysis implicated *SRPK2*, *ZFP64*, *CBLN4* and *DCC* as the most likely impacted functional genes (Table 4). Sentinel hits from the male-specific meta-analysis implicated *CAMTA1*, *VRK2* and *KCTD10* as the most likely impacted functional genes (Table 4).

### Ancestry-combined and sex-combined meta-analysis

Last, we performed an ancestry-combined and sex-combined meta-analysis, using all ancestry-specific and sex-specific preliminary GWAS analyses, through multi-ancestry meta-regression, again using MR-MEGA^37^. This analysis identified 19 loci reaching the conventional significance threshold (Table 4, Extended Data Fig. 6 and Supplementary Figs. 69–87). Sentinel hits from the ancestry-combined and sex-combined meta-analysis were mapped to *SLC39A8*, *CAMTA1*, *CBLN4*, *VRK2*, *CREB3L4*, *KCTD10*, *CTSC*, *SOX5* and *SCN8A* as the most likely impacted functional genes (Table 4).

From these sentinel hits, we report 57 distinct loci (defined as non-overlapping within ±1 Mb of the sentinel single-nucleotide polymorphism (SNP)) associated with self-reported stuttering across the primary ancestry-specific and sex-specific GWAS and the secondary sex-combined, ancestry-specific meta-analyses, ancestry-combined, sex-specific meta-analyses, and ancestry-combined, sex-combined meta-analysis. However, sex-specific participation bias can result in spurious autosomal associations^38^. Hence, we compared the 57 stuttering-associated loci to 158 autosomal loci spuriously associated with sex in prior work^38^. We found six of the 57 stuttering-associated loci were located within the SNP windows for loci previously associated with sex-specific participation bias (Table 4). For consistency of association between non-overlapping samples within this study as well as with previously identified stuttering loci in the literature, please see the Supplementary Note.

### Genetic heritability

We calculated SNP-based liability-scale heritability for our male and female EUR studies using LDSC^34,35^. As with the genetic correlation analyses, for AFR and AMR, LDSC and other common approaches are biased in the presence of admixture. For EAS, the sample size prevented reliable heritability estimates^39^. Hence, heritability analyses were not performed for the AFR, AMR and EAS ancestries. Liability-scaled heritability was estimated to be 9.11% (SE = 0.0054) for EUR females and 9.62% (SE = 0.0052) for EUR males, assuming a stuttering population prevalence of 10%. LDSC intercept and observed scale heritability estimates are reported in Supplementary Table 7.

**Extended Data Fig. 7:**
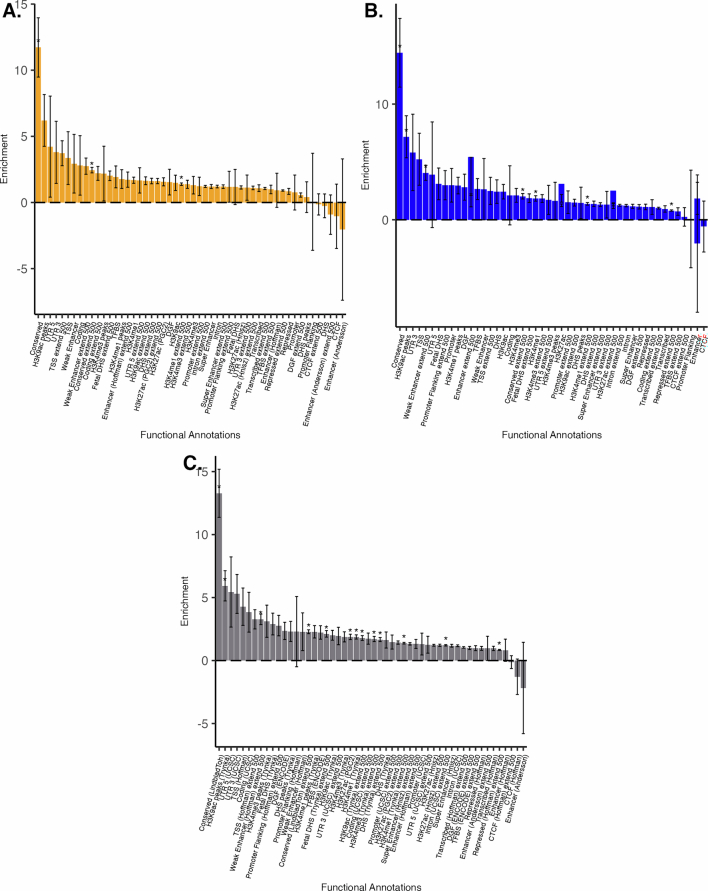
Partitioned heritability estimates for broad functional categories. Partitioned heritability was performed using the 52 broad functional annotations defined by Finucane et al. Enrichment is the proportion of *h*^2^/proportion of SNPs. The horizontal, dotted line indicates no enrichment. Error bars indicate enrichment estimates +/− standard error. Asterisks indicate enrichment estimates are significant based on a Bonferroni *P*-value of 9.6 × 10^−4^ (52 gene-sets). **a**, Broad functional categories partitioned heritability estimates in EUR female self-reported stuttering. **b**, Broad functional categories partitioned heritability estimates in EUR male self-reported stuttering. **c**, Broad functional categories partitioned heritability estimates in sex-combined EUR self-reported stuttering. Full results are presented in Supplementary Tables 8–10.

Partitioned SNP-based heritability of stuttering by broad functional annotation^35^ showed significant enrichments of conserved regions, as well as a chromatin mark for enhancers, H3K4me1, in EUR male, EUR female and sex-combined EUR stuttering results (Extended Data Fig. 7 and Supplementary Tables 8–10). EUR male and sex-combined EUR stuttering results were enriched for weak enhancers, repressed marks and a marker for active chromatin, H3K9ac (Extended Data Fig. 7 and Supplementary Tables 8–10; *P* < 9.6 × 10^−4^). Sex-combined EUR stuttering results were enriched for fetal and adult DNase hypersensitive sites, introns and marks of active chromatin sites H3K27ac and H3K4me3 (Extended Data Fig. 7 and Supplementary Table 8; *P* < 9.60 × 10^−4^).

**Extended Data Fig. 8:**
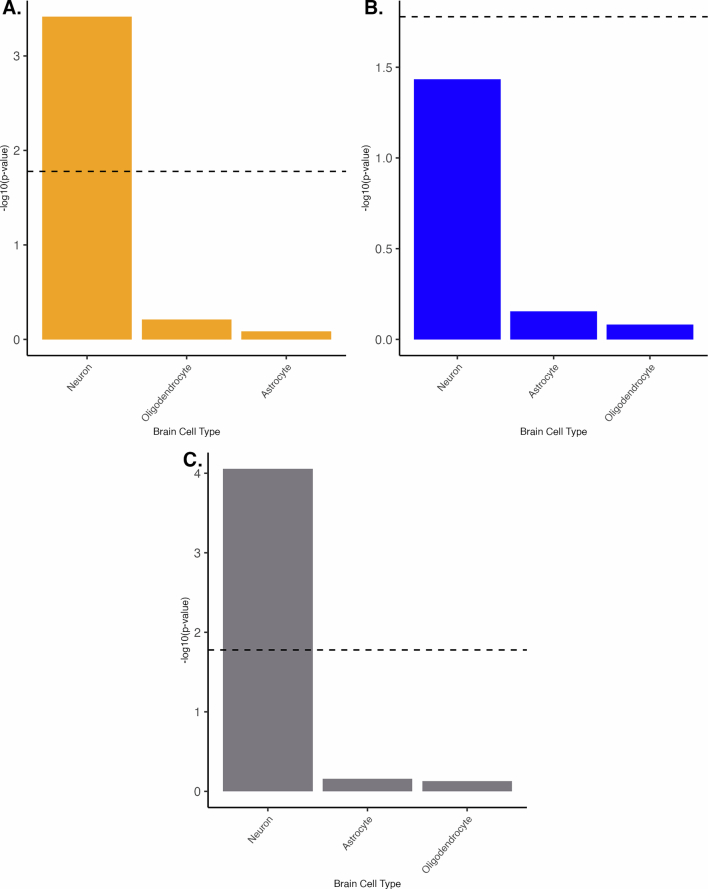
Partitioned heritability estimates for brain cell types. Partitioned heritability was performed using the three cell-type annotations defined by Finucane et al. The −log_10_(*P*-value) for enrichment estimates is indicated on the *y*-axis. The horizontal dotted line indicates significant after Bonferroni correction (*P* < 1.7 × 10^−2^, three tests). **a**, Brain cell type partitioned heritability estimates in EUR female self-reported stuttering. **b**, Brain cell type partitioned heritability estimates in EUR male self-reported stuttering. **c**, Brain cell type partitioned heritability estimates in sex-combined EUR self-reported stuttering. Full results are presented in Supplementary Tables 11–13.

**Extended Data Fig. 9:**
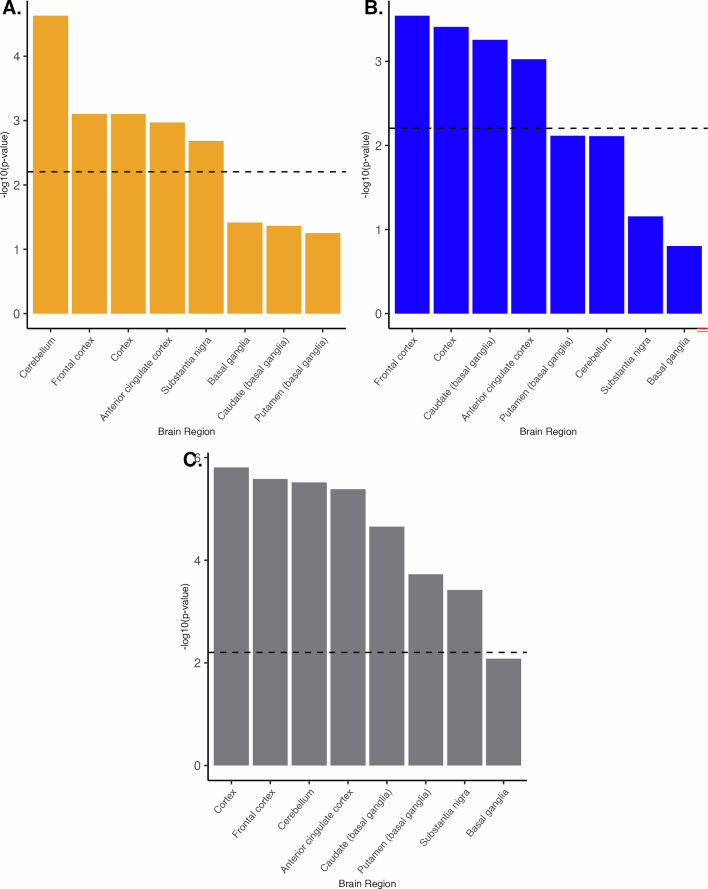
Partitioned heritability estimates for tissue-specific gene expression. Partitioned heritability was performed using the tissue-specific expression annotations defined by Finucane et al. The −log_10_(*P*-value) for enrichment estimates is indicated on the *y*-axis. The horizontal dotted line indicates significant after Bonferroni correction (*P* < 6.25 × 10^−3^, eight tests). **a**, Tissue-specific gene expression partitioned heritability estimates in EUR female self-reported stuttering. **b**, Tissue-specific gene expression partitioned heritability estimates in EUR male self-reported stuttering. **c**, Tissue-specific gene expression partitioned heritability estimates in sex-combined EUR self-reported stuttering. Full results are presented in Supplementary Tables 11–13.

**Extended Data Fig. 10:**
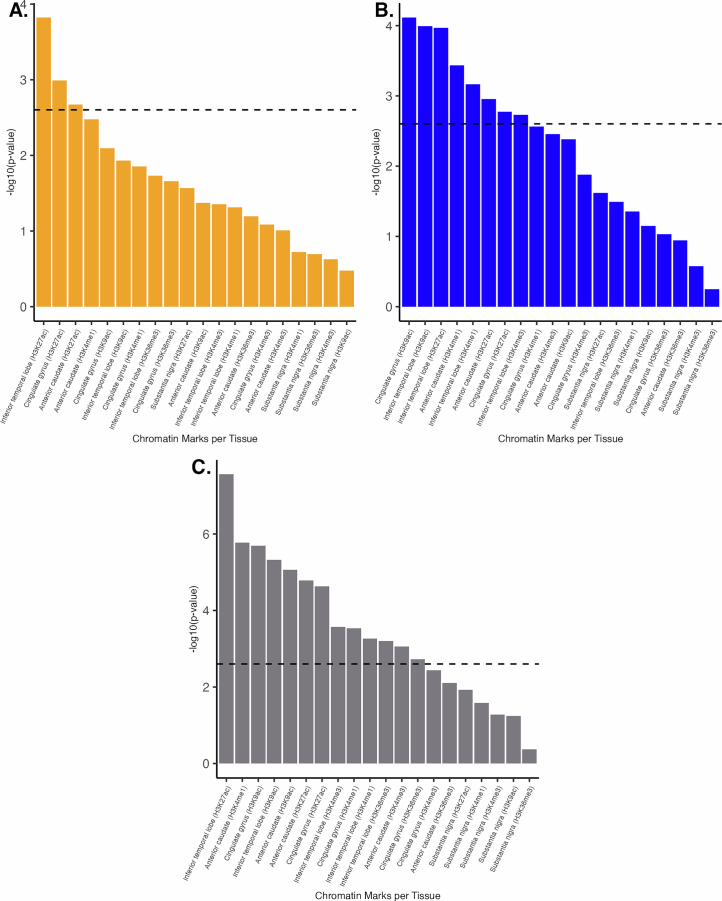
Partitioned heritability estimates for tissue-specific chromatin marks. Partitioned heritability was performed using the tissue-specific chromatin annotations defined by Finucane et al. The −log_10_(*P*-value) for enrichment estimates is indicated on the *y*-axis. The horizontal dotted line indicates significant after Bonferroni correction (*P* < 2.5 × 10^−3^, 20 tests). **a**, Tissue-specific chromatin partitioned heritability estimates in EUR female self-reported stuttering. **b**, Tissue-specific chromatin partitioned heritability estimates in EUR male self-reported stuttering. **c**, Chromatin partitioned heritability estimates in sex-combined EUR self-reported stuttering. Full results are presented in Supplementary Tables 11–13.

Furthermore, we used LDSC to explore whether genes expressed in specific cell or tissue types are enriched for stuttering-associated variants^40^. For brain cell types, we found that our EUR female and sex-combined EUR stuttering results were enriched for neurons (Extended Data Fig. 8 and Supplementary Tables 11 and 12; *P* < 0.017). We then tested for enrichment of effects of stuttering-associated variants in brain tissues previously associated with stuttering in imaging studies (full results shown in Extended Data Fig. 9, Supplementary Tables 12 and 13 and Supplementary Note)^41–48^, and enrichment was further investigated by examining tissue-specific annotations for active chromatin and enhancers (specifically, known histone marks H3K27ac, H3K9ac, H3K4me1, H3K4me3 and H3K36me3) (see Extended Data Fig. 10, Supplementary Tables 11–13 and Supplementary Note for full results).

### Genetic correlation

**Fig. 2:**
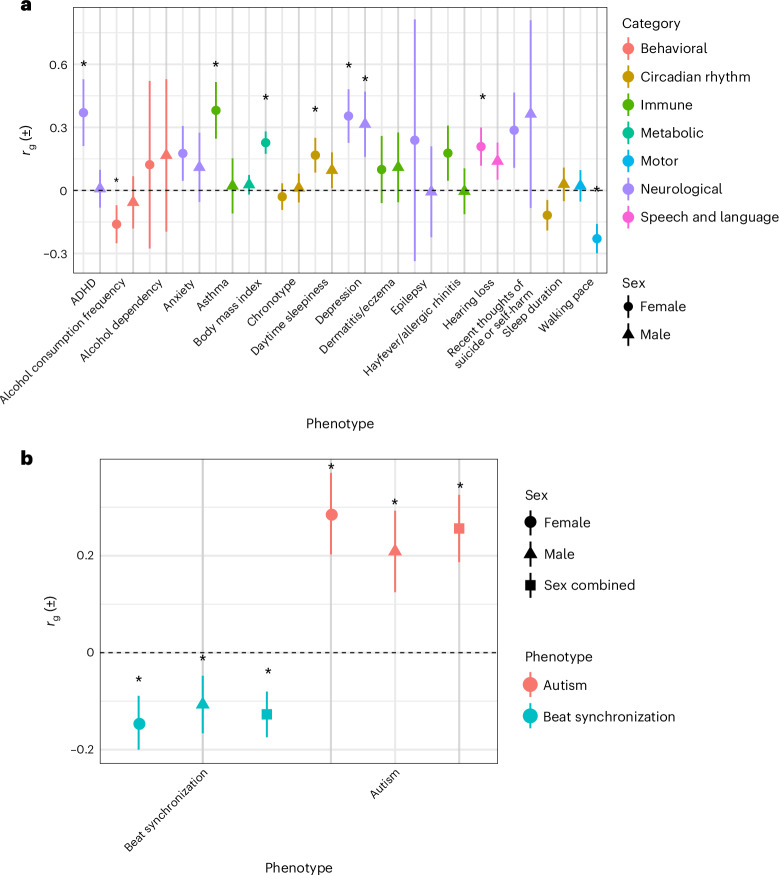
Forest plot showing genetic correlations for stuttering and previously reported comorbid traits. **a**, Ancestry-specific and sex-specific genetic correlations performed for each indicated trait with self-reported stuttering in EUR males and EUR females. Each trait is color-coded according to descriptive category (behavioral, circadian rhythm, immune, metabolic, motor, neurological, speech and language). **b**, Ancestry-specific and sex-specific genetic correlation estimates (EUR male and EUR female) and sex-combined, ancestry-specific (sex-combined EUR) genetic correlation estimates for beat synchronization and autism. Male-specific correlations are designated by triangles, female-specific correlations are designated by circles and sex-combined correlations are designated by squares. Data points represent the correlation coefficients; error bars, SE. Asterisks denote significant genetic correlations by LDSC. See Supplementary Table 15 for full information on traits used for analyses.

To explore whether the genetic architecture of stuttering is shared with previously identified comorbidities, we performed genetic correlation analyses with traits associated with stuttering in the literature (Supplementary Tables 14 and 15)^49–76^. For traits with sex-stratified summary statistics, we observed significant positive genetic correlations, after Bonferroni correction, within both our EUR male and EUR female studies for depression (EUR male, *P* = 6.82 × 10^−5^; EUR female, *P* = 4.53 × 10^−8^; Fig. 2a and Supplementary Table 16). We also observed a number of significant positive genetic correlations in EUR females only: for hearing loss (*P* = 6.50 × 10^−6^), asthma (*P* = 2.91 × 10^−8^), daytime sleepiness (*P* = 6.96 × 10^−8^), attention deficit hyperactivity disorder (ADHD) (*P* = 4.66 × 10^−6^) and body mass index (BMI) (*P* = 4.08 × 10^−17^) as well as a significant negative genetic correlation for frequency of alcohol consumption (*P* = 5.0 × 10^−4^) and walking pace (*P* = 8.81 × 10^−11^; Fig. 2a and Supplementary Table 16). No traits were significantly genetically correlated with stuttering in EUR males exclusively.

For traits without sex-stratified summary statistics, we observed significant positive genetic correlations, after Bonferroni correction, with autism (EUR male, *P* = 1.11 × 10^−6^; EUR female, *P* = 2.05 × 10^−11^; sex-combined EUR, *P* = 4.51 × 10^−13^) and significant negative genetic correlations with beat synchronization (EUR male, *P* = 4.00 × 10^−4^; EUR female, *P* = 3.33 × 10^−7^; sex-combined EUR, *P* = 1.13 × 10^−7^; Fig. 2b and Supplementary Table 16).

### Mendelian randomization

**Fig. 3:**
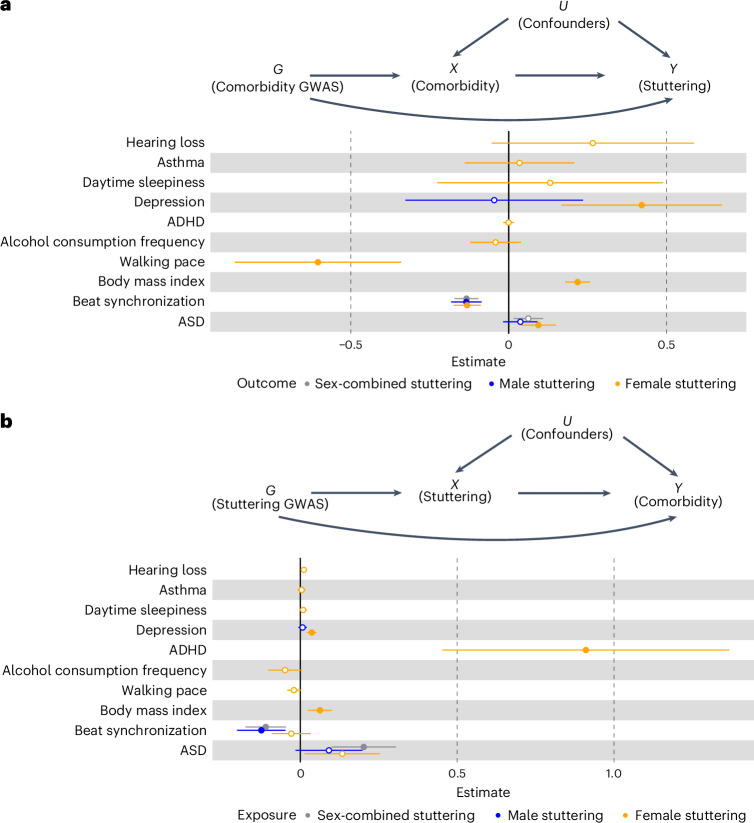
Forest plot showing results of Mendelian randomization analysis for stuttering and previously reported comorbid traits. **a**, Debiased inverse-variance weighted Mendelian randomization analysis estimating causal inference (comorbid trait → stuttering). **b**, Debiased inverse-variance weighted Mendelian randomization analysis estimating causal inference (stuttering → comorbid trait). Data are represented as estimates; error bars, SE. Filled circles are statistically significant (debiased inverse-variance weighted estimator *P* < 3.33 × 10^−3^ after Bonferroni correction for testing 15 associations that were genetically correlated with stuttering). Full information on traits used for analyses can be found in Supplementary Table 15. Full results along with other Mendelian randomization methods can be found in Supplementary Table 17. ASD, autism spectrum disorder.

We performed debiased inverse-variance weighted Mendelian randomization analyses, which handle balanced horizontal pleiotropy and allow for weak instruments^77,78^, to assess the causal relationships between stuttering and previously reported co-occurring traits with significant genetic correlations. Analyses were performed for 11 sex-specific traits and two sex-combined traits genetically correlated with stuttering (after Bonferroni correction) to estimate causal effects between these traits and the self-reported stuttering phenotype captured in our EUR male GWAS, EUR female GWAS or sex-combined EUR meta-analysis. We estimated a significant causal effect of slower walking pace, autism and impaired rhythm on EUR female stuttering (Fig. 3a and Supplementary Table 17). Additionally, we found evidence of bi-directional causal relationships between both higher BMI and depression and EUR female stuttering (Fig. 3a,b and Supplementary Table 17). We also observed a bi-directional relationship between impaired beat synchronization and stuttering in the EUR male and sex-combined EUR analyses (Fig. 3a,b and Supplementary Table 17). Furthermore, we observed a bi-directional relationship between autism and stuttering in the sex-combined EUR analysis (Fig. 3b and Supplementary Table 17). Lastly, in EUR females, we observed significant causal effect estimates of female stuttering on ADHD risk (Fig. 3b and Supplementary Table 17). Results of other Mendelian randomization methods can be found in Supplementary Table 17.

### PRS analyses

**Fig. 4:**
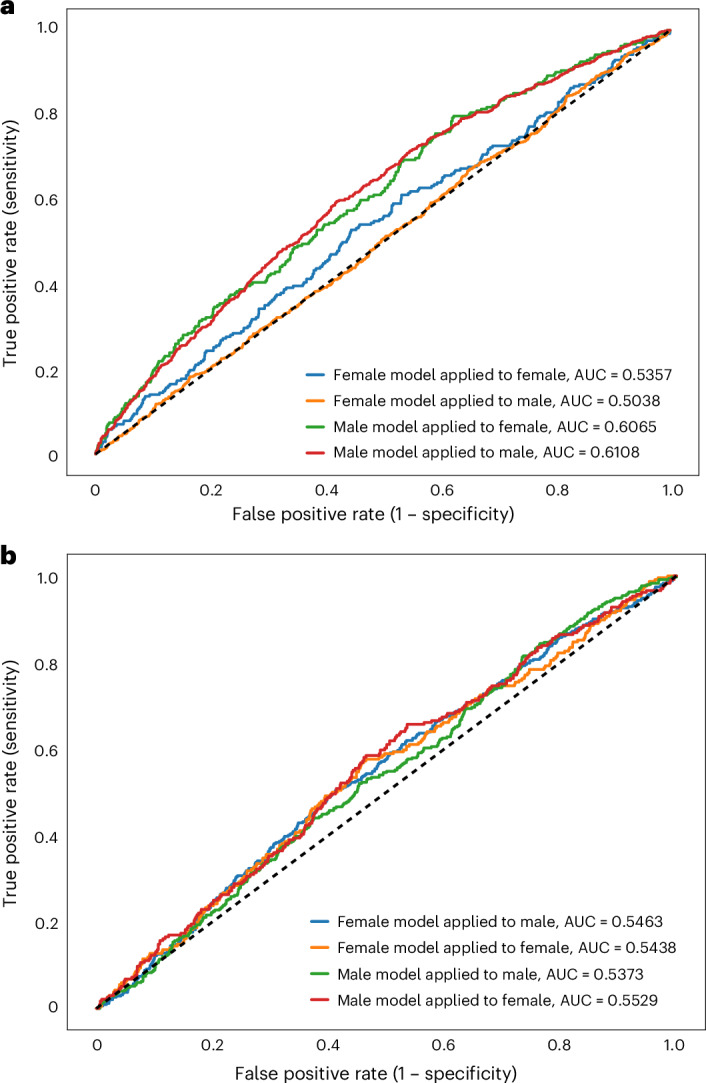
Performance of self-reported stuttering PRS model in independent EUR stuttering datasets. PRS were developed using EUR male or EUR female GWAS results and applied to clinically validated ISP and self-report Add Health subjects, and demonstrate increased stuttering liability within stuttering cases. The model was developed and trained using the default auto-phi shrinkage parameter through PRScs. LD panels were constructed using 1000 Genomes Project phase 3 EUR reference data. **a**, AUC model performance in the ISP cohort. **b**, AUC model performance in the Add Health cohort.

Stuttering PRS were derived from our EUR female and EUR male GWAS, and applied to EUR participants in two independent studies of developmental stuttering: ISP (893 EUR cases and 6,052 EUR controls)^30^ and Add Health (588 EUR cases and 6,621 EUR controls)^32^. Overall, male-specific PRS models out-performed female-specific PRS models (Fig. 4). In particular, the male-specific PRS model derived from the EUR male GWAS demonstrated significant predictive value for both male and female EUR in the ISP (area under the curve (AUC), 0.6108 for male, 0.6065 for female; Fig. 4a and Supplementary Fig. 88) and Add Health (AUC, 0.5373 for male, 0.5529 for female; Fig. 4b and Supplementary Fig. 88). PRS values for cases and controls within the ISP cohort and Add Health study participants can be found in Supplementary Table 18. The lack of large genetic data resources from clinically ascertained participants matching other ancestries prevented validation of PRS derived from non-EUR analyses; however, despite low power, we present preliminary results of cross-ancestry testing of the EUR male and EUR female PRS models in AFR ISP and AFR Add Health study participants (Supplementary Fig. 89).

## Discussion

Our GWAS of self-reported stuttering in males and females is the largest to date, comprising nearly 100,000 cases and over one million controls. Although some complex traits with differences in prevalence by sex or ancestry show similar genetic architectures across these groups^79,80^, we found that there are both shared and distinct genetic effects impacting stuttering risk within sex and/or ancestry groups. Our primary GWAS and secondary meta-analyses identified 57 distinct signals (mapping to 48 unique genes), none of which have been previously reported in stuttering literature. Our estimates of stuttering heritability are in line with SNP-based estimates for other common, complex traits such as insomnia^81^, type 2 diabetes^82^ and beat synchronization^83^. Most prior studies of genetic risk factors for stuttering have explored rare variant effects in pedigrees; however, we find effects that are consistent with high polygenicity, suggesting a genetic architecture similar to other common complex traits^81–83^.

To validate whether the genetic architecture captured by this analysis reflects the genetic etiology of clinically ascertained developmental stuttering, we developed PRS models from the sex-specific EUR GWAS results and applied them to the ISP^30^ and Add Health^32^ stuttering cohorts (which are predominantly EUR samples) for validation. Within the ISP, a clinically ascertained cohort enriched with males and persistent cases of stuttering, the EUR male-derived models showed significant differences in liability scores between stuttering cases and controls in both sexes. By contrast, the EUR female model had significant predictive performance only in EUR females in the ISP. In Add Health, a self-report cohort, both the male and female PRS models significantly predicted case or control status. The difference in the predictive performance between females and males in these two external validation cohorts is notable, with several possible non-mutually exclusive explanations: (1) compared to 23andMe EUR males, the trait captured by 23andMe EUR females is less representative of developmental stuttering; (2) the genetic liability for developmental stuttering varies between males and females and is perhaps confounded by differences in genetic susceptibility to persistent versus recovered stuttering; or (3) genetic variation contributing to developmental stuttering risk may be confounded by horizontal pleiotropy modulated by sex.

The first possibility, that the EUR female stuttering phenotype is not capturing clinically diagnosed developmental stuttering as well as the EUR male stuttering phenotype, may have different possible explanations as well. Prior studies have found that sex-differential participation bias in 23andMe data can lead to spurious genetic associations with sex, particularly in complex traits associated with sex-differential participation bias^38^. For the 57 unique stuttering-associated loci, six were within the SNP windows for loci previously identified to be spuriously associated with sex^38^. We may also be observing sex-specific differences in adult recall accuracy. Females are more likely to recover from stuttering in early childhood^1,13,14^; therefore, the self-report stuttering phenotype may be more impacted by the accuracy of recall of stuttering in childhood in females than in males. There may be other explanations for a difference in how well the self-report phenotype serves as a proxy for clinical diagnosis by sex; for example, female participants in our study may be more likely to report subclinical stuttering than males. Future research will be needed to deconvolute genetic risk factors that are specific to sex and persistence.

To our knowledge, all variants reaching genome-wide significance in our study are novel findings for stuttering. The Open Targets Genetics ‘Variant-to-Gene’ (V2G) pipeline^84,85^ assigned *VRK2* as the most likely functional gene (*r*^2^ between these variants is 0.31 in the CEU population) for one locus spanning the two top hits in EUR male (rs35609938 and rs1040225)^86^. Specifically, rs35609938 occurs downstream of *VRK2* and upstream of *FANCL*, and rs1040225 occurs within either an intronic or upstream region of *VRK2*, depending on isoform (Table 2 and Supplementary Figs. 1 and 2). Interestingly, *FANCL* and *VRK2* were recently implicated in musical beat synchronization^83^. Rhythm perception impairments have been linked to several speech and language conditions, including stuttering^51,52^. Complex rhythm discrimination is below average in adults^51^ and children^50^ who stutter, consistent with the Atypical Rhythm Risk Hypothesis^52^, which posits that those with atypical rhythm may be at risk for developmental speech and language disorders. Clinically, synchronizing speech with external pacing cues, such as a metronome, can often temporarily decrease stuttering disfluencies^50,87–89^. Impairments in rhythm processing may be functionally related to stuttering, and our findings offer the first genetic support for this hypothesis.

Our study also provides additional evidence of the effect for three genes that have been previously reported in family-based studies of stuttering. Although the previously reported variants were not directly genotyped and were too rare for accurate imputation or not present in the 23andMe data, we found three unique signals surpassing Bonferroni significance (see Methods) for the following genes: *GNPTAB*, *GNPTG*^19,27^ and *AP4E1* (ref. ^24^); for *GNPTG*, the sentinel variant in our replication analysis was extremely rare (minor allele frequency of 4.22 × 10^−5^). *AP4E1* interacts with previously reported gene *NAGPA*^24,29^, another gene that has been previously implicated in family studies; together, these results provide modest additional support for the role of these genes in stuttering.

Imaging studies have demonstrated that people who stutter exhibit differences in a variety of brain areas^42,43^, including the frontal cortex^44^, cingulate cortex^41,44^, basal ganglia (caudate, substantia nigra)^45–48^, inferior temporal lobe^90–92^ and cerebellum^41^. Our gene module enrichment analysis (see Supplementary Note) revealed enrichments in the frontal cortex, cortex, anterior cingulate cortex, nucleus accumbens of the basal ganglia and cerebellum. These results, as well as our finding that stuttering-associated variants are involved in neurons and regulation of gene expression in the brain (see Extended Data Figs. 7–10 and Supplementary Tables 11–13) and that more than 20 genes identified in our analyses have been previously implicated in neurological and mental disorder traits, provide additional evidence for the neurological underpinning of stuttering risk^41–48,93,94^. Future follow-up analyses are needed to functionally validate distinct enrichment features in males and females.

Genetic correlation analysis revealed a significant correlation of increased stuttering risk in both EUR males and females with increased risk of depression. In EUR females, we also observed a significant correlation of increased stuttering risk with hearing loss, asthma, daytime sleepiness, ADHD and BMI, and a negative correlation with alcohol consumption and walking pace. The connection between stuttering and sleep is further supported by a recent large-scale multi-ancestry GWAS meta-analysis of short and long sleep duration^95^, which identified five genes associated with sleep duration that overlap with our stuttering findings: *PTPB2, SRPK2*, *KCTD10*, *MMAB* and *SLC39A8*. In addition, for traits without available sex-stratified summary statistics, genetic correlation analysis revealed increased risk of autism and impaired beat synchronization. These genetic correlations, and their respective directions, are largely consistent with previous literature identifying traits comorbid with stuttering^49–76^. However, we recognize that as a volunteer-based study, there are documented biases in UK Biobank that may affect our genetic correlation and Mendelian randomization results, especially when it comes to genetic correlates of behavior, lifestyle and social outcomes^96^. Therefore, increasing representativeness in biobanks and increasing representation and documentation of communication disorders in particular will be paramount for future studies. We regret that many language and motor-related phenotypes that may be correlated with stuttering are underrepresented in the genetics literature, do not have publicly available GWAS summary statistics or did not meet our minimum binary trait case requirement of *n* > 1,000 and were not tested in our genetic correlation analyses.

We also performed Mendelian randomization analyses^77^ to assess causal relationships between stuttering and traits that have been previously reported as co-occurring with stuttering (see Supplementary Table 14). Overall, walking pace and autism were predicted to have a causal effect on stuttering. In addition, stuttering was predicted to have a causal effect on ADHD and autism. Furthermore, we observed significant bi-directional effects between stuttering and BMI. In general, these results bolster previous studies showing preliminary evidence for associations between stuttering and BMI^53^, as well as gross motor coordination^97^. We also observed significant bi-directional causal effects between stuttering and depression. This result is consistent with several studies suggesting that both males and females who stutter report elevated symptoms of depression compared to their fluent counterparts^65,66,98^. Specifically, communication difficulties caused by stuttering can result in feelings of frustration and hopelessness and, along with broader societal stigma toward stuttering, can negatively impact psychological health^61,99,100^. We also observed significant bi-directional effects between stuttering and beat synchronization, or the ability to clap to a beat. This finding is especially compelling considering the role rhythm perception may have in stuttering: as previously mentioned, rhythm discrimination is below average in adults^51^ and children^50^ who stutter, and synchronizing speech with external pacing cues, such as a metronome^88,89^, can temporarily decrease stuttering disfluencies. Interestingly, we observed different significant causal relationships across sexes, which may be a result of observed differences in genetic risk between sexes. The distinct causal pathways in males and females relating stuttering to genetically correlated traits are notable. However, as previously discussed, females are more likely to recover from stuttering than males^13,14^; thus, one limitation of this study is an inability to fully differentiate between effects related to sex and those related to stuttering persistence. Improved granularity of self-report with information regarding stuttering persistence will be necessary to further clarify these effects.

Overall, we leveraged 99,776 cases and over one million controls to identify 57 unique genome-wide significant loci associated with ancestry-specific and sex-specific self-reported stuttering and validated male and female PRS for self-reported stuttering in two independent stuttering datasets. This study provides insight into the genetic contributions to stuttering at the population level, demonstrating that genetic risk is complex, polygenic and dominated by low to modest genetic effects. After decades of progress examining the behavioral, neural and physiological contributions of language, articulation, speech–motor coordination, temperament and emotion to stuttering, the addition of genetic studies may help provide a mechanistic framework for integrating findings across these domains. We demonstrate shared molecular underpinnings between stuttering and other associated traits, including depression, autism and beat synchronization. An unresolved question in the field of stuttering, with lengthy historical speculation, is whether persistent stuttering and recovery from stuttering represent distinct subtypes^101,102^. Thus far, studies have yielded conflicting results with no clear answer^103–105^; the analyses presented here motivate continued research into causal differences between females and males as well as between persistent and recovered stuttering. These findings represent a critical step toward the next era of research for this common, complex, costly and heritable condition.

## Methods

### Ethics

We have complied with all ethical guidelines. All participants provided informed consent to participate in the research. This study has been approved by Vanderbilt IRB (181575 and 180583).

### Studies

#### 23andMe, Inc

GWAS included research participants from 23andMe who self-reported stuttering status through a questionnaire. Cases included participants who answered ‘yes’ (99,776 individuals) to the question ‘Have you ever had a stammer or stutter?’ Controls (1,023,243 individuals) included participants who answered ‘no’ to this same question (Table 1 and Supplementary Table 1). As is common in population-based studies investigating stuttering, our study relies on self-report (see ref. ^106^, in which all but two of the reviewed papers were based on retrospective questionnaire or interview-style surveys)^1,106^. All individuals included in the analyses provided informed consent and answered surveys online according to the 23andMe human subject protocol, which was reviewed and approved by Ethical & Independent Review Services, a private institutional review board (http://www.eandireview.com). Although developmental stuttering is by far the most common form of stuttering, the self-report phenotype in this study may also include other rarer forms of stuttering, such as acquired neurogenic stuttering. Compared to developmental stuttering, acquired stuttering is uncommon in clinical practice^107^. Therefore, although we expect the genetic signatures captured in these analyses to reflect effects for developmental stuttering, we use the general term ‘stuttering’ to describe the self-reported phenotype. Despite these phenotyping considerations, large-scale sample collection through surveys can dramatically increase power—a major limitation in genetic studies of stuttering thus far—and enable the discovery of robust and reproducible effects^108–111^.

#### ISP

We assessed the predictive performance of polygenic scores derived from 23andMe using participants with developmental stuttering from the ISP. Stuttering status in the ISP cohort was confirmed by speech–language pathologists with expertise in stuttering and fluency disorders (see ref. ^30^ for a detailed description of this study and genotyping information).

#### Add Health

We also assessed the predictive performance of polygenic scores derived from 23andMe using participants who self-reported stuttering on an Add Health questionnaire. Add Health is an ongoing, longitudinal study investigating social, behavioral and biological factors that influence health and developmental outcomes from early adolescence through adulthood. Add Health collects general demographics, health survey data, in-home physical data and biological data from all participants (see ref. ^32^ for genotyping information). Stuttering cases were defined as participants who at any point answered ‘yes’ to the following survey question: ‘Do you have a problem with stuttering or stammering?’ All control individuals answered ‘no’ to the above question. Self-reported race/ethnicity was used to group participants.

### Statistical analysis

Eight ancestry-specific and sex-specific genome-wide association analyses were performed to determine variant association with stuttering (Table 1 and Supplementary Table 1). Each GWAS used a logistic regression that assumed an additive model for allelic effects, where *P* is the probability of self-reported stuttering:

$$\begin{array}{l}\mathrm{ln}\left(\displaystyle\frac{P}{1-P}\right)={\beta }_{0}+{\beta }_{{\mathrm{age}}}{\mathrm{age}}+{\beta }_{{\mathrm{pc}}.0}{\mathrm{pc}}.0+{\beta }_{{\mathrm{pc}}.1}{\mathrm{pc}}.1+{\beta }_{{\mathrm{pc}}.2}{\mathrm{pc}}.2\\+{\beta }_{{\mathrm{pc}}.3}{\mathrm{pc}}.3+{\beta }_{{\mathrm{pc}}.4}{\mathrm{pc}}.4+{\beta }_{{\mathrm{platform}}}{\mathrm{platform}}+{\beta }_{{\mathrm{genotype}}}{\mathrm{genotype}}+{\rm{\varepsilon }}\end{array}$$

SNP significance was evaluated by a likelihood ratio test. Results for the X chromosome were computed similarly, in sex-stratified analyses with male genotypes coded as if they were homozygous diploid for the observed allele. Principal components (pc) for each logistic regression model were derived independently for each ancestry, using ~65,000 high-quality genotyped variants present across all five genotyping platforms. Principal components were computed on a subset of participants randomly sampled across all the genotyping platforms (137k, 102k, 1,000k and 360k participants were used for AFR, EAS, EUR and AMR, respectively). Principal components for participants not included in the analysis were obtained by projection, using the eigenvectors from the analysis and the SNP weights. Summary statistics were reported for imputed autosomal and X chromosome variants that were successfully imputed across all platforms (v2, v3, v4 and v5) and reached the following quality control thresholds: average *r*^2^ > 0.5, minimum *r*^2^ > 0.3, batch check *P* > 1 × 10^−50^ and surpassed a minor allele count of 30. Loci in our primary analyses met a two-tiered multiple test correction: first, we applied a false discovery rate threshold of 5% across all autosomal and X chromosome variants; second, a traditional *P* value threshold of 5 × 10^−8^ was applied. Sentinel variants were defined as the most significant variant found within a ±1 Mb window. The top 10,000 SNPs for the ancestry-specific and sex-specific GWAS can be found in Supplementary Data 1–8. For more information on obtaining access to the full dataset, see Data availability.

For our secondary analyses, we performed ancestry-specific, sex-combined meta-analyses, using METAL with the inverse-variance weighted (STDERR command) option^36^ (https://genome.sph.umich.edu/wiki/METAL_Documentation) to meta-analyze our EUR male and EUR female summary statistics, EAS male and EAS female summary statistics, AFR male and AFR female summary statistics and AMR male and AMR female summary statistics. Our sex-combined EUR summary statistics were used for partitioned heritability, genetic correlations and Mendelian randomization analyses.

We aggregated association summary statistics across ancestry-specific association studies using multi-ancestry meta-regression, as implemented in MR-MEGA^37^. Secondary analyses were also performed for the female-specific and the male-specific meta-analyses, and an ancestry-combined, sex-combined meta-analysis. We included three axes of genetic variation as covariates in the ancestry-combined, sex-combined meta-analysis and, given the lower number of contributing analyses in the female-specific and male-specific meta-analyses and limits on the number of possible axes of genetic variation, included one axis as a covariate in the sex-specific analyses. Resulting *P* values were adjusted for genomic control.

### Annotation

The sentinel variant for each genome-wide significant locus was reported for each ancestry-specific and sex-specific study. Genome-wide significance^112^ was defined as *P* < 5 × 10^−8^. Annotated gene(s) for each locus included the predicted functional gene(s) for each loci (when available) according to the Open Targets Genetics V2G pipeline, which combines evidence from molecular quantitative trait loci, chromatin interactions, in silico functional predictions from Ensembl and distance between the variant and gene canonical transcription start site^84,85^. Loci were defined according to independent LD blocks identified in 1000 Genomes reference data, using the matched ancestry reference. Reported sentinel variants represent the variant with the smallest *P* value within each associated region. All reported positional coordinates (chromosome and base pair locations) refer to human genome reference build 37. We also checked for replication (consistency across sex and/or ancestry) of genome-wide significant signals in the primary and secondary GWAS analyses, with replication defined as a Bonferroni-adjusted correction for the number of variants and look-ups performed (*P* < 1.92 × 10^−4^, using a Bonferroni correction; Supplementary Table 4).

### Within-ancestry genetic correlation

To better understand the shared genetic relationship within ancestry groups, we performed genetic correlations within the EUR and EAS ancestries using LDSC^34,35^ and relevant ancestry reference panels. EUR and EAS reference were generated using 1000 Genomes reference data and accessed through LDSC (https://alkesgroup.broadinstitute.org/LDSCORE). Genetic correlations were not performed for the AFR and AMR ancestries, given that LDSC and other methods can produce biased results owing to admixture^113^. Within-ancestry genetic correlation results can be found in Supplementary Table 2. Our within-ancestry genetic correlation results indicate there may be a strong relationship between EUR male and EUR female findings; however, there are differences in genome-wide significant sentinel variants (Table 2). Given that the LDSC approach and other common genetic correlation approaches rely on LD estimates, they can produce biased estimates of correlation when the data being compared have different LD patterns^113^. Therefore, genetic correlation analyses were not performed within AFR and AMR analyses because genetic correlation approaches are biased in the presence of admixture, and correlations were also not tested across ancestry groups^113^.

### Variant effect size concordance analysis

We compared summary statistics from each ancestry-specific and sex-specific GWAS to all others to test for concordance between the summary statistics (Supplementary Table 3)^31^. As we were interested in investigating a broader genetic architecture, rather than including only variants with associations surpassing genome-wide significance, we examined variants with *P* < 0.005. To establish our observed concordance rate, we determined the number of shared genetic variants with the same direction of effect divided by the total number of shared variants between the two datasets being compared. Given that datasets may have different LD patterns, we sought to estimate the expected concordance effects under the null hypothesis, preserving the LD structure observed in the data. To do so, we defined blocks of variants with the same direction of effect within 10 kb windows within each dataset. Next, we performed 25 permutations to randomly assign the direction of effect for these blocks to compute a simulated concordance rate for each permutation (defined as the number of shared genetic variants having the same direction of effect divided by the total number of shared variants between the two datasets). To establish our concordance rate under the null, we took the mean of the 25 simulated concordance rates. The rate of concordance expected under the null was used to compare the observed and expected concordance rates using a binomial *t*-test.

### Effect size heterogeneity

To test for heterogeneity of effect estimates for our 24 sentinel variants from our eight ancestry-specific and sex-specific primary analyses, we used a Wald hypothesis test^114^ to examine the differential effects for each sentinel variant compared to the effects of the other seven primary GWAS. We calculated the test statistic, defined as the quotient of the difference in coefficients and the standard error of this difference. Under the null hypothesis positing equality of coefficients across subgroups, the test statistic conforms approximately to a standard normal distribution, as dictated by the principles of the Wald test. Significance was determined by using a Bonferroni-adjusted *P* value threshold of 2.98 × 10^−4^, correcting for the 24 sentinel variants in our primary analysis multiplied by seven (the number of Wald test comparisons performed).

### SNP heritability and partitioned heritability

Genome-wide SNP-based heritability (*h*^2^) was calculated using summary statistics resulting from the EUR male and EUR female GWAS results using the LDSC software. We used LDSC to estimate liability-scaled *h*^2^, assuming a 10% lifetime population prevalence of stuttering based on the observed frequency of stuttering cases (Table 1 and Supplementary Table 1). EUR LD maps were generated using 1000 Genomes reference data and accessed through LDSC (https://alkesgroup.broadinstitute.org/LDSCORE). Heritability calculations were not estimated for the AFR, AMR and EAS ancestries. For AFR and AMR, heritability estimates are often biased in the presence of admixture^113^. For EAS, given the limited sample size, heritability estimates are likely to be unreliable^39^.

To better understand the types of variation that contribute most to stuttering, we estimated partitioned SNP heritability for our EUR male GWAS, EUR female GWAS and sex-combined EUR meta-analysis, using stratified LDSC^34,35^. LD scores, regression weights and allele frequencies for EUR populations were obtained from https://alkesgroup.broadinstitute.org/LDSCORE. We performed 80 different tests, resulting in a Bonferroni-corrected global significance threshold of *P* < 6.25 × 10^−4^. Partitioning was performed for 52 baseline annotations as previously described^40^. Enrichment was considered significant for *P* < 9.6 × 10^−4^, derived by Bonferroni correction for 52 gene sets.

Next, we tested for enrichment of cell-type-specific and tissue-specific heritability^40^ in our EUR male GWAS, EUR female GWAS and sex-combined EUR meta-analysis, while controlling for the baseline models. Data for the brain cell types used to estimate enrichment of heritability consisted of neuron, astrocyte and oligodendrocyte data from a previous publication^115^. Enrichment tests were considered significant at *P* < 0.017, derived by Bonferroni correction for three gene sets. Gene expression data (computed from GTEx Project data^116^) used to estimate enrichment of heritability consisted of eight brain regions with empirical evidence of relation to stuttering^41–48^. Enrichments were considered significant at *P* < 6.25 × 10^−3^, derived by Bonferroni correction for eight gene sets. Lastly, 20 tissue-specific annotations of active chromatin sequences and enhancers, derived from the Roadmap Epigenomics consortium^117^ and EN-TEx^40,118^ with epigenetic marks of monomethylation (me1), trimethylation (me3) and acetylation (ac) were used to estimate enrichment of heritability. Analyses were performed using epigenetic marks of H3K27ac, H3K9ac, H3K4me1, H3K4me3 and H3K36me3 in four brain regions previously associated with stuttering^41,43,44,46–48^. These active histone marks were considered significant at *P* < 2.5 × 10^−3^, a Bonferroni adjustment for 20 gene sets.

### Validation of loci from the literature

Locus validation analysis was performed using methods detailed in a previous publication^30^; however, the previously calculated effective number of tests was multiplied by eight, given that we checked for validation of signals across our eight ancestry-specific and sex-specific GWAS. As such, the effective number of tests used for our Bonferroni correction represented the number of independent tag SNPs in each gene, with pairwise *r*^2^ < 0.4 multiplied by eight. Gene validation results were Bonferroni-corrected for the effective number of tests in each gene, and we report the variant with the minimum *P* value for each gene.

SNP-based replications looked for replication of the top hits reported in previous publications^30,31^ across all eight ancestry-specific and sex-specific studies. Replication for the 16 sentinel variants identified in ref. ^30^ and replication results for the 11 sentinel variants identified in ref. ^31^ can be found in Supplementary Table 6.

### Stuttering PRS model development

PRS models were trained using the EUR male and EUR female self-reported stuttering GWAS results in PRScs^119^ using a continuous shrinkage before adjusting individual SNP weights for LD and variant significance. Default auto-phi parameters were used in both the male-derived and female-derived models and were not optimized to prevent overfitting. EUR LD reference panels were constructed using the 1000 Genomes Project phase 3 EUR reference and used for EUR and AFR analyses. The male PRS model included 1,024,432 variant predictors, and the female PRS model included 1,024,431 variant predictors. Although the PRS we developed are not clinically useful, here we leveraged these scores to evaluate whether the risk of self-reported stuttering captured in the 23andMe analyses is predictive of risk in clinically validated cohorts. Each model was applied to both the ISP^30^ and Add Health samples^32^, matched according to ancestry and stratified by sex. The ISP testing set included 651 EUR male stuttering cases and 4,264 sex-matched controls, as well as 242 EUR female cases and 1,788 female controls; 48 AFR male stuttering cases and 308 sex-matched controls were included as well as 16 AFR female stuttering cases and 90 sex-matched controls. The Add Health testing set included 352 EUR male stuttering cases and 3,104 sex-matched controls; 236 EUR female cases and 3,517 female controls; 117 AFR male stuttering cases and 847 sex-matched controls were included as well as 107 AFR female stuttering cases and 1,101 sex-matched controls.

Genetic datasets were scored using PLINK (v.1.9)^120^. Genetic liability scores were *z*-score-normalized. Liability score distributions between cases and controls were compared by Student’s two-sample *t*-test. Predictive performance was interpreted using the AUC metric.

### Genetic correlation

The phenotypes chosen for the genetic correlation analyses were either found to be associated with stuttering within electronic health records at Vanderbilt University Medical Center^53^ or previously associated with stuttering in other extant literature (see Supplementary Table 14 for phenotypes and references). Specifically, we compared our EUR male stuttering GWAS results to summary statistics from GWAS of EUR males and our EUR female stuttering GWAS results to summary statistics from GWAS of EUR females for 16 traits encompassing the following categories, for which sex-specific summary statistics were available: behavioral, circadian rhythm, immune, metabolic, motor, neurological and hearing traits. Sex-specific GWAS results were obtained from http://www.nealelab.is/uk-biobank (see Supplementary Table 15). We also explored the genetic correlation of stuttering with two traits for which sex-stratified summary statistics were not available to us: beat synchronization and autism (Supplementary Table 15). For beat synchronization and autism, we compared our EUR male, EUR female and sex-combined EUR summary statistics for each trait to the available sex-combined EUR GWAS summary statistics for the trait. Overall, we performed genetic correlation analyses for 18 traits previously associated with stuttering. A case sample size of >1,000 was required for binary traits. Owing to these constraints, genetic correlation analyses were only performed in the EUR-specific GWAS. All genetic correlation estimates were calculated using LDSC^34,35^. Results were Bonferroni-corrected for the number of tests performed (32 sex-specific analyses and six sex-combined analyses, *P* < 1.32 × 10^−3^). Full results are shown in Supplementary Table 16.

### Mendelian randomization

We performed debiased inverse-variance weighted^77^ Mendelian randomization analysis using the MendelianRandomization R package^121^. Debiased inverse-variance weighted Mendelian randomization can robustly handle weak instrument bias, as well as balanced horizontal pleiotropy^77,78^. We performed Mendelian randomization analysis for all associations that were significantly genetically correlated with stuttering (Fig. 2 and Supplementary Table 16). Specifically, we performed a sex-specific Mendelian randomization analysis for depression, walking pace, BMI, ADHD, beat synchronization and autism, and sex-combined Mendelian randomization analyses for beat synchronization and autism. All of these traits have prior evidence of association with stuttering in the literature^49–76^ (see Supplementary Table 14 for summary). All summary statistics for depression, walking pace, BMI, autism and beat synchronization were obtained from either http://www.nealelab.is/uk-biobank, PGC+iPSYCH data^122^ or 23andMe^83^). Significant variants for each comorbid trait (*P* < 5 × 10^−6^) were filtered to include only those tested in both the comorbid trait GWAS and the stuttering GWAS. Furthermore, independent instrumental SNPs were selected based on LD using the 1000 Genomes Project EUR reference data, retaining SNPs with *r*^2^ < 0.02 in 1,000 kb windows using PLINK^120^. Analysis details and results of other common Mendelian randomization methods are annotated in Supplementary Table 17.

### GWAS Catalog

After filtering the GWAS Catalog^123^ (release date, 2022-21-12) to contain only genome-wide significant loci (*P* < 5.00 × 10^−8^), 48 unique genes from all ancestry-specific and sex-specific GWAS, sex-combined, ancestry-specific meta-analyses, ancestry-combined, sex-specific meta-analyses and ancestry-combined, sex-combined meta-analysis were included in the GWAS Catalog search. Traits associated with these genes in the GWAS Catalog were binned into 22 trait categories (Supplementary Table 19). Our genome-wide significant hits and the associated GWAS Catalog findings can be found in Supplementary Table 19b. The number of unique genes per category can be seen in Supplementary Fig. 90.

### Gene module enrichment

We performed an enrichment test for gene modules using our most likely genes for each identified signal in either the EUR male or EUR female GWAS to identify sets of highly correlated genes (gene modules) that were associated with stuttering risk (see Supplementary Note). The top associated genes were determined for all variants with *P* < 5.00 × 10^−6^ using the Open Targets Genetics V2G pipeline^84,85^. Gene co-expression networks, as previously defined^124^, consist of groups of functionally related genes or ‘modules’. Module enrichments were reported for any gene-tissue-specific analysis with a false discovery rate-adjusted *P* value of <0.05 among any of the 49 available GTEx tissues. Using g:Profiler, we conducted a competitive gene pathway analysis for reported module enrichments, followed by annotation for the biological pathways identified (Supplementary Tables 20–24).

### Colocalization

Bayesian colocalization analysis was performed between our EUR male and EUR female genome-wide significant associations and tissue-specific expression quantitative trait locus signals from GTEx V8 data^116^ using fast enrichment aided colocalization analysis (see Supplementary Note)^125,126^. We examined colocalization solely within regions where a variant was identified as a top hit (Table 2). Evaluated regions included all sentinel variants in either the EUR male, EUR female, AFR male, AMR male or AMR female GWAS, as well as any other variant found in the same LD block. LD blocks were defined based on LD calculated in the 1000 Genomes EUR population reference data^127^. Colocalization analyses were tissue-specific and included all GTEx V8 tissues available. For significant colocalized signals, we report regional colocalization probability (that is, the probability that the association signal is in the shared region across stuttering and expression^128^) of >0.05 (Supplementary Table 25).

### Reporting summary

Further information on research design is available in the Nature Portfolio Reporting Summary linked to this article.

## Online content

Any methods, additional references, Nature Portfolio reporting summaries, source data, extended data, supplementary information, acknowledgements; details of author contributions and competing interests; and statements of data and code availability are available at 10.1038/s41588-025-02267-2.

## Supplementary information

Supplementary Information Supplementary Note and Supplementary Figs. 1–90.

Reporting Summary

Supplementary Table 1 Supplementary Tables 1–25.

Supplementary Data 1 Supplementary Data 1–8.

## Figures and Tables

**Extended Data Fig. 4:**
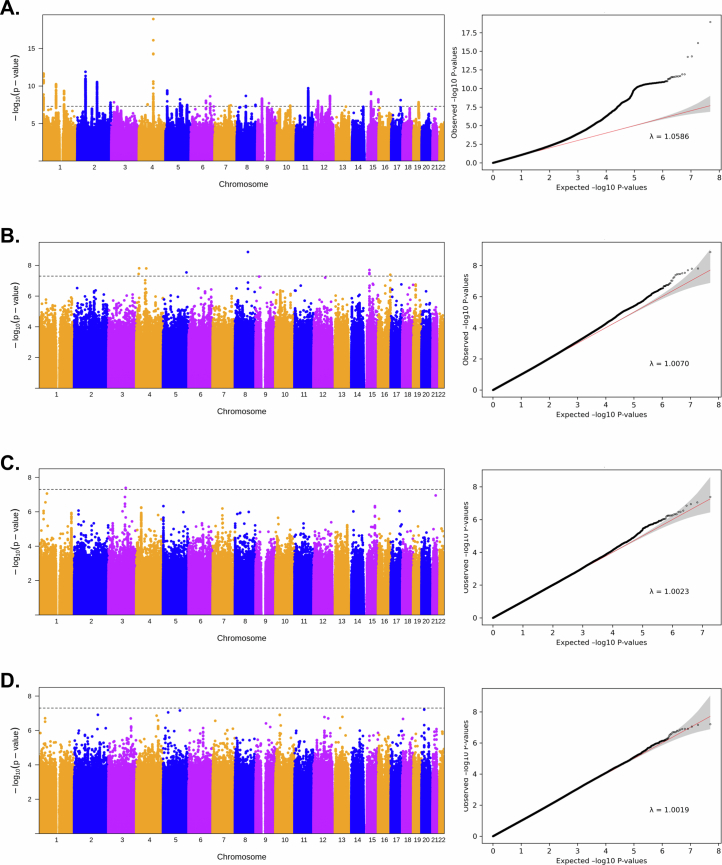
Manhattan and Q-Q plots for the sex-combined meta-analyses. Manhattan plots for sex-combined meta-analyses performed using the inverse variance weighted option in METAL (see Methods). The dotted line represents genome-wide significance (*P* < 5 × 10^−8^). Q-Q plot x-axes represent expected −log_10_(*P*-value), and y-axes represent observed −log_10_(*P*-value) with 95% CI indicated by shaded region. **a**, The meta-analysis includes 944,350 total samples (78,394 self-reported stuttering cases) and 29,566,899 autosomal variants observed across EUR male and EUR female GWAS; λ_gc_ = 1.0586. **b**, The meta-analysis includes 119,393 total samples (13,555 self-reported stuttering cases) and 29,948,846 autosomal variants observed across AMR male and AMR female GWAS; λ_gc_ = 1.0070. **c**, The meta-analysis includes 20,516 total samples (2,339 self-reported stuttering cases) and 10,656,266 autosomal variants observed across EAS male and EAS female GWAS; λ_gc_ = 1.0023. **d**, The meta-analysis includes 38,760 total samples (5,488 self-reported stuttering cases) and 29,705,842 autosomal variants observed across AFR male and AFR female GWAS; λ_gc_ = 1.0019.

**Extended Data Fig. 6:**
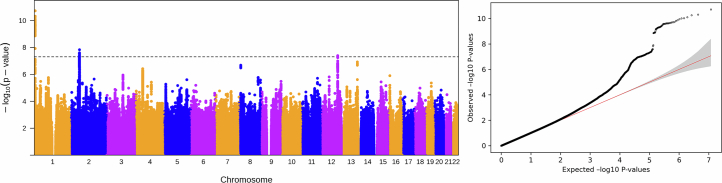
Manhattan and Q-Q plot for the ancestry- and sex-combined meta-analysis. The multi-ancestry meta-regression meta-analysis performed via MR-MEGA (see Methods) included 1,023,243 total samples (99,776 self-reported stuttering cases) and 42,031,853 autosomal variants observed across all eight independent sex and ancestries-specific GWAS. The dotted line represents genome-wide significance (*P* < 5 × 10^−8^). Q-Q plot *x*-axis represents expected −log_10_(*P*-value), and *y*-axis represents observed −log_10_(*P*-value) with 95% CI indicated by shaded region. Note: *P*-values were adjusted for genomic control.

## Data Availability

Ancestry-specific and sex-specific summary statistics of self-reported stuttering will be made available through the 23andMe website to qualified researchers under agreement with 23andMe that protects the privacy of the 23andMe participants. Interested investigators should visit the 23andMe Publication Dataset Access Program at https://research.23andme.com/dataset-access. The top 10,000 SNPs for all primary analyses are provided in Supplementary Data 1–8.

## Code availability

The code for the concordance analysis can be found on GitHub (https://github.com/belowlab/Concordance-Analysis) and Zenodo (10.5281/zenodo.14884575)^129^.
