# Supplementary material for: Large-scale genome-wide analyses of stuttering
Source: Nat Genet. 2025 Jul 28;57(8):1835–47. doi: 10.1038/s41588-025-02267-2 (PMC12339392; doi:10.1038/s41588-025-02267-2)
Supplement: Supplementary file 1 — Supplementary Note and Supplementary Figs. 1–90. [file 41588_2025_2267_MOESM1_ESM.pdf]

nature genetics

Supplementary information https://doi.org/10.1038/s41588-025-02267-2

Large-scale genome-wide analyses of stuttering

In the format provided by the authors and unedited

Supplementary Note

Supplementary Methods

Definition of sex and ancestry groups

First, in analyses that pool data with different genetic structure in which there is unbalanced subgroup size, effects specific to minority subgroups can be masked by the larger groups. Indeed, large-scale studies of many complex traits and behaviors, such as diabetes,[1] lipid traits,[2] height,[3] and smoking status,[4] have shown that research conducted in samples dominated by European ancestry have missed genetic variants that contribute to variation in other ancestry groups. This may be driven by population differences in allele frequencies, effect sizes, and linkage disequilibrium patterns. Second, increasing sample diversity results in substantial improvements in fine-mapping functional variants, assessment of the portability of polygenic prediction, and greater numbers of discovered loci and ancestry-specific variants, even after robust multiple test correction.[2] Finally, stratification provides an opportunity to identify genetic signals that underlie the significant differences in prevalence and recovery rates of stuttering by sex.[5] This study represents the first well-powered exploration of self-reported stuttering in non-European ancestries and by sex, and the best powered analysis of stuttering in European ancestry individuals to date.

For our primary analyses, we determined sex groups genetically and genetic ancestry through an analysis of local ancestry.[6] This analysis phases genomic data into windows of 300 SNPs. For each window, a support vector machine is used to categorize haplotypes into 31 reference populations. Then, support vector machine classifications are inputted into a hidden markov model (HMM), which accounts for switch errors, incorrect assignments, and provides

probabilities for each reference population for each window. To refine HMM probabilities, we used simulated admixed individuals to recalibrate the HMM probabilities so that the reported assignments are consistent with the simulated admixture proportions. The reference population data is derived from public datasets (the Human Genome Diversity Project, HapMap, and 1000 Genomes), as well as 23andMe customers who have reported having four grandparents from the same country.

Genetic Relatedness

Individuals were defined as related if they shared more than 700cM IBD, including regions where the two individuals share either one or both genomic segments IBD. This level of relatedness (roughly 20% of the genome) corresponds approximately to the minimal expected sharing between first cousins in an outbred population. When selecting individuals for case/control phenotype analyses, the selection process is designed to maximize case sample size by preferentially retaining cases over controls. Specifically, if both an individual case and an individual control are found to be related, then the case is retained in the analysis.

Supplementary Results

Testing for heterogeneity of effects in our primary GWAS

To test for heterogeneity of effect estimates for the 24 sentinel variants that reached genome-wide significance in the eight ancestry- and sex-specific analyses, we performed Wald tests to identify significant differences of effect by sex and ancestry compared to the other seven primary GWAS.[7] We found seven loci showed significantly different within ancestry sex-

specific effects, and five loci showed significantly different effects by ancestry (*p*-value < 2.98×10^{-4}, a Bonferroni correction for the 24 sentinel variants discovered in our primary analysis multiplied by seven Wald test comparisons performed for each variant; Extended Data Fig. 3). These findings highlight the distinct sex- and ancestry-specific effects observed in these analyses of self-reported stuttering, a trait which exhibits sex-specific prevalence and recovery rates.

Concordance analysis reveals both shared and distinct genetic effects across sex and ancestry

To compare results across ancestry groups and within recently admixed ancestry groups, we performed a concordance analysis, which compares the direction of effect from variants with *p*-value < 0.005 (see Methods).[8] This analysis reveals both shared and distinct genetic effects by sex and ancestry across the studies (Supplementary Table 3) with cross-ancestry concordance rates ranging from 0.339 to 0.800. The observed concordance and correlation rates may be driven by the comparatively lower power in non-EUR populations or by differences in genetic architecture of stuttering in females and males and across ancestry groups which have not been explored in prior research. Alternatively, the concordance and correlation rates may be driven by differences in genetic architecture of recovered and persistent stuttering which is strongly associated with sex.[9,10] As women are more likely to stop stuttering in childhood,[5,11] recall bias may differentially impact the self-report phenotype in females and males in our analyses, which may also impact observed genetic correlations, and further motivates stratified analyses and formal statistical testing of the variant level differences in effect size.

Consistency of associations between non-overlapping samples

We checked for replication and generalizability for stuttering-associated loci across analyses of the same type (i.e., ancestry-combined, sex-specific meta-analyses) without sample overlap with the discovery sample. To do this, we identified variants that achieved replication-level significance in a non-discovery sample, having adjusted for multiple testing (*p*-value < 1.92×10^{-4}, using a Bonferroni correction for the number of variants and look ups performed; Supplementary Table 4).

In our eight primary ancestry- and sex-specific analyses, one of the 10 EUR male loci replicated in EUR female GWAS (*p*-value < 1.92×10^{-4}; Tables 2 and S4). In the EUR female GWAS, the top locus replicated in the EUR male and AMR female GWAS (Tables 2 and S4). We did not observe any replication for the five signals identified in our non-EUR groups (Tables 2 and Supplementary Table 4), reflecting ancestry-specific effects, diminished power due to sample size, and/or differences in minor allele frequency by population, and highlighting the need for additional studies in these groups to further explore the nature of these effects.

For our sex-combined, ancestry-specific meta-analyses, one of the 28 sex-combined EUR loci replicated in the sex-combined AFR (*p*-value < 1.92×10^{-4}; Tables 2 and S4). We did not observe any replication in the sex-combined AMR and sex-combined EAS genome-wide significant loci (Tables 2 and Supplementary Table 4).

For our ancestry-combined, sex-specific meta-analysis, one of the three male-specific meta-analysis loci replicated in the female-specific meta-analysis (*p*-value < 1.92×10^{-4}; Tables 2

and Supplementary Table 4). We did not observe any replication in the female-specific meta-analysis genome-wide significant loci (Tables 2 and Supplementary Table 4).

Analysis of previously identified stuttering loci in the literature

We considered the evidence of genetic association for the six genes previously implicated as causal genes in family studies by evaluating all variants that passed our QC metrics within each gene across all eight primary GWAS. Since previously described variants from family-based studies[12-15] were not directly genotyped and were too rare to impute, we considered all variants in and around these genes. None of the variants tested in the six genes reached genome-wide significance. We uncovered variant signals reaching replication-wide statistical significance after adjusting for multiple testing (see Methods) for the following genes: *GNPTAB, GNPTG,* and *AP4E1* (Supplementary Table 5). The most strongly associated variant in *GNPTAB*, rs76300806, represents a common indel (EAF = 0.484 in EUR males) found in the 5' UTR region. Top variants for all other genes were rare, intronic variants, including: rs111790048 in *GNPTG*, (EAF = 4.22×10^{-5} in EUR males) which is also close to *TSR3* (~2Kb upstream), and rs565776226 in *AP4E1* (MAF = 0.001 in AMR males).

We also tested for replication of the stuttering-associated variants reported in Shaw *et al*.[8] and Polikowsky *et al*.[16] in our GWAS. No variants reached nominal significance after Bonferroni adjustment. The minimum *p*-value observed in 16 lookups of sentinel variants from Polikowsky *et al*.[16] was rs34919320 (unadjusted *p*-value = 8.20×10^{-3}), and the minimum *p*-value observed in 11 lookups of sentinel variants from Shaw *et al.*[8] was rs115024493 (unadjusted *p*-value = 0.016). Results for all variants are shown in Supplementary Table 6.

Partitioned Heritability

We used LDSC to explore whether genes expressed in specific cell or tissue types are enriched for stuttering-associated variants.[17] For brain cell types, we found that our EUR female and sex-combined EUR stuttering results were enriched for neurons (Extended Data Fig. 8, Supplementary Tables 11-12, p-value < 0.017). We then tested for enrichment of effects of stuttering-associated variants in brain tissues previously associated with stuttering in imaging studies.[18-25] For genes expressed within specific brain tissues, the results from our EUR female analyses were enriched for genes expressed in the cerebellum, frontal cortex, cortex, anterior cingulate cortex, and substantia nigra (Extended Data Fig. 9, Supplementary Table 12, p-value < 6.25×10^{-3}). EUR male stuttering results were enriched for genes expressed in the frontal cortex, cortex, caudate and anterior cingulate cortex (Extended Data Fig. 9, Supplementary Table 13, p-value < 6.25×10^{-3}). Sex-combined EUR stuttering results were enriched for genes expressed in the cortex, frontal cortex, cerebellum, anterior cingulate cortex, caudate, putamen, and substantia nigra (Extended Data Fig. 9, Supplementary Table 11, p-value < 6.25×10^{-3}). Enrichment was further investigated by examining tissue-specific annotations for active chromatin and enhancers (specifically, known histone marks: H3K27ac, H3K9ac, H3K4me1, H3K4me3, and H3K36me3). For tissue-specific annotations of activating histone marks, EUR female stuttering results were enriched for regulation in the inferior temporal lobe, identified by the presence of H3K27ac; cingulate gyrus, identified by the presence of H3K27ac; and anterior caudate, identified by the presence of H3K27ac (Extended Data Fig. 10, Supplementary Table 12, p-value < 2.5×10^{-3}). EUR male stuttering results were enriched for regulation in the cingulate gyrus, identified by the presence of H3K9ac and H3K27ac; inferior temporal lobe,

identified by the presence of H3K9ac, H3K27ac, and H3K4me1; and also, in the anterior caudate, identified by the presence of H3K4me1 and H3K27ac (Extended Data Fig. 10, Supplementary Table 13, p-value < 2.5 x 10^{-3}). Sex-combined EUR stuttering results were enriched for regulation in the anterior caudate and cingulate gyrus, identified by the presence of H3K27ac, H3K9ac, H3K4me1, and H3K4me3; and inferior temporal lobe, identified by the presence of H3K27ac, H3K9ac, H3K4me3, H3K36me3, and H3K4me1 chromatin marks (Extended Data Fig. 10, Supplementary Table 11, p-value < 2.5 x 10^{-3}).

When comparing all partitioned heritability results for EUR male and EUR female stuttering, there are robust similarities; however, some differences emerged. Specifically, EUR male stuttering results were enriched for weak enhancers, repressed markers, and the chromatin mark H3K8ac, a marker for active chromatin. For brain cell types, EUR female stuttering results were enriched for neurons. In addition, EUR male and EUR female stuttering results were enriched for genes expressed in the frontal cortex, cortex, anterior cingulate cortex, and areas of the basal ganglia, while only EUR female stuttering was enriched for genes expressed in the cerebellum. Last, both EUR male and EUR female stuttering results were enriched for active chromatin marks in the inferior temporal lobe, cingulate gyrus, and anterior caudate, while only EUR male showed enrichment for additional active chromatin and enhancer marks within these overlapping regions.

Differences in partitioned heritability enrichments observed between the sexes in neurons and cerebellum (significantly enriched only in female) could be spurious due to differences in power or self-report recall, differences associated with recovery, which occurs at a higher rate in females;[9,10] or could reflect true biological differences in stuttering risk by sex.

Colocalization analysis

To explore colocalization of stuttering-association signals with regulatory variation, we integrated tissue-specific *cis*-eQTL signals from GTEx v.8 data with our stuttering genome-wide association hits and performed Bayesian colocalization[26,27] across all 49 GTEx v.8 tissues.[28] Colocalization analysis between the EUR male GWAS and *cis*-eQTLs in GTEX v.8 identified one region (chr12:109025901-110336719, b37) (Supplementary Table 25) with regional colocalization probability (RCP) > 0.05 across 11 different tissues: Esophagus gastroesophageal junction (RCP = 0.0949), heart left ventricle (RCP = 0.0936), colon transverse (RCP = 0.0924), whole blood (RCP = 0.0811), heart atrial appendage (RCP = 0.0783), adipose visceral omentum (RCP = 0.0725), artery tibial (RCP = 0.0699), colon sigmoid (RCP = 0.0679), cells cultured fibroblasts (RCP = 0.0653), esophagus mucosa (RCP = 0.0612), and artery aorta (RCP = 0.0558). The lead SNP in each tissue was rs12314392, which is an eQTL for *MMAB* and *MVK* (Supplementary Table 25). We did not observe colocalization between genome-wide significant loci in either our EUR female GWAS, AFR male GWAS, AMR male GWAS, or AMR female GWAS with *cis*-eQTLs in GTEx v.8.

Tissue-specific gene module enrichment

We performed a tissue-specific gene module enrichment analysis to further elucidate the gene processes and biology of our stuttering associations, particularly in non-coding regions of the genome. Genetic risk variants are highly enriched in non-coding regions of the genome and can affect gene expression; moreover, genes may be co-expressed and regulate one another's activity in highly organized networks ("gene modules").[29] To facilitate the

identification of gene processes contributing to stuttering risk, we looked for an enrichment of the GTEx tissue-specific gene co-expression networks (i.e. "gene modules") developed by Gerring *et al.*[29] within our stuttering association studies. These analyses highlight the regulatory role of stuttering-associated variation in several tissues including: brain frontal cortex (FDR-corrected *p*-value [q-value] = 2.47×10^{-2} in EUR male, q-value = 3.09×10^{-2} in AFR male), brain anterior cingulate (q-value = 3.28×10^{-2} in EUR male), brain nucleus accumbens of basal ganglia (q-value = 2.54×10^{-3} in EUR female), brain cerebellum (q-value = 9.61×10^{-4} in AFR male, q-value = 1.01×10^{-2} in AMR male), brain cerebellar hemisphere (q-value = 1.39×10^{-3} in AFR male), brain cervical spinal cord (cervical c-1) (q-value = 1.90×10^{-2} in AMR female), brain cortex (q-value = 2.24×10^{-2} in AFR male, q-value = 3.12×10^{-2} in AMR male), esophagus mucosa (q-value = 6.10×10^{-3} in EUR female), esophagus muscularis (q-value = 4.29×10^{-3} in AFR male), adipose visceral omentum (q-value = 2.01×10^{-2} in AMR female), and adipose subcutaneous (q-value = 2.71×10^{-2} in EUR male) (Supplementary Tables 20-24).

Stuttering-associated genes and implicated GWAS Catalog traits

In total, 48 unique genes were mapped via the Open Targets Genetics V2G pipeline[30,31] from the 87 loci identified across all analyses. To assess which traits may be associated with the 48 stuttering genes, we queried the GWAS Catalog (Tables 2-4 and Supplementary Fig. 90).[32] Of our stuttering-associated genes, 36 have been previously associated with obesity/endocrine/metabolic traits, 32 with cardiac/circulatory traits, 30 with lifestyle/behavior traits, 29 with neurological traits, 24 with education, and 21 with mental disorder traits (Supplementary Fig. 90). Trait assignments to trait categories can be found within

Supplementary Table 19a. Full genome-wide association results and respective GWAS catalog results can be found within Supplementary Table 19b.

References

1. Mahajan, A. *et al.* Multi-ancestry genetic study of type 2 diabetes highlights the power of diverse populations for discovery and translation. *Nature Genetics* **54**, 560-572 (2022).
2. Graham, S.E. *et al.* The power of genetic diversity in genome-wide association studies of lipids. *Nature* **600**, 675-679 (2021).
3. Yengo, L. *et al.* A saturated map of common genetic variants associated with human height. *Nature* **610**, 704-712 (2022).
4. Saunders, G.R.B. *et al.* Genetic diversity fuels gene discovery for tobacco and alcohol use. *Nature* **612**, 720-724 (2022).
5. Yairi, E. & Ambrose, N. Epidemiology of stuttering: 21st century advances. *Journal of Fluency Disorders* **38**, 66-87 (2013).
6. Durand, E.Y., Do, C.B., Mountain, J.L. & Macpherson, J.M. Ancestry Composition: A Novel, Efficient Pipeline for Ancestry Deconvolution. (Bioinformatics, 2014).
7. Clogg, C.C., Petkova, E. & Haritou, A. Statistical Methods for Comparing Regression Coefficients Between Models. *American Journal of Sociology* **100**, 1261-1293 (1995).
8. Shaw, D.M. *et al.* Phenome risk classification enables phenotypic imputation and gene discovery in developmental stuttering. *The American Journal of Human Genetics* **108**, 2271-2283 (2021).
9. Singer, C.M., Hessling, A., Kelly, E.M., Singer, L. & Jones, R.M. Clinical Characteristics Associated With Stuttering Persistence: A Meta-Analysis. *Journal of Speech, Language, and Hearing Research* **63**, 2995-3018 (2020).
10. Singer, C.M., Otieno, S., Chang, S.-E. & Jones, R.M. Predicting Persistent Developmental Stuttering Using a Cumulative Risk Approach. *Journal of Speech, Language, and Hearing Research* **65**, 70-95 (2022).
11. Craig, A., Hancock, K., Tran, Y., Craig, M. & Peters, K. Epidemiology of Stuttering in the Community Across the Entire Life Span. *Journal of Speech, Language, and Hearing Research* **45**, 1097-1105 (2002).
12. Kang, C. *et al.* Mutations in the Lysosomal Enzyme–Targeting Pathway and Persistent Stuttering. *New England Journal of Medicine* **362**, 677-685 (2010).
13. Lan, J. *et al.* Association between dopaminergic genes (SLC6A3 and DRD2) and stuttering among Han Chinese. *Journal of Human Genetics* **54**, 457-460 (2009).
14. Mohammadi, H. *et al.* Sex steroid hormones and sex hormone binding globulin levels, CYP17 MSP AI (−34 T:C) and CYP19 codon 39 (Trp:Arg) variants in children with developmental stuttering. *Brain and Language* **175**, 47-56 (2017).
15. Raza, M.H. *et al.* Association between Rare Variants in AP4E1, a Component of Intracellular Trafficking, and Persistent Stuttering. *The American Journal of Human Genetics* **97**, 715-725 (2015).
16. Polikowsky, H.G. *et al.* Population-based genetic effects for developmental stuttering. *Human Genetics and Genomics Advances* **3**, 100073 (2022).
17. Finucane, H.K. *et al.* Heritability enrichment of specifically expressed genes identifies disease-relevant tissues and cell types. *Nature Genetics* **50**, 621-629 (2018).

18. Lu, C. *et al.* The neural substrates for atypical planning and execution of word production in stuttering. *Experimental Neurology* **221**, 146-156 (2010).
19. Chang, S.-E., Garnett, E.O., Etchell, A. & Chow, H.M. Functional and Neuroanatomical Bases of Developmental Stuttering: Current Insights. *The Neuroscientist* **25**, 566-582 (2019).
20. Etchell, A.C., Civier, O., Ballard, K.J. & Sowman, P.F. A systematic literature review of neuroimaging research on developmental stuttering between 1995 and 2016. *Journal of Fluency Disorders* **55**, 6-45 (2018).
21. Liu, J. *et al.* A Functional Imaging Study of Self-Regulatory Capacities in Persons Who Stutter. *PLoS ONE* **9**, e89891 (2014).
22. Neef, N.E. *et al.* Altered morphology of the nucleus accumbens in persistent developmental stuttering. *Journal of Fluency Disorders* **55**, 84-93 (2018).
23. Toyomura, A., Fujii, T. & Kuriki, S. Effect of an 8-week practice of externally triggered speech on basal ganglia activity of stuttering and fluent speakers. *NeuroImage* **109**, 458-468 (2015).
24. Chang, S.E. & Zhu, D.C. Neural network connectivity differences in children who stutter. *Brain* **136**, 3709-3726 (2013).
25. Chang, S.-E., Horwitz, B., Ostuni, J., Reynolds, R. & Ludlow, C.L. Evidence of Left Inferior Frontal–Premotor Structural and Functional Connectivity Deficits in Adults Who Stutter. *Cerebral Cortex* **21**, 2507-2518 (2011).
26. Wen, X., Pique-Regi, R. & Luca, F. Integrating molecular QTL data into genome-wide genetic association analysis: Probabilistic assessment of enrichment and colocalization. *PLOS Genetics* **13**, e1006646 (2017).
27. Pividori, M. *et al.* PhenomeXcan: Mapping the genome to the phenome through the transcriptome. *Science Advances* **6**, eaba2083 (2020).
28. Consortium, G.T. Human genomics. The Genotype-Tissue Expression (GTEx) pilot analysis: multitissue gene regulation in humans. *Science* **348**, 648-60 (2015).
29. Gerring, Z.F., Gamazon, E.R., Derks, E.M. & for the Major Depressive Disorder Working Group of the Psychiatric Genomics, C. A gene co-expression network-based analysis of multiple brain tissues reveals novel genes and molecular pathways underlying major depression. *PLOS Genetics* **15**, e1008245 (2019).
30. Mountjoy, E. *et al.* An open approach to systematically prioritize causal variants and genes at all published human GWAS trait-associated loci. *Nature Genetics* **53**, 1527-1533 (2021).
31. Ghoussaini, M. *et al.* Open Targets Genetics: systematic identification of trait-associated genes using large-scale genetics and functional genomics. *Nucleic Acids Research* **49**, D1311-D1320 (2021).
32. Buniello, A. *et al.* The NHGRI-EBI GWAS Catalog of published genome-wide association studies, targeted arrays and summary statistics 2019. *Nucleic Acids Research* **47**, D1005-D1012 (2019).

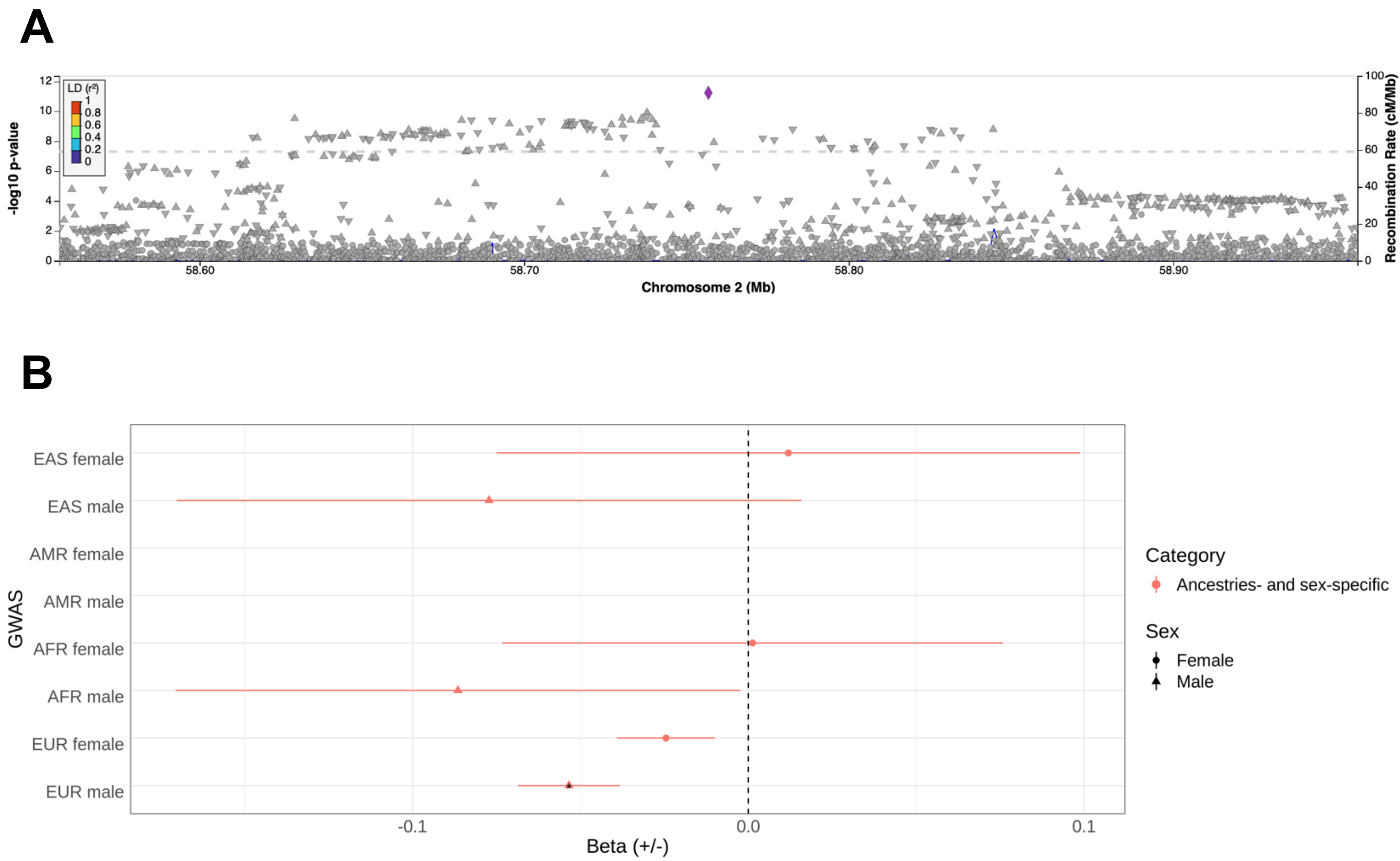

Supplementary Fig 1. Locus zoom and variant effect forest plot of rs35609938. A) Locus zoom plot of EUR male stuttering associations, where the sentinel variant is denoted in purple and surrounding variants are color coded by r2 bin using linkage disequilibrium (LD) generated from 1000 Genomes EUR reference. The x axis represents chromosome position (hg37) with annotated genes found within the region, the y axis represents $\log_{10}$ (*p*-value) of the association between the genetic variant and stuttering. Sentinel variant is located more than 500kb (upstream or downstream) from a protein-coding gene. B) Variant effect forest plots of rs35609938 found within the genetic ancestries of European male (EUR male), European female (EUR female), African male (AFR male), African female (AFR female), East Asian male (EAS male), and East Asian female (EAS female). Variant was not found in Latino/Admixed American male (AMR male), and Latino/Admixed American female (AMR male). Male variant effects are designated by triangles, and female variant effects are designed by circles. Line length indicates standard error for the betas found in the respective GWAS. Variants reaching replicative significance, *p*-value < 8.77×10^{-4} (.05/57 unique loci) are indicated by asterisks.

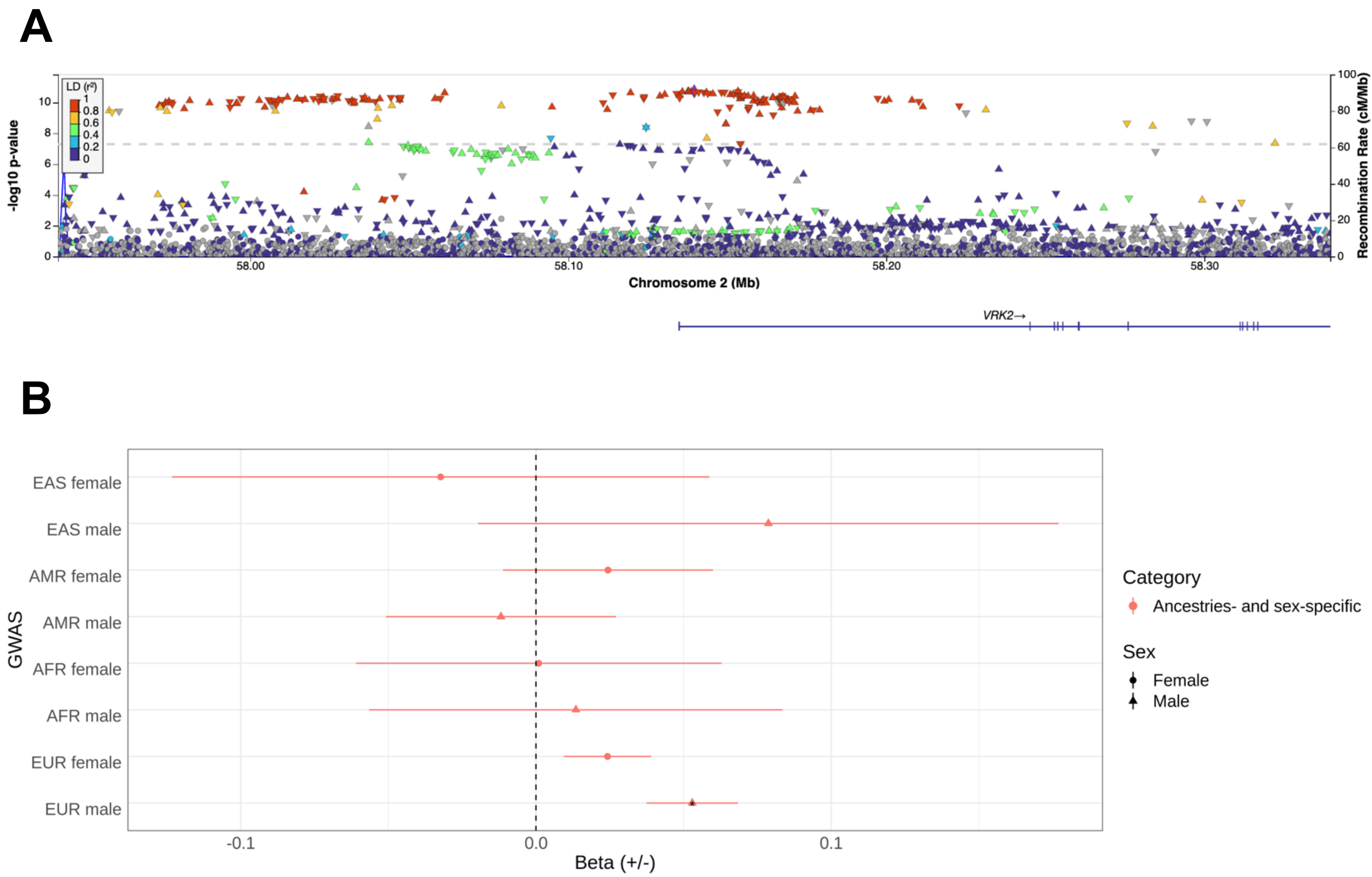

Supplementary Fig 2. Locus zoom and variant effect forest plot of rs1040225. A) Locus zoom plot of EUR male stuttering associations, where the sentinel variant is denoted in purple and surrounding variants are color coded by r2 bin using linkage disequilibrium (LD) generated from 1000 Genomes EUR reference. The x axis represents chromosome position (hg37) with annotated genes found within the region, the y axis represents $\log_{10}$ (*p*-value) of the association between the genetic variant and stuttering. Sentinel variant is a genetic upstream transcript or intronic variant within *VRK2*. B) Variant effect forest plots of rs1040225 found within the genetic ancestries of European male (EUR male), European female (EUR female), African male (AFR male), African female (AFR female), Latino/Admixed American male (AMR male), Latino/Admixed American female (AMR female), East Asian male (EAS male), and East Asian female (EAS female). Male variant effects are designated by triangles, and female variant effects are designed by circles. Line length indicates standard error for the betas found in the respective GWAS. Variants reaching replicative significance, *p*-value < 8.77×10^{-4} (.05/57 unique loci) are indicated by asterisks.

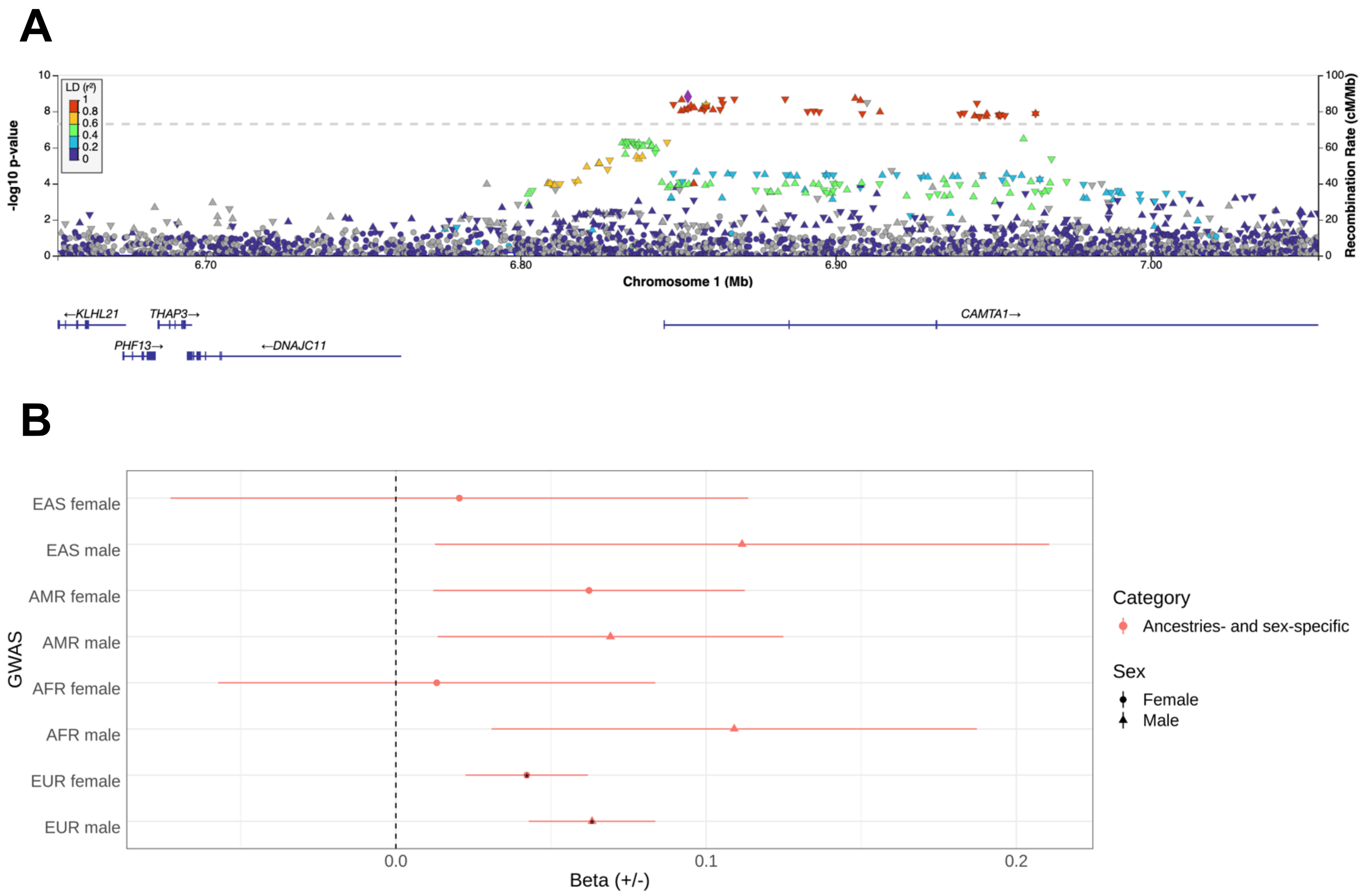

Supplementary Fig 3. Locus zoom and variant effect forest plot of rs34394051. A) Locus zoom plot of EUR male stuttering associations, where the sentinel variant is denoted in purple and surrounding variants are color coded by r2 bin using linkage disequilibrium (LD) generated from 1000 Genomes EUR reference. The x axis represents chromosome position (hg37) with annotated genes found within the region, the y axis represents $\log_{10}$ (*p*-value) of the association between the genetic variant and stuttering. Sentinel variant is a genetic upstream transcript or intronic variant within *CAMTA1*. B) Variant effect forest plots of rs34394051 found within the genetic ancestries of European male (EUR male), European female (EUR female), African male (AFR male), African female (AFR female), Latino/Admixed American male (AMR male), Latino/Admixed American female (AMR female), East Asian male (EAS male), and East Asian female (EAS female). Male variant effects are designated by triangles, and female variant effects are designed by circles. Line length indicates standard error for the betas found in the respective GWAS. Variants reaching replicative significance, *p*-value < 8.77×10^{-4} (.05/57 unique loci) are indicated by asterisks.

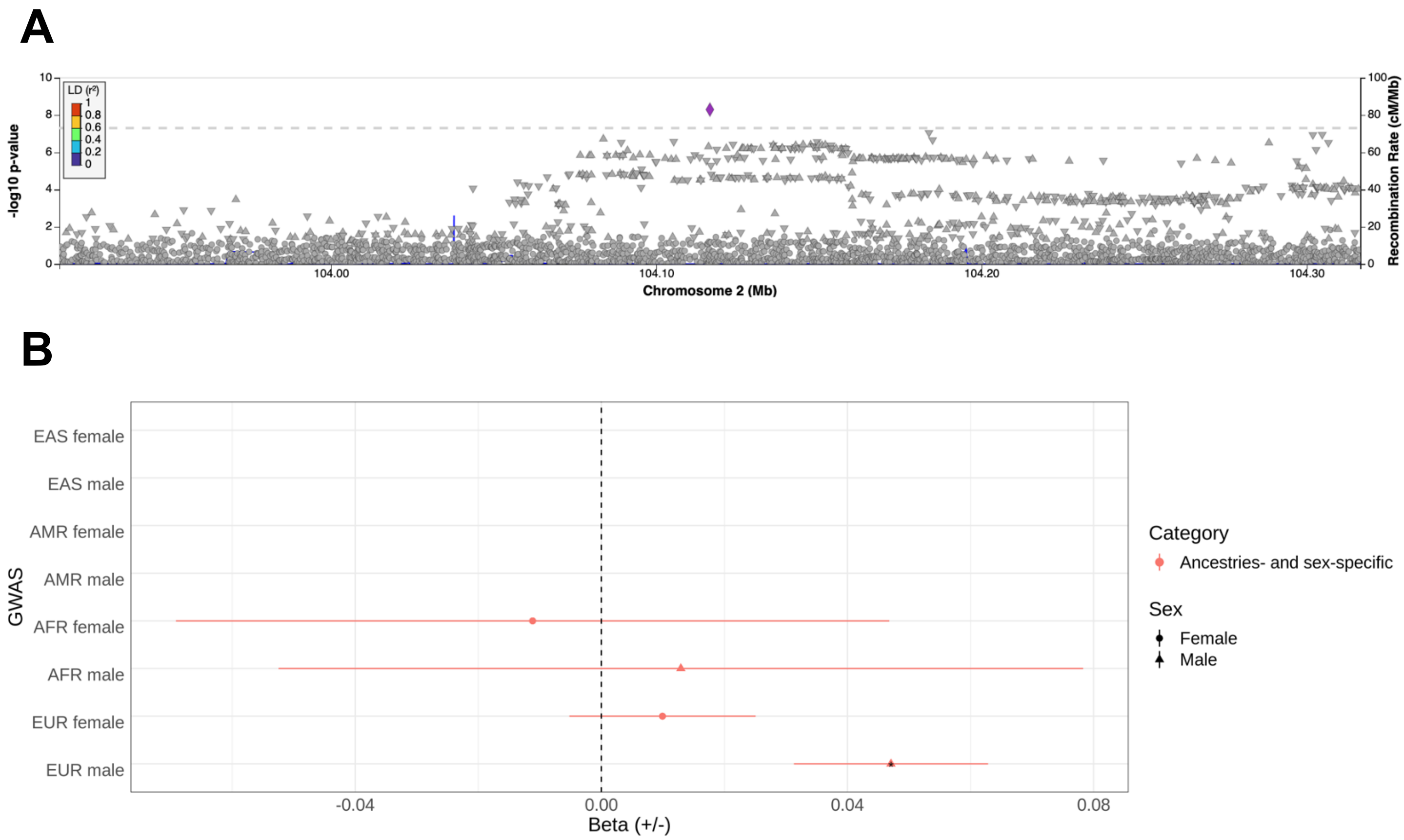

Supplementary Fig 4. Locus zoom and variant effect forest plot of rs545889942. A) Locus zoom plot of EUR male stuttering associations, where the sentinel variant is denoted in purple and surrounding variants are color coded by r2 bin using linkage disequilibrium (LD) generated from 1000 Genomes EUR reference. The x axis represents chromosome position (hg37) with annotated genes found within the region, the y axis represents $\log_{10}$ (*p*-value) of the association between the genetic variant and stuttering. Sentinel variant is located more than 500kb (upstream or downstream) from a protein-coding gene. B) Variant effect forest plots of rs545889942 found within the genetic ancestries of European male (EUR male), European female (EUR female), African male (AFR male), and African female (AFR female). Variant not found in Latino/Admixed American male (AMR male), Latino/Admixed American female (AMR female), East Asian male (EAS male), and East Asian female (EAS female). Male variant effects are designated by triangles, and female variant effects are designed by circles. Line length indicates standard error for the betas found in the respective GWAS. Variants reaching replicative significance, *p*-value < 8.77×10^{-4} (.05/57 unique loci) are indicated by asterisks.

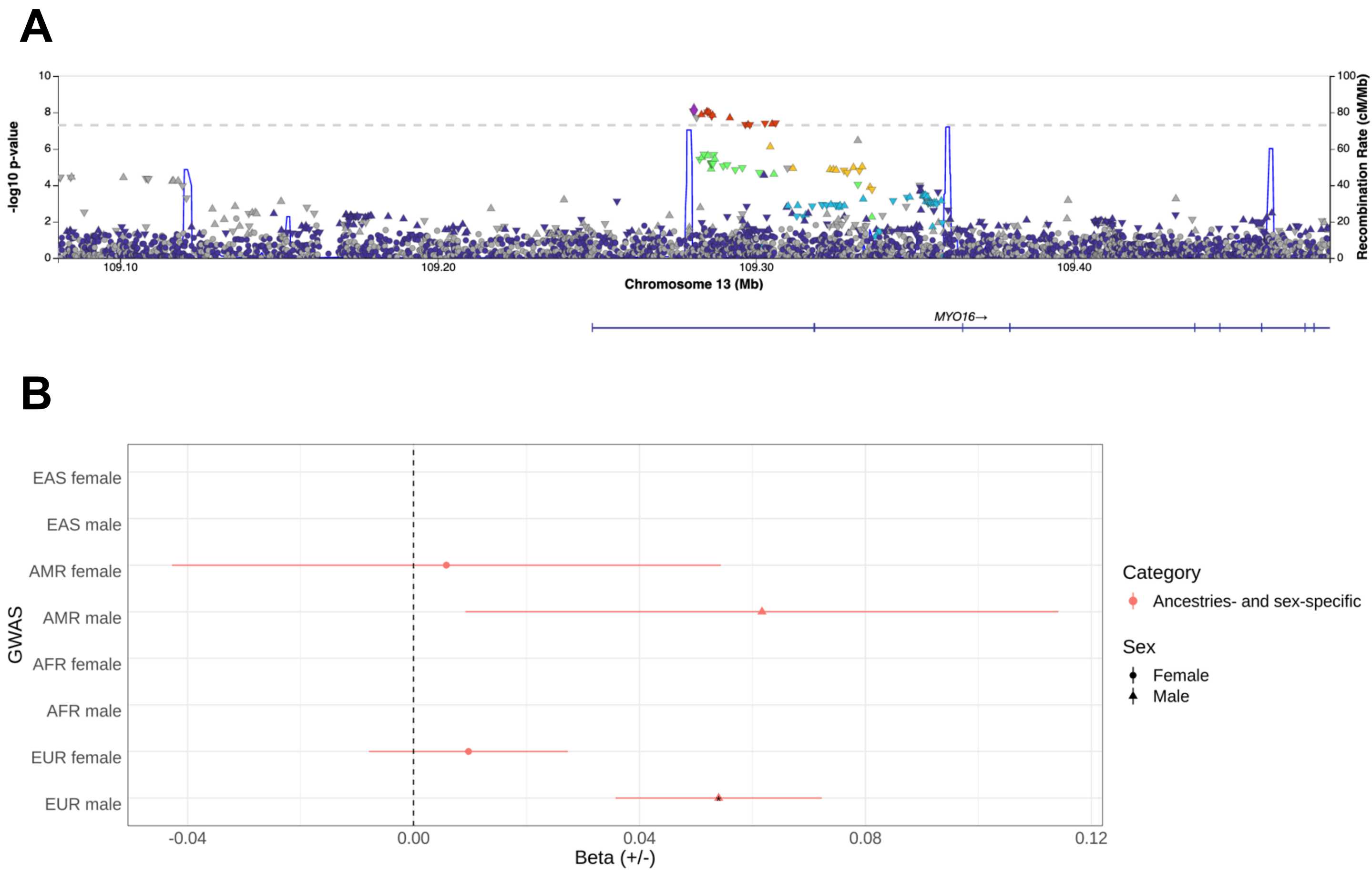

Supplementary Fig 5. Locus zoom and variant effect forest plot of rs72664949. A) Locus zoom plot of EUR male stuttering associations, where the sentinel variant is denoted in purple and surrounding variants are color coded by r2 bin using linkage disequilibrium (LD) generated from 1000 Genomes EUR reference. The x axis represents chromosome position (hg37) with annotated genes found within the region, the y axis represents $\log_{10}$ (*p*-value) of the association between the genetic variant and stuttering. Sentinel variant is a genetic upstream transcript or intronic variant within *MY016*. B) Variant effect forest plots of rs72664949 found within the genetic ancestries of European male (EUR male), European female (EUR female), Latino/Admixed American male (AMR male), and Latino/Admixed American female (AMR female). Variant not found in African male (AFR male), African female (AFR female), East Asian male (EAS male), and East Asian female (EAS female). Male variant effects are designated by triangles, and female variant effects are designed by circles. Line length indicates standard error for the betas found in the respective GWAS. Variants reaching replicative significance, *p*-value < 8.77×10^{-4} (.05/57 unique loci) are indicated by asterisks.

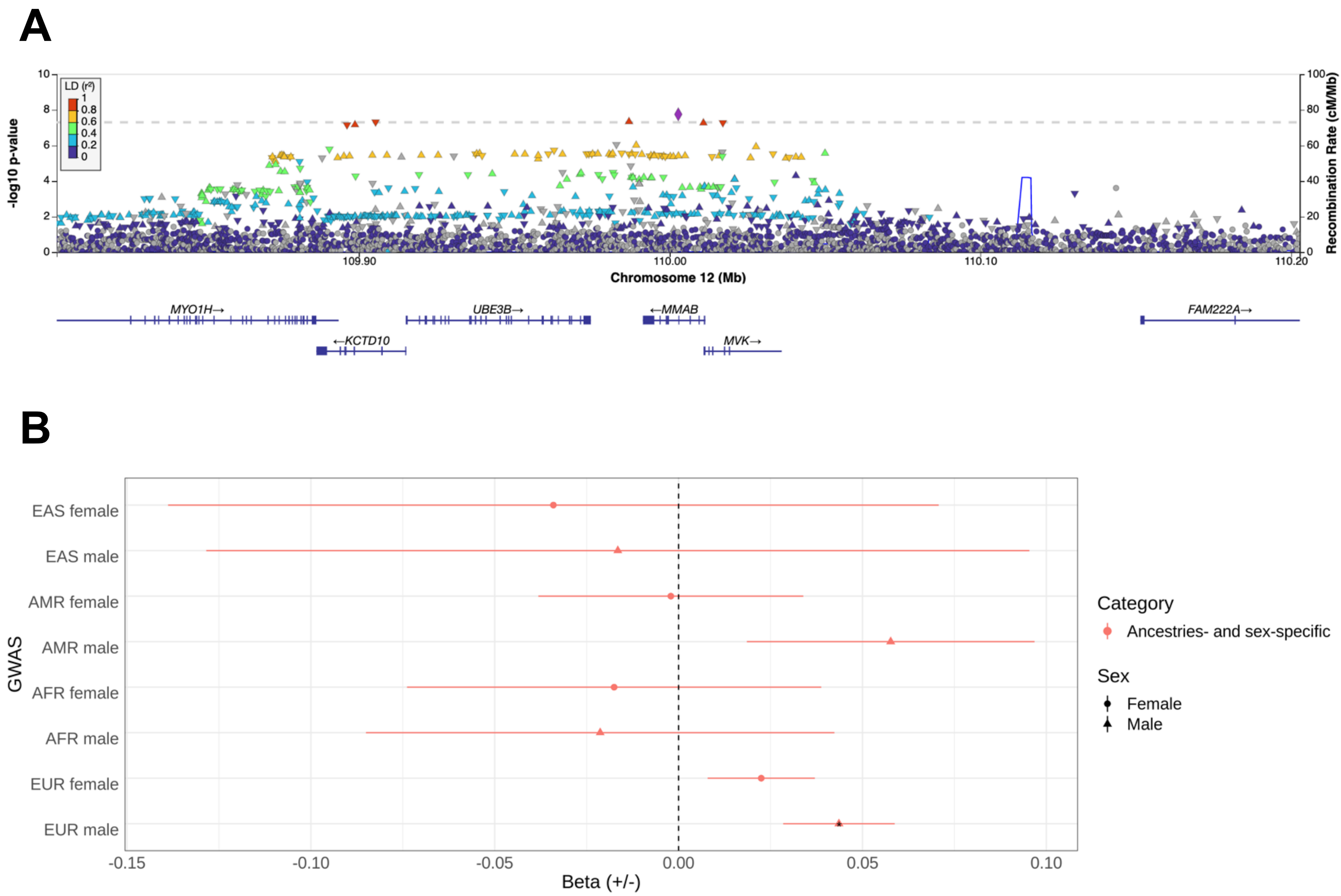

Supplementary Fig 6. Locus zoom and variant effect forest plot of rs10850379. A) Locus zoom plot of EUR male stuttering associations, where the sentinel variant is denoted in purple and surrounding variants are color coded by r2 bin using linkage disequilibrium (LD) generated from 1000 Genomes EUR reference. The x axis represents chromosome position (hg37) with annotated genes found within the region, the y axis represents $\log_{10}$ (*p*-value) of the association between the genetic variant and stuttering. Sentinel variant is a transcript or intronic variant within *MMAB*. B) Variant effect forest plots of rs10850379 found within the genetic ancestries of European male (EUR male), European female (EUR female), African male (AFR male), African female (AFR female), Latino/Admixed American male (AMR male), Latino/Admixed American female (AMR female), East Asian male (EAS male), and East Asian female (EAS female). Male variant effects are designated by triangles, and female variant effects are designed by circles. Line length indicates standard error for the betas found in the respective GWAS. Variants reaching replicative significance, *p*-value < 8.77×10^{-4} (.05/57 unique loci) are indicated by asterisks.

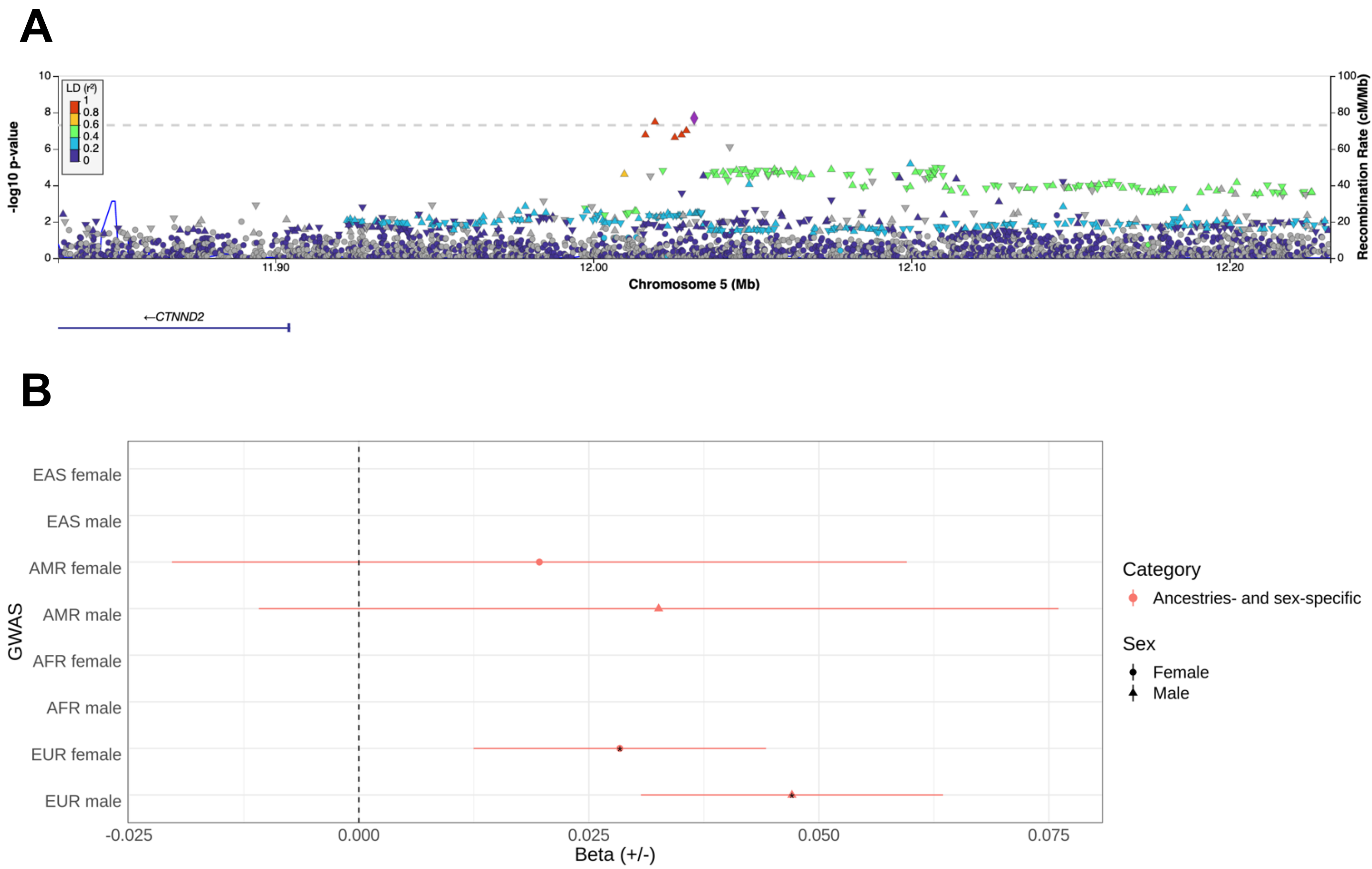

Supplementary Fig 7. Locus zoom and variant effect forest plot of rs62337988. A) Locus zoom plot of EUR male stuttering associations, where the sentinel variant is denoted in purple and surrounding variants are color coded by r2 bin using linkage disequilibrium (LD) generated from 1000 Genomes EUR reference. The x axis represents chromosome position (hg37) with annotated genes found within the region, the y axis represents $\log_{10}$ (*p*-value) of the association between the genetic variant and stuttering. Sentinel variant is a genetic upstream transcript or intronic variant within *MY016*. B) Variant effect forest plots of rs72664949 found within the genetic ancestries of European male (EUR male), European female (EUR female), Latino/Admixed American male (AMR male), and Latino/Admixed American female (AMR female). Variant not found in African male (AFR male), African female (AFR female), East Asian male (EAS male), and East Asian female (EAS female). Male variant effects are designated by triangles, and female variant effects are designed by circles. Line length indicates standard error for the betas found in the respective GWAS. Variants reaching replicative significance, *p*-value < 8.77×10^{-4} (.05/57 unique loci) are indicated by asterisks.

A

B

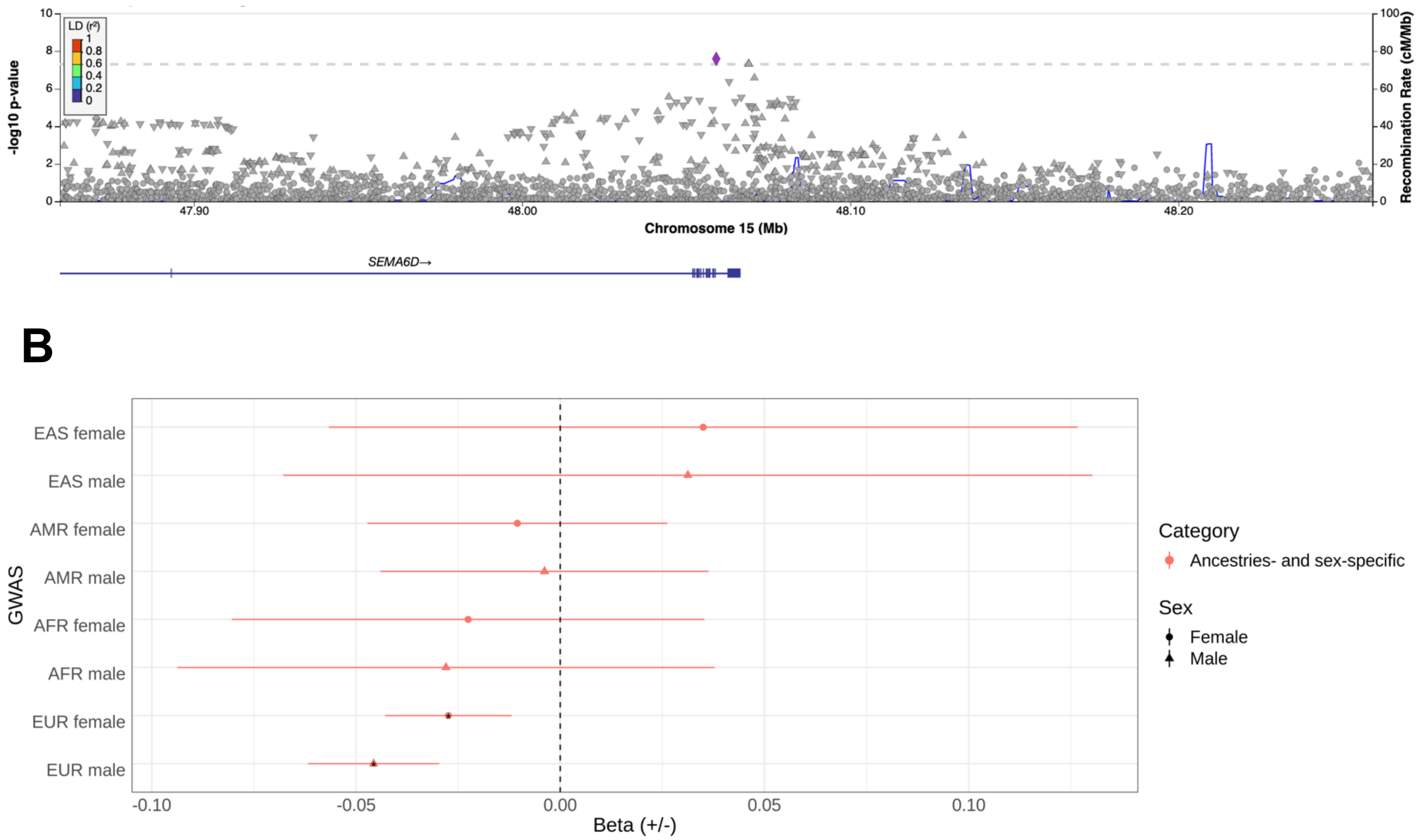

Supplementary Fig 8. Locus zoom and variant effect forest plot of rs11353659. A) Locus zoom plot of EUR male stuttering associations, where the sentinel variant is denoted in purple and surrounding variants are color coded by r2 bin using linkage disequilibrium (LD) generated from 1000 Genomes EUR reference. The x axis represents chromosome position (hg37) with annotated genes found within the region, the y axis represents $\log_{10}$ (*p*-value) of the association between the genetic variant and stuttering. Sentinel variant is a genetic downstream transcript or intronic variant within *SEMA6D*. B) Variant effect forest plots of rs11353659 found within the genetic ancestries of European male (EUR male), European female (EUR female), African male (AFR male), African female (AFR female), Latino/Admixed American male (AMR male), Latino/Admixed American female (AMR female), East Asian male (EAS male), and East Asian female (EAS female). Male variant effects are designated by triangles, and female variant effects are designed by circles. Line length indicates standard error for the betas found in the respective GWAS. Variants reaching replicative significance, *p*-value < 8.77×10^{-4} (.05/57 unique loci) are indicated by asterisks.

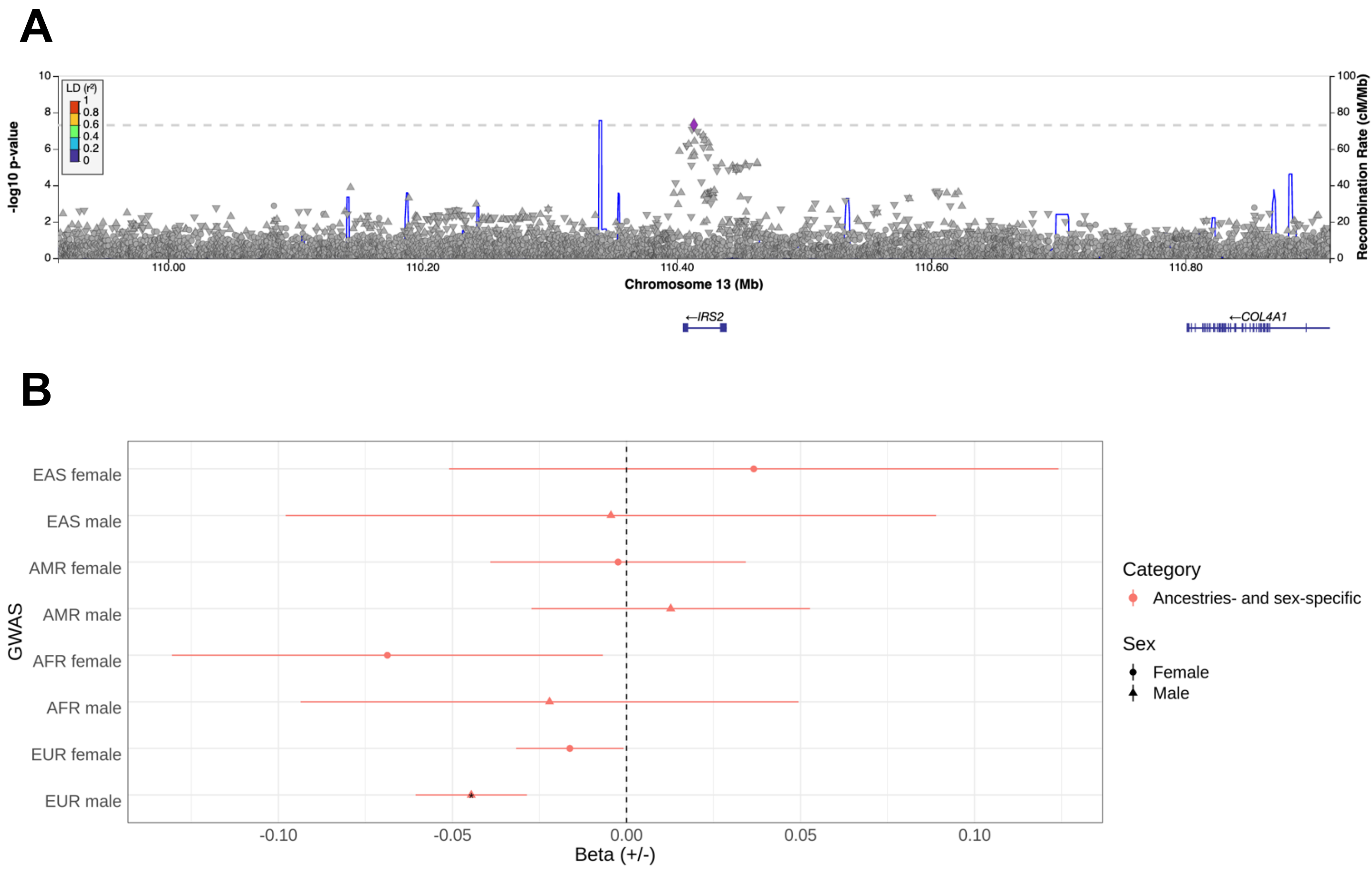

Supplementary Fig 9. Locus zoom and variant effect forest plot of rs58120907. A) Locus zoom plot of EUR male stuttering associations, where the sentinel variant is denoted in purple and surrounding variants are color coded by r2 bin using linkage disequilibrium (LD) generated from 1000 Genomes EUR reference. The x axis represents chromosome position (hg37) with annotated genes found within the region, the y axis represents $\log_{10}$ (*p*-value) of the association between the genetic variant and stuttering. Sentinel variant is an intronic variant within *IRS2*. B) Variant effect forest plots of rs58120907 found within the genetic ancestries of European male (EUR male), European female (EUR female), African male (AFR male), African female (AFR female), Latino/Admixed American male (AMR male), Latino/Admixed American female (AMR female), East Asian male (EAS male), and East Asian female (EAS female). Male variant effects are designated by triangles, and female variant effects are designed by circles. Line length indicates standard error for the betas found in the respective GWAS. Variants reaching replicative significance, *p*-value < 8.77×10^{-4} (.05/57 unique loci) are indicated by asterisks.

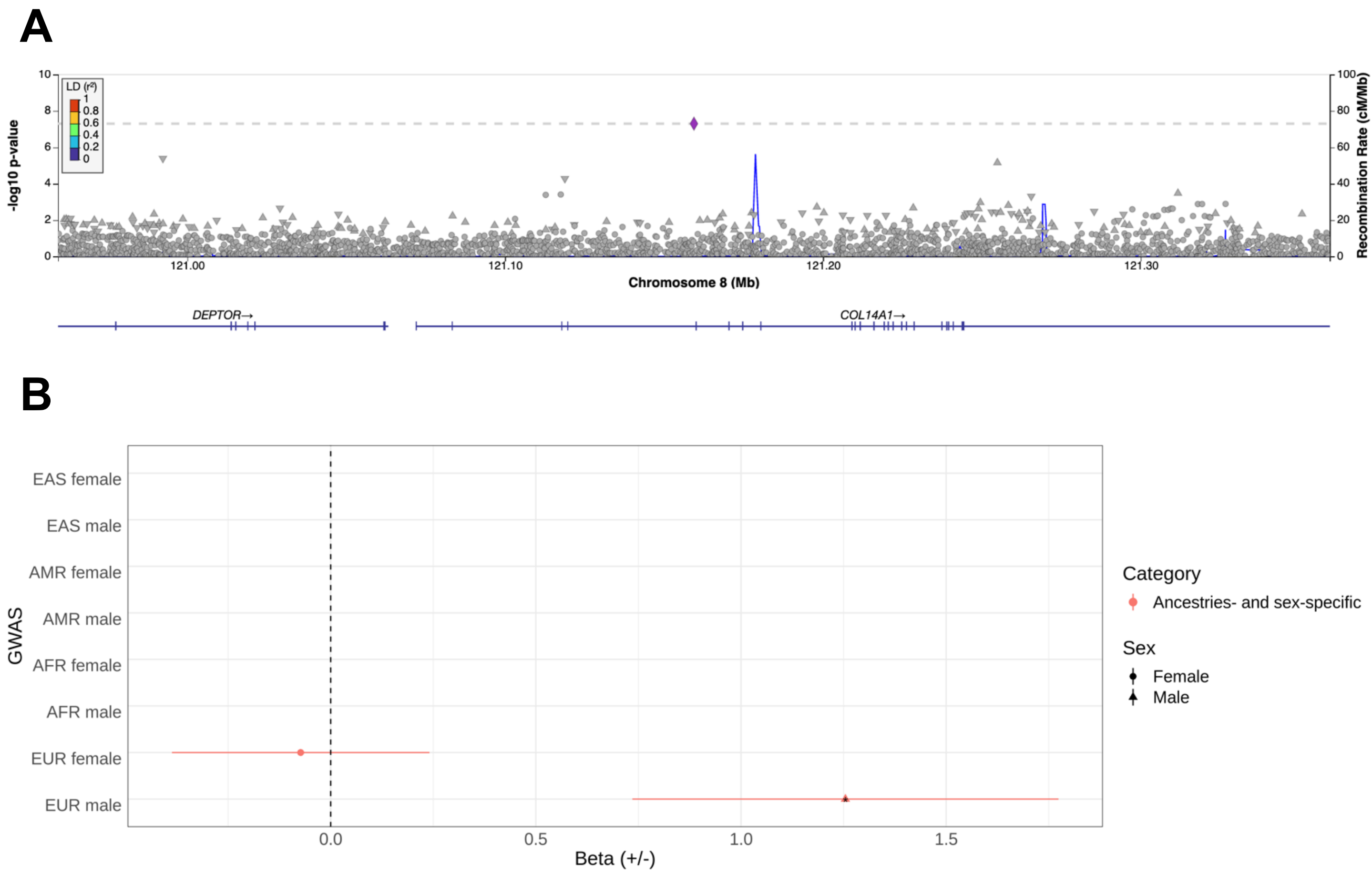

Supplementary Fig 10. Locus zoom and variant effect forest plot of rs558002155. A) Locus zoom plot of EUR male stuttering associations, where the sentinel variant is denoted in purple and surrounding variants are color coded by r2 bin using linkage disequilibrium (LD) generated from 1000 Genomes EUR reference. The x axis represents chromosome position (hg37) with annotated genes found within the region, the y axis represents $\log_{10}$ (*p*-value) of the association between the genetic variant and stuttering. Sentinel variant is an intronic variant within *COL14A1*. B) Variant effect forest plots of rs558002155 found within the genetic ancestries of European male (EUR male), and European female (EUR female). Variant not found in African male (AFR male), African female (AFR female), Latino/Admixed American male (AMR male), Latino/Admixed American female (AMR female), East Asian male (EAS male), and East Asian female (EAS female). Male variant effects are designated by triangles, and female variant effects are designed by circles. Line length indicates standard error for the betas found in the respective GWAS. Variants reaching replicative significance, *p*-value < 8.77×10^{-4} (.05/57 unique loci) are indicated by asterisks.

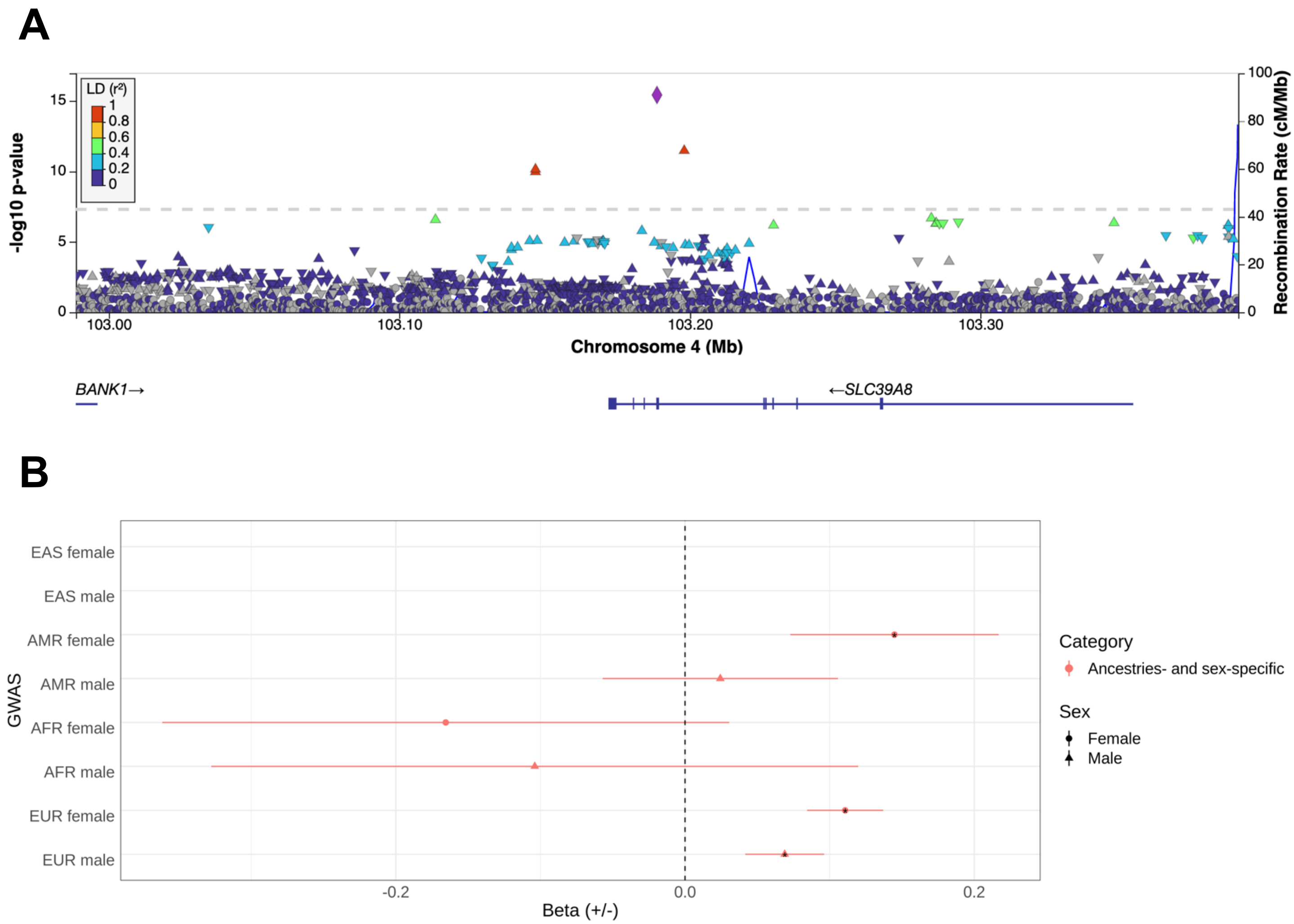

Supplementary Fig 11. Locus zoom and variant effect forest plot of rs13107325. A) Locus zoom plot of EUR female stuttering associations, where the sentinel variant is denoted in purple and surrounding variants are color coded by r2 bin using linkage disequilibrium (LD) generated from 1000 Genomes EUR reference. The x axis represents chromosome position (hg37) with annotated genes found within the region, the y axis represents $\log_{10}$ (*p*-value) of the association between the genetic variant and stuttering. Sentinel variant is a missense variant within *SLC39A8*. B) Variant effect forest plots of rs13107325 found within the genetic ancestries of European male (EUR male), European female (EUR female), African male (AFR male), African female (AFR female), Latino/Admixed American female (AMR female), and Latino/Admixed American male (AMR male). Variant not found in East Asian male (EAS male), and East Asian female (EAS female). Male variant effects are designated by triangles, and female variant effects are designed by circles. Line length indicates standard error for the betas found in the respective GWAS. Variants reaching replicative significance, *p*-value < 8.77×10^{-4} (.05/57 unique loci) are indicated by asterisks.

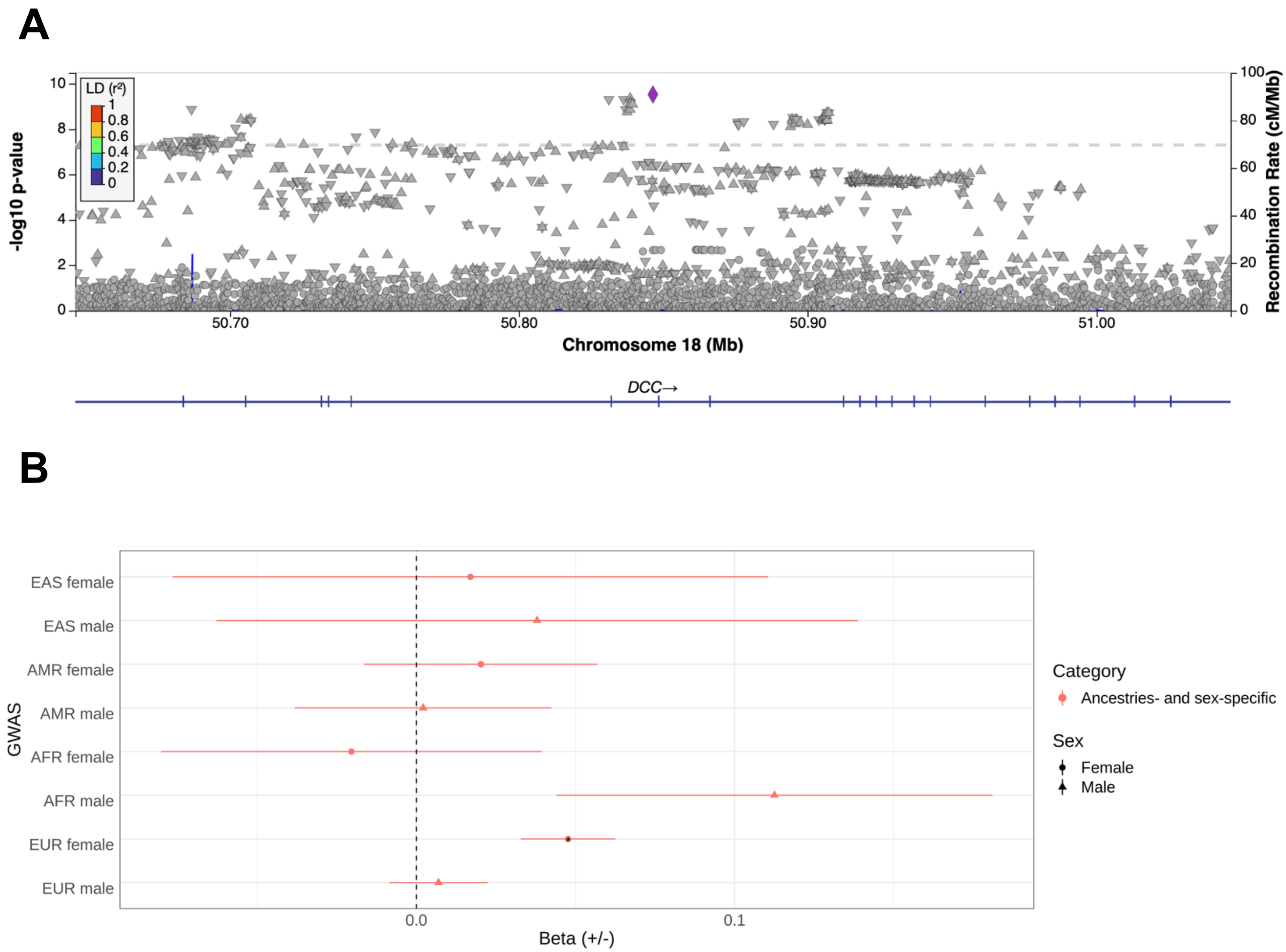

Supplementary Fig 12. Locus zoom and variant effect forest plot of rs572319557. A) Locus zoom plot of EUR female stuttering associations, where the sentinel variant is denoted in purple and surrounding variants are color coded by r2 bin using linkage disequilibrium (LD) generated from 1000 Genomes EUR reference. The x axis represents chromosome position (hg37) with annotated genes found within the region, the y axis represents $\log_{10}$ (*p*-value) of the association between the genetic variant and stuttering. Sentinel variant is an intronic variant within *DCC*. B) Variant effect forest plots of rs572319557 found within the genetic ancestries of European male (EUR male), European female (EUR female), African male (AFR male), African female (AFR female), Latino/Admixed American male (AMR male), Latino/Admixed American female (AMR female), East Asian male (EAS male), and East Asian female (EAS female). Male variant effects are designated by triangles, and female variant effects are designed by circles. Line length indicates standard error for the betas found in the respective GWAS. Variants reaching replicative significance, *p*-value < 8.77×10^{-4} (.05/57 unique loci) are indicated by asterisks.

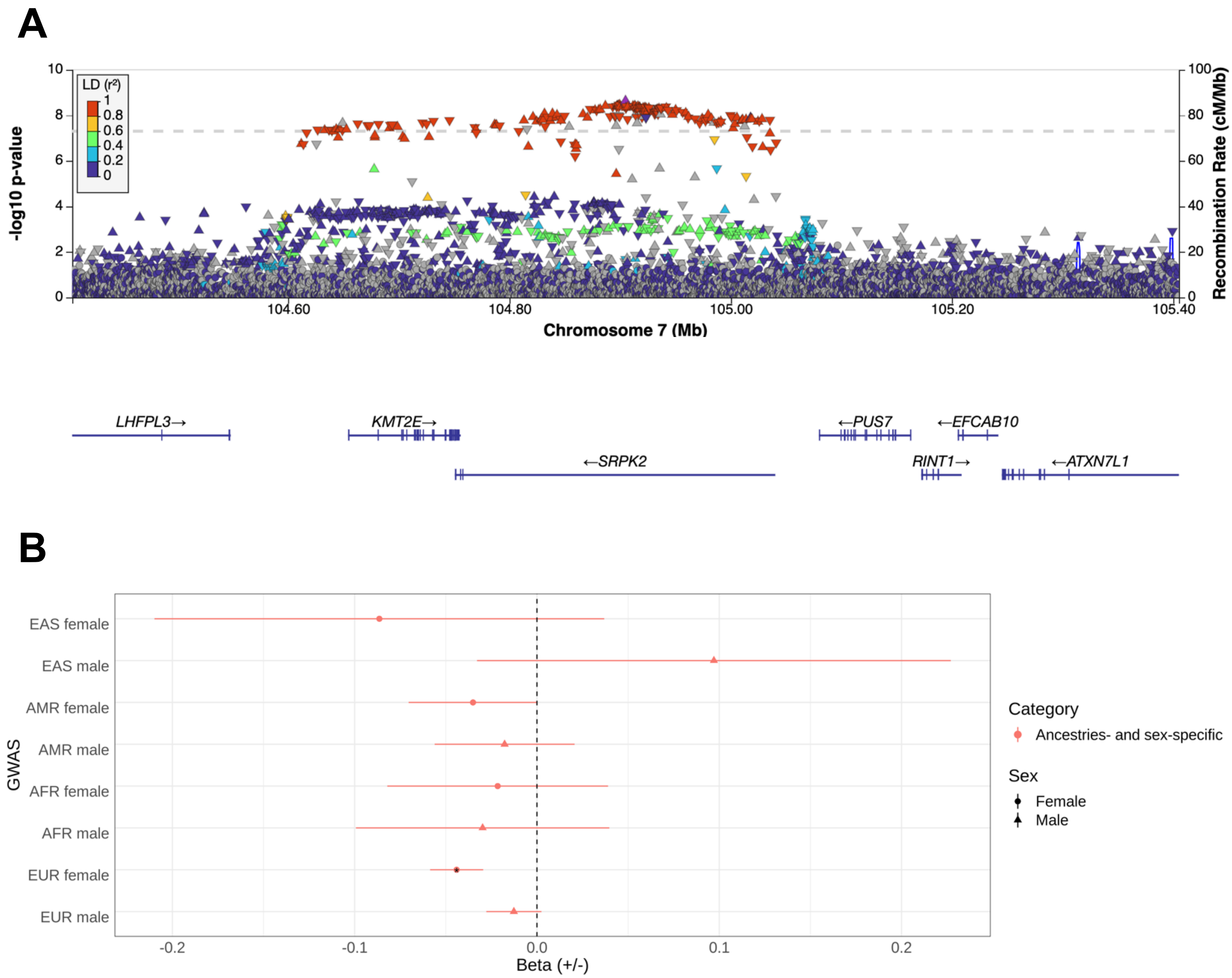

Supplementary Fig 13. Locus zoom and variant effect forest plot of rs3801279. A) Locus zoom plot of EUR female stuttering associations, where the sentinel variant is denoted in purple and surrounding variants are color coded by r2 bin using linkage disequilibrium (LD) generated from 1000 Genomes EUR reference. The x axis represents chromosome position (hg37) with annotated genes found within the region, the y axis represents $\log_{10}$ (*p*-value) of the association between the genetic variant and stuttering. Sentinel variant is an intronic variant within *SRPK2*. B) Variant effect forest plots of rs3801279 found within the genetic ancestries of European male (EUR male), European female (EUR female), African male (AFR male), African female (AFR female), Latino/Admixed American male (AMR male), Latino/Admixed American female (AMR female), East Asian male (EAS male), and East Asian female (EAS female). Male variant effects are designated by triangles, and female variant effects are designed by circles. Line length indicates standard error for the betas found in the respective GWAS. Variants reaching replicative significance, *p*-value $< 8.77 \times 10^{-4}$ (.05/57 unique loci) are indicated by asterisks.

A

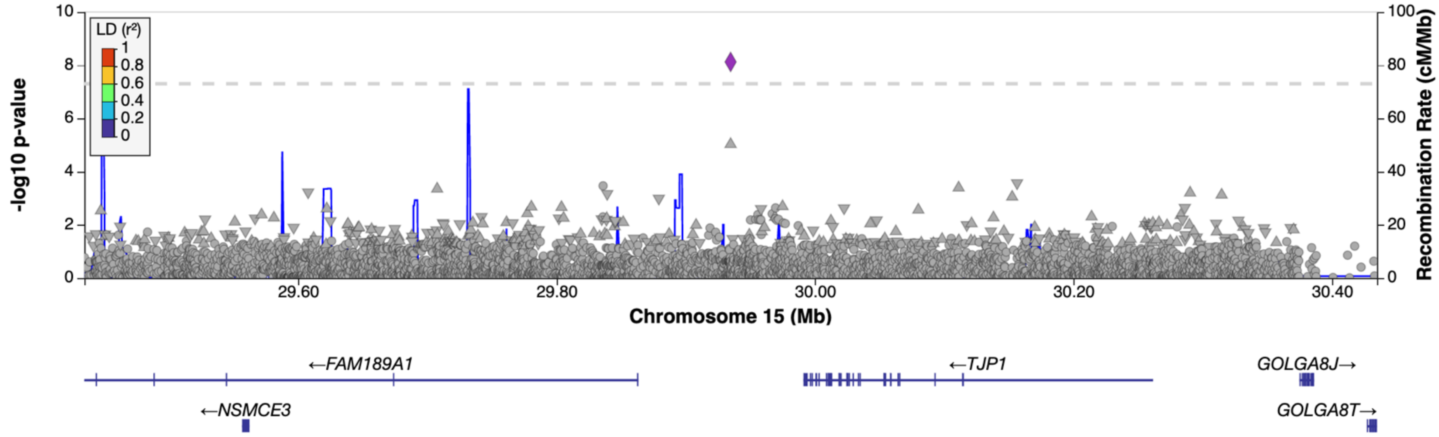

B

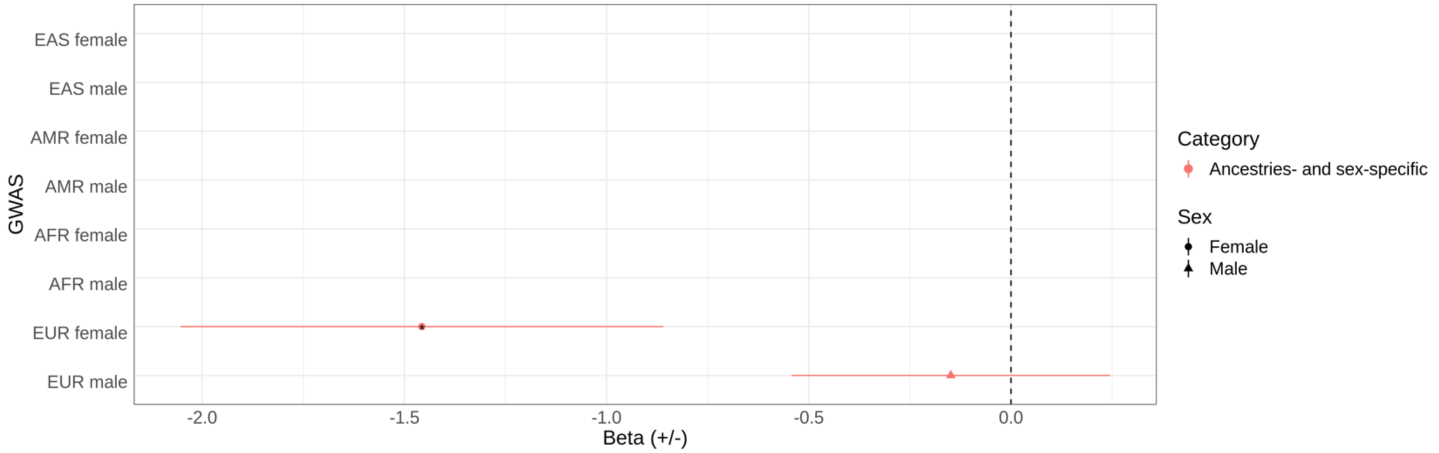

Supplementary Fig 14. Locus zoom and variant effect forest plot of 15:29934686. A) Locus zoom plot of EUR female stuttering associations, where the sentinel variant is denoted in purple and surrounding variants are color coded by r2 bin using linkage disequilibrium (LD) generated from 1000 Genomes EUR reference. The x axis represents chromosome position (hg37) with annotated genes found within the region, the y axis represents $\log_{10}$ (*p*-value) of the association between the genetic variant and stuttering. Sentinel variant is between *FAM189A1* and *TJP1*. B) Variant effect forest plots of 15:29934686 found within the genetic ancestries of European male (EUR male), and European female (EUR female). Variant not found in African male (AFR male), African female (AFR female), Latino/Admixed American male (AMR male), Latino/Admixed American female (AMR female), East Asian male (EAS male), and East Asian female (EAS female). Male variant effects are designated by triangles, and female variant effects are designed by circles. Line length indicates standard error for the betas found in the respective GWAS. Variants reaching replicative significance, *p*-value < 8.77×10^{-4} (.05/57 unique loci) are indicated by asterisks.

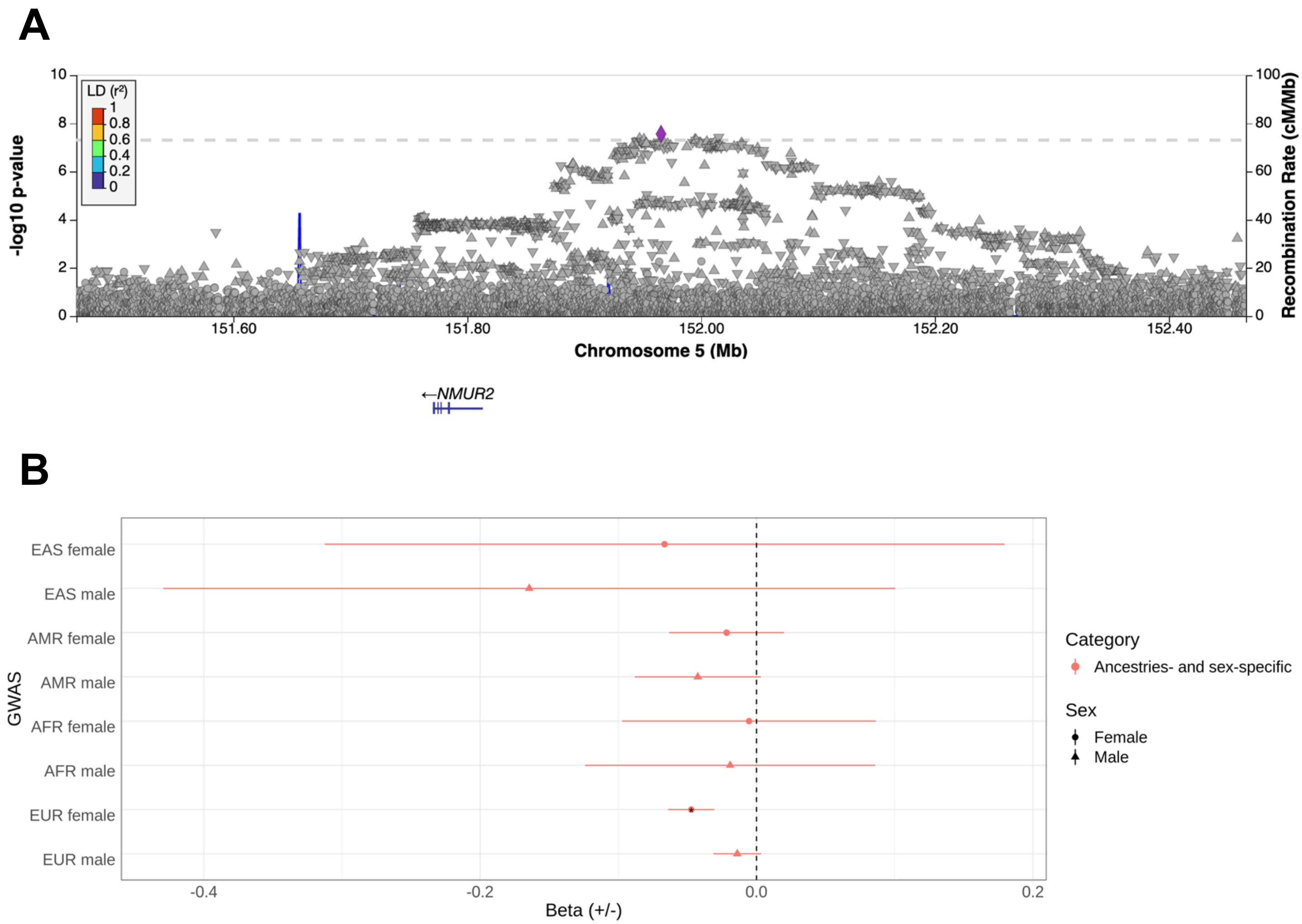

Supplementary Fig 15. Locus zoom and variant effect forest plot of rs535503154. A) Locus zoom plot of EUR female stuttering associations, where the sentinel variant is denoted in purple and surrounding variants are color coded by r2 bin using linkage disequilibrium (LD) generated from 1000 Genomes EUR reference. The x axis represents chromosome position (hg37) with annotated genes found within the region, the y axis represents $\log_{10}$ (*p*-value) of the association between the genetic variant and stuttering. Sentinel variant is upstream of *NMUR2*. B) Variant effect forest plots of rs535503154 found within the genetic ancestries of European male (EUR male), European female (EUR female), African male (AFR male), African female (AFR female), Latino/Admixed American male (AMR male), Latino/Admixed American female (AMR female), East Asian male (EAS male), and East Asian female (EAS female). Male variant effects are designated by triangles, and female variant effects are designed by circles. Line length indicates standard error for the betas found in the respective GWAS. Variants reaching replicative significance, *p*-value < 8.77×10^{-4} (.05/57 unique loci) are indicated by asterisks.

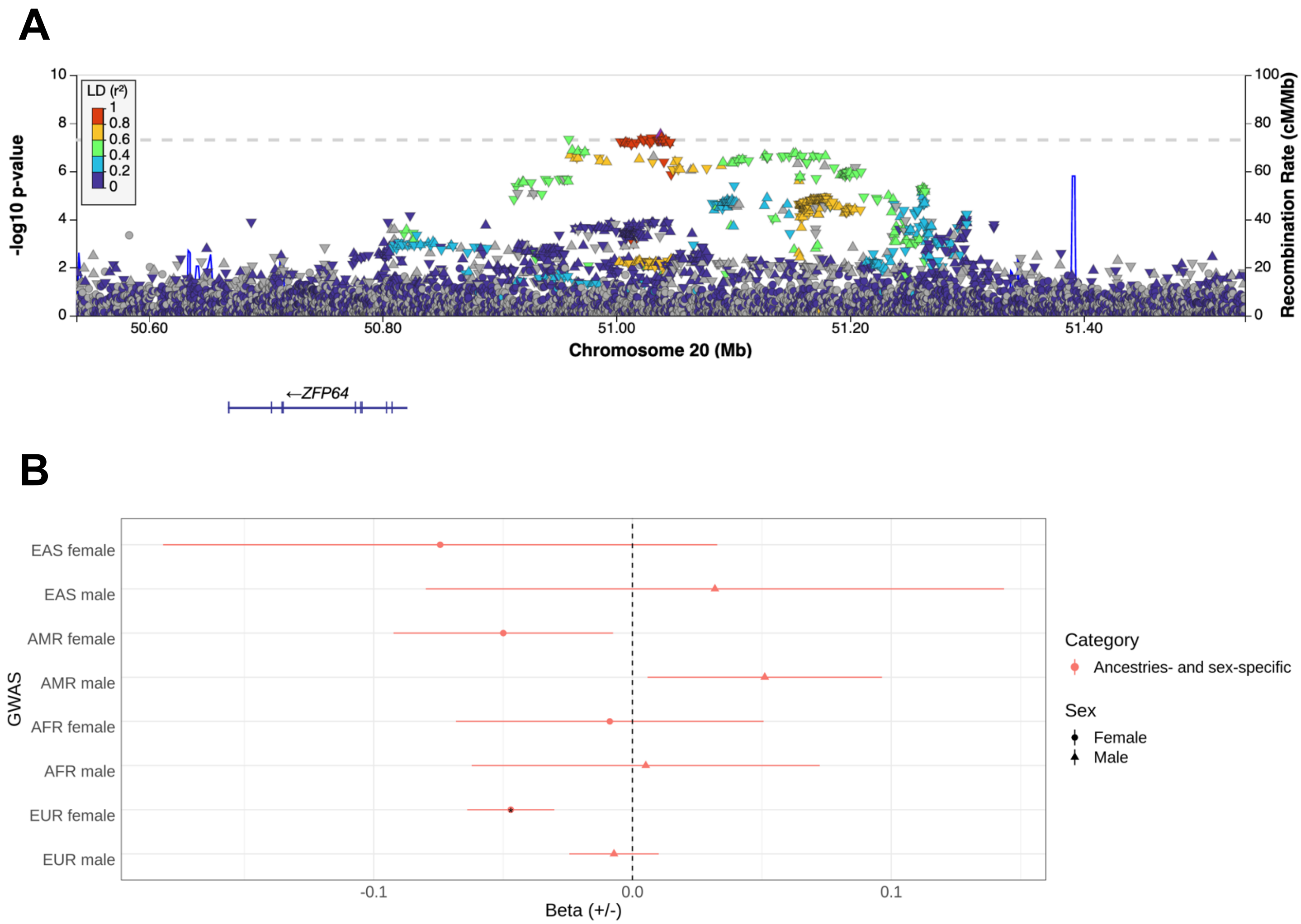

Supplementary Fig 16. Locus zoom and variant effect forest plot of rs968163. A) Locus zoom plot of EUR female stuttering associations, where the sentinel variant is denoted in purple and surrounding variants are color coded by r2 bin using linkage disequilibrium (LD) generated from 1000 Genomes EUR reference. The x axis represents chromosome position (hg37) with annotated genes found within the region, the y axis represents $\log_{10}$ (*p*-value) of the association between the genetic variant and stuttering. Sentinel variant is upstream of *ZFP64*. B) Variant effect forest plots of rs968163 found within the genetic ancestries of European male (EUR male), European female (EUR female), African male (AFR male), African female (AFR female), Latino/Admixed American male (AMR male), Latino/Admixed American female (AMR female), East Asian male (EAS male), and East Asian female (EAS female). Male variant effects are designated by triangles, and female variant effects are designed by circles. Line length indicates standard error for the betas found in the respective GWAS. Variants reaching replicative significance, *p*-value < 8.77×10^{-4} (.05/57 unique loci) are indicated by asterisks.

A

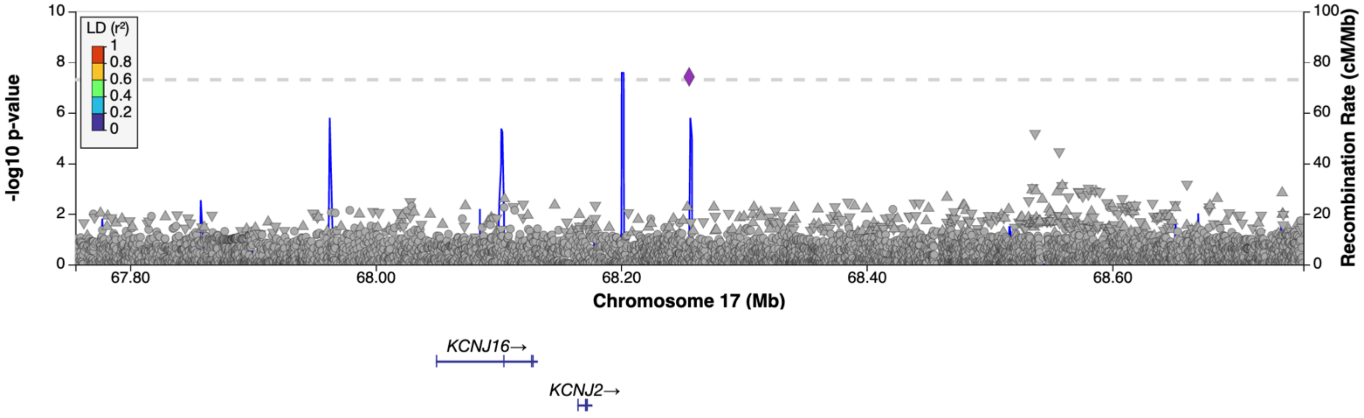

B

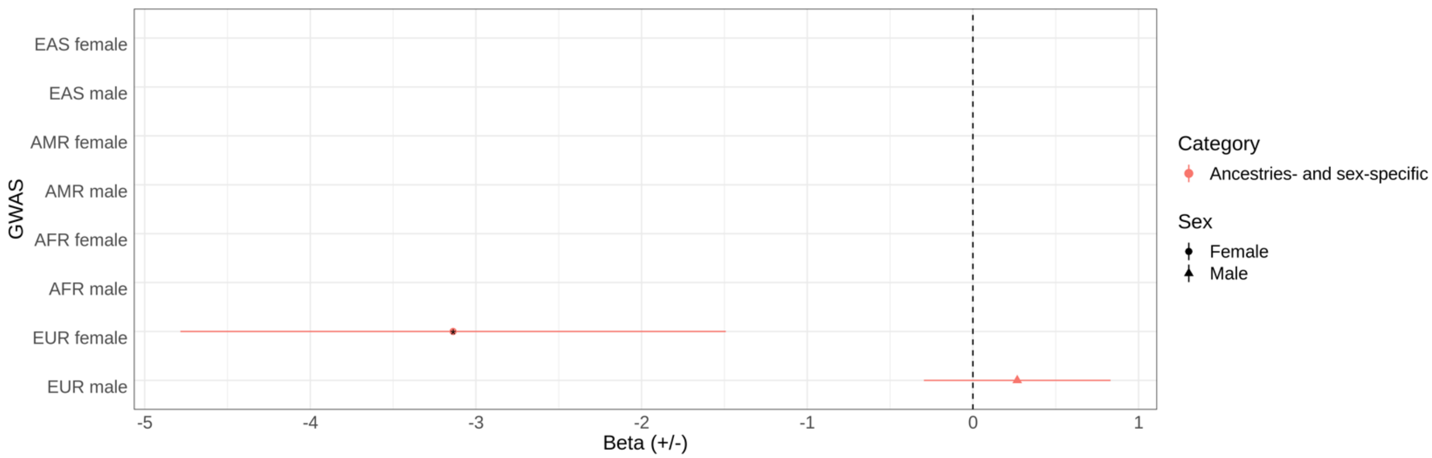

Supplementary Fig 17. Locus zoom and variant effect forest plot of rs529593131. A) Locus zoom plot of EUR female stuttering associations, where the sentinel variant is denoted in purple and surrounding variants are color coded by r2 bin using linkage disequilibrium (LD) generated from 1000 Genomes EUR reference. The x axis represents chromosome position (hg37) with annotated genes found within the region, the y axis represents $\log_{10}$ (*p*-value) of the association between the genetic variant and stuttering. Sentinel variant is downstream of *KCNJ2*. B) Variant effect forest plots of rs529593131 found within the genetic ancestries of European male (EUR male), and European female (EUR female). Variant not found in African male (AFR male), African female (AFR female), Latino/Admixed American male (AMR male), Latino/Admixed American male (AMR female), East Asian male (EAS male), and East Asian female (EAS female). Male variant effects are designated by triangles, and female variant effects are designed by circles. Line length indicates standard error for the betas found in the respective GWAS. Variants reaching replicative significance, *p*-value < 8.77×10^{-4} (.05/57 unique loci) are indicated by asterisks.

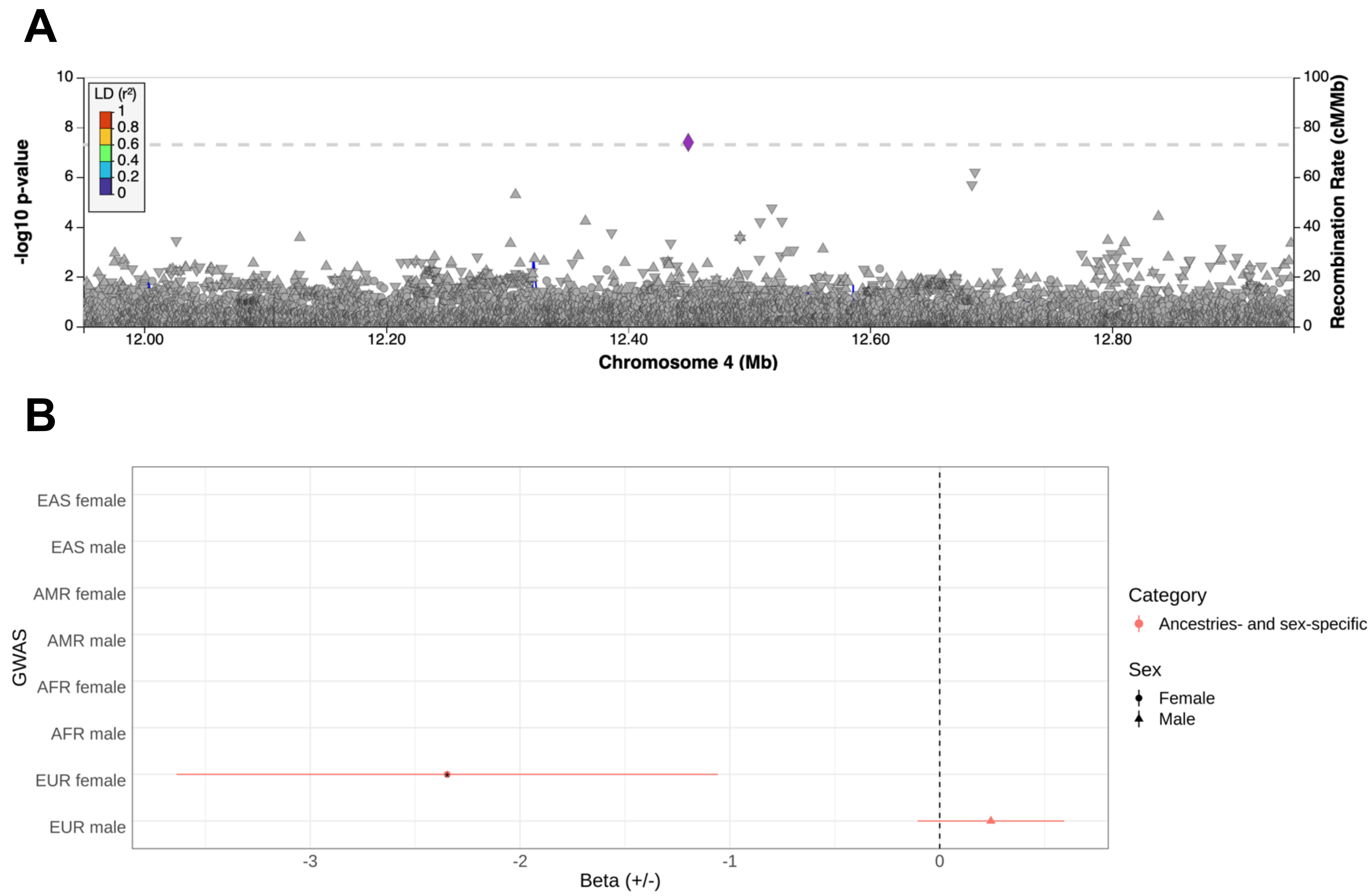

Supplementary Fig 18. Locus zoom and variant effect forest plot of rs779897701. A) Locus zoom plot of EUR female stuttering associations, where the sentinel variant is denoted in purple and surrounding variants are color coded by r2 bin using linkage disequilibrium (LD) generated from 1000 Genomes EUR reference. The x axis represents chromosome position (hg37) with annotated genes found within the region, the y axis represents $\log_{10}$ (*p*-value) of the association between the genetic variant and stuttering. Sentinel variant is located more than 500kb (upstream or downstream) from a protein-coding gene. B) Variant effect forest plots of rs779897701 found within the genetic ancestries of European male (EUR male), and European female (EUR female). Variant not found in African male (AFR male), African female (AFR female), Latino/Admixed American male (AMR male), Latino/Admixed American female (AMR female), East Asian male (EAS male), and East Asian female (EAS female). Male variant effects are designated by triangles, and female variant effects are designed by circles. Line length indicates standard error for the betas found in the respective GWAS. Variants reaching replicative significance, *p*-value < 8.77×10^{-4} (.05/57 unique loci) are indicated by asterisks.

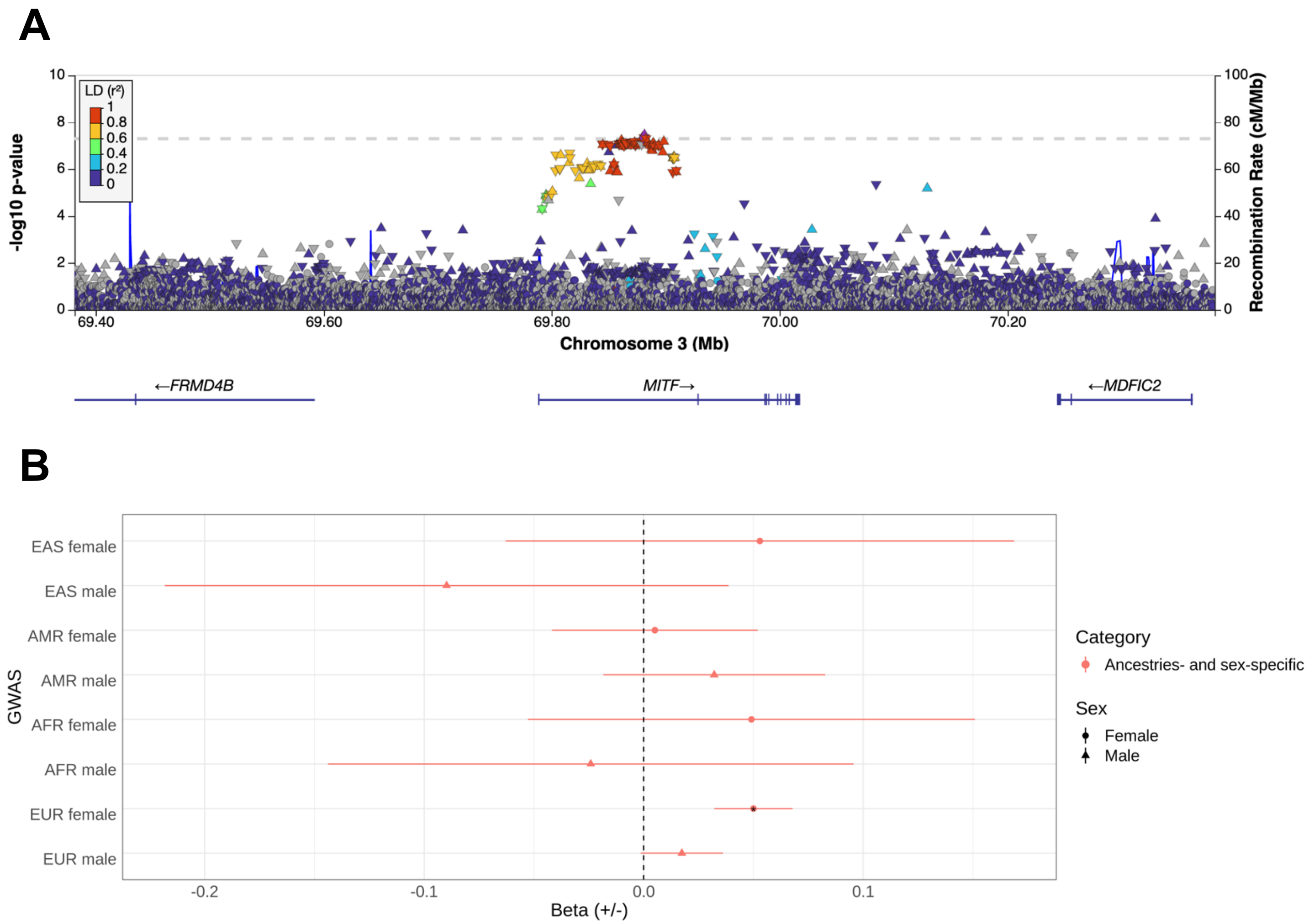

Supplementary Fig 19. Locus zoom and variant effect forest plot of rs62252182. A) Locus zoom plot of EUR female stuttering associations, where the sentinel variant is denoted in purple and surrounding variants are color coded by r2 bin using linkage disequilibrium (LD) generated from 1000 Genomes EUR reference. The x axis represents chromosome position (hg37) with annotated genes found within the region, the y axis represents $\log_{10}$ (*p*-value) of the association between the genetic variant and stuttering. Sentinel variant is an intronic or genic upstream transcript variant within *MITF*. B) Variant effect forest plots of rs62252182 found within the genetic ancestries of European male (EUR male), European female (EUR female), African male (AFR male), African female (AFR female), Latino/Admixed American male (AMR male), Latino/Admixed American female (AMR female), East Asian male (EAS male), and East Asian female (EAS female). Male variant effects are designated by triangles, and female variant effects are designed by circles. Line length indicates standard error for the betas found in the respective GWAS. Variants reaching replicative significance, *p*-value < 8.77×10^{-4} (.05/57 unique loci) are indicated by asterisks.

A

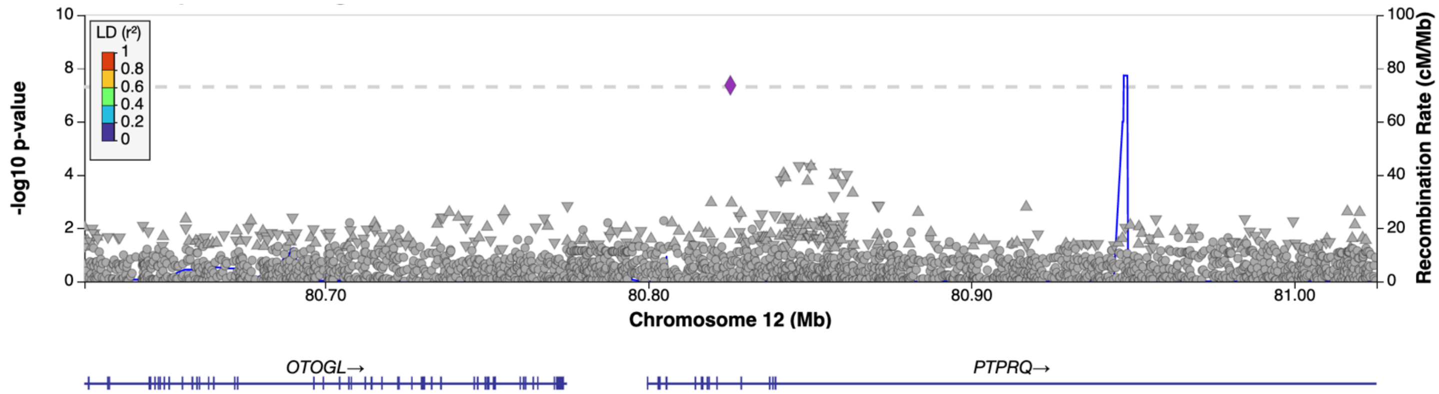

B

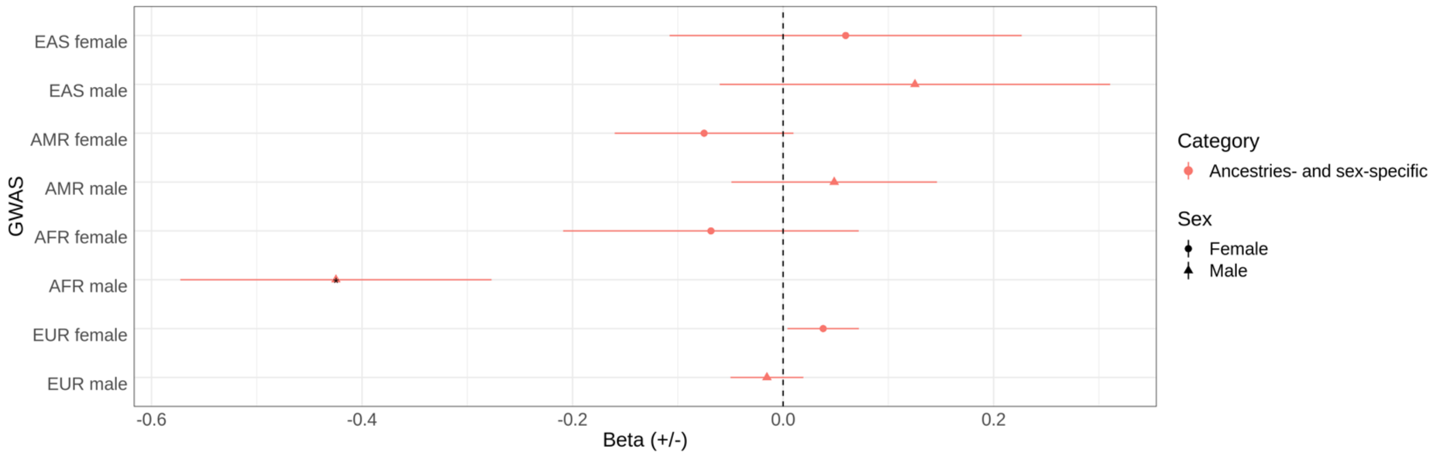

Supplementary Fig 20. Locus zoom and variant effect forest plot of rs541395135. A) Locus zoom plot of AFR male stuttering associations, where the sentinel variant is denoted in purple and surrounding variants are color coded by r2 bin using linkage disequilibrium (LD) generated from 1000 Genomes AFR reference. The x axis represents chromosome position (hg37) with annotated genes found within the region, the y axis represents $\log_{10}$ (*p*-value) of the association between the genetic variant and stuttering. Sentinel variant is an intronic variant within *PTPRQ*. B) Variant effect forest plots of rs541395135 found within the genetic ancestries of European male (EUR male), European female (EUR female), African male (AFR male), African female (AFR female), Latino/Admixed American male (AMR male), Latino/Admixed American female (AMR female), East Asian male (EAS male), and East Asian female (EAS female). Male variant effects are designated by triangles, and female variant effects are designed by circles. Line length indicates standard error for the betas found in the respective GWAS. Variants reaching replicative significance, *p*-value < 8.77×10^{-4} (.05/57 unique loci) are indicated by asterisks.

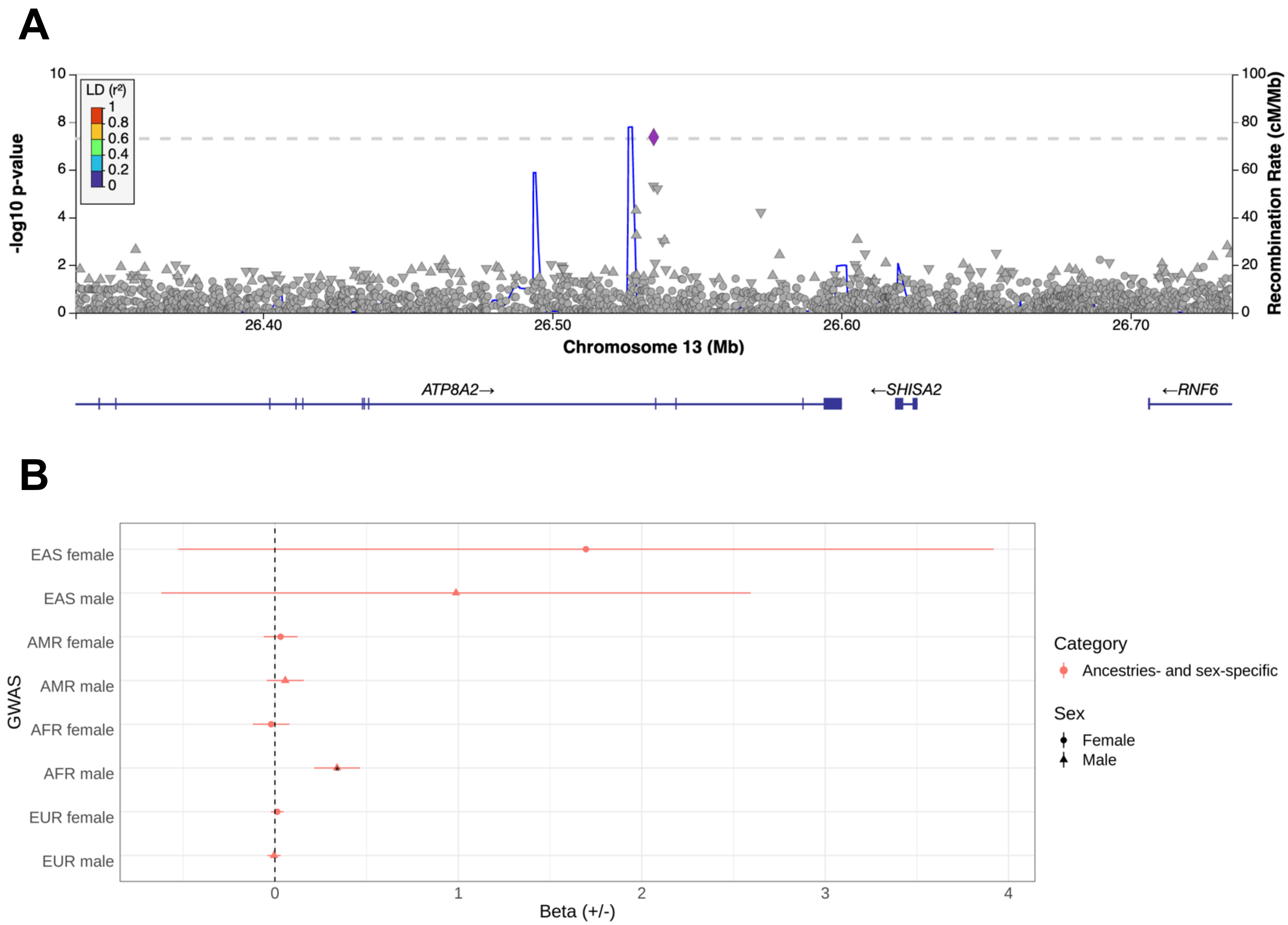

Supplementary Fig 21. Locus zoom and variant effect forest plot of rs7333000. A) Locus zoom plot of AFR male stuttering associations, where the sentinel variant is denoted in purple and surrounding variants are color coded by r2 bin using linkage disequilibrium (LD) generated from 1000 Genomes AFR reference. The x axis represents chromosome position (hg37) with annotated genes found within the region, the y axis represents $\log_{10}$ (*p*-value) of the association between the genetic variant and stuttering. Sentinel variant is an intronic variant within *ATP8A2*. B) Variant effect forest plots of rs7333000 found within the genetic ancestries of European male (EUR male), European female (EUR female), African male (AFR male), African female (AFR female), Latino/Admixed American male (AMR male), Latino/Admixed American female (AMR female), East Asian male (EAS male), and East Asian female (EAS female). Male variant effects are designated by triangles, and female variant effects are designed by circles. Line length indicates standard error for the betas found in the respective GWAS. Variants reaching replicative significance, *p*-value < 8.77 x 10^{-4} (.05/57 unique loci) are indicated by asterisks.

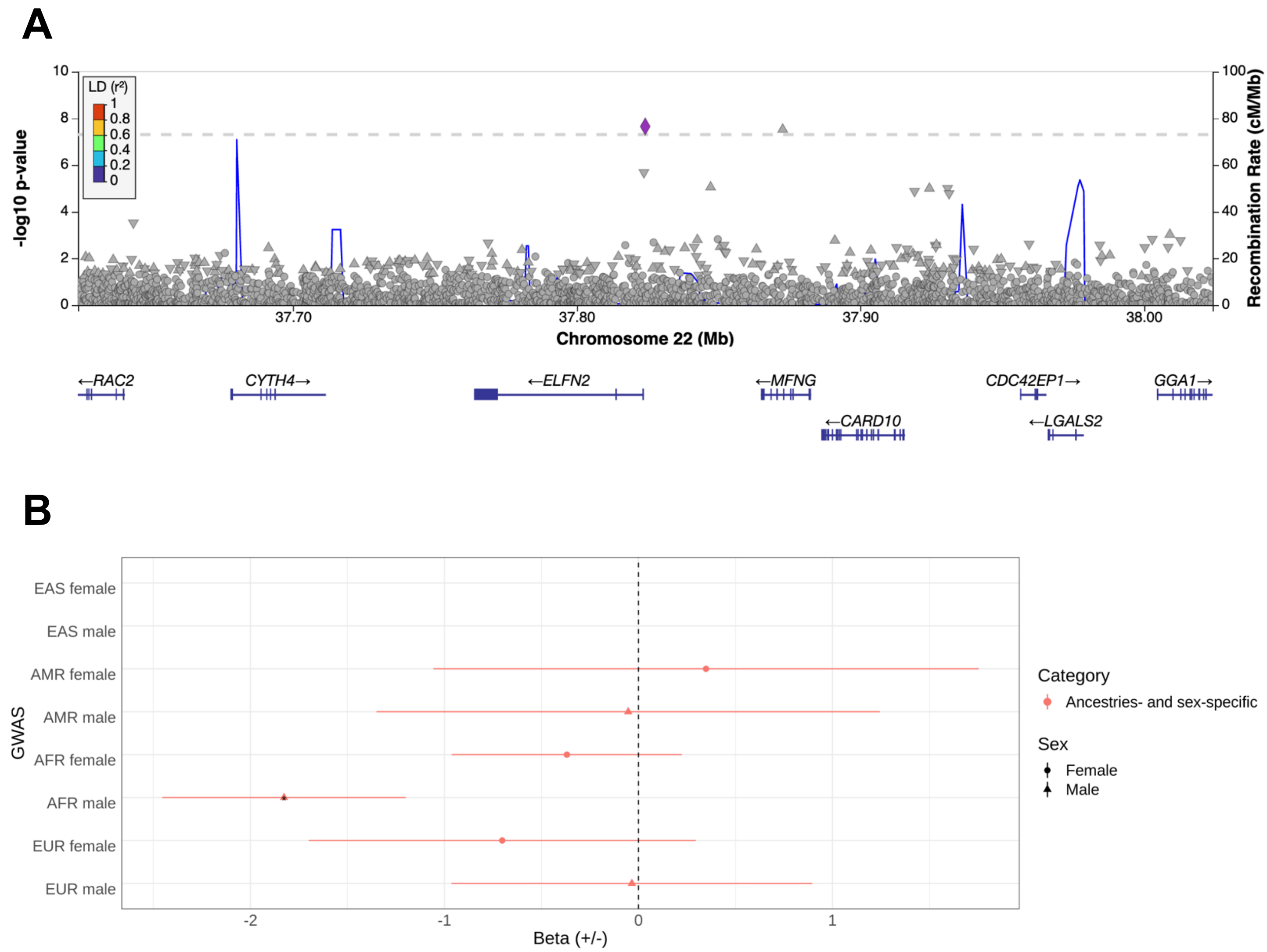

Supplementary Fig 22. Locus zoom and variant effect forest plot of rs192857772. A) Locus zoom plot of AFR male stuttering associations, where the sentinel variant is denoted in purple and surrounding variants are color coded by r2 bin using linkage disequilibrium (LD) generated from 1000 Genomes AFR reference. The x axis represents chromosome position (hg37) with annotated genes found within the region, the y axis represents $\log_{10}$ (*p*-value) of the association between the genetic variant and stuttering. Sentinel variant is an upstream transcript variant for *ELFN2*. B) Variant effect forest plots of rs192857772 found within the genetic ancestries of European male (EUR male), European female (EUR female), African male (AFR male), African female (AFR female), Latino/Admixed American male (AMR male), and Latino/Admixed American female (AMR female). Variant not found in East Asian male (EAS male), and East Asian female (EAS female). Male variant effects are designated by triangles, and female variant effects are designed by circles. Line length indicates standard error for the betas found in the respective GWAS. Variants reaching replicative significance, *p*-value < 8.77×10^{-4} (.05/57 unique loci) are indicated by asterisks.

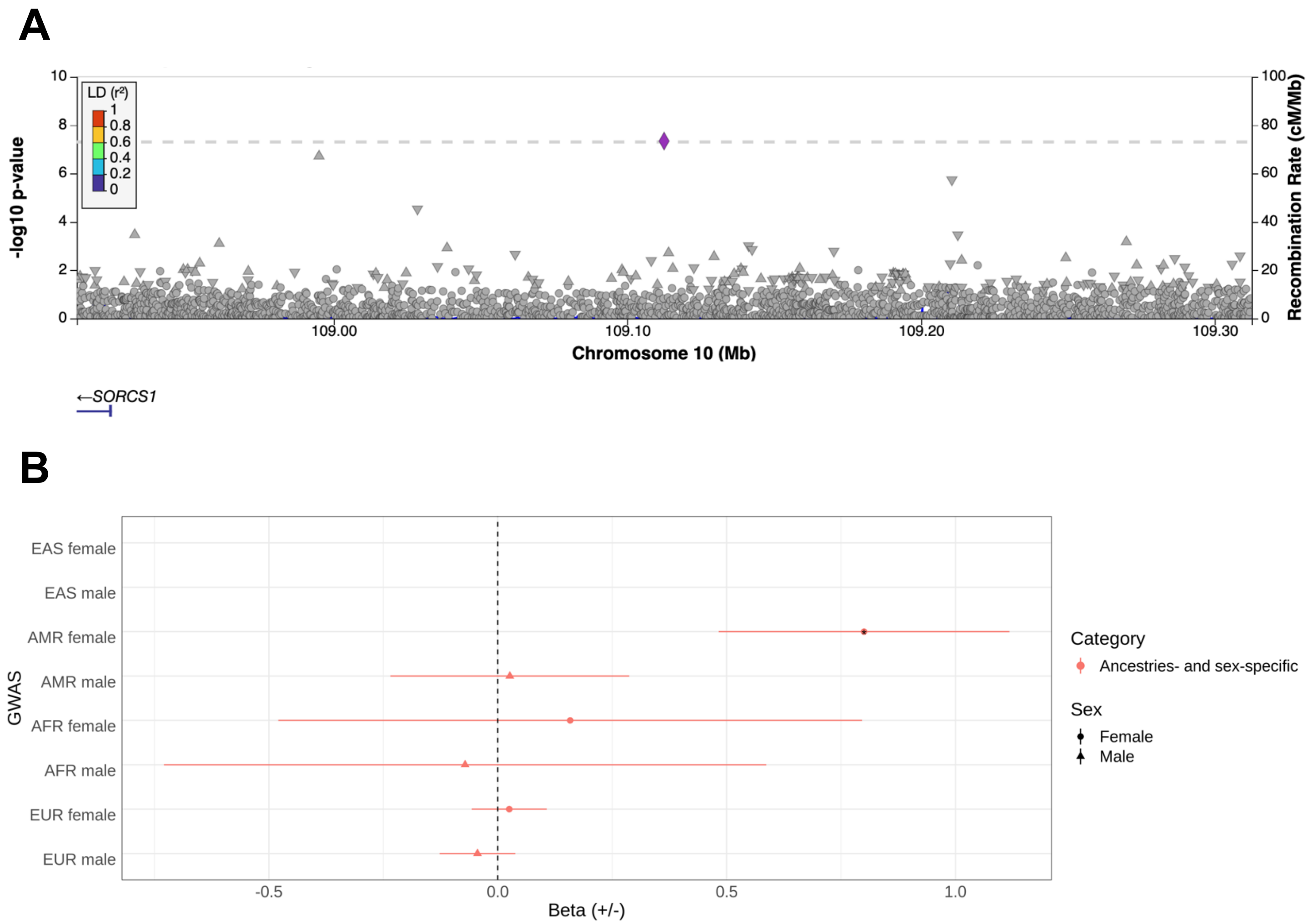

Supplementary Fig 23. Locus zoom and variant effect forest plot of rs35713684. A) Locus zoom plot of AMR female stuttering associations, where the sentinel variant is denoted in purple and surrounding variants are color coded by r2 bin using linkage disequilibrium (LD) generated from 1000 Genomes AMR reference. The x axis represents chromosome position (hg37) with annotated genes found within the region, the y axis represents $\log_{10}$ (*p*-value) of the association between the genetic variant and stuttering. Sentinel variant is upstream of *SORCS1*. B) Variant effect forest plots of rs35713684 found within the genetic ancestries of European male (EUR male), European female (EUR female), African male (AFR male), African female (AFR female), Latino/Admixed American male (AMR male), and Latino/Admixed American female (AMR female). Variant not found in East Asian male (EAS male), and East Asian female (EAS female). Male variant effects are designated by triangles, and female variant effects are designed by circles. Line length indicates standard error for the betas found in the respective GWAS. Variants reaching replicative significance, *p*-value < 8.77×10^{-4} (.05/57 unique loci) are indicated by asterisks.

A

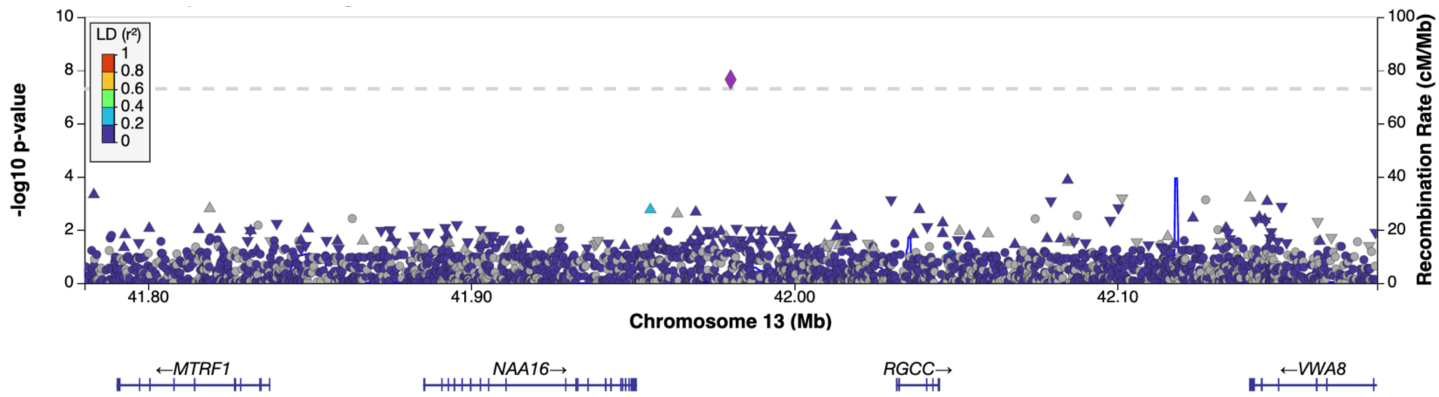

B

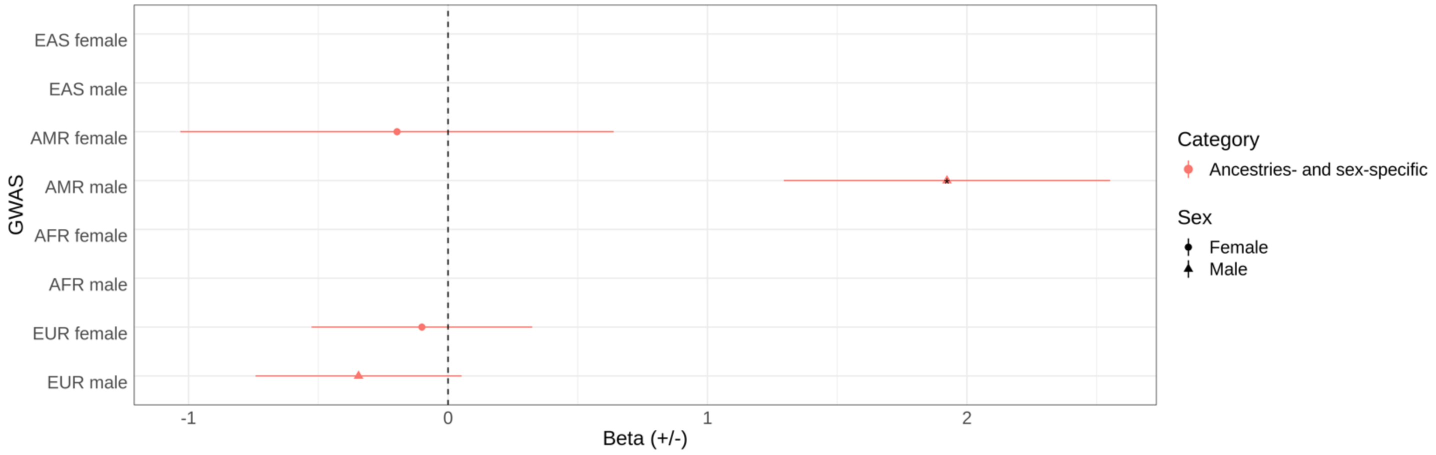

Supplementary Fig 24. Locus zoom and variant effect forest plot of rs556601931. A) Locus zoom plot of AMR male stuttering associations, where the sentinel variant is denoted in purple and surrounding variants are color coded by r2 bin using linkage disequilibrium (LD) generated from 1000 Genomes AMR reference. The x axis represents chromosome position (hg37) with annotated genes found within the region, the y axis represents $\log_{10}$ (*p*-value) of the association between the genetic variant and stuttering. Sentinel variant is between *NAA16* and *RGCC*. B) Variant effect forest plots of rs556601931 found within the genetic ancestries of European male (EUR male), European female (EUR female), Latino/Admixed American male (AMR male), and Latino/Admixed American female (AMR female). Variant not found in African male (AFR male), African female (AFR female), East Asian male (EAS male), and East Asian female (EAS female). Male variant effects are designated by triangles, and female variant effects are designed by circles. Line length indicates standard error for the betas found in the respective GWAS. Variants reaching replicative significance, *p*-value < 8.77×10^{-4} (.05/57 unique loci) are indicated by asterisks.

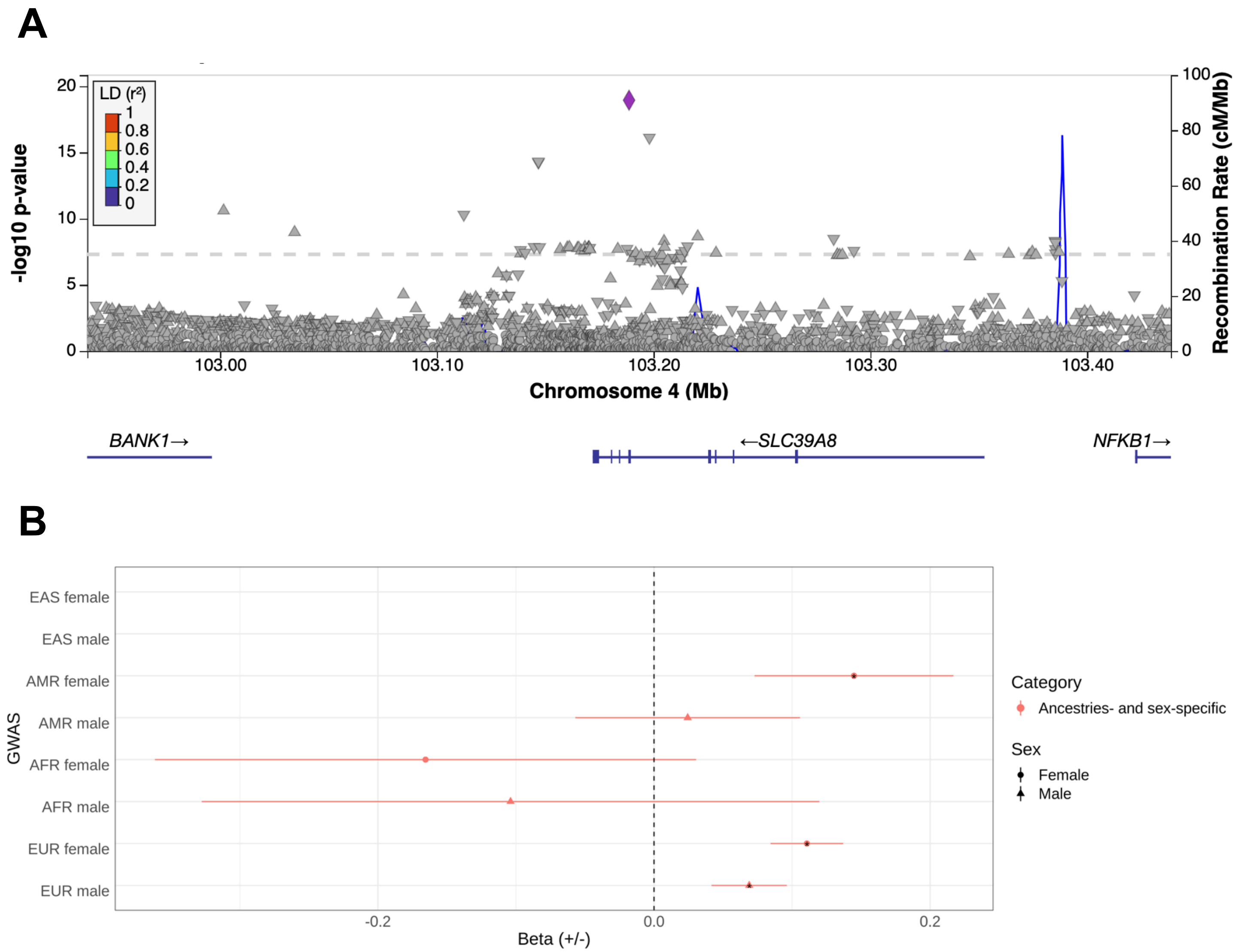

Supplementary Fig 25. Locus zoom and variant effect forest plot of rs13107325. A) Locus zoom plot of sex-combined EUR stuttering associations, where the sentinel variant is denoted in purple and surrounding variants are color coded by r2 bin using linkage disequilibrium (LD) generated from 1000 Genomes ALL reference. The x axis represents chromosome position (hg37) with annotated genes found within the region, the y axis represents $\log_{10}$ (*p*-value) of the association between the genetic variant and stuttering. Sentinel variant is a missense variant within *SLC39A8*. B) Variant effect forest plots of rs13107325 found within the genetic ancestries of European male (EUR male), European female (EUR female), African male (AFR male), African female (AFR female), Latino/Admixed American male (AMR male), and Latino/Admixed American female (AMR female). Variant not found in East Asian male (EAS male), and East Asian female (EAS female). Male variant effects are designated by triangles, and female variant effects are designed by circles. Line length indicates standard error for the betas found in the respective GWAS. Variants reaching replicative significance, *p*-value < 8.77×10^{-4} (.05/57 unique loci) are indicated by asterisks.

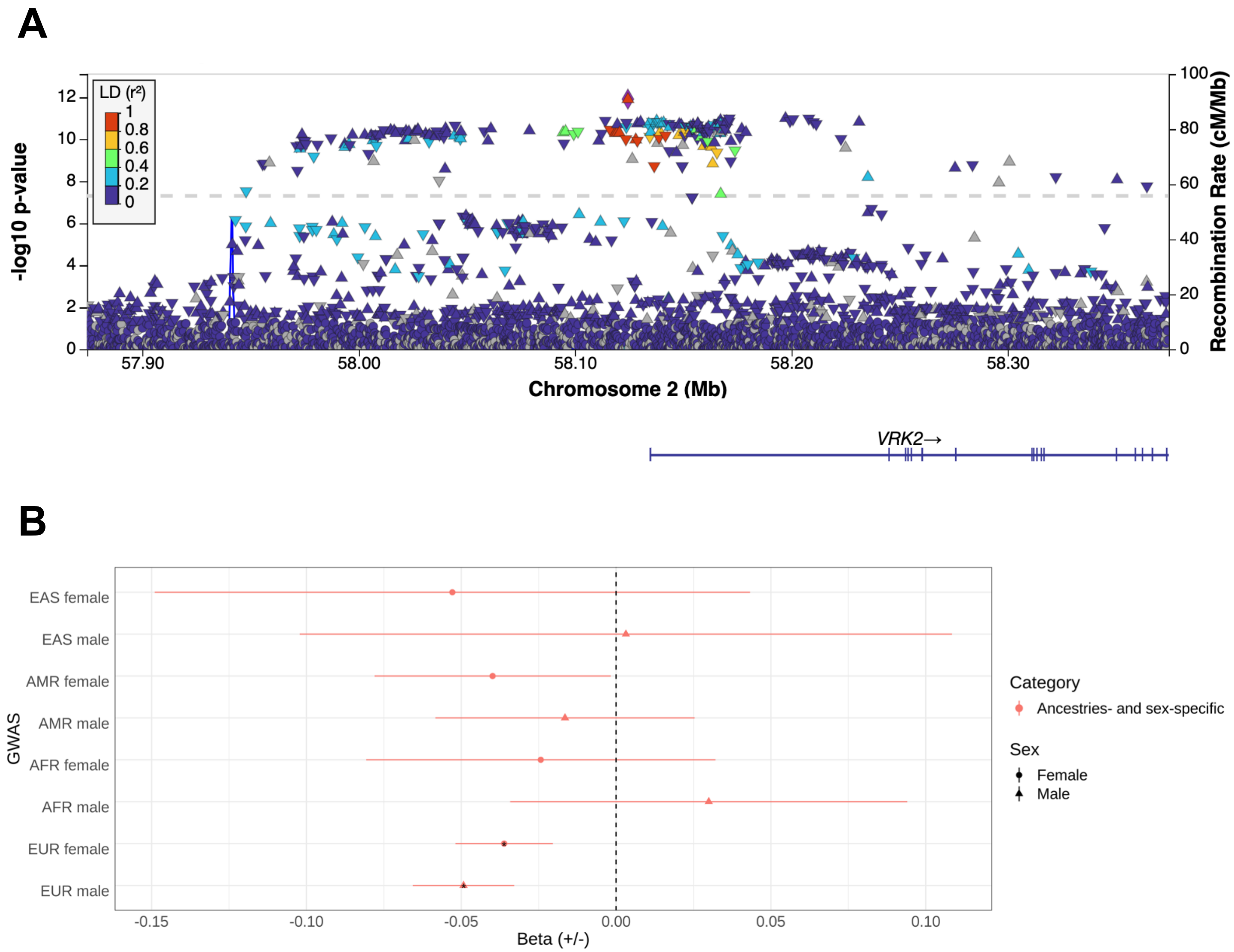

Supplementary Fig 26. Locus zoom and variant effect forest plot of rs72808425. A) Locus zoom plot of sex-combined EUR stuttering associations, where the sentinel variant is denoted in purple and surrounding variants are color coded by r2 bin using linkage disequilibrium (LD) generated from 1000 Genomes ALL reference. The x axis represents chromosome position (hg37) with annotated genes found within the region, the y axis represents $\log_{10}$ (*p*-value) of the association between the genetic variant and stuttering. Sentinel variant is upstream of *VRK2*. B) Variant effect forest plots of rs72808425 found within the genetic ancestries of European male (EUR male), European female (EUR female), African male (AFR male), African female (AFR female), Latino/Admixed American male (AMR male), Latino/Admixed American female (AMR female), East Asian male (EAS male), and East Asian female (EAS female). Male variant effects are designated by triangles, and female variant effects are designed by circles. Line length indicates standard error for the betas found in the respective GWAS. Variants reaching replicative significance, *p*-value < 8.77×10^{-4} (.05/57 unique loci) are indicated by asterisks.

A

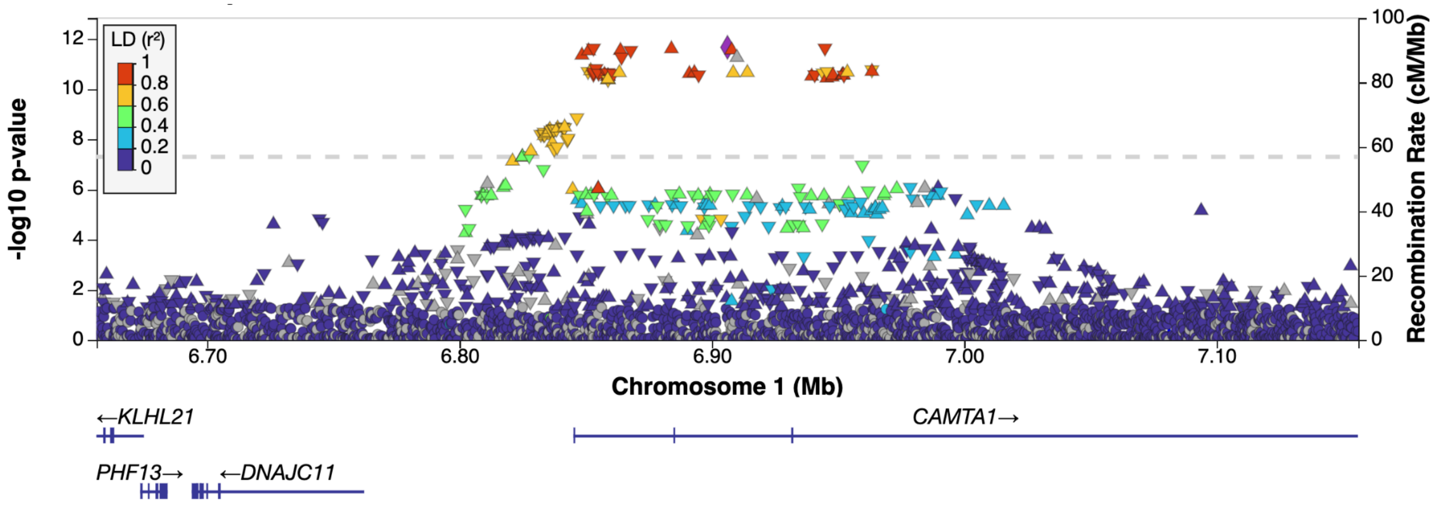

B

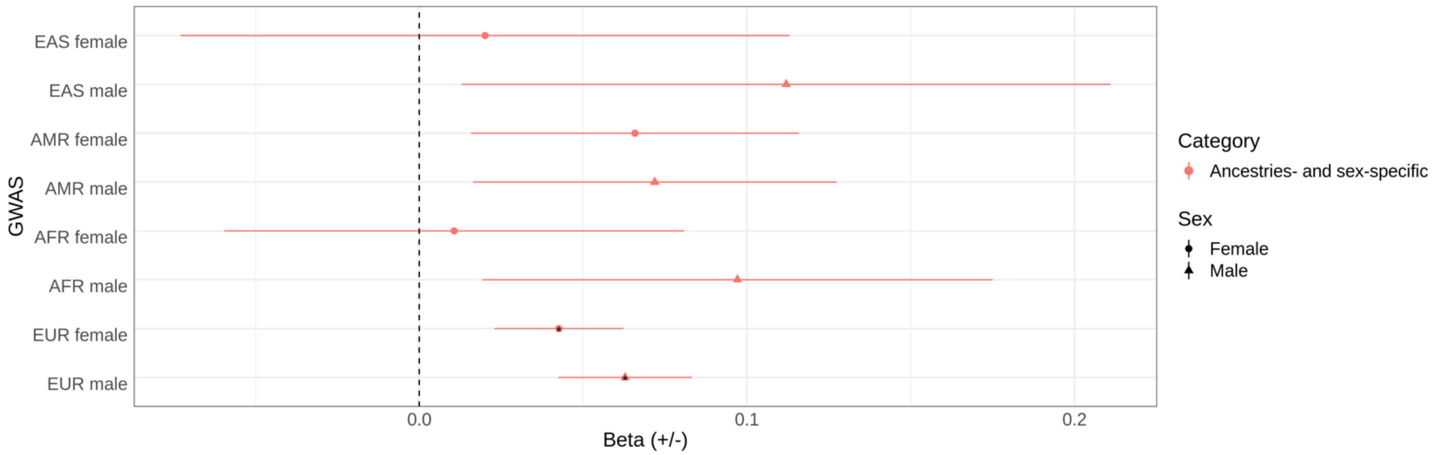

Supplementary Fig 27. Locus zoom and variant effect forest plot of rs12035477. A) Locus zoom plot of sex-combined EUR stuttering associations, where the sentinel variant is denoted in purple and surrounding variants are color coded by r2 bin using linkage disequilibrium (LD) generated from 1000 Genomes ALL reference. The x axis represents chromosome position (hg37) with annotated genes found within the region, the y axis represents $\log_{10}$ (*p*-value) of the association between the genetic variant and stuttering. Sentinel variant is an intronic variant within *CAMTA1*. B) Variant effect forest plots of rs12035477 found within the genetic ancestries of European male (EUR male), European female (EUR female), African male (AFR male), African female (AFR female), Latino/Admixed American male (AMR male), Latino/Admixed American female (AMR female), East Asian male (EAS male), and East Asian female (EAS female). Male variant effects are designated by triangles, and female variant effects are designed by circles. Line length indicates standard error for the betas found in the respective GWAS. Variants reaching replicative significance, *p*-value < 8.77×10^{-4} (.05/57 unique loci) are indicated by asterisks.

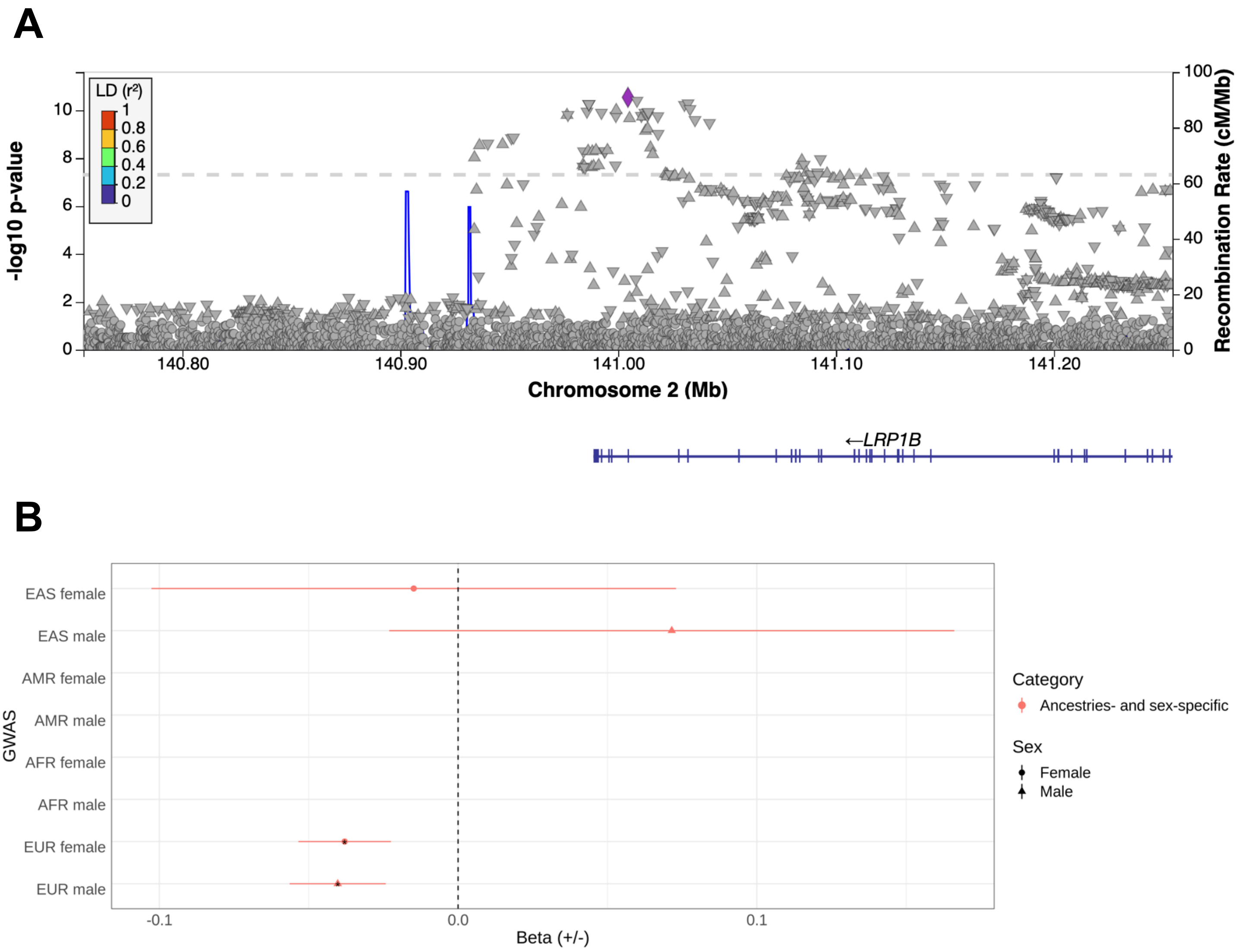

Supplementary Fig 28. Locus zoom and variant effect forest plot of rs11895946. A) Locus zoom plot of sex-combined EUR stuttering associations, where the sentinel variant is denoted in purple and surrounding variants are color coded by r2 bin using linkage disequilibrium (LD) generated from 1000 Genomes ALL reference. The x axis represents chromosome position (hg37) with annotated genes found within the region, the y axis represents $\log_{10}$ (*p*-value) of the association between the genetic variant and stuttering. Sentinel variant is an intronic variant within *LRP1B*. B) Variant effect forest plots of rs11895946 found within the genetic ancestries of European male (EUR male), European female (EUR female), Latino/Admixed American male (AMR male), and Latino/Admixed American female (AMR female). Variant not found in African male (AFR male), African female (AFR female), East Asian male (EAS male), and East Asian female (EAS female). Male variant effects are designated by triangles, and female variant effects are designed by circles. Line length indicates standard error for the betas found in the respective GWAS. Variants reaching replicative significance, *p*-value < 8.77×10^{-4} (.05/57 unique loci) are indicated by asterisks.

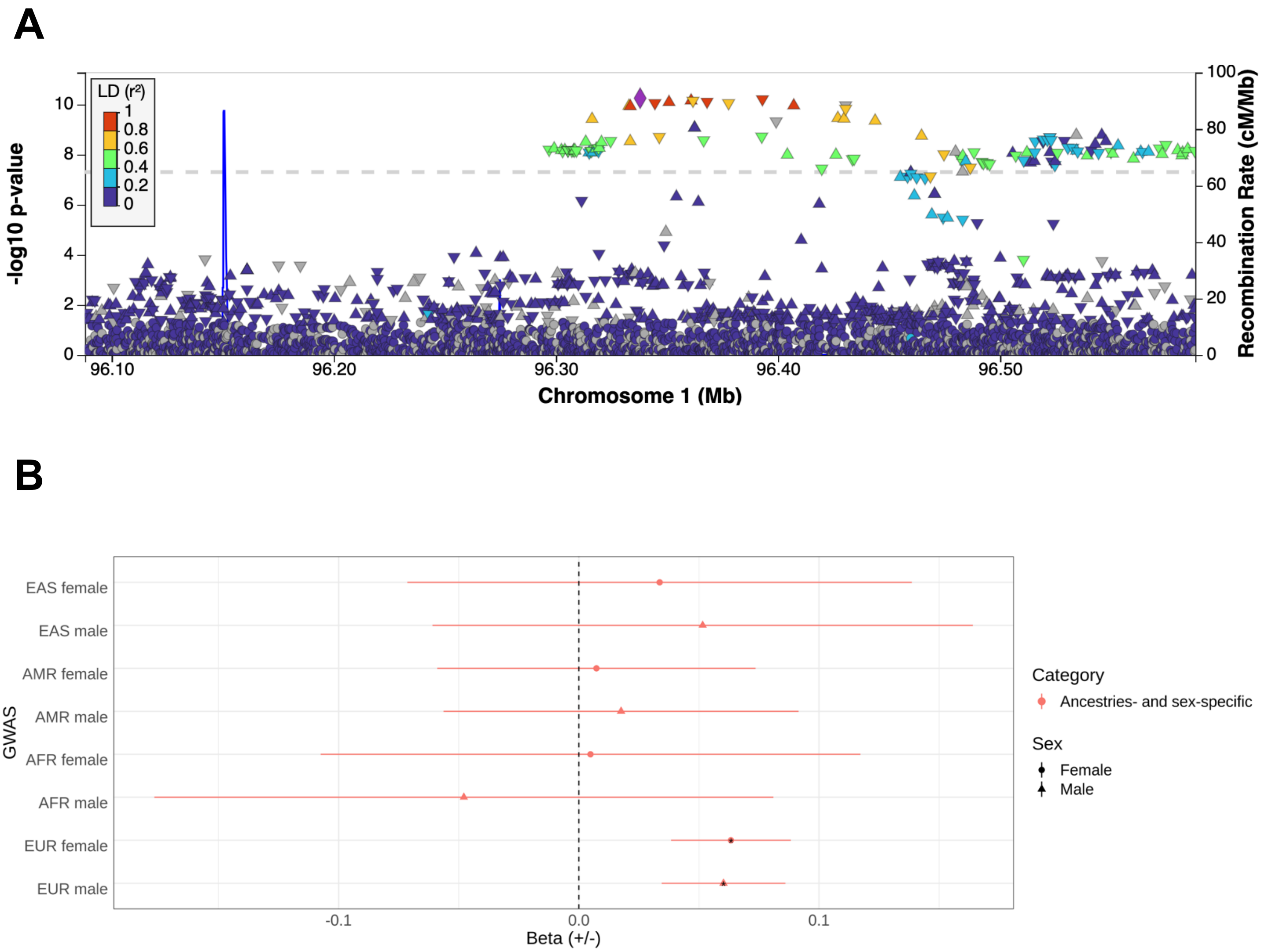

Supplementary Fig 29. Locus zoom and variant effect forest plot of rs12040559. A) Locus zoom plot of sex-combined EUR stuttering associations, where the sentinel variant is denoted in purple and surrounding variants are color coded by r2 bin using linkage disequilibrium (LD) generated from 1000 Genomes ALL reference. The x axis represents chromosome position (hg37) with annotated genes found within the region, the y axis represents $\log_{10}$ (*p*-value) of the association between the genetic variant and stuttering. Sentinel variant is located more than 500kb (upstream or downstream) from a protein-coding gene. B) Variant effect forest plots of rs12040559 found within the genetic ancestries of European male (EUR male), European female (EUR female), African male (AFR male), African female (AFR female), Latino/Admixed American male (AMR male), Latino/Admixed American female (AMR female), East Asian male (EAS male), and East Asian female (EAS female). Male variant effects are designated by triangles, and female variant effects are designed by circles. Line length indicates standard error for the betas found in the respective GWAS. Variants reaching replicative significance, *p*-value < 8.77×10^{-4} (.05/57 unique loci) are indicated by asterisks.

A

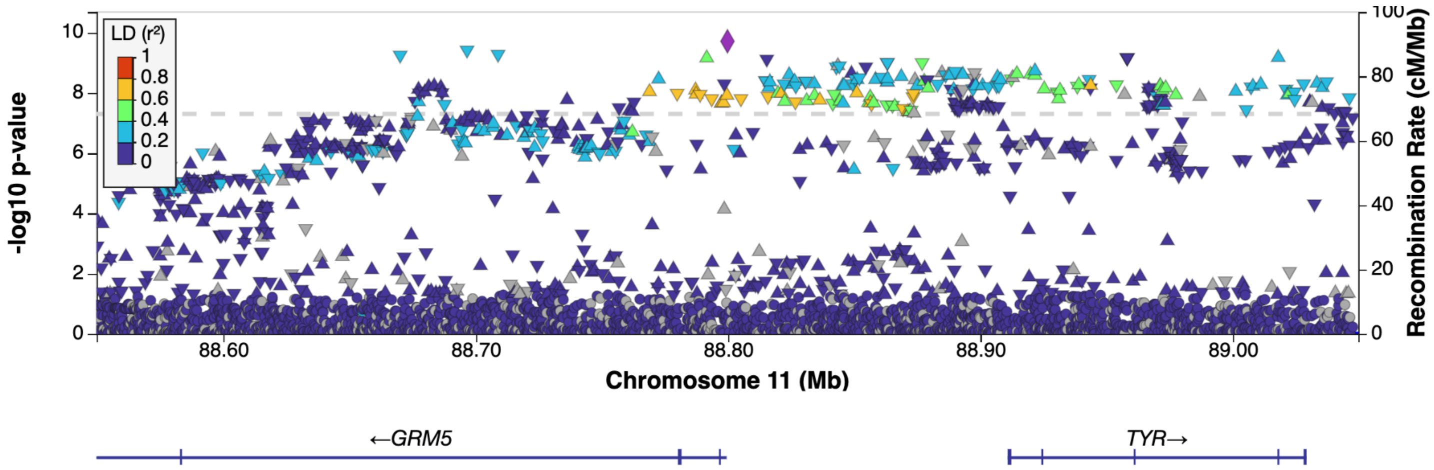

B

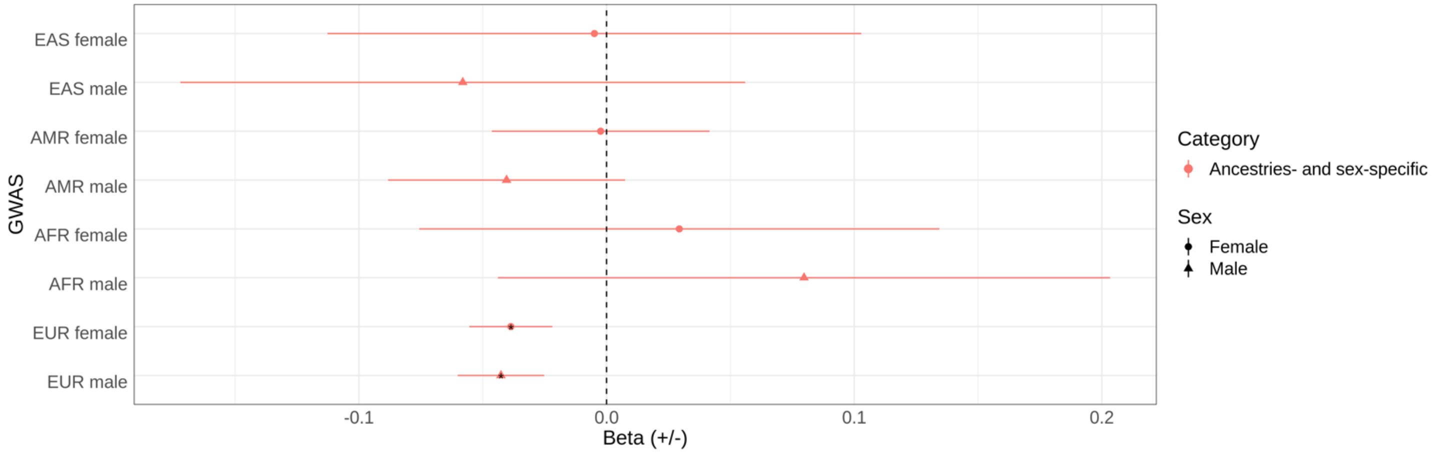

Supplementary Fig 30. Locus zoom and variant effect forest plot of rs34749698. A) Locus zoom plot of sex-combined EUR stuttering associations, where the sentinel variant is denoted in purple and surrounding variants are color coded by r2 bin using linkage disequilibrium (LD) generated from 1000 Genomes ALL reference. The x axis represents chromosome position (hg37) with annotated genes found within the region, the y axis represents $\log_{10}$ (*p*-value) of the association between the genetic variant and stuttering. Sentinel variant is between *GRM5* and *TYR*. B) Variant effect forest plots of rs34749698 found within the genetic ancestries of European male (EUR male), European female (EUR female), African male (AFR male), African female (AFR female), Latino/Admixed American male (AMR male), Latino/Admixed American female (AMR female), East Asian male (EAS male), and East Asian female (EAS female). Male variant effects are designated by triangles, and female variant effects are designed by circles. Line length indicates standard error for the betas found in the respective GWAS. Variants reaching replicative significance, *p*-value < 8.77 x 10^{-4} (.05/57 unique loci) are indicated by asterisks.

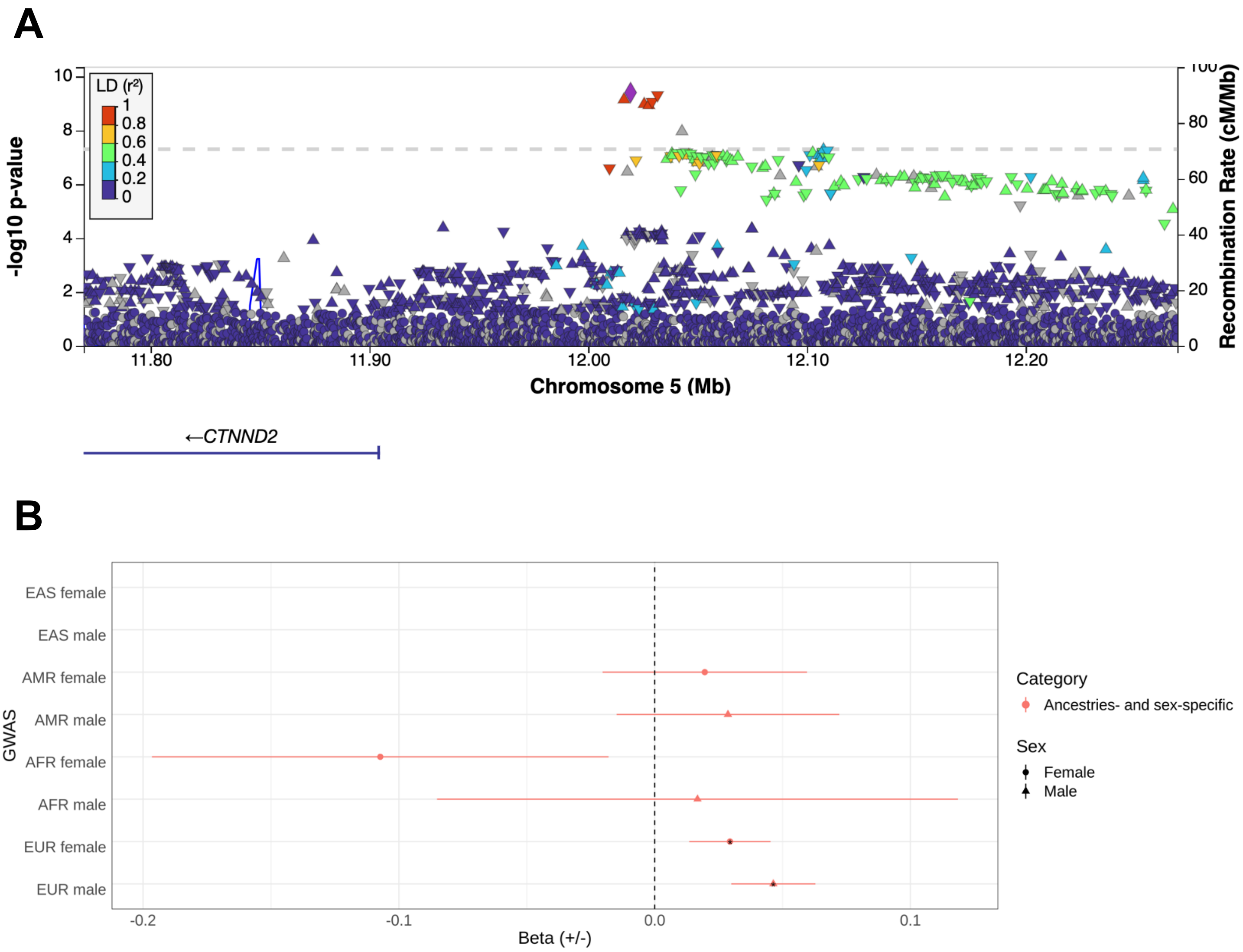

Supplementary Fig 31. Locus zoom and variant effect forest plot of rs4331897. A) Locus zoom plot of sex-combined EUR stuttering associations, where the sentinel variant is denoted in purple and surrounding variants are color coded by r2 bin using linkage disequilibrium (LD) generated from 1000 Genomes ALL reference. The x axis represents chromosome position (hg37) with annotated genes found within the region, the y axis represents $\log_{10}$ (*p*-value) of the association between the genetic variant and stuttering. Sentinel variant is upstream of *CTNND2*. B) Variant effect forest plots of rs4331897 found within the genetic ancestries of European male (EUR male), European female (EUR female), African male (AFR male), African female (AFR female), Latino/Admixed American male (AMR male), and Latino/Admixed American female (AMR female). Variant not found in East Asian male (EAS male), and East Asian female (EAS female). Male variant effects are designated by triangles, and female variant effects are designed by circles. Line length indicates standard error for the betas found in the respective GWAS. Variants reaching replicative significance, *p*-value < 8.77 x 10^{-4} (.05/57 unique loci) are indicated by asterisks.

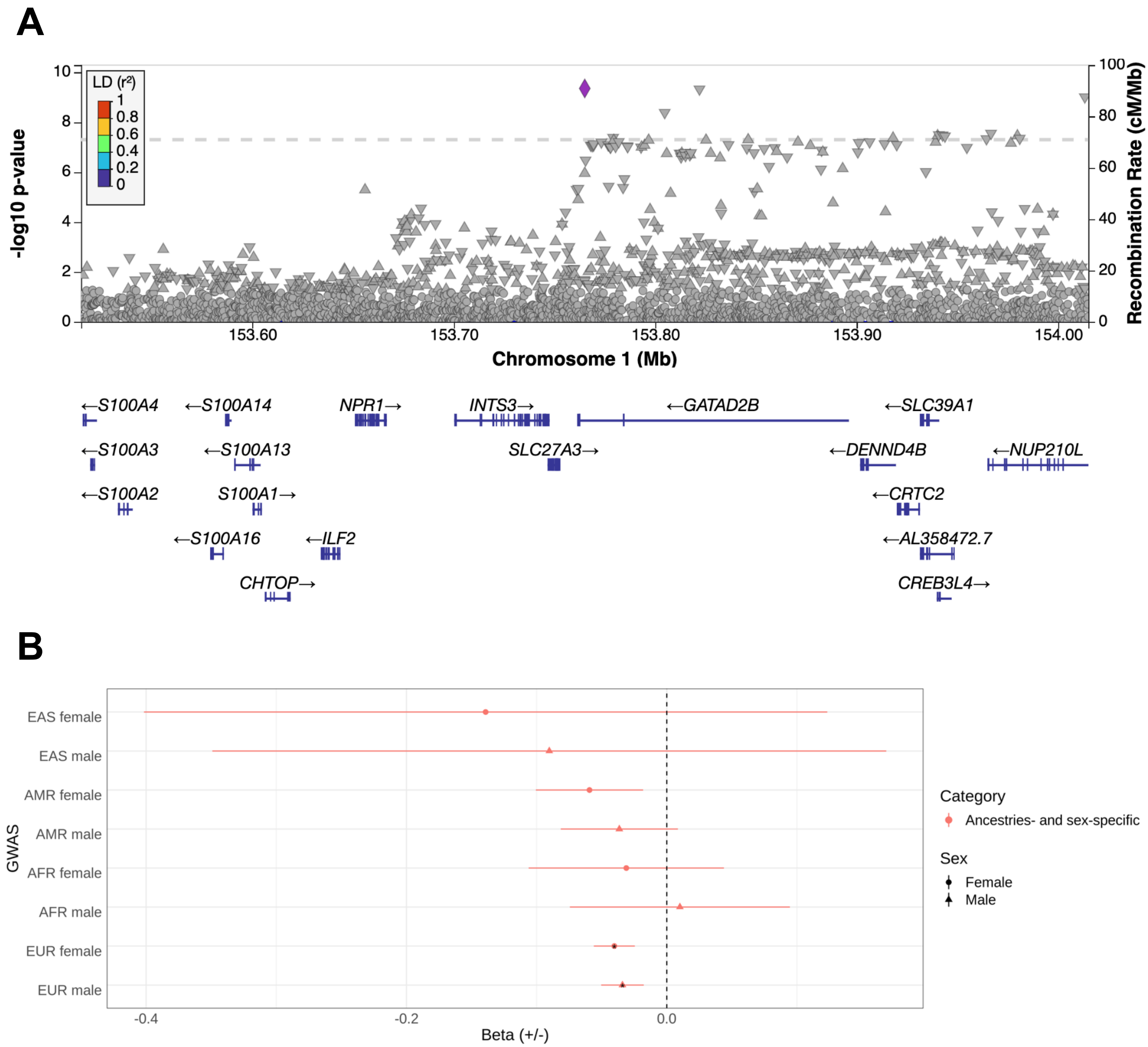

Supplementary Fig 32. Locus zoom and variant effect forest plot of rs11264408. A) Locus zoom plot of sex-combined EUR stuttering associations, where the sentinel variant is denoted in purple and surrounding variants are color coded by r2 bin using linkage disequilibrium (LD) generated from 1000 Genomes ALL reference. The x axis represents chromosome position (hg37) with annotated genes found within the region, the y axis represents $\log_{10}$ (*p*-value) of the association between the genetic variant and stuttering. Sentinel variant is within *GATAD2B*. B) Variant effect forest plots of rs11264408 found within the genetic ancestries of European male (EUR male), European female (EUR female), African male (AFR male), African female (AFR female), Latino/Admixed American male (AMR male), Latino/Admixed American female (AMR

female), East Asian male (EAS male), and East Asian female (EAS female). Male variant effects are designated by triangles, and female variant effects are designed by circles. Line length indicates standard error for the betas found in the respective GWAS. Variants reaching replicative significance, *p*-value < 8.77×10^{-4} (.05/57 unique loci) are indicated by asterisks.

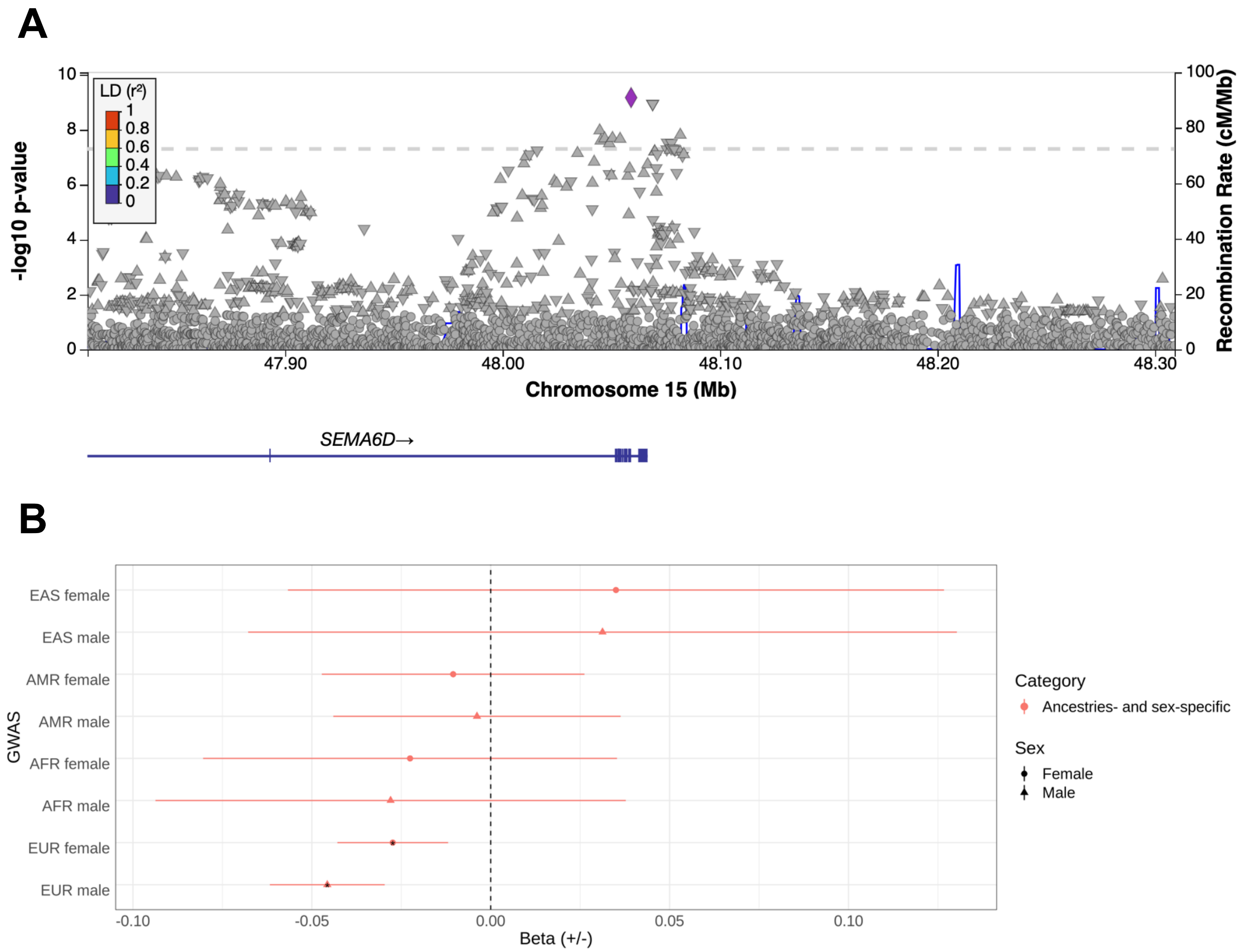

Supplementary Fig 33. Locus zoom and variant effect forest plot of rs11353659. A) Locus zoom plot of sex-combined EUR stuttering associations, where the sentinel variant is denoted in purple and surrounding variants are color coded by r2 bin using linkage disequilibrium (LD) generated from 1000 Genomes ALL reference. The x axis represents chromosome position (hg37) with annotated genes found within the region, the y axis represents $\log_{10}$ (*p-value*) of the association between the genetic variant and stuttering. Sentinel variant is a genetic downstream transcript or intronic variant within *SEMA6D*. B) Variant effect forest plots of rs11353659 found within the genetic ancestries of European male (EUR male), European female (EUR female), African male (AFR male), African female (AFR female), Latino/Admixed American male (AMR male), Latino/Admixed American female (AMR female), East Asian male (EAS male), and East Asian female (EAS female). Male variant effects are designated by triangles, and female variant effects are designed by circles. Line length indicates standard error for the betas found in the respective GWAS. Variants reaching replicative significance, *p*-value < 8.77×10^{-4} (.05/57 unique loci) are indicated by asterisks.

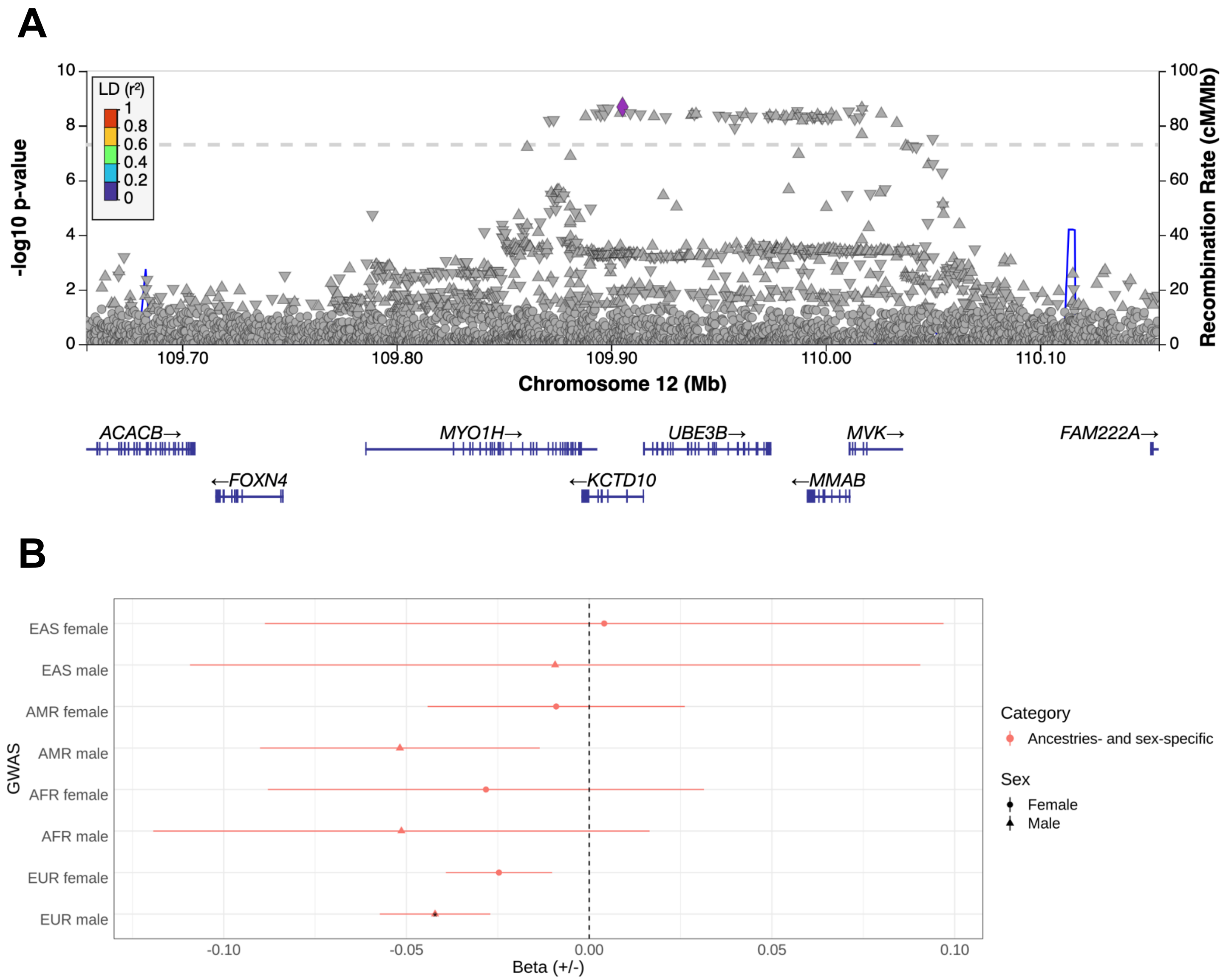

Supplementary Fig 34. Locus zoom and variant effect forest plot of rs6606725. A) Locus zoom plot of sex-combined EUR stuttering associations, where the sentinel variant is denoted in purple and surrounding variants are color coded by r2 bin using linkage disequilibrium (LD) generated from 1000 Genomes ALL reference. The x axis represents chromosome position (hg37) with annotated genes found within the region, the y axis represents $\log_{10}$ (*p*-value) of the association between the genetic variant and stuttering. Sentinel variant is within *KCTD10*. B) Variant effect forest plots of rs6606725 found within the genetic ancestries of European male (EUR male), European female (EUR female), African male (AFR male), African female (AFR female), Latino/Admixed American male (AMR male), Latino/Admixed American female (AMR female), East Asian male (EAS male), and East Asian female (EAS female). Male variant effects are designated by triangles, and female variant effects are designed by circles. Line length indicates standard error for the betas found in the respective GWAS. Variants reaching replicative significance, *p*-value < 8.77×10^{-4} (.05/57 unique loci) are indicated by asterisks.

A

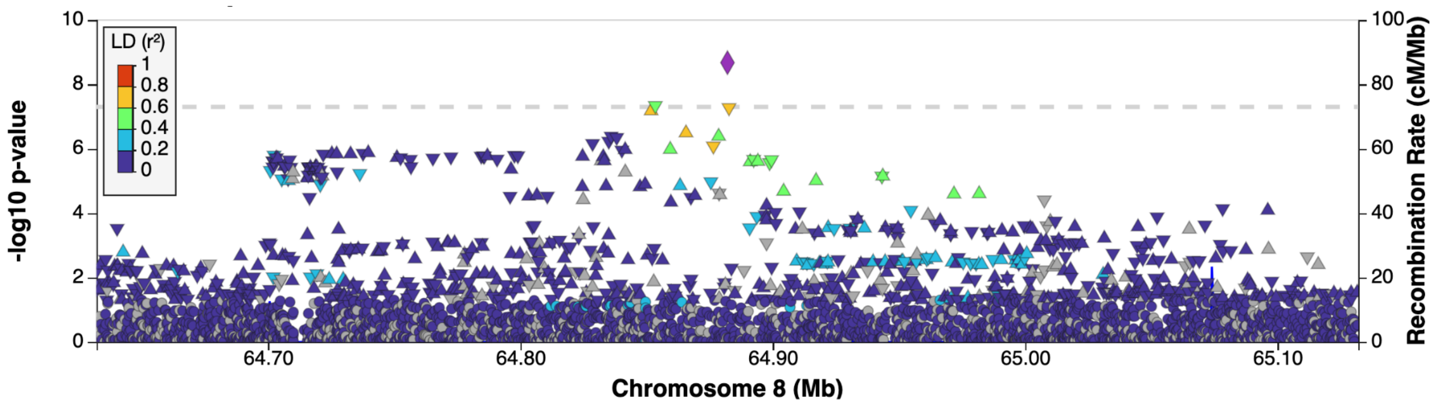

B

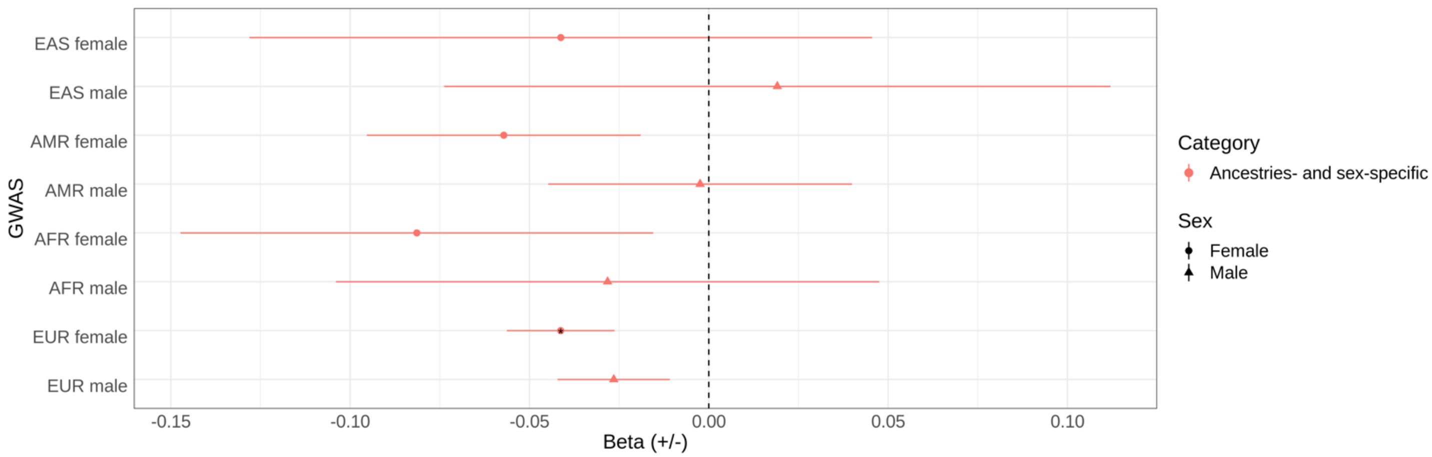

Supplementary Fig 35. Locus zoom and variant effect forest plot of rs10109510. A) Locus zoom plot of sex-combined EUR stuttering associations, where the sentinel variant is denoted in purple and surrounding variants are color coded by r2 bin using linkage disequilibrium (LD) generated from 1000 Genomes ALL reference. The x axis represents chromosome position (hg37) with annotated genes found within the region, the y axis represents $\log_{10}$ (*p*-value) of the association between the genetic variant and stuttering. Sentinel variant is located more than 500kb (upstream or downstream) from a protein-coding gene. B) Variant effect forest plots of rs10109510 found within the genetic ancestries of European male (EUR male), European female (EUR female), African male (AFR male), African female (AFR female), Latino/Admixed American male (AMR male), Latino/Admixed American female (AMR female), East Asian male (EAS male), and East Asian female (EAS female). Male variant effects are designated by triangles, and female variant effects are designed by circles. Line length indicates standard error for the betas found in the respective GWAS. Variants reaching replicative significance, *p*-value < 8.77×10^{-4} (.05/57 unique loci) are indicated by asterisks.

A

B

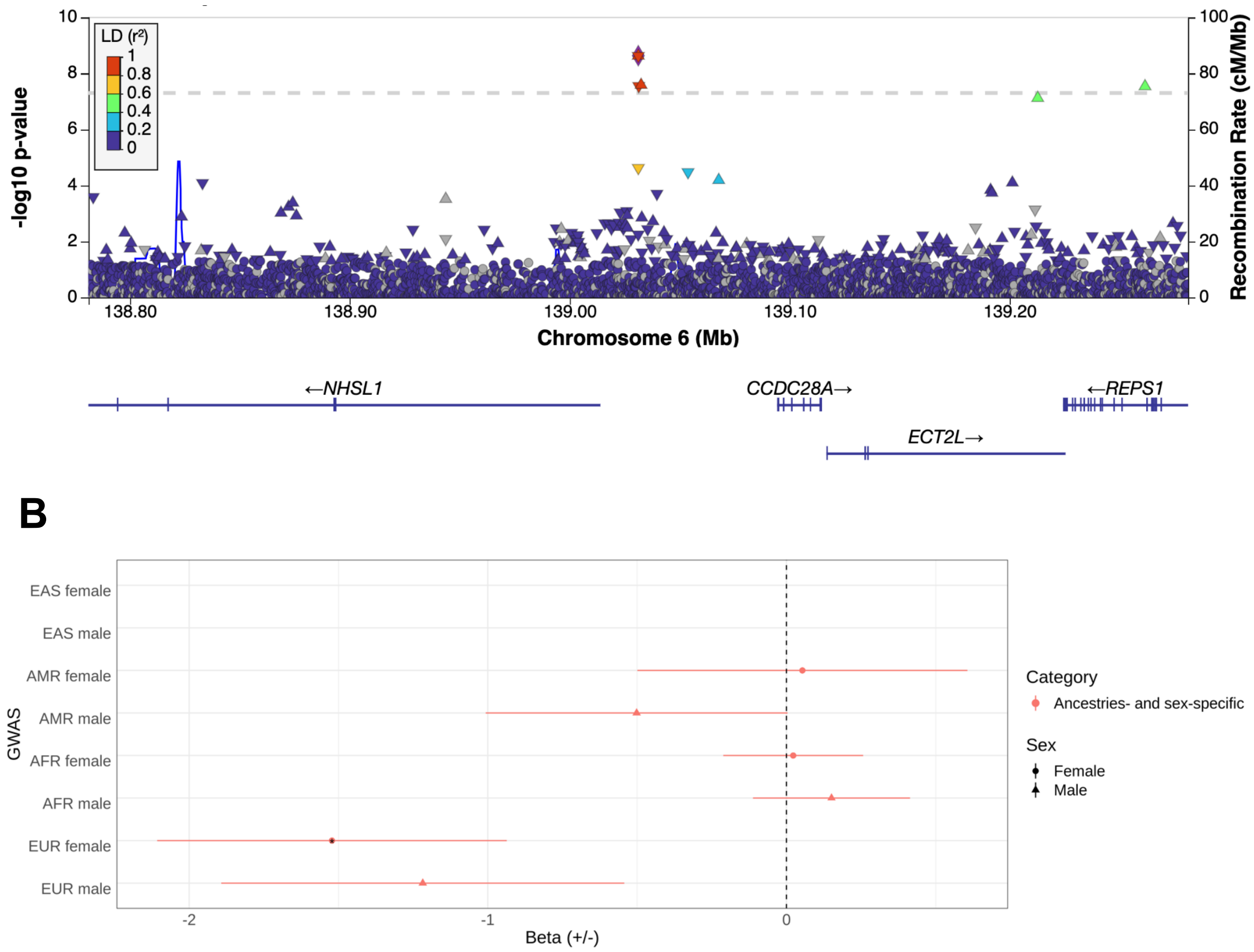

Supplementary Fig 36. Locus zoom and variant effect forest plot of rs559830614. A) Locus zoom plot of sex-combined EUR stuttering associations, where the sentinel variant is denoted in purple and surrounding variants are color coded by r2 bin using linkage disequilibrium (LD) generated from 1000 Genomes ALL reference. The x axis represents chromosome position (hg37) with annotated genes found within the region, the y axis represents $\log_{10}$ (*p*-value) of the association between the genetic variant and stuttering. Sentinel variant is between *NHSL1* and *CCDC28A*. B) Variant effect forest plots of rs559830614 found within the genetic ancestries of European male (EUR male), European female (EUR female), African male (AFR male), African female (AFR female), Latino/Admixed American male (AMR male), and Latino/Admixed American female (AMR female). Variant not found in East Asian male (EAS male), and East Asian female (EAS female). Male variant effects are designated by triangles, and female variant effects are designed by circles. Line length indicates standard error for the betas found in the

respective GWAS. Variants reaching replicative significance, *p*-value < 8.77 x 10^{-4} (.05/57 unique loci) are indicated by asterisks.

A

B

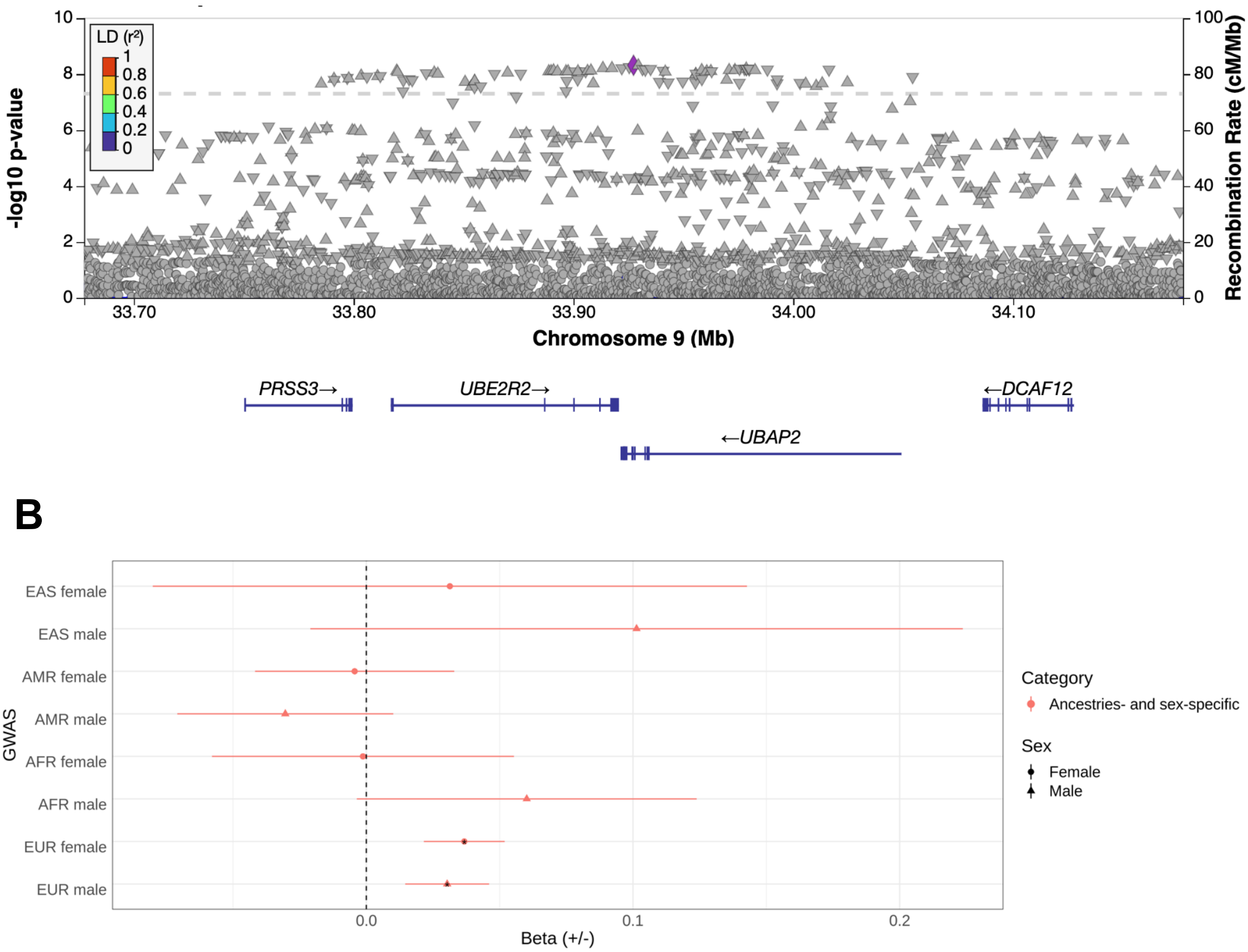

Supplementary Fig 37. Locus zoom and variant effect forest plot of rs745532. A) Locus zoom plot of sex-combined EUR stuttering associations, where the sentinel variant is denoted in purple and surrounding variants are color coded by r2 bin using linkage disequilibrium (LD) generated from 1000 Genomes ALL reference. The x axis represents chromosome position (hg37) with annotated genes found within the region, the y axis represents $\log_{10}$ (*p*-value) of the association between the genetic variant and stuttering. Sentinel variant is a transcript or intronic variant within *UBAP2*. B) Variant effect forest plots of rs745532 found within the genetic ancestries of European male (EUR male), European female (EUR female), African male (AFR male), African female (AFR female), Latino/Admixed American male (AMR male), Latino/Admixed American female (AMR female), East Asian male (EAS male), and East Asian female (EAS female). Male variant effects are designated by triangles, and female variant effects are designed by circles. Line length indicates standard error for the betas found in the

respective GWAS. Variants reaching replicative significance, *p*-value < 8.77×10^{-4} (.05/57 unique loci) are indicated by asterisks.

A

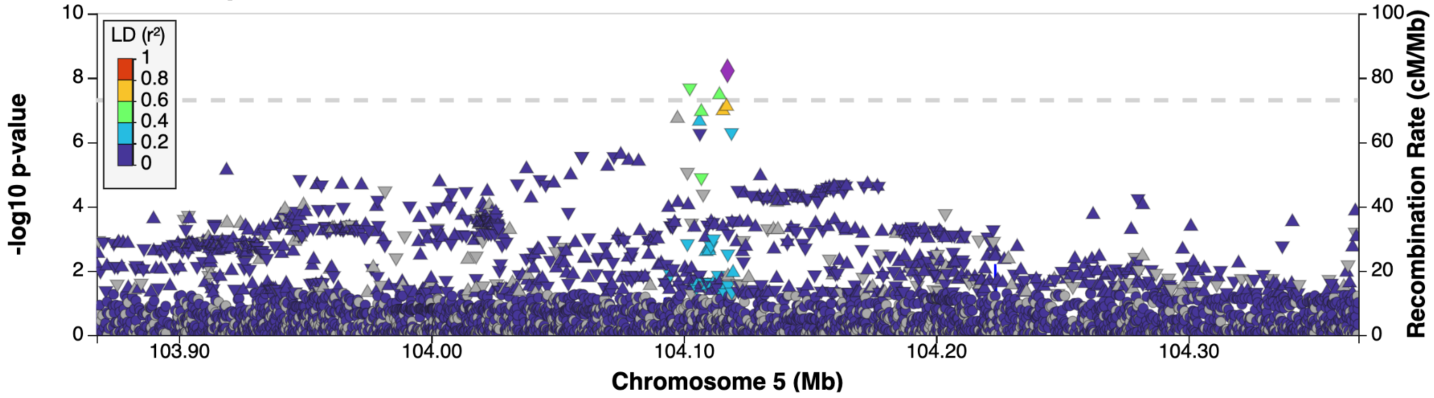

B

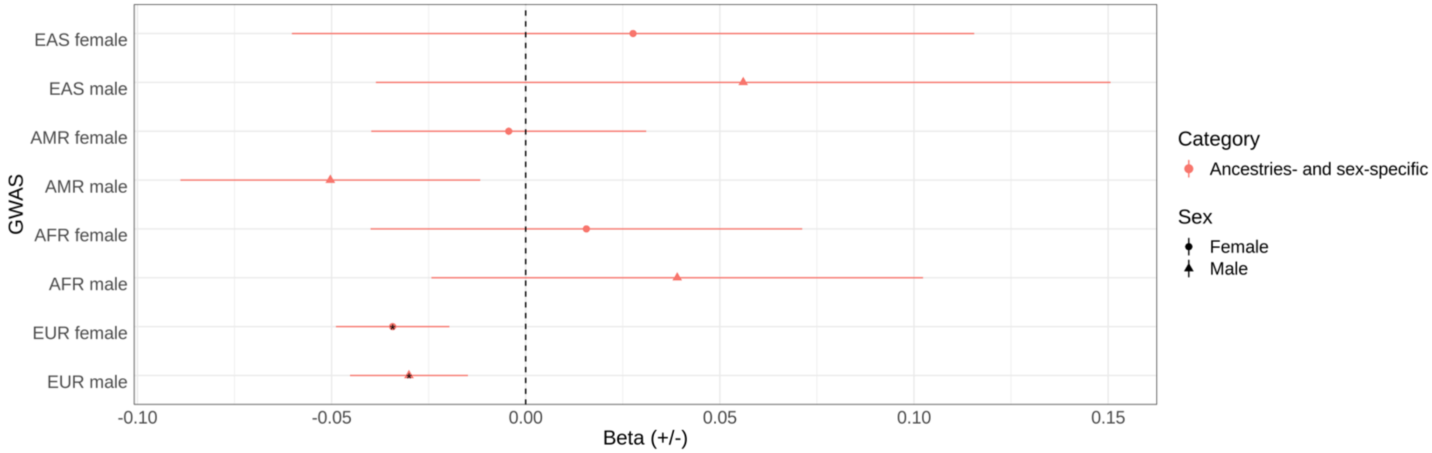

Supplementary Fig 38. Locus zoom and variant effect forest plot of rs1836999. A) Locus zoom plot of sex-combined EUR stuttering associations, where the sentinel variant is denoted in purple and surrounding variants are color coded by r2 bin using linkage disequilibrium (LD) generated from 1000 Genomes ALL reference. The x axis represents chromosome position (hg37) with annotated genes found within the region, the y axis represents $\log_{10}$ (*p*-value) of the association between the genetic variant and stuttering. Sentinel variant is located more than 500kb (upstream or downstream) from a protein-coding gene. B) Variant effect forest plots of rs1836999 found within the genetic ancestries of European male (EUR male), European female (EUR female), African male (AFR male), African female (AFR female), Latino/Admixed American male (AMR male), Latino/Admixed American female (AMR female), East Asian male (EAS male), and East Asian female (EAS female). Male variant effects are designated by triangles, and female variant effects are designed by circles. Line length indicates standard error for the betas found in the respective GWAS. Variants reaching replicative significance, *p*-value < 8.77×10^{-4} (.05/57 unique loci) are indicated by asterisks.

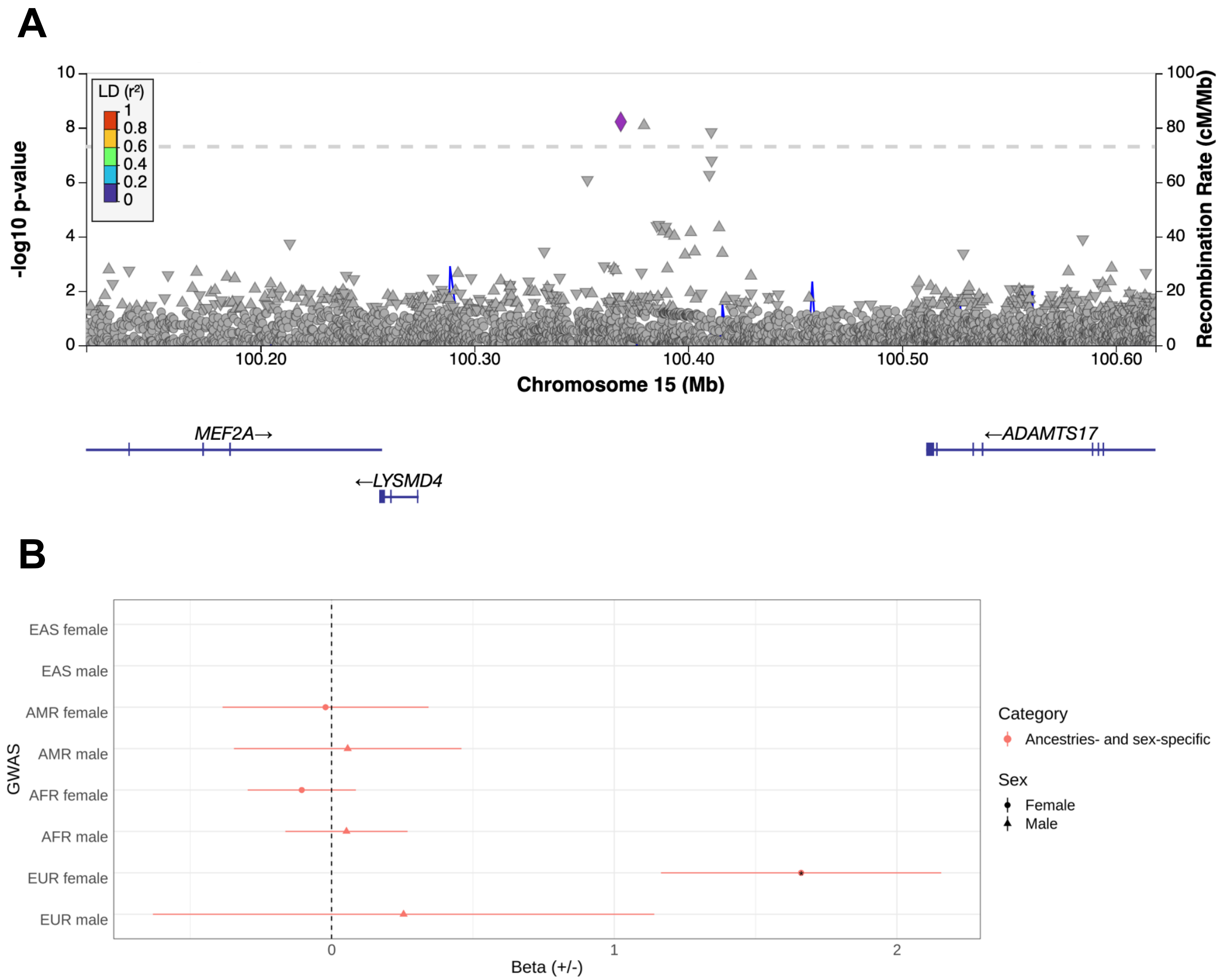

Supplementary Fig 39. Locus zoom and variant effect forest plot of rs147393141. A) Locus zoom plot of sex-combined EUR stuttering associations, where the sentinel variant is denoted in purple and surrounding variants are color coded by r2 bin using linkage disequilibrium (LD) generated from 1000 Genomes ALL reference. The x axis represents chromosome position (hg37) with annotated genes found within the region, the y axis represents $\log_{10}$ (*p*-value) of the association between the genetic variant and stuttering. Sentinel variant is between *LYSMD4* and *ADAMTS17*. B) Variant effect forest plots of rs147393141 found within the genetic ancestries of European male (EUR male), European female (EUR female), African male (AFR male), African female (AFR female), Latino/Admixed American male (AMR male), and Latino/Admixed American female (AMR female). Variant not found in East Asian male (EAS male), and East Asian female (EAS female). Male variant effects are designated by triangles, and female variant effects are designed by circles. Line length indicates standard error for the betas

found in the respective GWAS. Variants reaching replicative significance, *p*-value < 8.77×10^{-4} (.05/57 unique loci) are indicated by asterisks.

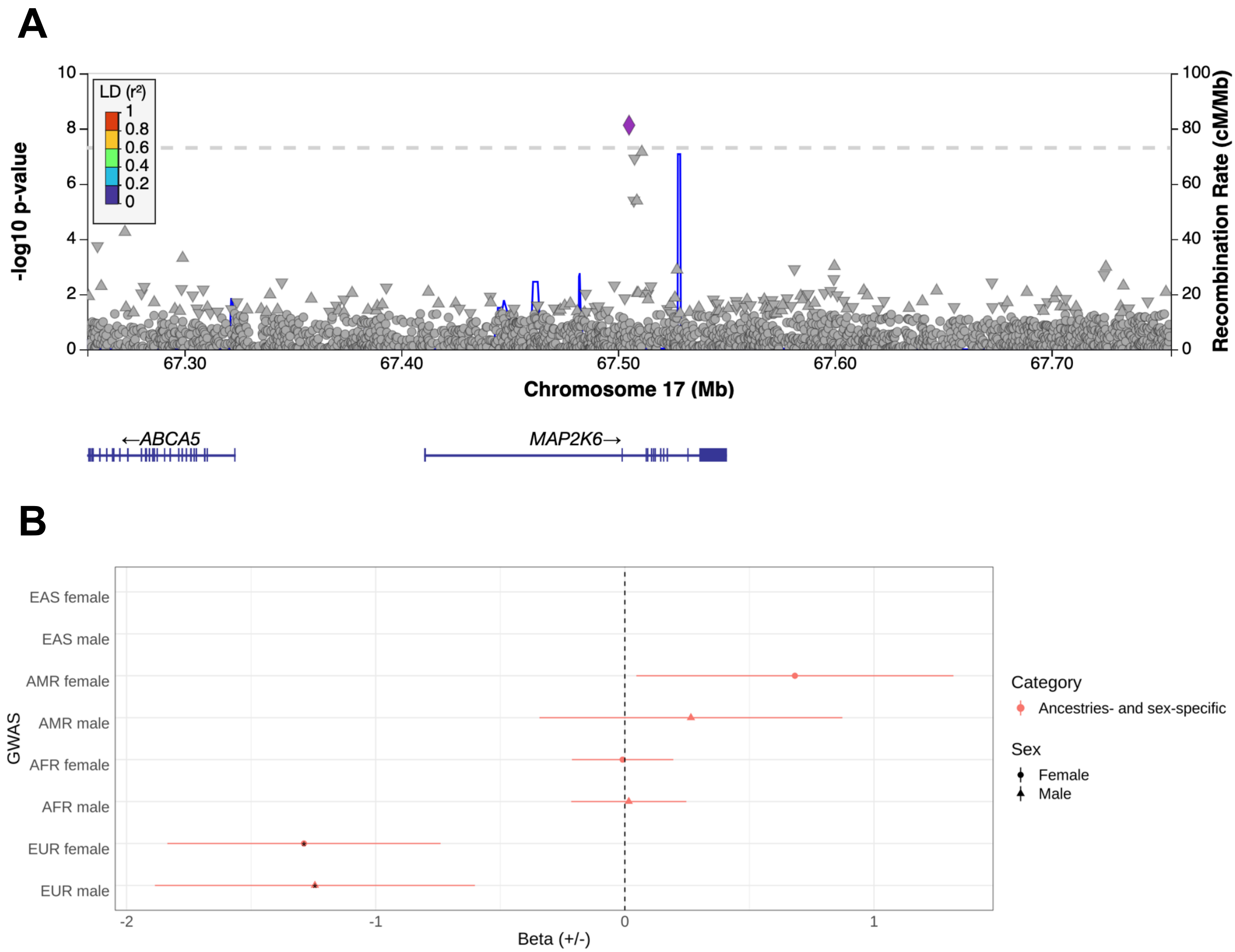

Supplementary Fig 40. Locus zoom and variant effect forest plot of rs59183769. A) Locus zoom plot of sex-combined EUR stuttering associations, where the sentinel variant is denoted in purple and surrounding variants are color coded by r2 bin using linkage disequilibrium (LD) generated from 1000 Genomes ALL reference. The x axis represents chromosome position (hg37) with annotated genes found within the region, the y axis represents $\log_{10}$ (*p*-value) of the association between the genetic variant and stuttering. Sentinel variant is a transcript or intronic variant within *MAP2K6*. B) Variant effect forest plots of rs59183769 found within the genetic ancestries of European male (EUR male), European female (EUR female), African male (AFR male), African female (AFR female), Latino/Admixed American male (AMR male), and Latino/Admixed American female (AMR female). Variant not found in East Asian male (EAS male), and East Asian female (EAS female). Male variant effects are designated by triangles, and female variant effects are designed by circles. Line length indicates standard error for the betas found in the respective GWAS. Variants reaching replicative significance, *p*-value < 8.77×10^{-4} (.05/57 unique loci) are indicated by asterisks.

A

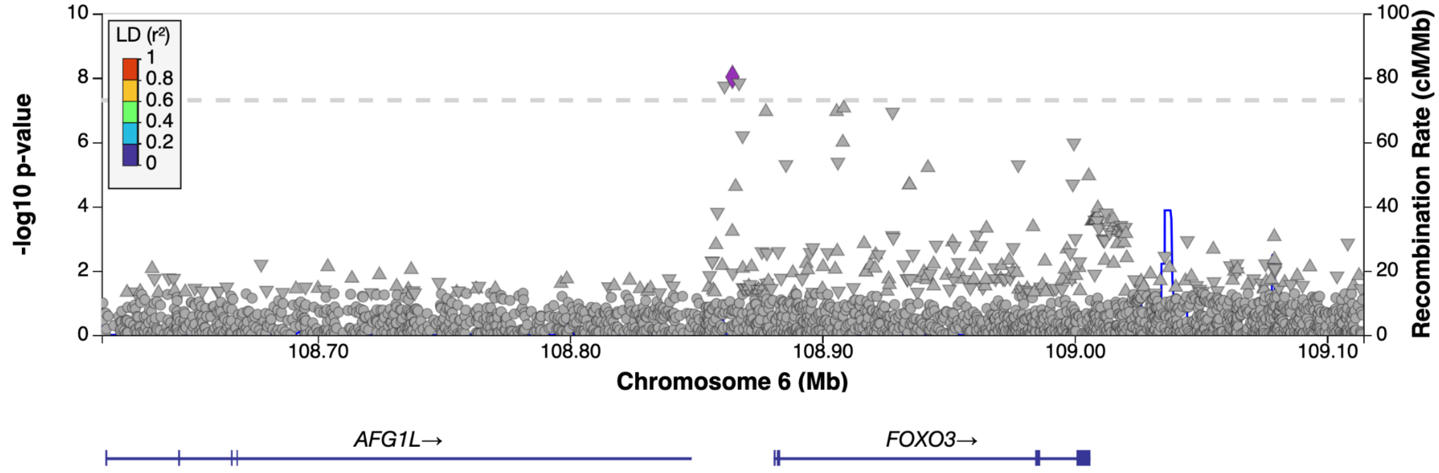

B

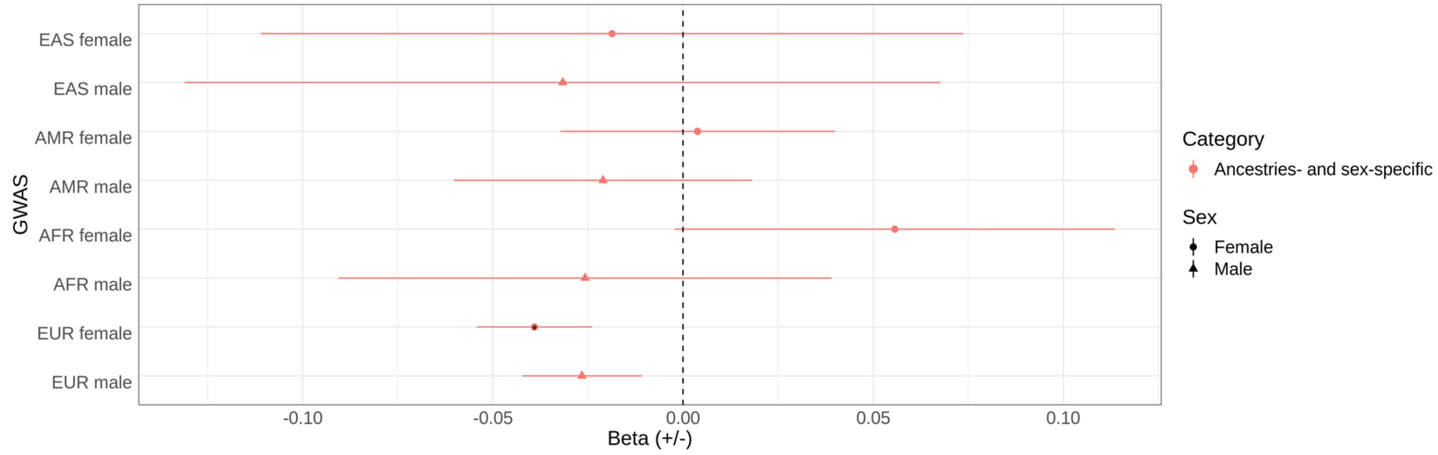

Supplementary Fig 41. Locus zoom and variant effect forest plot of rs9384679. A) Locus zoom plot of sex-combined EUR stuttering associations, where the sentinel variant is denoted in purple and surrounding variants are color coded by r2 bin using linkage disequilibrium (LD) generated from 1000 Genomes ALL reference. The x axis represents chromosome position (hg37) with annotated genes found within the region, the y axis represents $\log_{10}$ (*p*-value) of the association between the genetic variant and stuttering. Sentinel variant is between *AFG1L* and *FOXO3*. B) Variant effect forest plots of rs9384679 found within the genetic ancestries of European male (EUR male), European female (EUR female), African male (AFR male), African female (AFR female), Latino/Admixed American male (AMR male), Latino/Admixed American female (AMR female), East Asian male (EAS male), and East Asian female (EAS female). Male variant effects are designated by triangles, and female variant effects are designed by circles. Line length indicates standard error for the betas found in the respective GWAS. Variants reaching replicative significance, *p*-value < 8.77 x 10^{-4} (.05/57 unique loci) are indicated by asterisks.

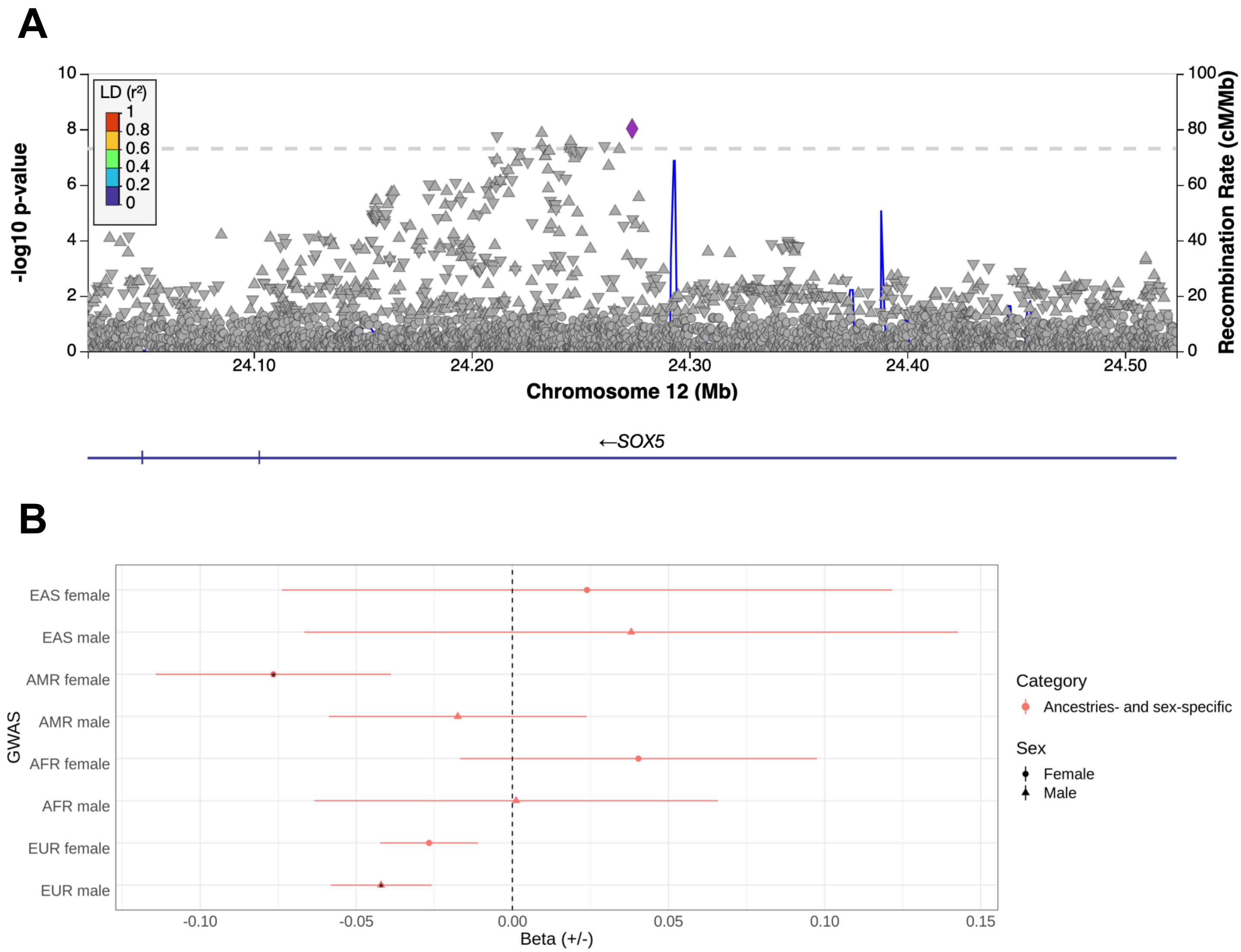

Supplementary Fig 42. Locus zoom and variant effect forest plot of rs7139049. A) Locus zoom plot of sex-combined EUR stuttering associations, where the sentinel variant is denoted in purple and surrounding variants are color coded by r2 bin using linkage disequilibrium (LD) generated from 1000 Genomes ALL reference. The x axis represents chromosome position (hg37) with annotated genes found within the region, the y axis represents $\log_{10}$ (*p*-value) of the association between the genetic variant and stuttering. Sentinel variant is a transcript or intronic variant within *SOX5*. B) Variant effect forest plots of rs7139049 found within the genetic ancestries of European male (EUR male), European female (EUR female), African male (AFR male), African female (AFR female), Latino/Admixed American male (AMR male), Latino/Admixed American female (AMR female), East Asian male (EAS male), and East Asian female (EAS female). Male variant effects are designated by triangles, and female variant effects are designed by circles. Line length indicates standard error for the betas found in the respective GWAS. Variants reaching replicative significance, *p*-value < 8.77×10^{-4} (.05/57 unique loci) are indicated by asterisks.

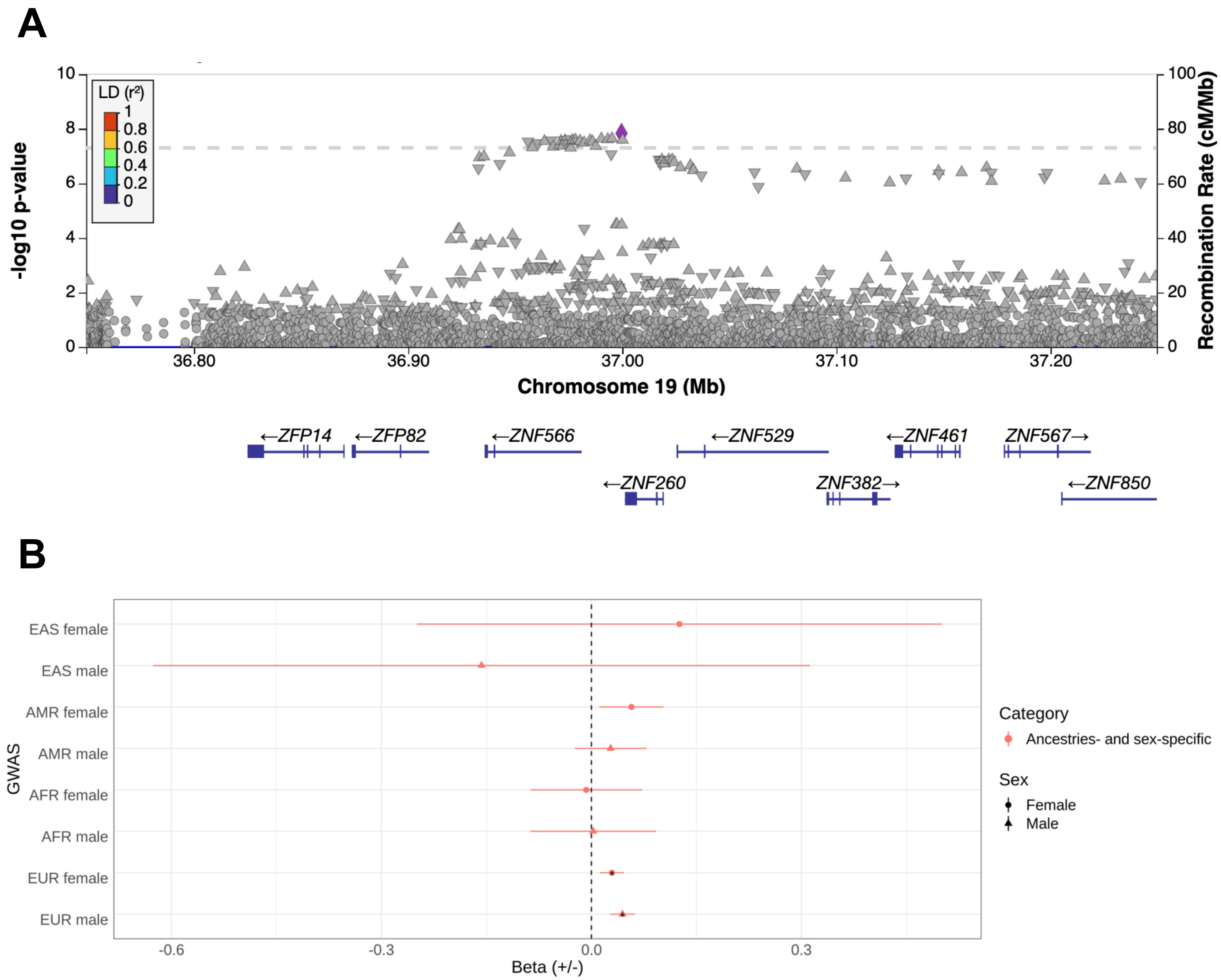

Supplementary Fig 43. Locus zoom and variant effect forest plot of rs8100340. A) Locus zoom plot of sex-combined EUR stuttering associations, where the sentinel variant is denoted in purple and surrounding variants are color coded by r2 bin using linkage disequilibrium (LD) generated from 1000 Genomes ALL reference. The x axis represents chromosome position (hg37) with annotated genes found within the region, the y axis represents $\log_{10}$ (*p*-value) of the association between the genetic variant and stuttering. Sentinel variant is between *ZNF566* and *ZNF260*. B) Variant effect forest plots of rs8100340 found within the genetic ancestries of European male (EUR male), European female (EUR female), African male (AFR male), African female (AFR female), Latino/Admixed American male (AMR male), Latino/Admixed American female (AMR female), East Asian male (EAS male), and East Asian female (EAS female). Male variant effects are designated by triangles, and female variant effects are designed by circles. Line length indicates standard error for the betas found in the respective GWAS. Variants

reaching replicative significance, p-value $< 8.77 \times 10^{-4}$ (.05/57 unique loci) are indicated by asterisks.

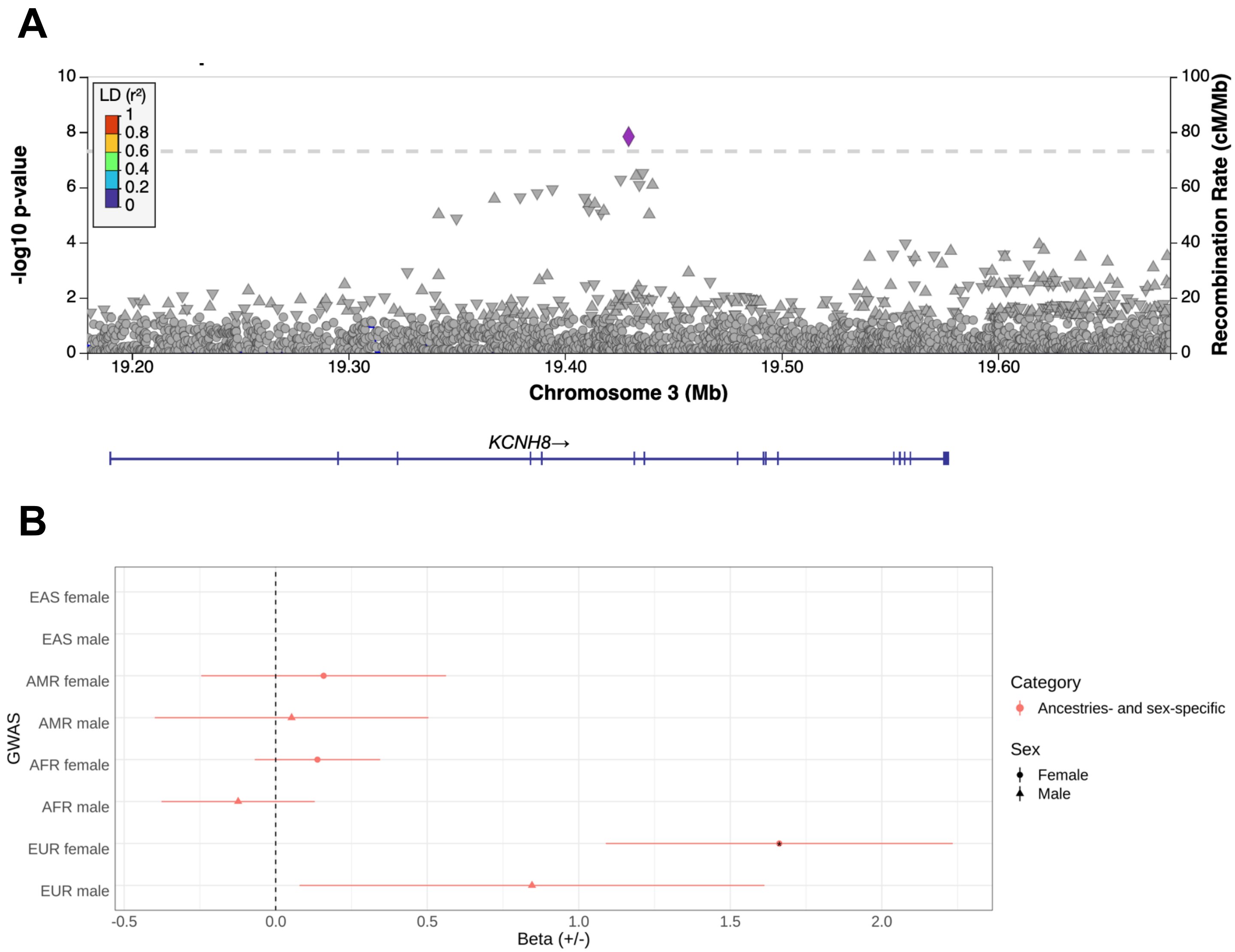

Supplementary Fig 44. Locus zoom and variant effect forest plot of rs182518401. A) Locus zoom plot of sex-combined EUR stuttering associations, where the sentinel variant is denoted in purple and surrounding variants are color coded by r2 bin using linkage disequilibrium (LD) generated from 1000 Genomes ALL reference. The x axis represents chromosome position (hg37) with annotated genes found within the region, the y axis represents $\log_{10}$ (*p*-value) of the association between the genetic variant and stuttering. Sentinel variant is an intronic variant within *KCNH8*. B) Variant effect forest plots of rs182518401 found within the genetic ancestries of European male (EUR male), European female (EUR female), African male (AFR male), African female (AFR female), Latino/Admixed American male (AMR male), and Latino/Admixed American female (AMR female). Variant not found in East Asian male (EAS male), and East Asian female (EAS female). Male variant effects are designated by triangles, and female variant effects are designed by circles. Line length indicates standard error for the betas found in the respective GWAS. Variants reaching replicative significance, *p*-value $< 8.77 \times 10^{-4}$ (.05/57 unique loci) are indicated by asterisks.

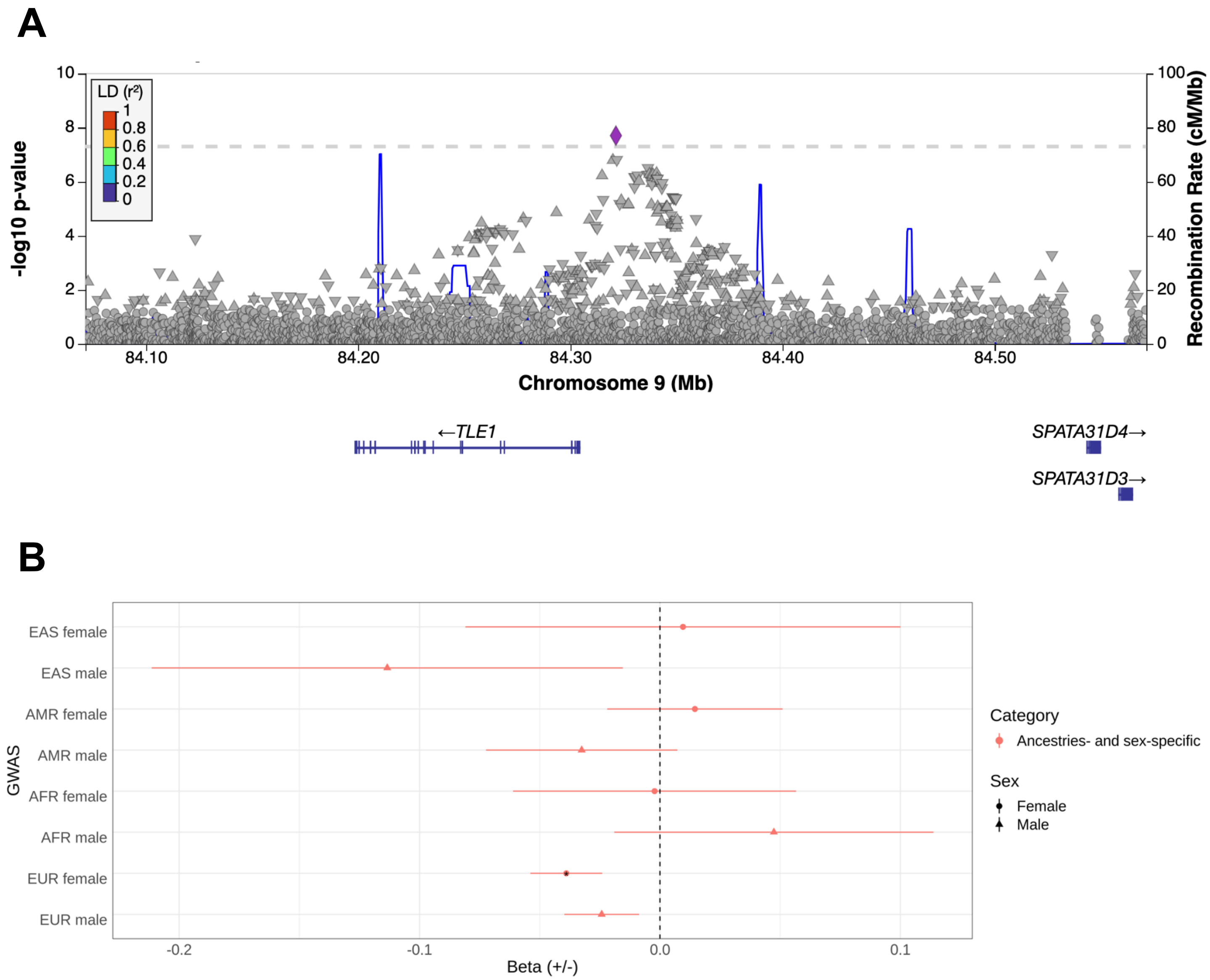

Supplementary Fig 45. Locus zoom and variant effect forest plot of rs537077603. A) Locus zoom plot of sex-combined EUR stuttering associations, where the sentinel variant is denoted in purple and surrounding variants are color coded by r2 bin using linkage disequilibrium (LD) generated from 1000 Genomes ALL reference. The x axis represents chromosome position (hg37) with annotated genes found within the region, the y axis represents $\log_{10}$ (*p*-value) of the association between the genetic variant and stuttering. Sentinel variant is between *TLE1* and *SPATA31D4*. B) Variant effect forest plots of rs537077603 found within the genetic ancestries of European male (EUR male), European female (EUR female), African male (AFR male), African female (AFR female), Latino/Admixed American male (AMR male), Latino/Admixed American female (AMR female), East Asian male (EAS male), and East Asian female (EAS female). Male variant effects are designated by triangles, and female variant effects are designed by circles. Line length indicates standard error for the betas found in the

respective GWAS. Variants reaching replicative significance, *p*-value < 8.77×10^{-4} (.05/57 unique loci) are indicated by asterisks.

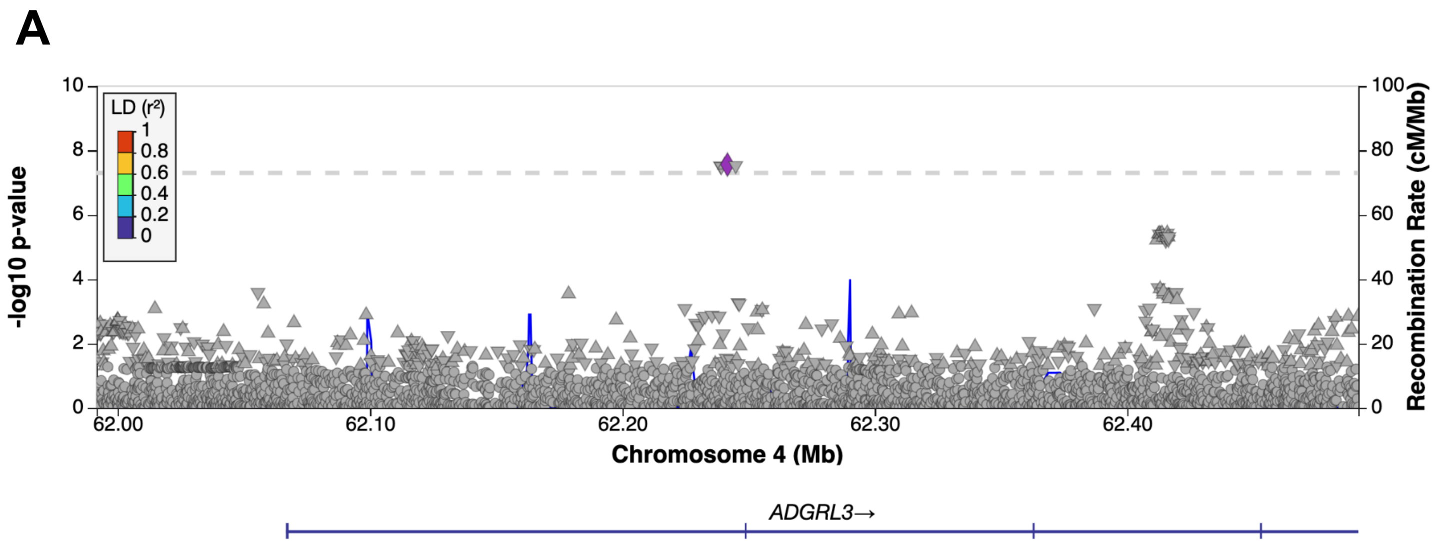

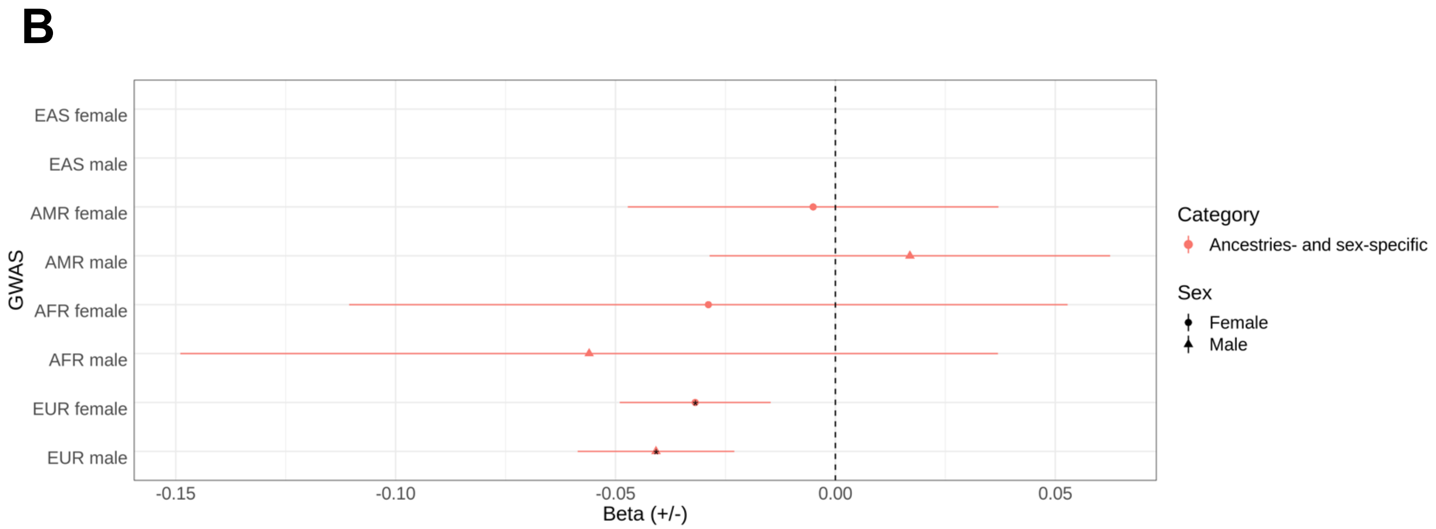

Supplementary Fig 46. Locus zoom and variant effect forest plot of rs11931307. A) Locus zoom plot of sex-combined EUR stuttering associations, where the sentinel variant is denoted in purple and surrounding variants are color coded by r2 bin using linkage disequilibrium (LD) generated from 1000 Genomes ALL reference. The x axis represents chromosome position (hg37) with annotated genes found within the region, the y axis represents $\log_{10}$ (*p*-value) of the association between the genetic variant and stuttering. Sentinel variant is a genetic upstream transcript or intronic variant within *ADGRL3*. B) Variant effect forest plots of rs11931307 found within the genetic ancestries of European male (EUR male), European female (EUR female), African male (AFR male), African female (AFR female), Latino/Admixed American male (AMR male), and Latino/Admixed American female (AMR female). Variant not found in East Asian male (EAS male), and East Asian female (EAS female). Male variant effects are designated by triangles, and female variant effects are designed by circles. Line length indicates

standard error for the betas found in the respective GWAS. Variants reaching replicative significance, *p*-value < 8.77×10^{-4} (.05/57 unique loci) are indicated by asterisks.

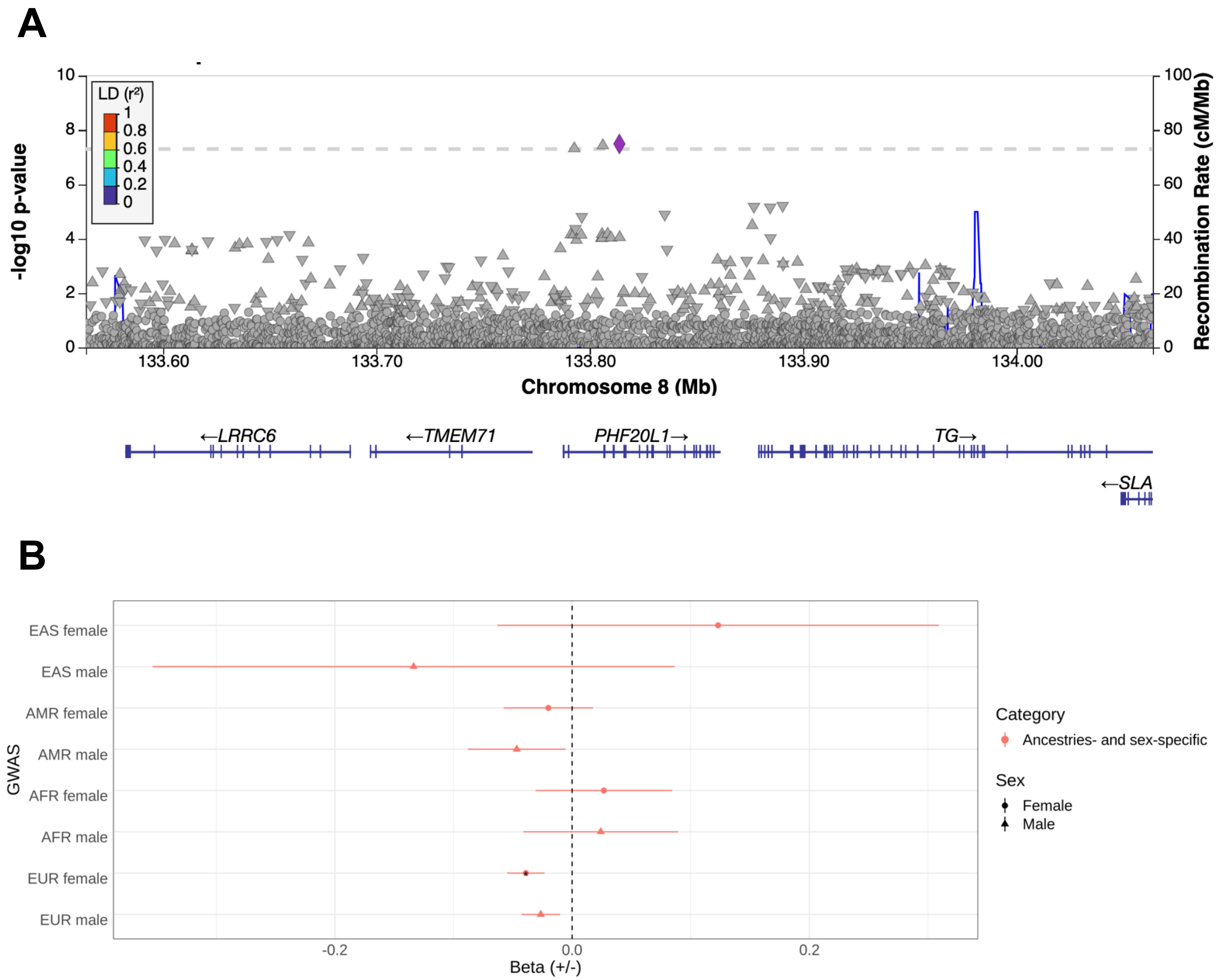

Supplementary Fig 47. Locus zoom and variant effect forest plot of rs235426. A) Locus zoom plot of sex-combined EUR stuttering associations, where the sentinel variant is denoted in purple and surrounding variants are color coded by r2 bin using linkage disequilibrium (LD) generated from 1000 Genomes ALL reference. The x axis represents chromosome position (hg37) with annotated genes found within the region, the y axis represents $\log_{10}$ (*p*-value) of the association between the genetic variant and stuttering. Sentinel variant is a genetic upstream transcript or intronic variant within *PHF20L1*. B) Variant effect forest plots of rs235426 found within the genetic ancestries of European male (EUR male), European female (EUR female), African male (AFR male), African female (AFR female), Latino/Admixed American male (AMR male), Latino/Admixed American female (AMR female), East Asian male (EAS male), and East Asian female (EAS female). Male variant effects are designated by triangles, and female variant effects are designed by circles. Line length indicates standard error for the betas

found in the respective GWAS. Variants reaching replicative significance, p-value < 8.77 x 10^{-4} (.05/57 unique loci) are indicated by asterisks.

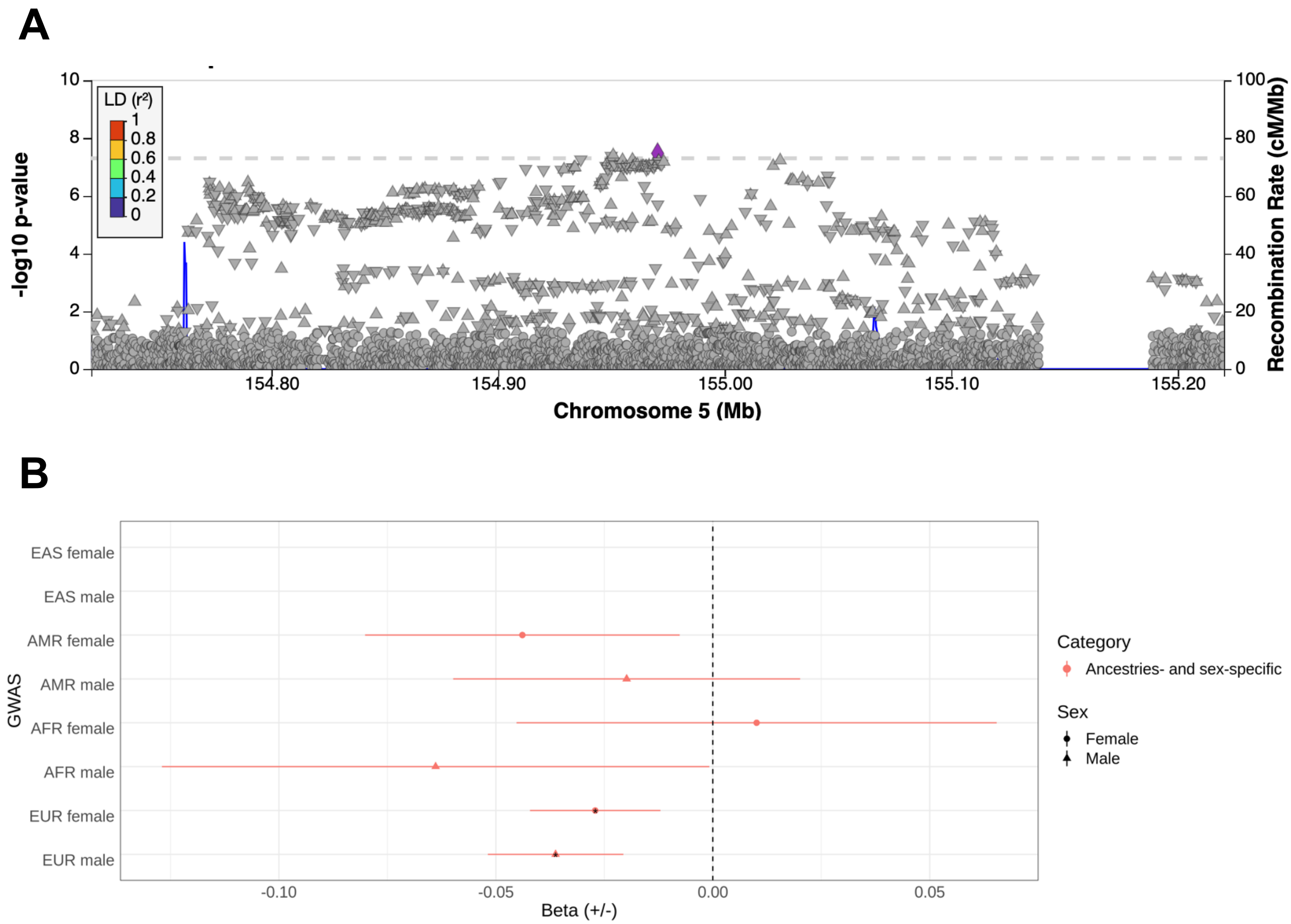

Supplementary Fig 48. Locus zoom and variant effect forest plot of rs11167732. A) Locus zoom plot of sex-combined EUR stuttering associations, where the sentinel variant is denoted in purple and surrounding variants are color coded by r2 bin using linkage disequilibrium (LD) generated from 1000 Genomes ALL reference. The x axis represents chromosome position (hg37) with annotated genes found within the region, the y axis represents $\log_{10}$ (*p*-value) of the association between the genetic variant and stuttering. Sentinel variant is located more than 500kb (upstream or downstream) from a protein-coding gene. B) Variant effect forest plots of rs11167732 found within the genetic ancestries of European male (EUR male), European female (EUR female), African male (AFR male), African female (AFR female), Latino/Admixed American male (AMR male), and Latino/Admixed American female (AMR female). Variant not found in East Asian male (EAS male), and East Asian female (EAS female). Male variant effects are designated by triangles, and female variant effects are designed by circles. Line length indicates standard error for the betas found in the respective GWAS. Variants reaching replicative significance, *p*-value < 8.77×10^{-4} (.05/57 unique loci) are indicated by asterisks.

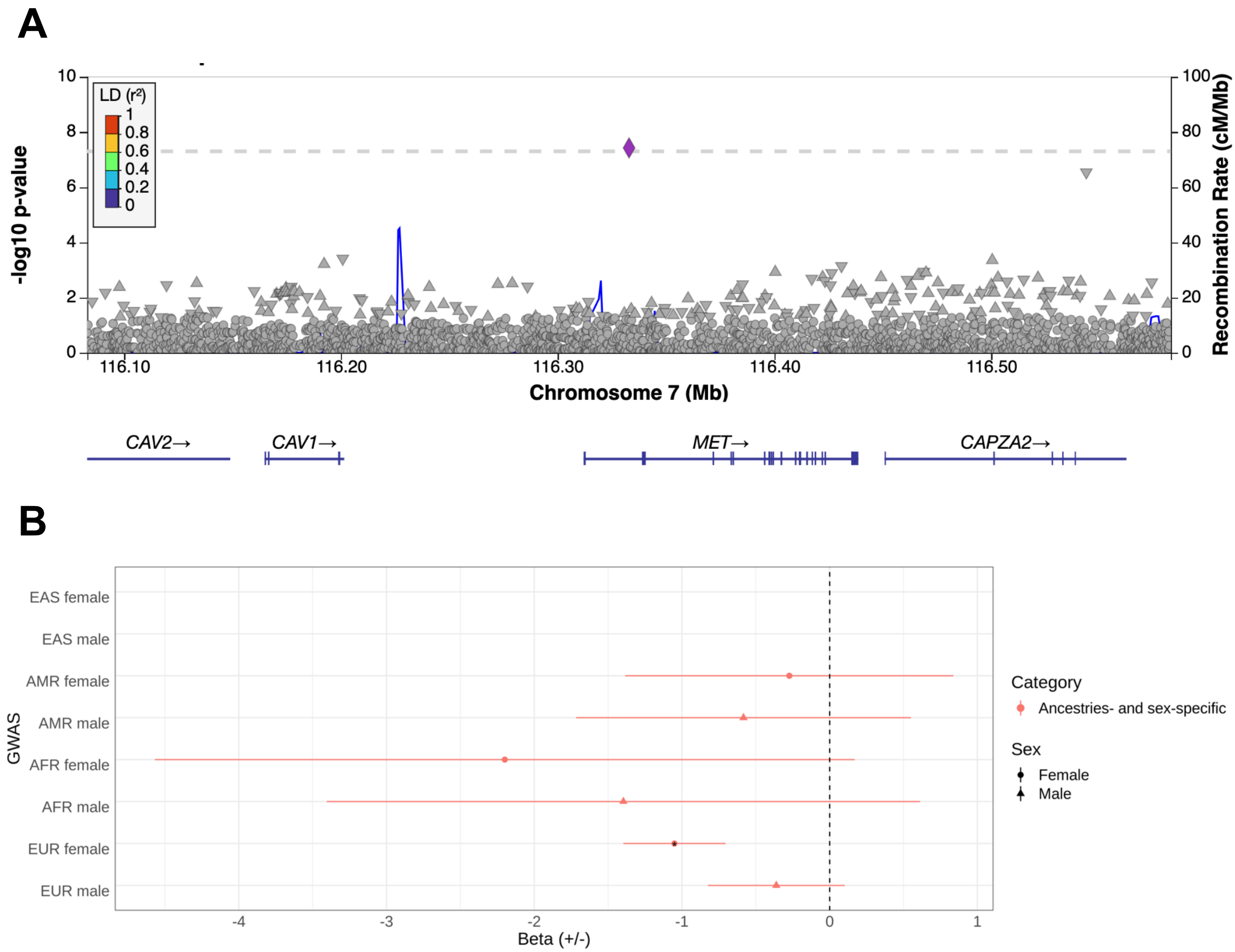

Supplementary Fig 49. Locus zoom and variant effect forest plot of rs758717390. A) Locus zoom plot of sex-combined EUR stuttering associations, where the sentinel variant is denoted in purple and surrounding variants are color coded by r2 bin using linkage disequilibrium (LD) generated from 1000 Genomes ALL reference. The x axis represents chromosome position (hg37) with annotated genes found within the region, the y axis represents $\log_{10}$ (*p*-value) of the association between the genetic variant and stuttering. Sentinel variant is an intronic variant within *MET*. B) Variant effect forest plots of rs758717390 found within the genetic ancestries of European male (EUR male), European female (EUR female), African male (AFR male), African female (AFR female), Latino/Admixed American male (AMR male), and Latino/Admixed American female (AMR female). Variant not found in East Asian male (EAS male), and East Asian female (EAS female). Male variant effects are designated by triangles, and female variant effects are designed by circles. Line length indicates standard error for the betas found in the respective GWAS. Variants reaching replicative significance, *p*-value < 8.77×10^{-4} (.05/57 unique loci) are indicated by asterisks.

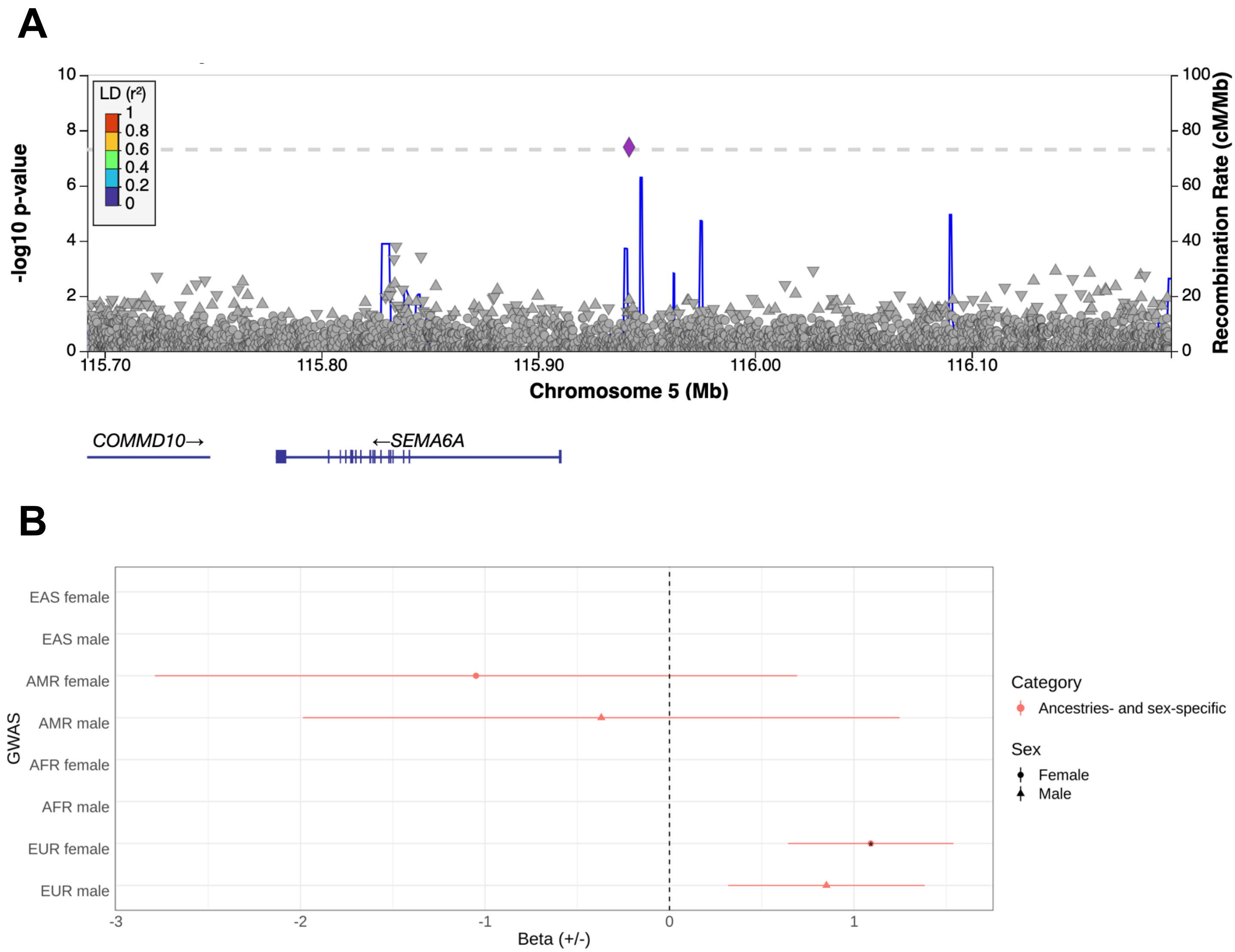

Supplementary Fig 50. Locus zoom and variant effect forest plot of 5:115941955. A) Locus zoom plot of sex-combined EUR stuttering associations, where the sentinel variant is denoted in purple and surrounding variants are color coded by r2 bin using linkage disequilibrium (LD) generated from 1000 Genomes ALL reference. The x axis represents chromosome position (hg37) with annotated genes found within the region, the y axis represents $\log_{10}$ (*p*-value) of the association between the genetic variant and stuttering. Sentinel variant is upstream of *SEMA6A*. B) Variant effect forest plots of 5:115941955 found within the genetic ancestries of European male (EUR male), European female (EUR female), Latino/Admixed American male (AMR male), and Latino/Admixed American female (AMR female). Variant not found in African male (AFR male), African female (AFR female), East Asian male (EAS male), and East Asian female (EAS female). Male variant effects are designated by triangles, and female variant effects are designed by circles. Line length indicates standard error for the betas found in the respective GWAS. Variants reaching replicative significance, *p*-value < 8.77 x 10^{-4} (.05/57 unique loci) are indicated by asterisks.

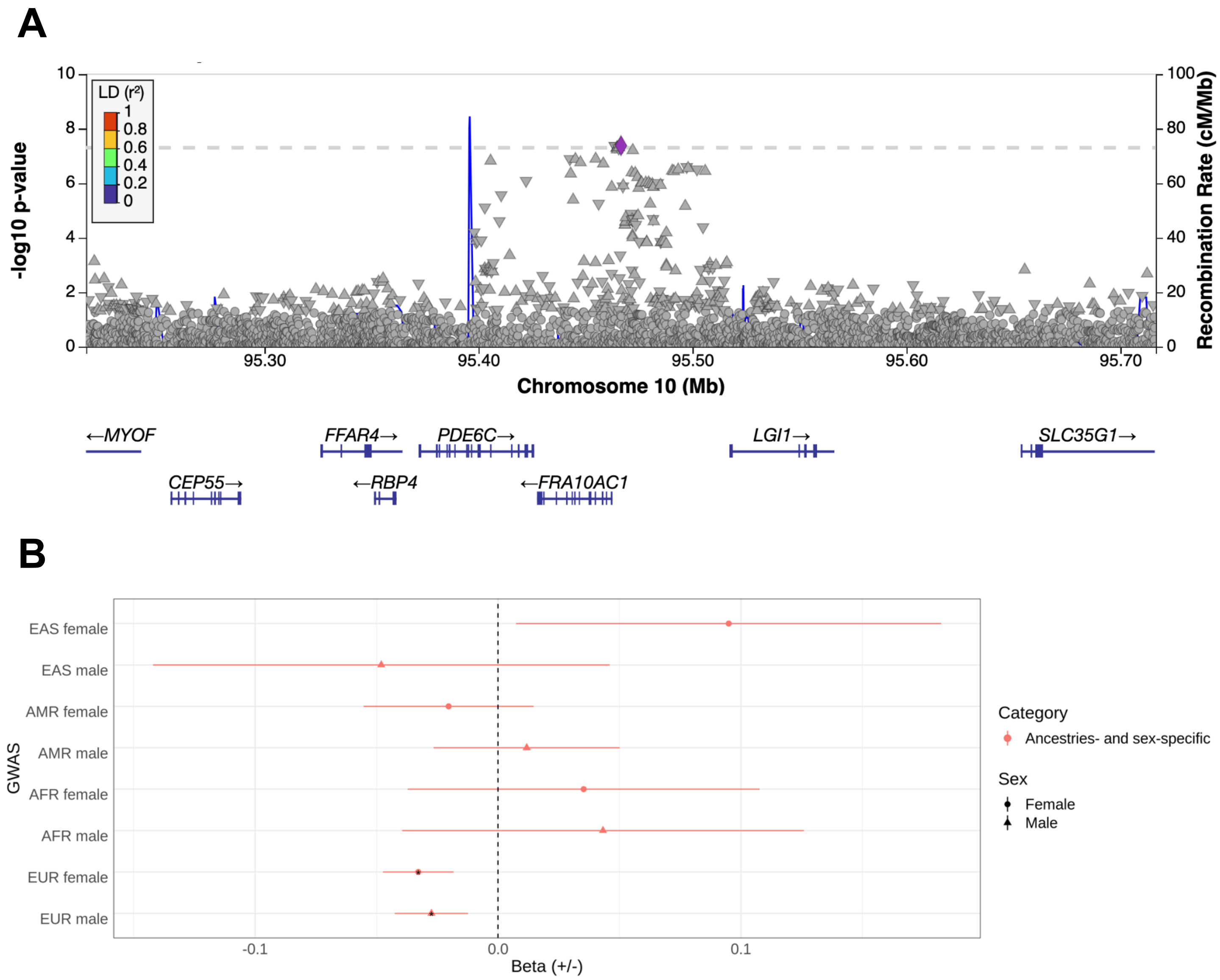

Supplementary Fig 51. Locus zoom and variant effect forest plot of rs35026344. A) Locus zoom plot of sex-combined EUR stuttering associations, where the sentinel variant is denoted in purple and surrounding variants are color coded by r2 bin using linkage disequilibrium (LD) generated from 1000 Genomes ALL reference. The x axis represents chromosome position (hg37) with annotated genes found within the region, the y axis represents $\log_{10}$ (*p*-value) of the association between the genetic variant and stuttering. Sentinel variant is between *FRAC10AC1* and *LGI1*. B) Variant effect forest plots of rs35026344 found within the genetic ancestries of European male (EUR male), European female (EUR female), African male (AFR male), African female (AFR female), Latino/Admixed American male (AMR male), Latino/Admixed American female (AMR female), East Asian male (EAS male), and East Asian female (EAS female). Male variant effects are designated by triangles, and female variant effects are designed by circles. Line length indicates standard error for the betas found in the

respective GWAS. Variants reaching replicative significance, *p*-value < 8.77 x 10^{-4} (.05/57 unique loci) are indicated by asterisks.

A

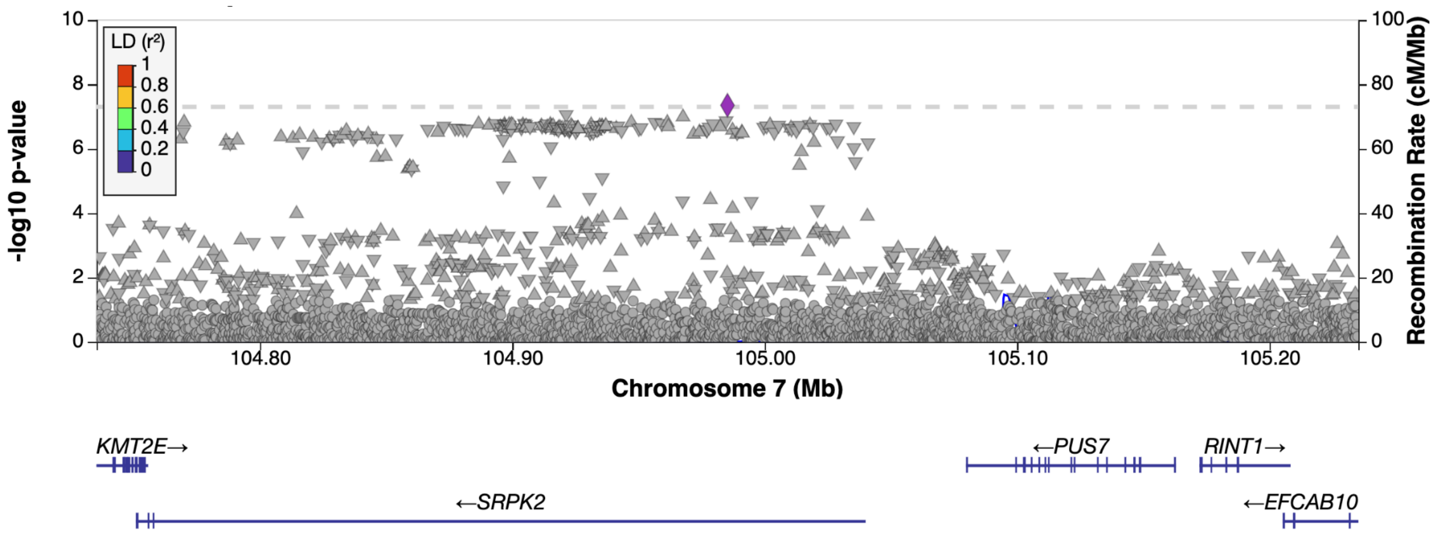

B

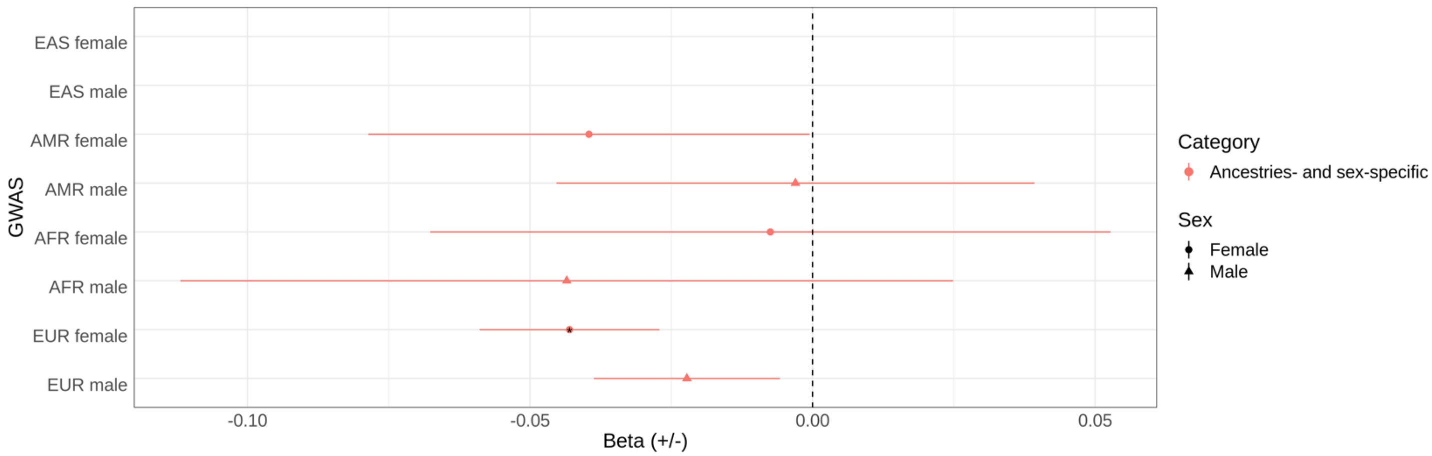

Supplementary Fig 52. Locus zoom and variant effect forest plot of rs112352911. A) Locus zoom plot of sex-combined EUR stuttering associations, where the sentinel variant is denoted in purple and surrounding variants are color coded by r2 bin using linkage disequilibrium (LD) generated from 1000 Genomes ALL reference. The x axis represents chromosome position (hg37) with annotated genes found within the region, the y axis represents $\log_{10}$ (*p*-value) of the association between the genetic variant and stuttering. Sentinel variant is a genetic upstream transcript or intronic variant within *SRPK2*. B) Variant effect forest plots of rs112352911 found within the genetic ancestries of European male (EUR male), European female (EUR female), African male (AFR male), African female (AFR female), Latino/Admixed American male (AMR male), and Latino/Admixed American female (AMR female). Variant not found in East Asian male (EAS male), and East Asian female (EAS female). Male variant effects are designated by triangles, and female variant effects are designed by circles. Line length

indicates standard error for the betas found in the respective GWAS. Variants reaching replicative significance, *p*-value < 8.77 x 10^{-4} (.05/57 unique loci) are indicated by asterisks.

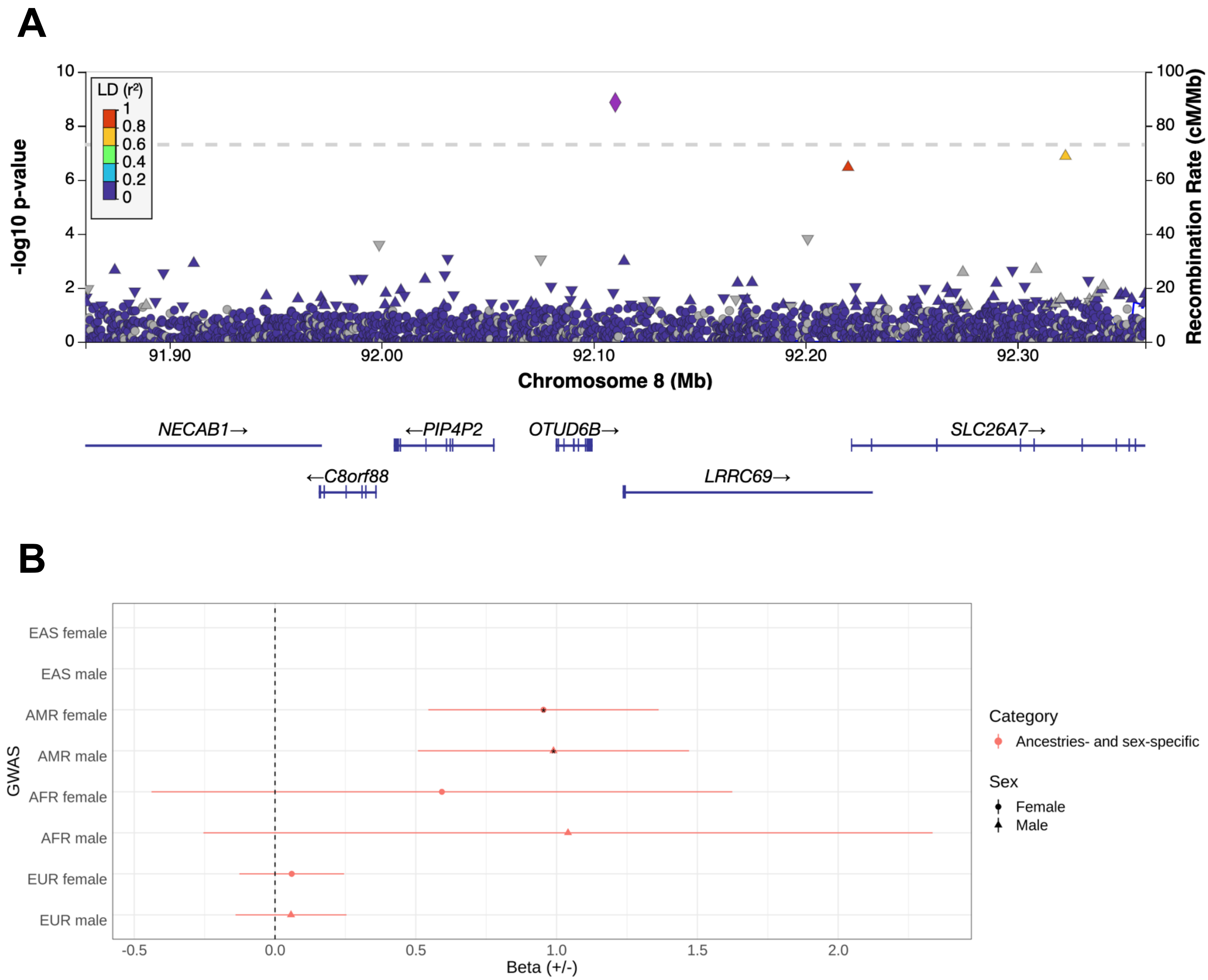

Supplementary Fig 53. Locus zoom and variant effect forest plot of rs141986240. A) Locus zoom plot of sex-combined AMR stuttering associations, where the sentinel variant is denoted in purple and surrounding variants are color coded by r2 bin using linkage disequilibrium (LD) generated from 1000 Genomes ALL reference. The x axis represents chromosome position (hg37) with annotated genes found within the region, the y axis represents $\log_{10}$ (*p*-value) of the association between the genetic variant and stuttering. Sentinel variant is between *OTUD6B* and *LRRC69*. B) Variant effect forest plots of rs141986240 found within the genetic ancestries of European male (EUR male), European female (EUR female), African male (AFR male), African female (AFR female), Latino/Admixed American male (AMR male), and Latino/Admixed American female (AMR female). Variant not found in East Asian male (EAS male), and East Asian female (EAS female). Male variant effects are designated by triangles, and female variant effects are designed by circles. Line length indicates standard error for the betas found in the

respective GWAS. Variants reaching replicative significance, *p*-value < 8.77×10^{-4} (.05/57 unique loci) are indicated by asterisks.

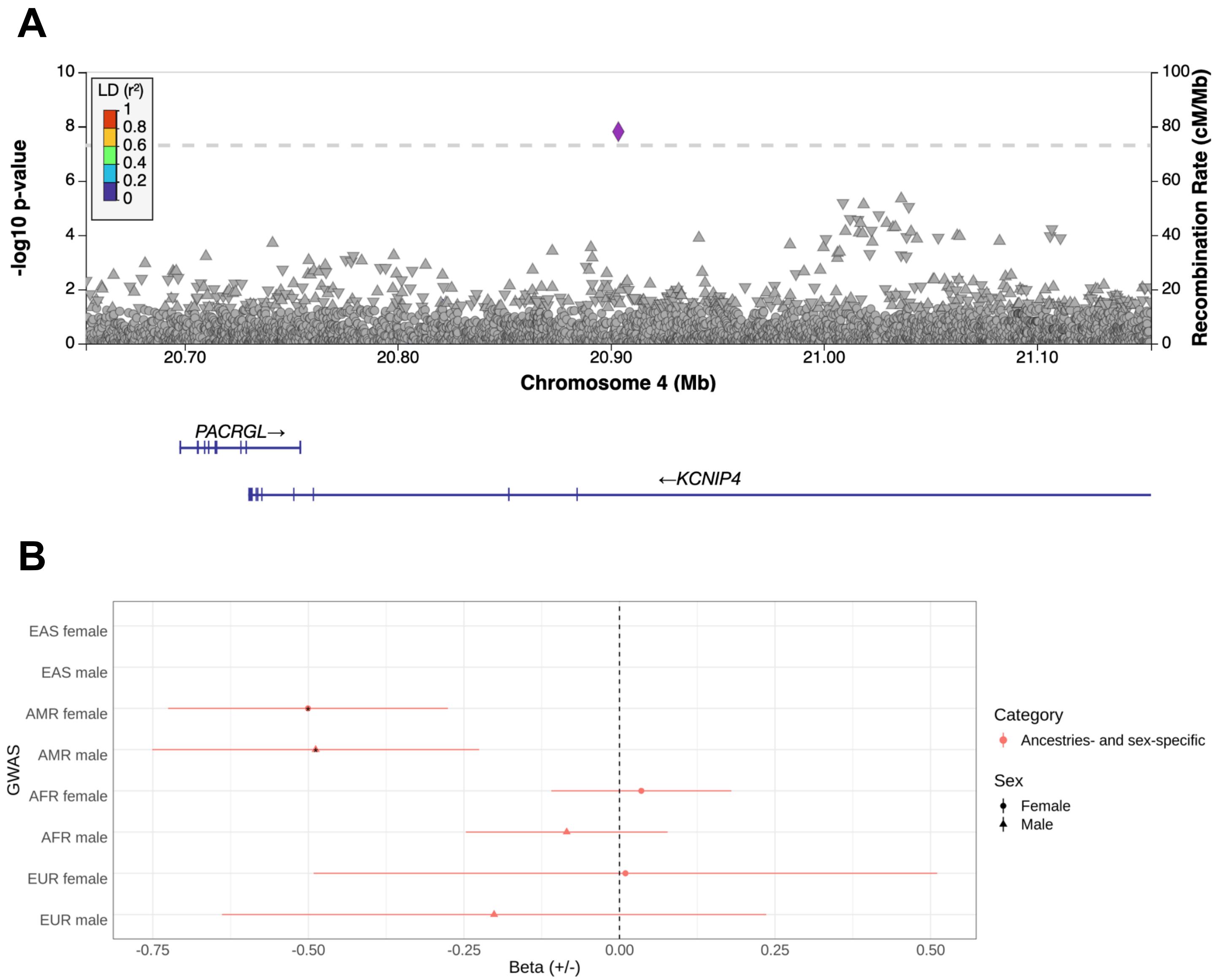

Supplementary Fig 54. Locus zoom and variant effect forest plot of rs146784668. A) Locus zoom plot of sex-combined AMR stuttering associations, where the sentinel variant is denoted in purple and surrounding variants are color coded by r2 bin using linkage disequilibrium (LD) generated from 1000 Genomes ALL reference. The x axis represents chromosome position (hg37) with annotated genes found within the region, the y axis represents $\log_{10}$ (*p*-value) of the association between the genetic variant and stuttering. Sentinel variant is an intronic variant within *KCNIP4*. B) Variant effect forest plots of rs146784668 found within the genetic ancestries of European male (EUR male), European female (EUR female), African male (AFR male), African female (AFR female), Latino/Admixed American male (AMR male), and Latino/Admixed American female (AMR female). Variant not found in East Asian male (EAS male), and East Asian female (EAS female). Male variant effects are designated by triangles, and female variant effects are designed by circles. Line length indicates standard error for the betas

found in the respective GWAS. Variants reaching replicative significance, *p*-value < 8.77×10^{-4} (.05/57 unique loci) are indicated by asterisks.

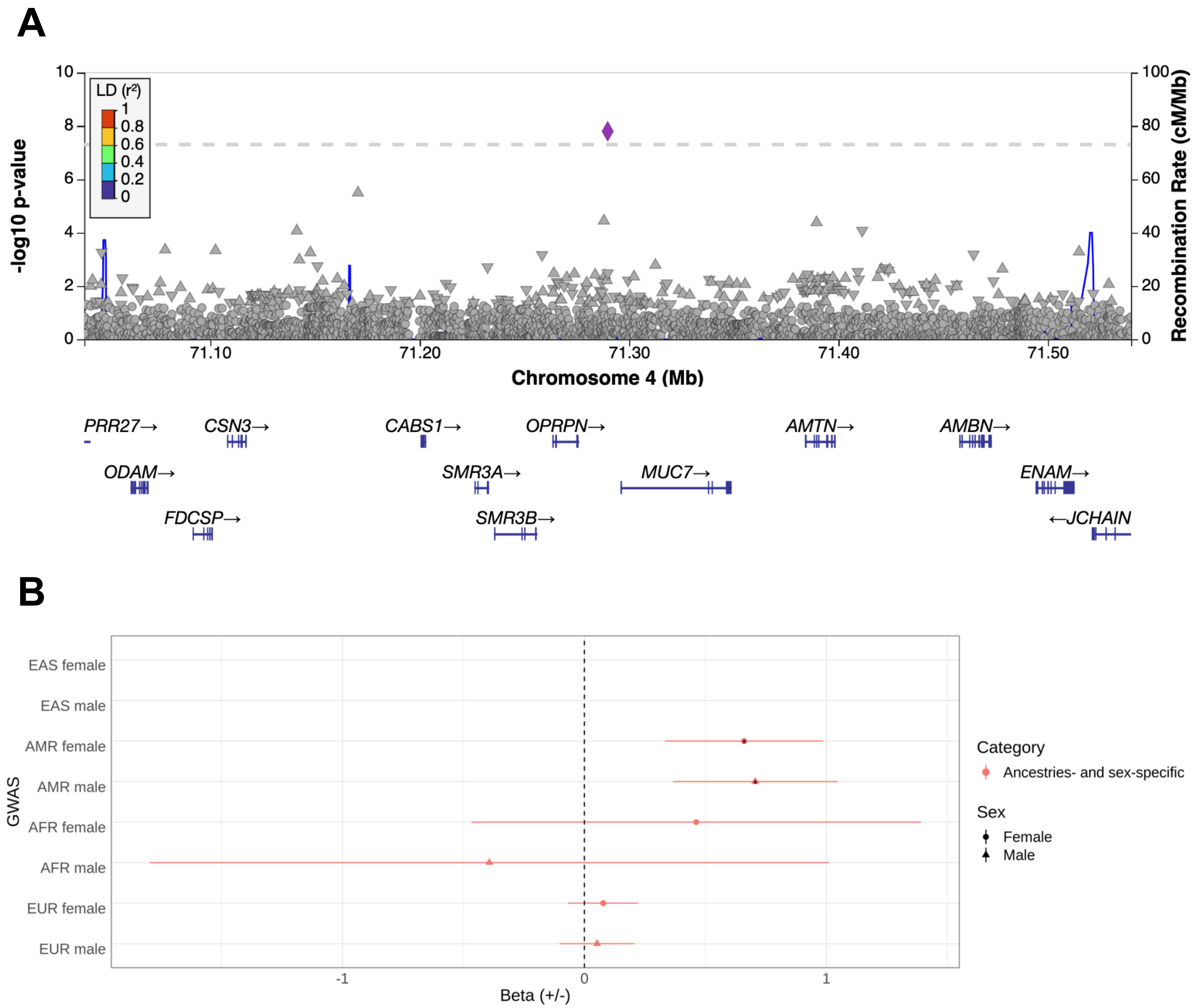

Supplementary Fig 55. Locus zoom and variant effect forest plot of rs559419877:GA. A) Locus zoom plot of sex-combined AMR stuttering associations, where the sentinel variant is denoted in purple and surrounding variants are color coded by r2 bin using linkage disequilibrium (LD) generated from 1000 Genomes ALL reference. The x axis represents chromosome position (hg37) with annotated genes found within the region, the y axis represents $\log_{10}$ (*p*-value) of the association between the genetic variant and stuttering. Sentinel variant is between *OPRPN* and *MUC7*. B) Variant effect forest plots of rs559419877:GA found within the genetic ancestries of European male (EUR male), European female (EUR female), African male (AFR male), African female (AFR female), Latino/Admixed American male (AMR male), and Latino/Admixed American female (AMR female). Variant not found in East Asian male (EAS male), and East Asian female (EAS female). Male variant effects are designated by triangles, and female variant

effects are designed by circles. Line length indicates standard error for the betas found in the respective GWAS. Variants reaching replicative significance, *p*-value < 8.77×10^{-4} (.05/57 unique loci) are indicated by asterisks.

A

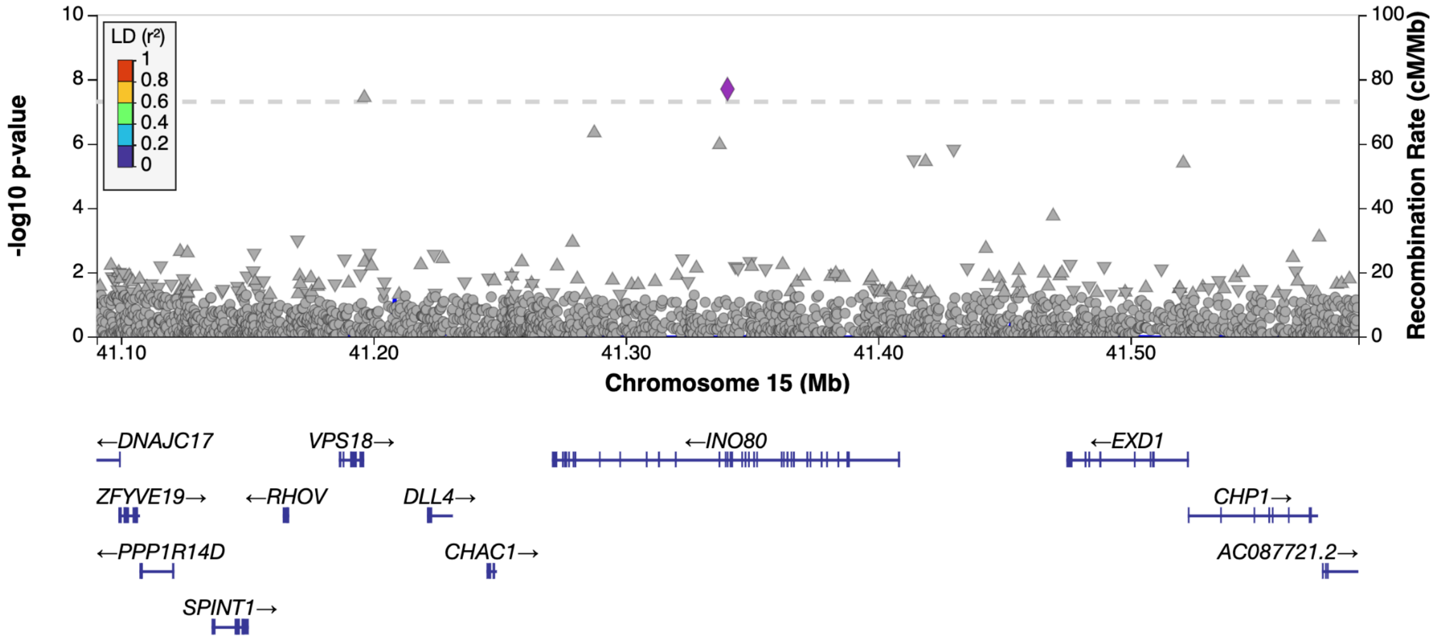

B

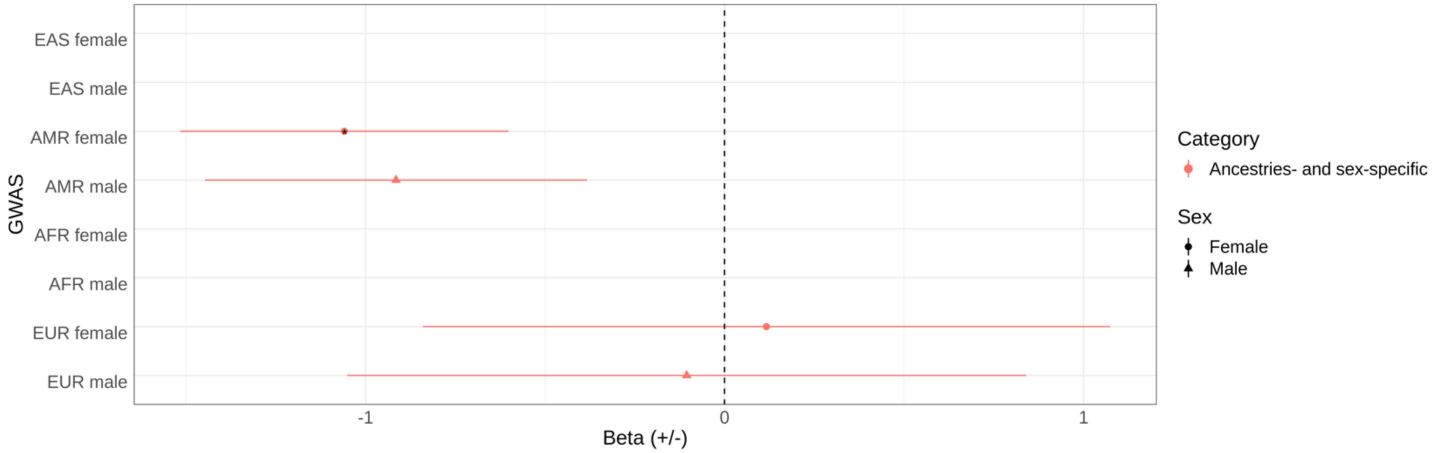

Supplementary Fig 56. Locus zoom and variant effect forest plot of rs188750450. A) Locus zoom plot of sex-combined AMR stuttering associations, where the sentinel variant is denoted in purple and surrounding variants are color coded by r2 bin using linkage disequilibrium (LD) generated from 1000 Genomes ALL reference. The x axis represents chromosome position (hg37) with annotated genes found within the region, the y axis represents $\log_{10}$ (*p*-value) of the association between the genetic variant and stuttering. Sentinel variant is an intronic variant within *INO80*. B) Variant effect forest plots of rs188750450 found within the genetic ancestries of European male (EUR male), European female (EUR female), Latino/Admixed American male (AMR male), and Latino/Admixed American female (AMR female). Variant not found in African male (AFR male), African female (AFR female), East Asian male (EAS male), and

East Asian female (EAS female). Male variant effects are designated by triangles, and female variant effects are designed by circles. Line length indicates standard error for the betas found in the respective GWAS. Variants reaching replicative significance, *p*-value < 8.77×10^{-4} (.05/57 unique loci) are indicated by asterisks.

A

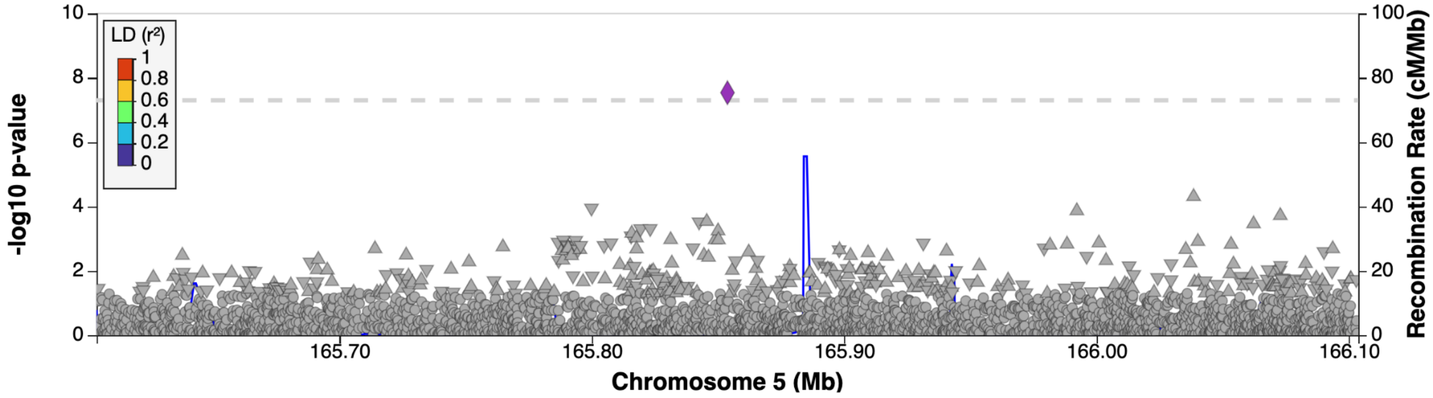

B

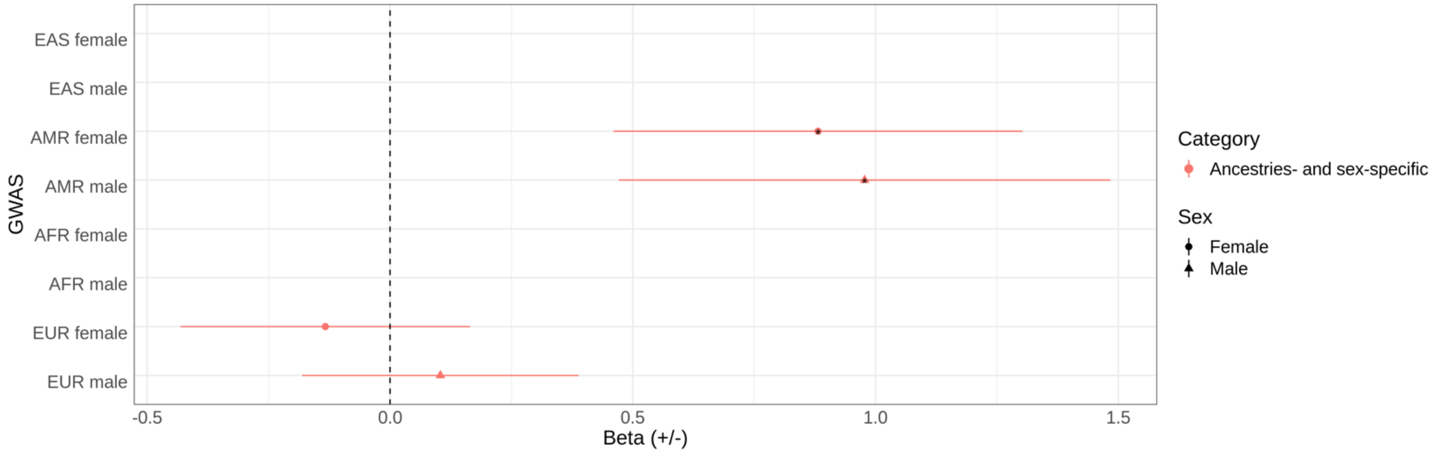

Supplementary Fig 57. Locus zoom and variant effect forest plot of rs537943976. A) Locus zoom plot of sex-combined AMR stuttering associations, where the sentinel variant is denoted in purple and surrounding variants are color coded by r2 bin using linkage disequilibrium (LD) generated from 1000 Genomes ALL reference. The x axis represents chromosome position (hg37) with annotated genes found within the region, the y axis represents $\log_{10}$ (*p*-value) of the association between the genetic variant and stuttering. Sentinel variant is located more than 500kb (upstream or downstream) from a protein-coding gene. B) Variant effect forest plots of rs537943976 found within the genetic ancestries of European male (EUR male), European female (EUR female), Latino/Admixed American male (AMR male), and Latino/Admixed American female (AMR female). Variant not found in African male (AFR male), African female (AFR female), East Asian male (EAS male), and East Asian female (EAS female). Male variant effects are designated by triangles, and female variant effects are designed by circles. Line length indicates standard error for the betas found in the respective GWAS. Variants reaching replicative significance, *p*-value < 8.77×10^{-4} (.05/57 unique loci) are indicated by asterisks.

A

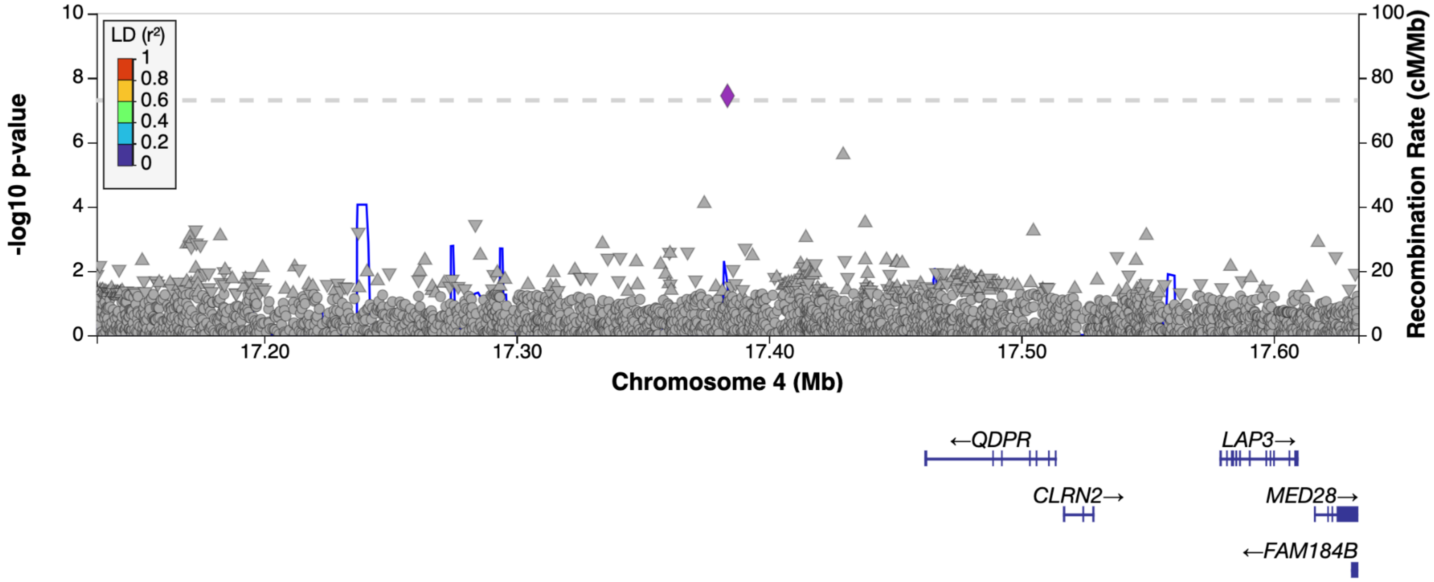

B

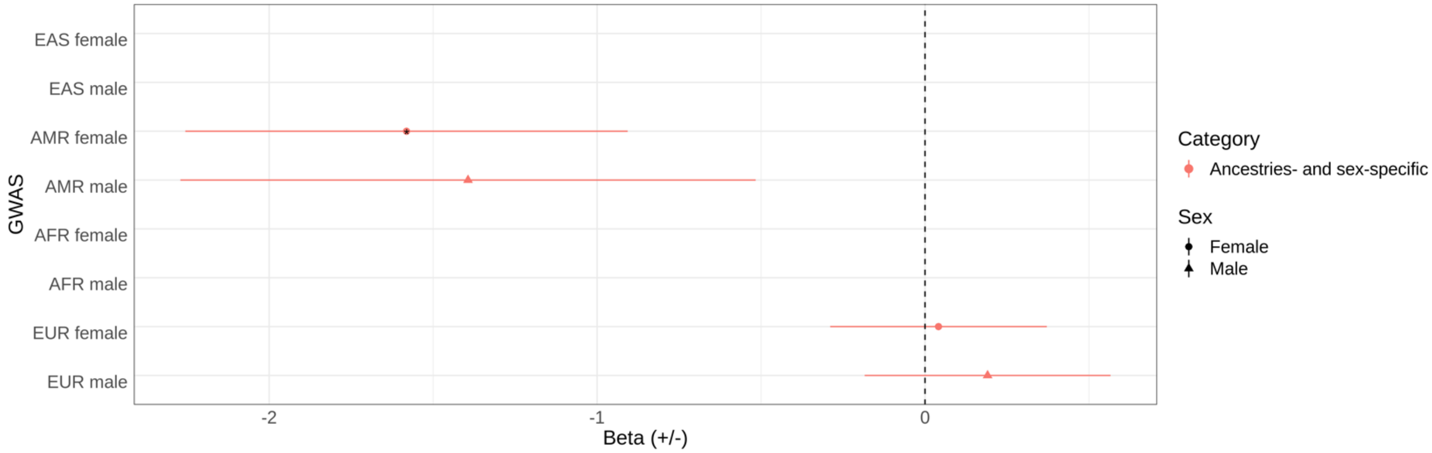

Supplementary Fig 58. Locus zoom and variant effect forest plot of rs578220221. A) Locus zoom plot of sex-combined AMR stuttering associations, where the sentinel variant is denoted in purple and surrounding variants are color coded by r2 bin using linkage disequilibrium (LD) generated from 1000 Genomes ALL reference. The x axis represents chromosome position (hg37) with annotated genes found within the region, the y axis represents $\log_{10}$ (*p*-value) of the association between the genetic variant and stuttering. Sentinel variant is upstream of *QDPR*. B) Variant effect forest plots of rs578220221 found within the genetic ancestries of European male (EUR male), European female (EUR female), Latino/Admixed American male (AMR male), and Latino/Admixed American female (AMR female). Variant not found in African male (AFR male), African female (AFR female), East Asian male (EAS male), and East Asian female (EAS female). Male variant effects are designated by triangles, and female variant

effects are designed by circles. Line length indicates standard error for the betas found in the respective GWAS. Variants reaching replicative significance, p-value < 8.77×10^{-4} (.05/57 unique loci) are indicated by asterisks.

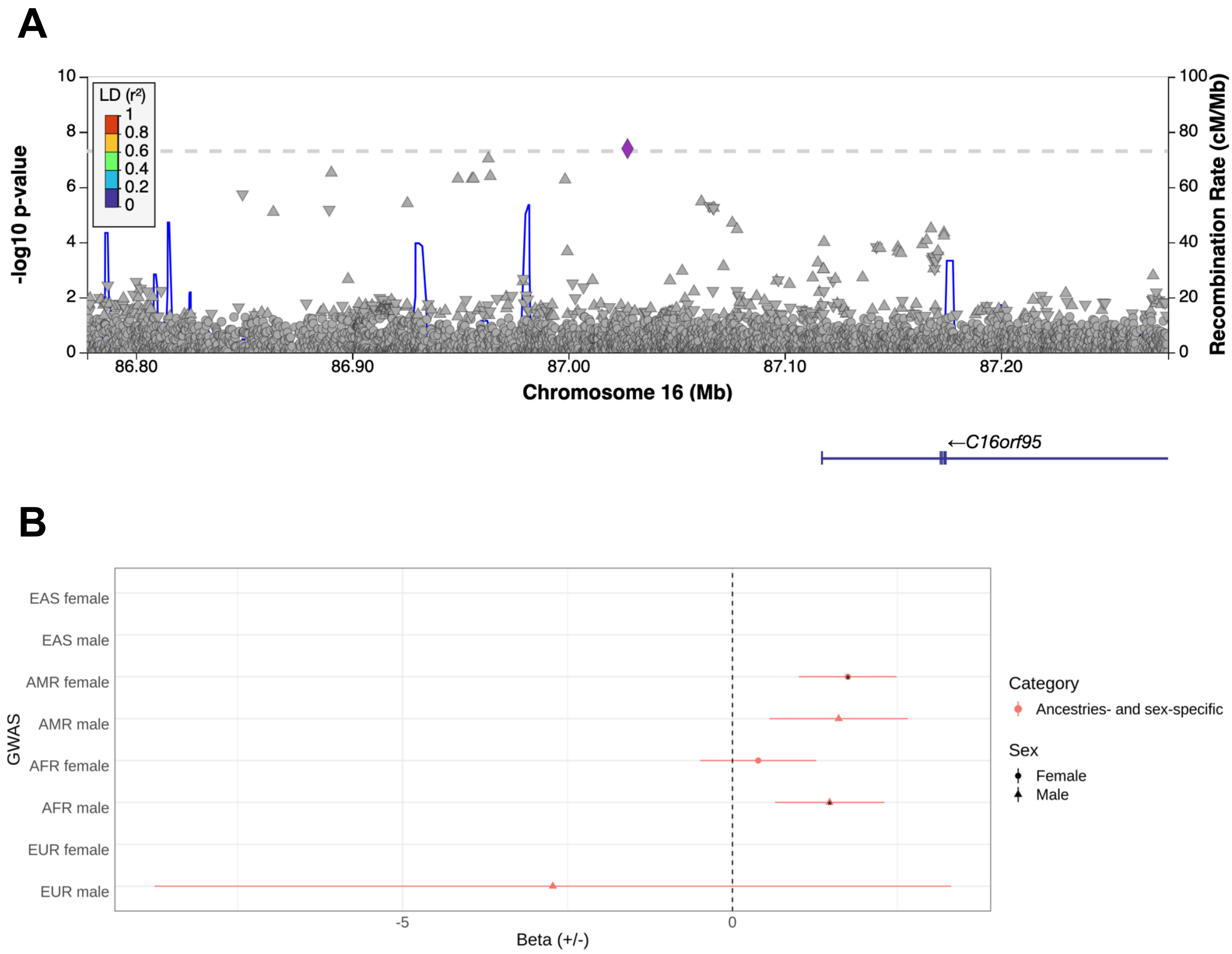

Supplementary Fig 59. Locus zoom and variant effect forest plot of rs556819764. A) Locus zoom plot of sex-combined AMR stuttering associations, where the sentinel variant is denoted in purple and surrounding variants are color coded by r2 bin using linkage disequilibrium (LD) generated from 1000 Genomes ALL reference. The x axis represents chromosome position (hg37) with annotated genes found within the region, the y axis represents $\log_{10}$ (*p*-value) of the association between the genetic variant and stuttering. Sentinel variant is upstream of *C16orf95*. B) Variant effect forest plots of rs556819764 found within the genetic ancestries of European male (EUR male), African male (AFR male), African female (AFR female), Latino/Admixed American male (AMR male), and Latino/Admixed American female (AMR female). Variant not found in European female (EUR female), East Asian male (EAS male), and East Asian female (EAS female). Male variant effects are designated by triangles, and female variant effects are designed by circles. Line length indicates standard error for the betas found in the respective GWAS. Variants reaching replicative significance, *p*-value < 8.77×10^{-4} (.05/57 unique loci) are indicated by asterisks.

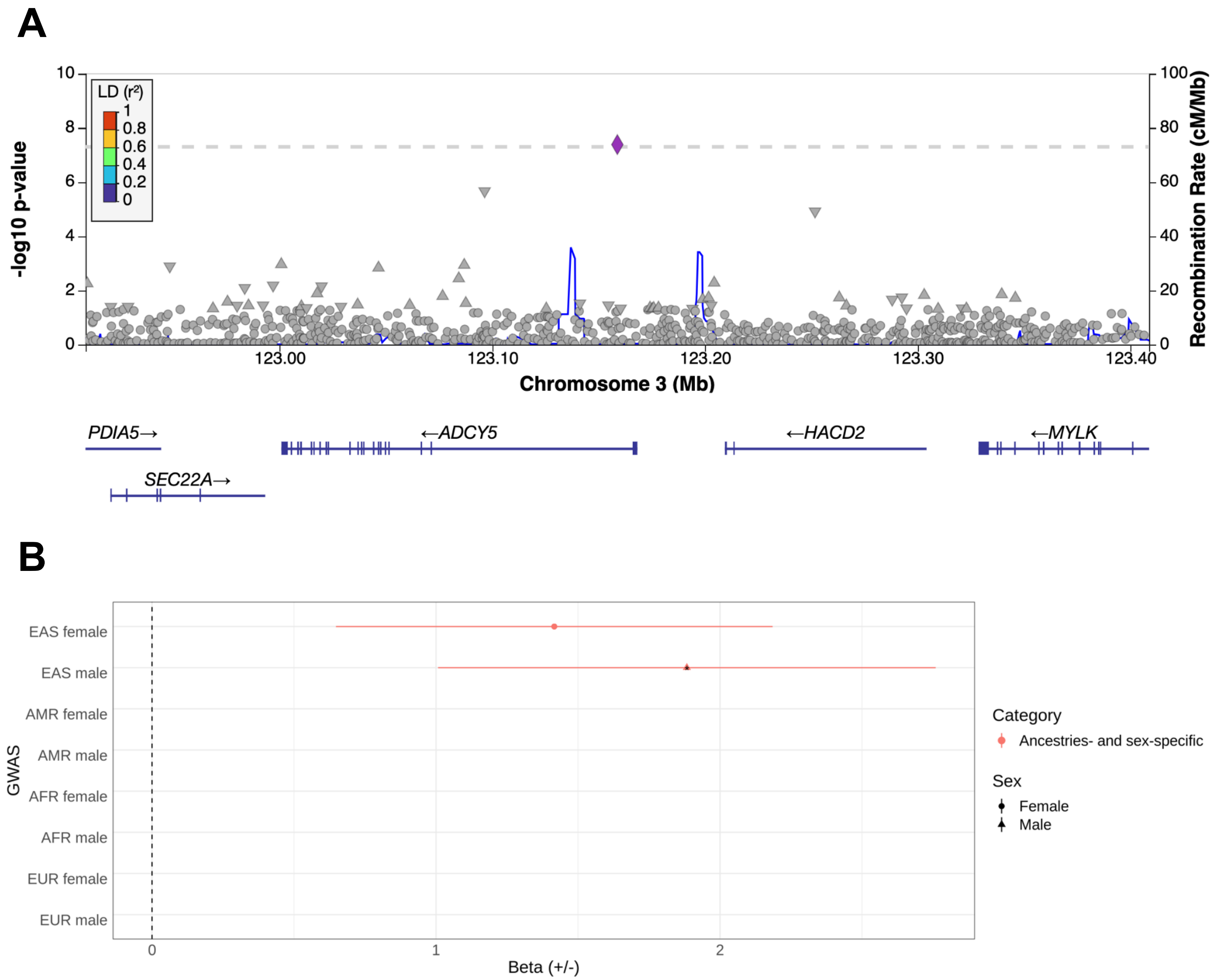

Supplementary Fig 60. Locus zoom and variant effect forest plot of rs140279480. A) Locus zoom plot of sex-combined EAS stuttering associations, where the sentinel variant is denoted in purple and surrounding variants are color coded by r2 bin using linkage disequilibrium (LD) generated from 1000 Genomes ALL reference. The x axis represents chromosome position (hg37) with annotated genes found within the region, the y axis represents $\log_{10}$ (*p*-value) of the association between the genetic variant and stuttering. Sentinel variant is a genetic upstream transcript or intronic variant within *ADCY5*. B) Variant effect forest plots of rs140279480 found within the genetic ancestries of East Asian male (EAS male), and East Asian female (EAS female). Variant not found in European male (EUR male), European female (EUR female), African male (AFR male), African female (AFR female), Latino/Admixed American male (AMR male), and Latino/Admixed American female (AMR female). Male variant effects are designated by triangles, and female variant effects are designed by circles. Line length indicates

standard error for the betas found in the respective GWAS. Variants reaching replicative significance, *p*-value < 8.77×10^{-4} (.05/57 unique loci) are indicated by asterisks.

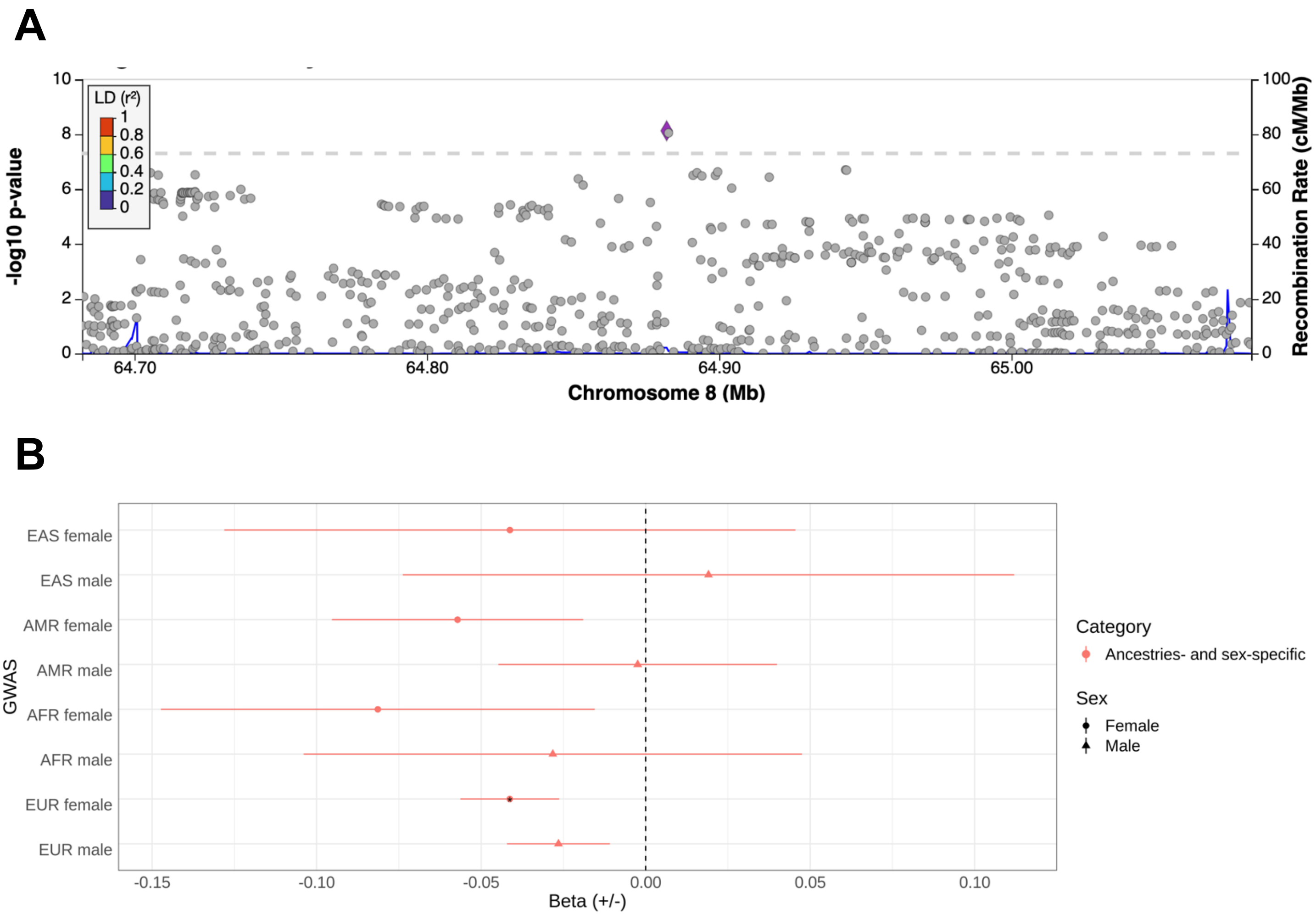

Supplementary Fig 61. Locus zoom and variant effect forest plot of rs10109510. A) Locus zoom plot of trans-ancestries female meta-analysis stuttering associations, where the sentinel variant is denoted in purple and surrounding variants are color coded by r2 bin using linkage disequilibrium (LD) generated from 1000 Genomes ALL reference. The x axis represents chromosome position (hg37) with annotated genes found within the region, the y axis represents log10 (*p*-value) of the association between the genetic variant and stuttering. Sentinel variant is located more than 500kb (upstream or downstream) from a protein-coding gene. B) Variant effect forest plots of rs10109510 found within the genetic ancestries of European male (EUR male), European female (EUR female), African male (AFR male), African female (AFR female), Latino/Admixed American male (AMR male), Latino/Admixed American female (AMR female), East Asian male (EAS male), and East Asian female (EAS female). Male variant effects are designated by triangles, and female variant effects are designed by circles. Line length indicates standard error for the betas found in the respective GWAS. Variants reaching replicative significance, *p*-value < 8.77 x 10^{-4} (.05/57 unique loci) are indicated by asterisks.

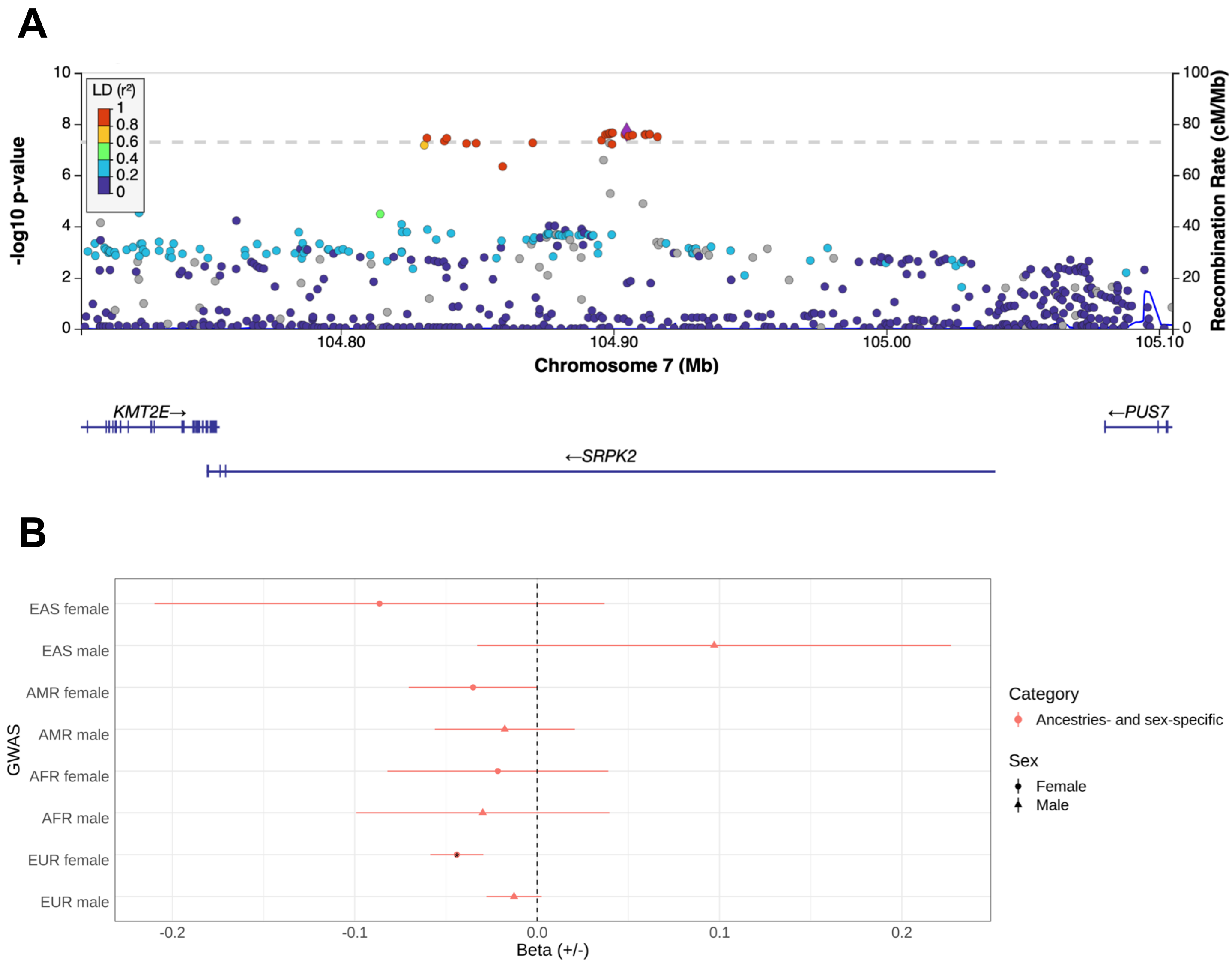

Supplementary Fig 62. Locus zoom and variant effect forest plot of rs3801279. A) Locus zoom plot of trans-ancestries female meta-analysis stuttering associations, where the sentinel variant is denoted in purple and surrounding variants are color coded by r2 bin using linkage disequilibrium (LD) generated from 1000 Genomes ALL reference. The x axis represents chromosome position (hg37) with annotated genes found within the region, the y axis represents $\log_{10}$ (*p*-value) of the association between the genetic variant and stuttering. Sentinel variant is within *SRPK2*. B) Variant effect forest plots of rs3801279 found within the genetic ancestries of European male (EUR male), European female (EUR female), African male (AFR male), African female (AFR female), Latino/Admixed American male (AMR male), Latino/Admixed American female (AMR female), East Asian male (EAS male), and East Asian female (EAS female). Male variant effects are designated by triangles, and female variant effects are designed by circles. Line length indicates standard error for the betas found in the respective GWAS. Variants reaching replicative significance, *p*-value < 8.77 x 10^{-4} (.05/57 unique loci) are indicated by asterisks.

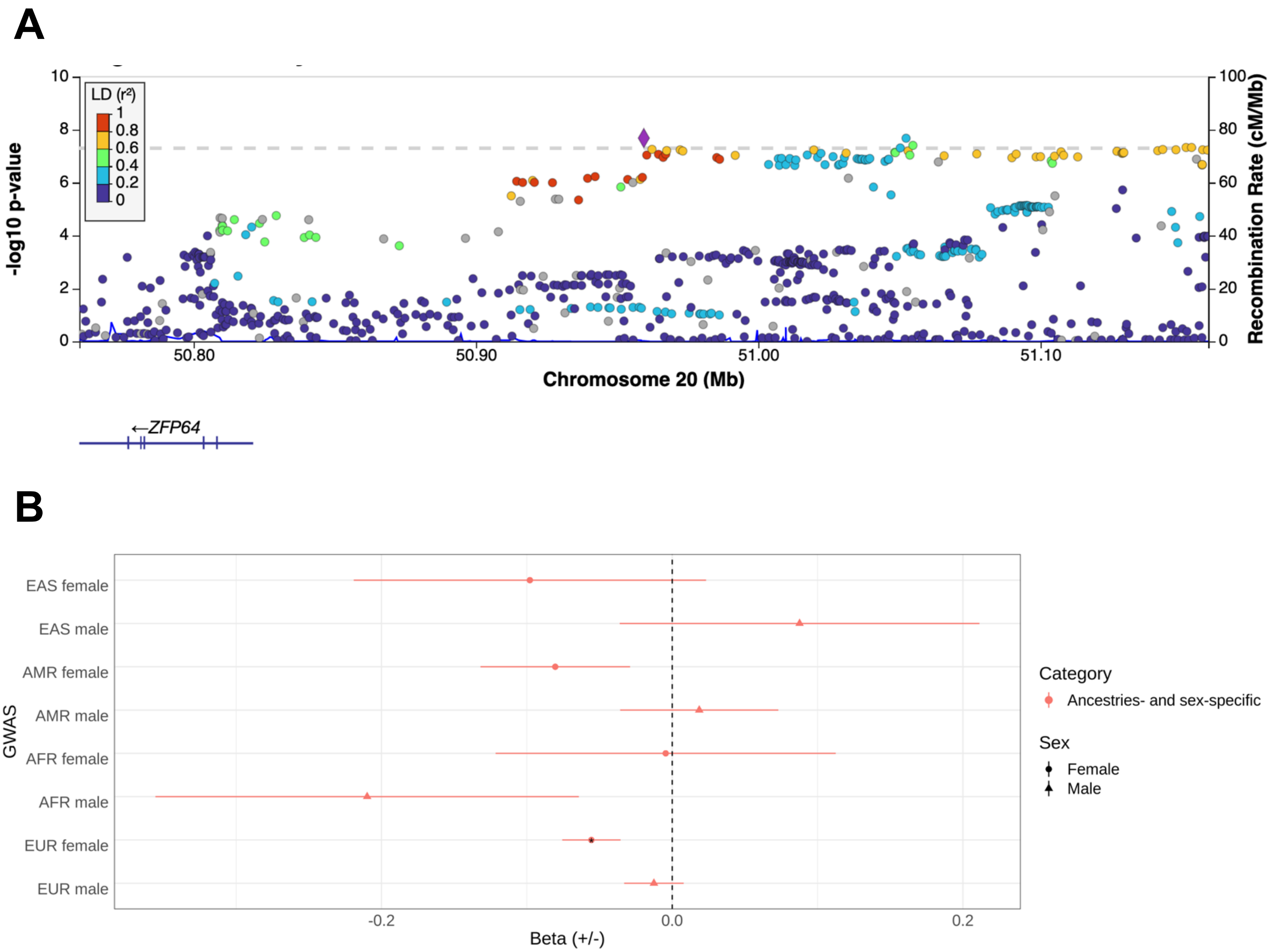

Supplementary Fig 63. Locus zoom and variant effect forest plot of rs13043844. A) Locus zoom plot of trans-ancestries female meta-analysis stuttering associations, where the sentinel variant is denoted in purple and surrounding variants are color coded by r2 bin using linkage disequilibrium (LD) generated from 1000 Genomes ALL reference. The x axis represents chromosome position (hg37) with annotated genes found within the region, the y axis represents $\log_{10}$ (*p*-value) of the association between the genetic variant and stuttering. Sentinel variant is upstream of *ZFP64*. B) Variant effect forest plots of rs13043844 found within the genetic ancestries of European male (EUR male), European female (EUR female), African male (AFR male), African female (AFR female), Latino/Admixed American male (AMR male), Latino/Admixed American female (AMR female), and East Asian male (EAS male), East Asian female (EAS female). Male variant effects are designated by triangles, and female variant effects are designed by circles. Line length indicates standard error for the betas found in the respective GWAS. Variants reaching replicative significance, *p*-value < 8.77×10^{-4} (.05/57 unique loci) are indicated by asterisks.

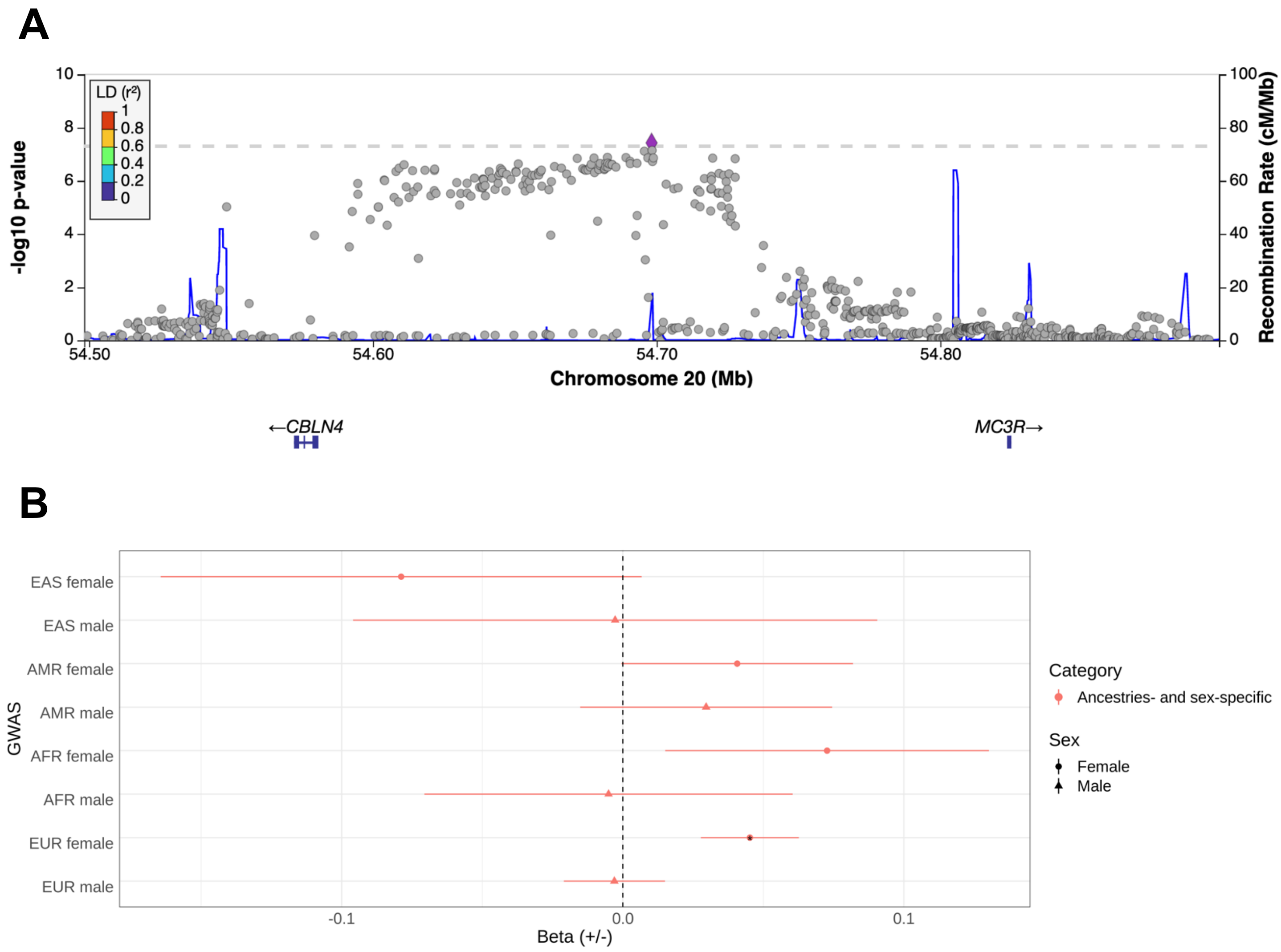

Supplementary Fig 64. Locus zoom and variant effect forest plot of rs36075020. A) Locus zoom plot of trans-ancestries female meta-analysis stuttering associations, where the sentinel variant is denoted in purple and surrounding variants are color coded by r2 bin using linkage disequilibrium (LD) generated from 1000 Genomes ALL reference. The x axis represents chromosome position (hg37) with annotated genes found within the region, the y axis represents $\log_{10}$ (*p*-value) of the association between the genetic variant and stuttering. Sentinel variant is between *CBLN4* and *MC3R*. B) Variant effect forest plots of rs36075020 found within the genetic ancestries of European male (EUR male), European female (EUR female), African male (AFR male), African female (AFR female), Latino/Admixed American male (AMR male), Latino/Admixed American female (AMR female), East Asian male (EAS male), and East Asian female (EAS female). Male variant effects are designated by triangles, and female variant effects are designed by circles. Line length indicates standard error for the betas found in the respective GWAS. Variants reaching replicative significance, *p*-value < 8.77×10^{-4} (.05/57 unique loci) are indicated by asterisks.

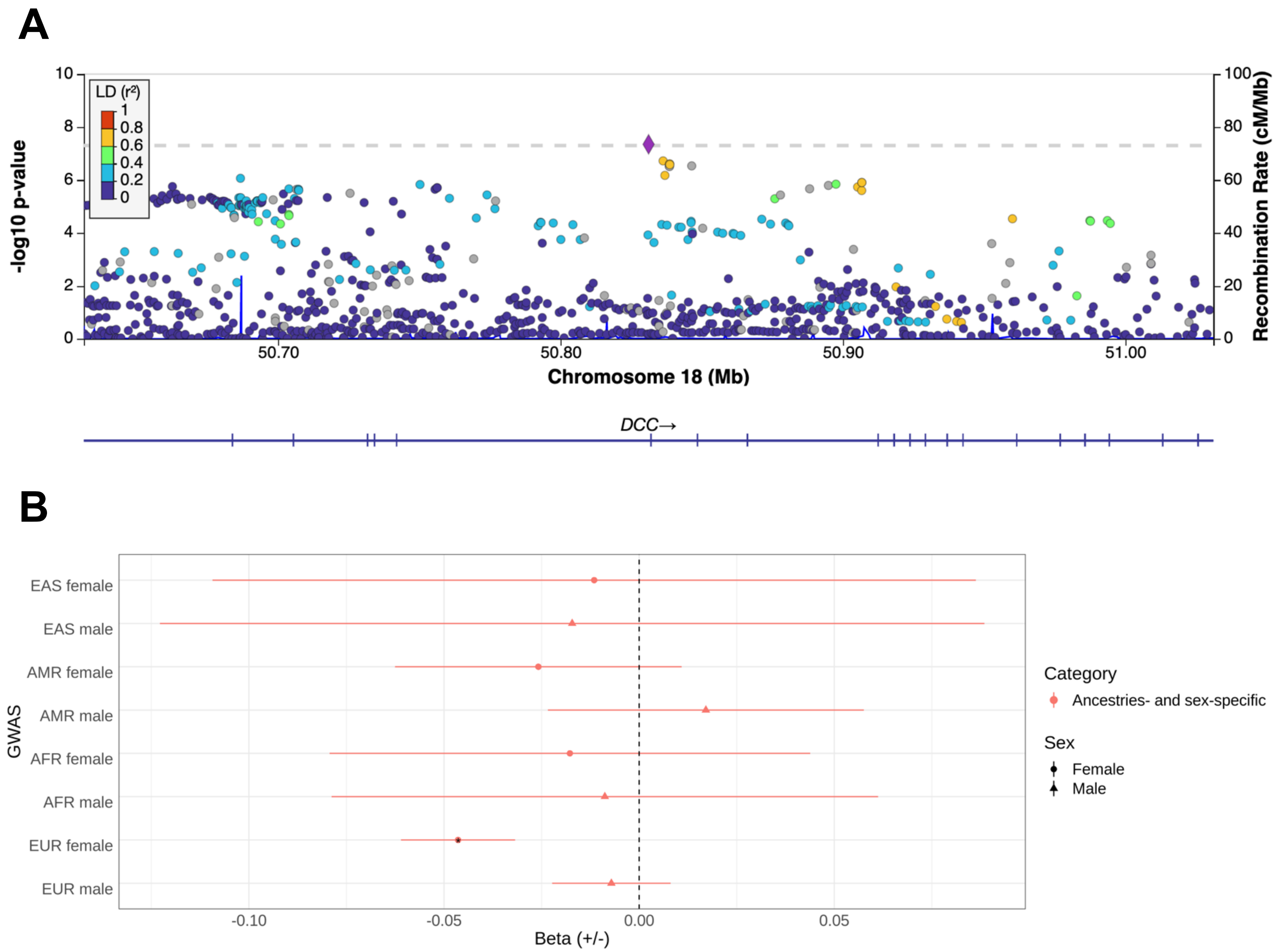

Supplementary Fig 65. Locus zoom and variant effect forest plot of rs6508220. A) Locus zoom plot of trans-ancestries female meta-analysis stuttering associations, where the sentinel variant is denoted in purple and surrounding variants are color coded by r2 bin using linkage disequilibrium (LD) generated from 1000 Genomes ALL reference. The x axis represents chromosome position (hg37) with annotated genes found within the region, the y axis represents $\log_{10}$ (*p*-value) of the association between the genetic variant and stuttering. Sentinel variant is within *DCC*. B) Variant effect forest plots of rs6508220 found within the genetic ancestries of European male (EUR male), European female (EUR female), African male (AFR male), African female (AFR female), Latino/Admixed American male (AMR male), Latino/Admixed American female (AMR female), East Asian male (EAS male), and East Asian female (EAS female). Male variant effects are designated by triangles, and female variant effects are designed by circles. Line length indicates standard error for the betas found in the respective GWAS. Variants reaching replicative significance, *p*-value < 8.77×10^{-4} (.05/57 unique loci) are indicated by asterisks.

A

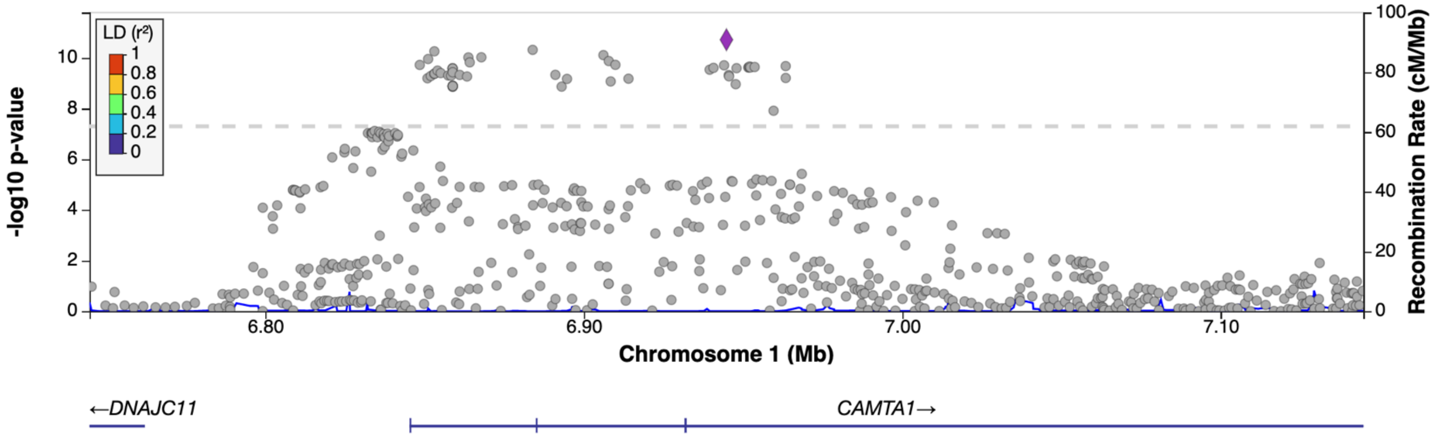

B

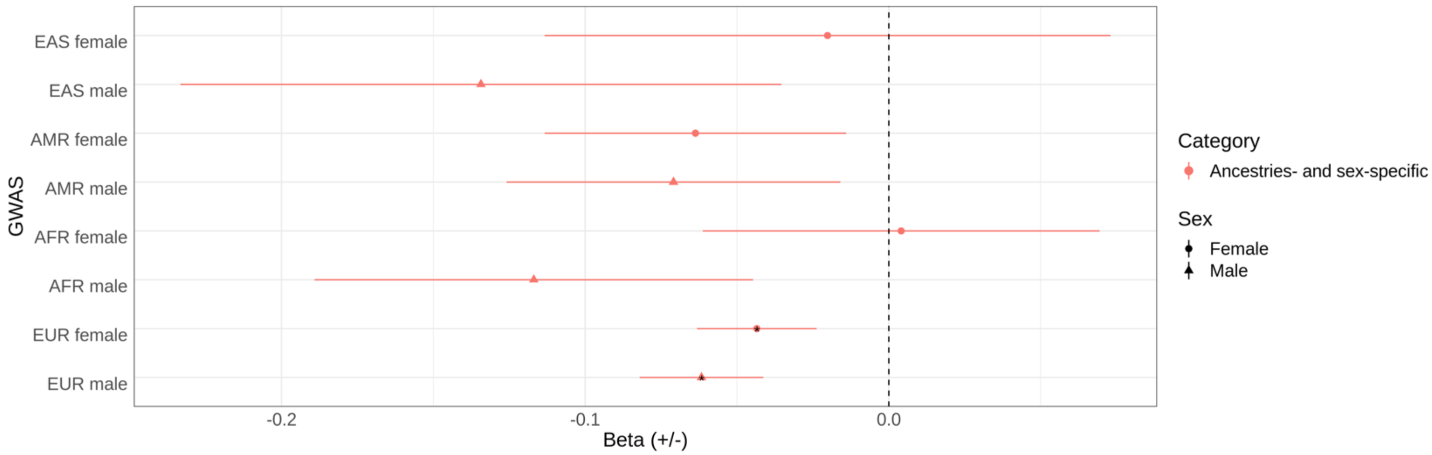

Supplementary Fig 66. Locus zoom and variant effect forest plot of rs950493. A) Locus zoom plot of trans-ancestries male meta-analysis stuttering associations, where the sentinel variant is denoted in purple and surrounding variants are color coded by r2 bin using linkage disequilibrium (LD) generated from 1000 Genomes ALL reference. The x axis represents chromosome position (hg37) with annotated genes found within the region, the y axis represents $\log_{10}$ (*p*-value) of the association between the genetic variant and stuttering. Sentinel variant is within *CAMTA1*. B) Variant effect forest plots of rs950493 found within the genetic ancestries of European male (EUR male), European female (EUR female), African male (AFR male), African female (AFR female), Latino/Admixed American male (AMR male), Latino/Admixed American female (AMR female), East Asian male (EAS male), and East Asian female (EAS female). Male variant effects are designated by triangles, and female variant effects are designed by circles. Line length indicates standard error for the betas found in the respective GWAS. Variants reaching replicative significance, *p*-value < 8.77×10^{-4} (.05/57 unique loci) are indicated by asterisks.

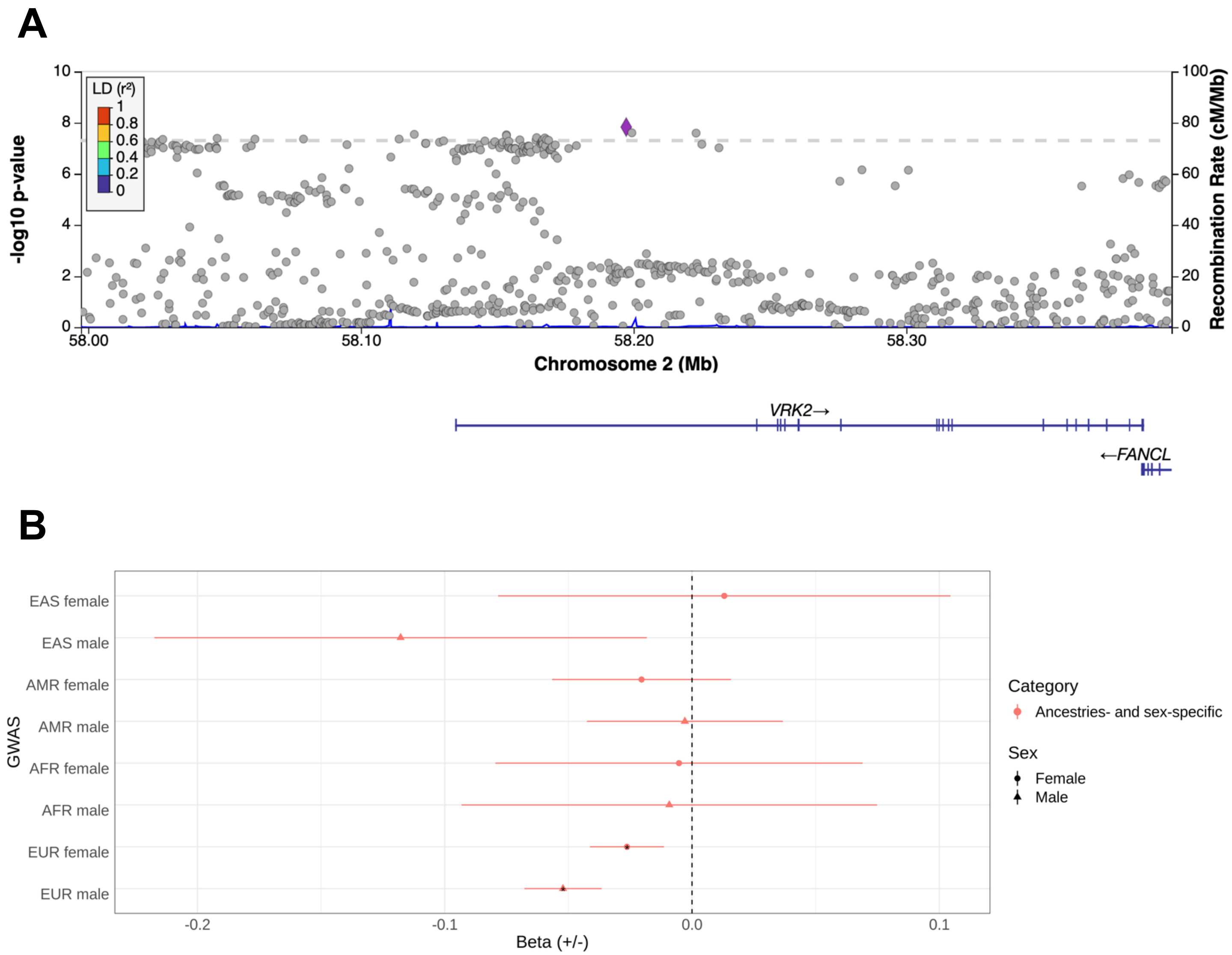

Supplementary Fig 67. Locus zoom and variant effect forest plot of rs966721. A) Locus zoom plot of trans-ancestries male meta-analysis stuttering associations, where the sentinel variant is denoted in purple and surrounding variants are color coded by r2 bin using linkage disequilibrium (LD) generated from 1000 Genomes ALL reference. The x axis represents chromosome position (hg37) with annotated genes found within the region, the y axis represents $\log_{10}$ (*p*-value) of the association between the genetic variant and stuttering. Sentinel variant is within *VRK2*. B) Variant effect forest plots of rs966721 found within the genetic ancestries of European male (EUR male), European female (EUR female), African male (AFR male), African female (AFR female), Latino/Admixed American male (AMR male), Latino/Admixed American female (AMR female), East Asian male (EAS male), and East Asian female (EAS female). Male variant effects are designated by triangles, and female variant effects are designed by circles. Line length indicates standard error for the betas found in the respective GWAS. Variants reaching replicative significance, *p*-value < 8.77×10^{-4} (.05/57 unique loci) are indicated by asterisks.

A

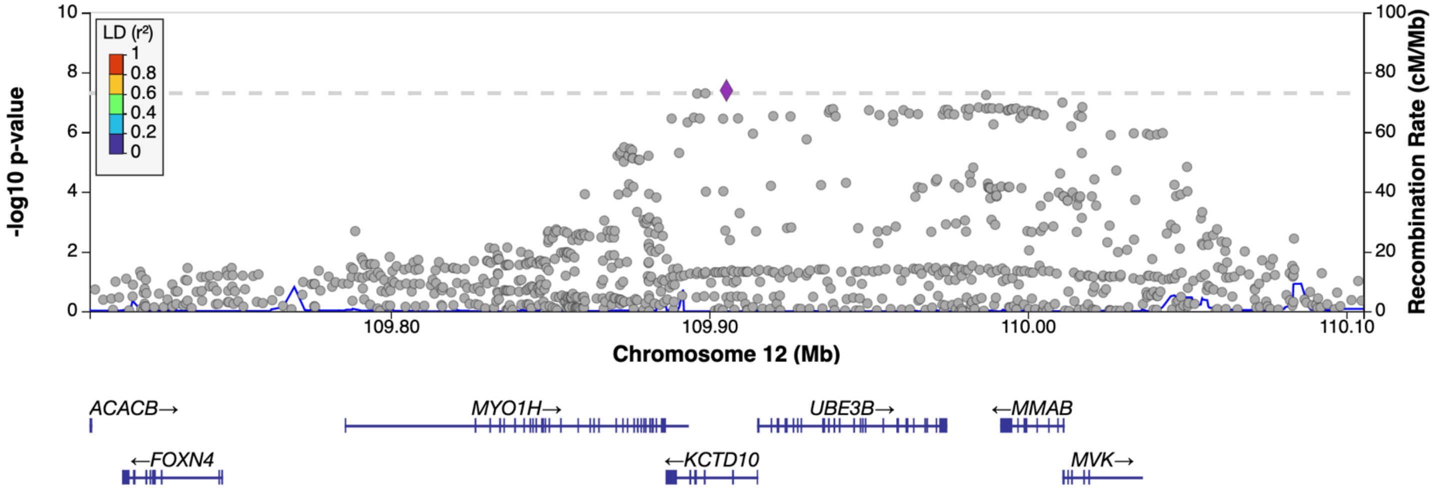

B

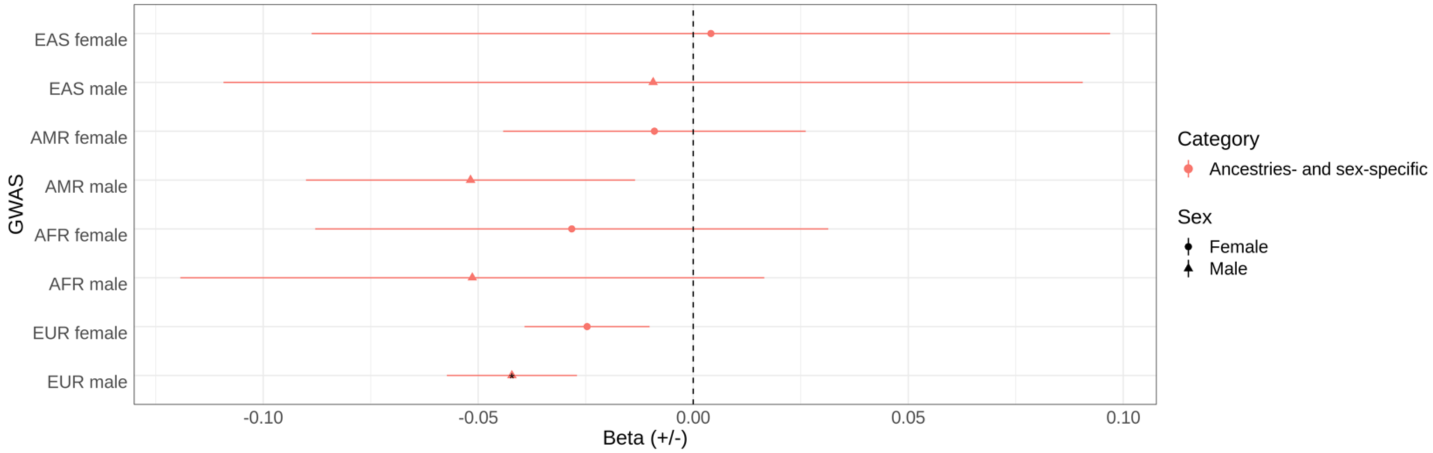

Supplementary Fig 68. Locus zoom and variant effect forest plot of rs6606725. A) Locus zoom plot of trans-ancestries male meta-analysis stuttering associations, where the sentinel variant is denoted in purple and surrounding variants are color coded by r2 bin using linkage disequilibrium (LD) generated from 1000 Genomes ALL reference. The x axis represents chromosome position (hg37) with annotated genes found within the region, the y axis represents $\log_{10}$ (*p*-value) of the association between the genetic variant and stuttering. Sentinel variant is within *KCTD10*. B) Variant effect forest plots of rs6606725 found within the genetic ancestries of European male (EUR male), European female (EUR female), African male (AFR male), African female (AFR female), Latino/Admixed American male (AMR male), Latino/Admixed American female (AMR female), East Asian male (EAS male), and East Asian female (EAS female). Male variant effects are designated by triangles, and female variant effects are designed by circles. Line length indicates standard error for the betas found in the respective GWAS. Variants reaching replicative significance, *p*-value < 8.77×10^{-4} (.05/57 unique loci) are indicated by asterisks.

A

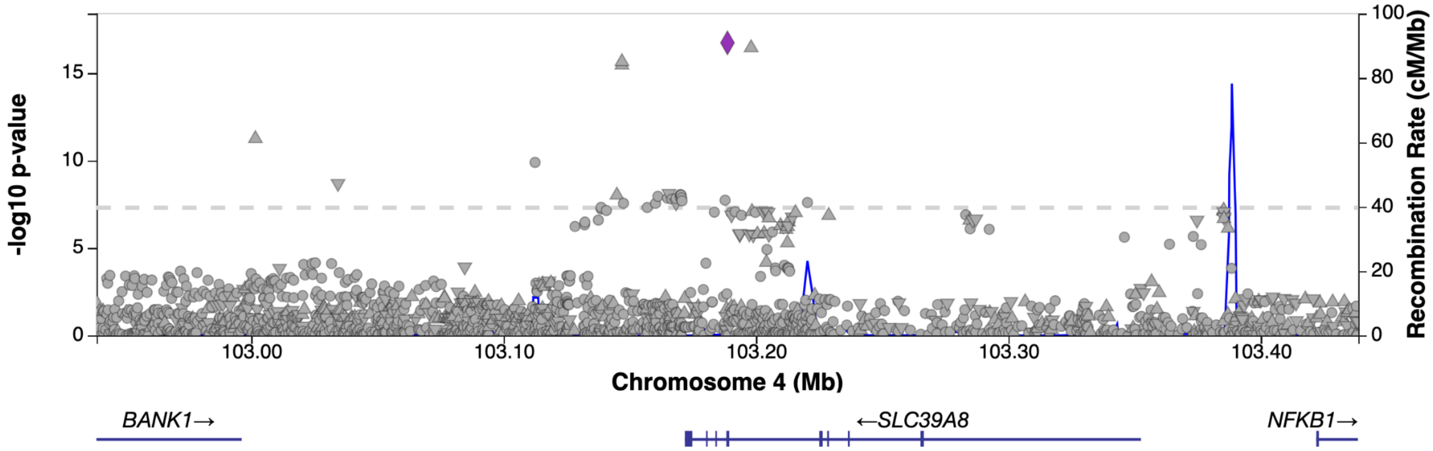

B

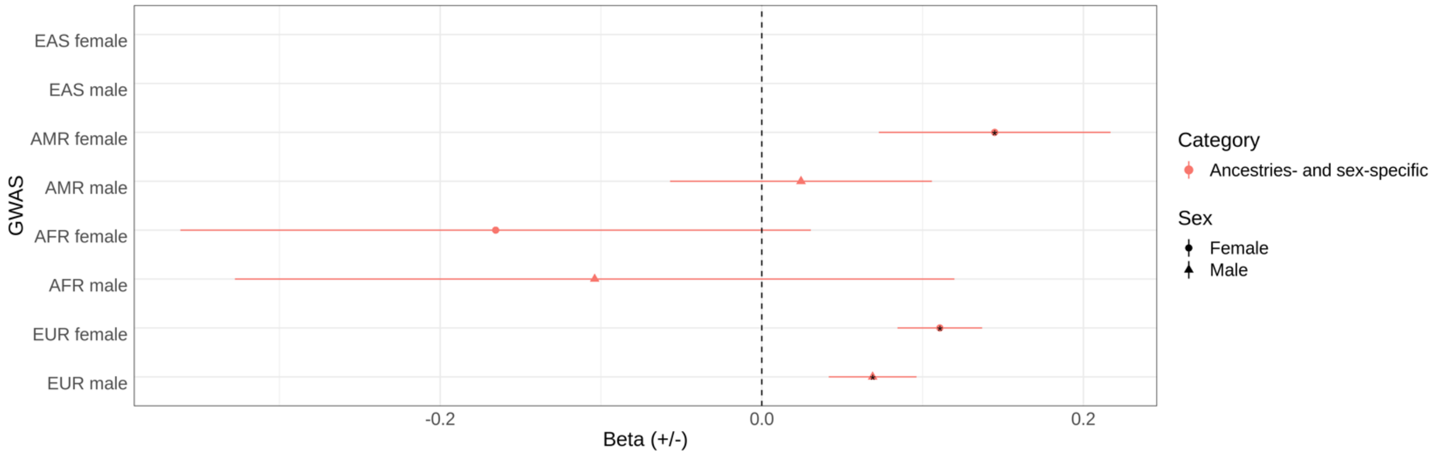

Supplementary Fig 69. Locus zoom and variant effect forest plot of rs13107325. A) Locus zoom plot of trans-ancestries sex-combined meta-analysis stuttering associations, where the sentinel variant is denoted in purple and surrounding variants are color coded by r2 bin using linkage disequilibrium (LD) generated from 1000 Genomes ALL reference. The x axis represents chromosome position (hg37) with annotated genes found within the region, the y axis represents $\log_{10}$ (*p*-value) of the association between the genetic variant and stuttering. Sentinel variant is a missense variant within *SLC39A8*. B) Variant effect forest plots of rs13107325 found within the genetic ancestries of European male (EUR male), European female (EUR female), African male (AFR male), African female (AFR female), Latino/Admixed American male (AMR male), and Latino/Admixed American female (AMR female). Variant not found in East Asian male (EAS male), and East Asian female (EAS female). Male variant effects are designated by triangles, and female variant effects are designed by circles. Line length indicates standard error for the betas found in the respective GWAS. Variants reaching replicative significance, *p*-value < 8.77×10^{-4} (.05/57 unique loci) are indicated by asterisks.

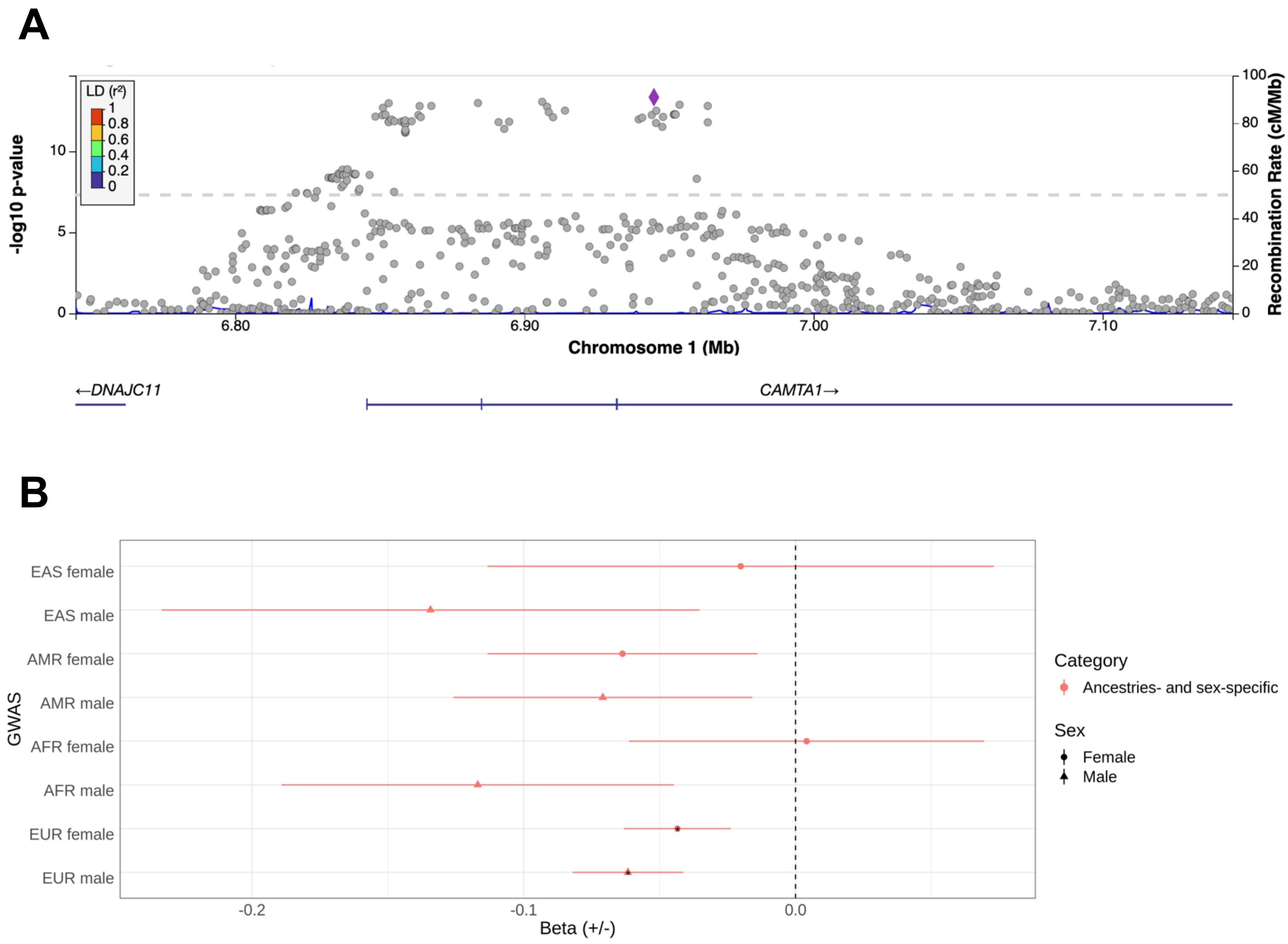

Supplementary Fig 70. Locus zoom and variant effect forest plot of rs950493. A) Locus zoom plot of trans-ancestries sex-combined meta-analysis stuttering associations, where the sentinel variant is denoted in purple and surrounding variants are color coded by r2 bin using linkage disequilibrium (LD) generated from 1000 Genomes ALL reference. The x axis represents chromosome position (hg37) with annotated genes found within the region, the y axis represents $\log_{10}$ (*p*-value) of the association between the genetic variant and stuttering. Sentinel variant is within *CAMTA1*. B) Variant effect forest plots of rs950493 found within the genetic ancestries of European male (EUR male), European female (EUR female), African male (AFR male), African female (AFR female), Latino/Admixed American male (AMR male), Latino/Admixed American female (AMR female), East Asian male (EAS male), and East Asian female (EAS female). Male variant effects are designated by triangles, and female variant effects are designed by circles. Line length indicates standard error for the betas found in the respective GWAS. Variants reaching replicative significance, *p*-value < 8.77×10^{-4} (.05/57 unique loci) are indicated by asterisks.

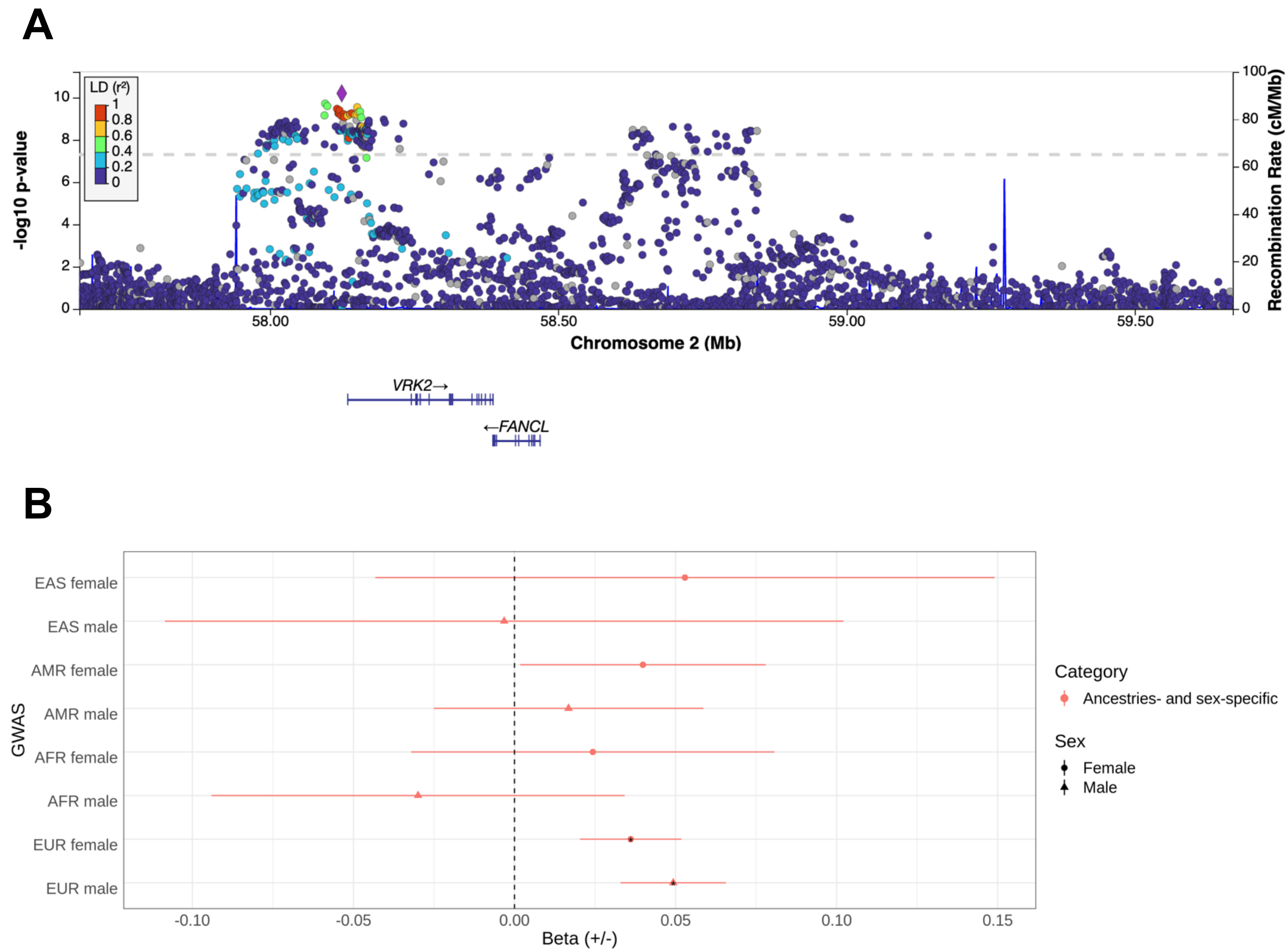

Supplementary Fig 71. Locus zoom and variant effect forest plot of rs13392894. A) Locus zoom plot of trans-ancestries sex-combined meta-analysis stuttering associations, where the sentinel variant is denoted in purple and surrounding variants are color coded by r2 bin using linkage disequilibrium (LD) generated from 1000 Genomes ALL reference. The x axis represents chromosome position (hg37) with annotated genes found within the region, the y axis represents $\log_{10}$ (*p*-value) of the association between the genetic variant and stuttering. Sentinel variant is upstream of *VRK2*. B) Variant effect forest plots of rs13392894 found within the genetic ancestries of European male (EUR male), European female (EUR female), African male (AFR male), African female (AFR female), Latino/Admixed American male (AMR male), Latino/Admixed American female (AMR female), East Asian male (EAS male), and East Asian female (EAS female). Male variant effects are designated by triangles, and female variant effects are designed by circles. Line length indicates standard error for the betas found in the respective GWAS. Variants reaching replicative significance, *p*-value < 8.77×10^{-4} (.05/57 unique loci) are indicated by asterisks.

A

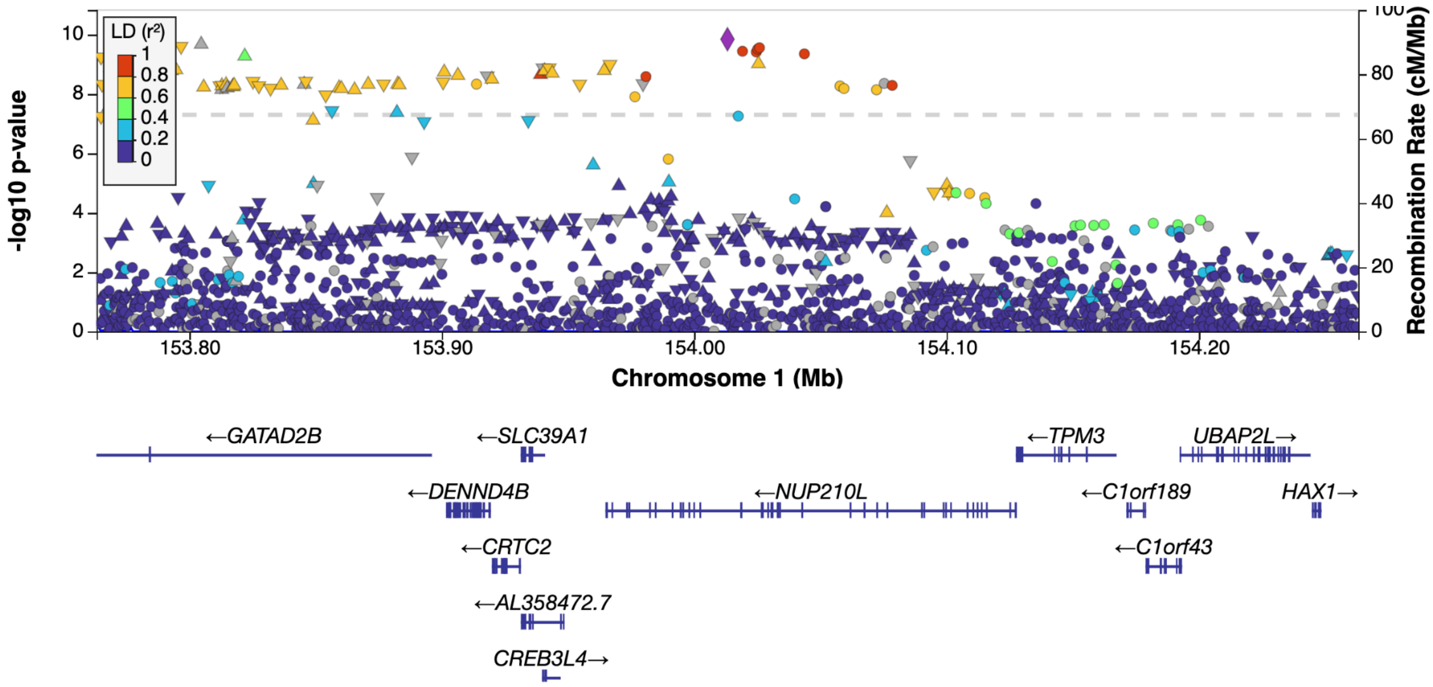

B

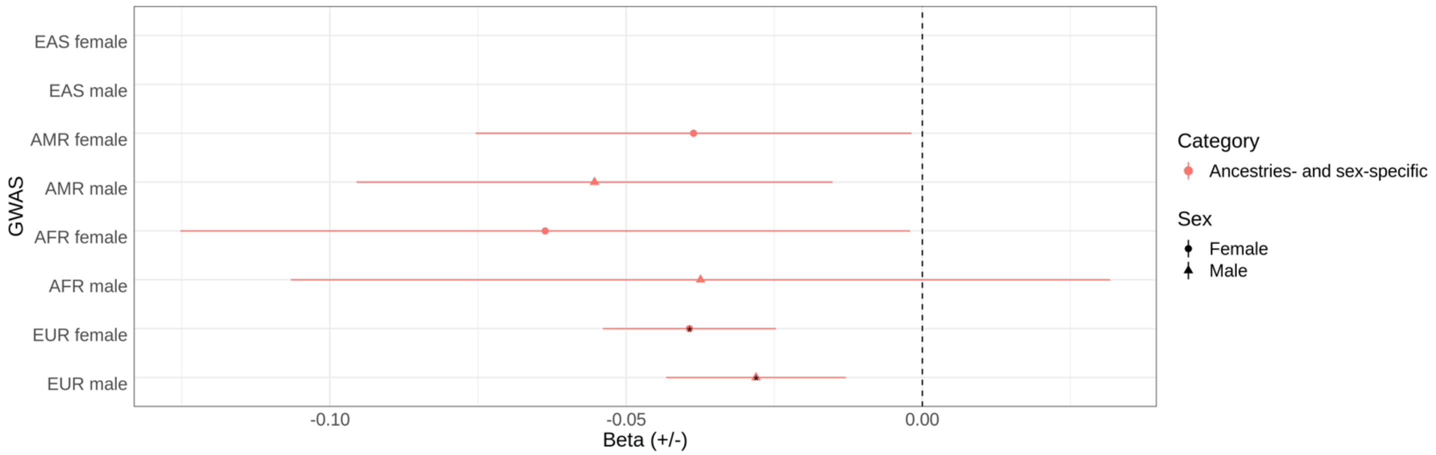

Supplementary Fig 72. Locus zoom and variant effect forest plot of rs2841108. A) Locus zoom plot of trans-ancestries sex-combined meta-analysis stuttering associations, where the sentinel variant is denoted in purple and surrounding variants are color coded by r2 bin using linkage disequilibrium (LD) generated from 1000 Genomes ALL reference. The x axis represents chromosome position (hg37) with annotated genes found within the region, the y axis represents $\log_{10}$ (*p*-value) of the association between the genetic variant and stuttering. Sentinel variant is an intronic variant within *NUP210L*. B) Variant effect forest plots of rs2841108 found within the genetic ancestries of European male (EUR male), European female (EUR female), African male (AFR male), African female (AFR female), Latino/Admixed American

male (AMR male), and Latino/Admixed American female (AMR female). Variant not found in East Asian male (EAS male), and East Asian female (EAS female). Male variant effects are designated by triangles, and female variant effects are designed by circles. Line length indicates standard error for the betas found in the respective GWAS. Variants reaching replicative significance, *p*-value $< 8.77 \times 10^{-4}$ (.05/57 unique loci) are indicated by asterisks.

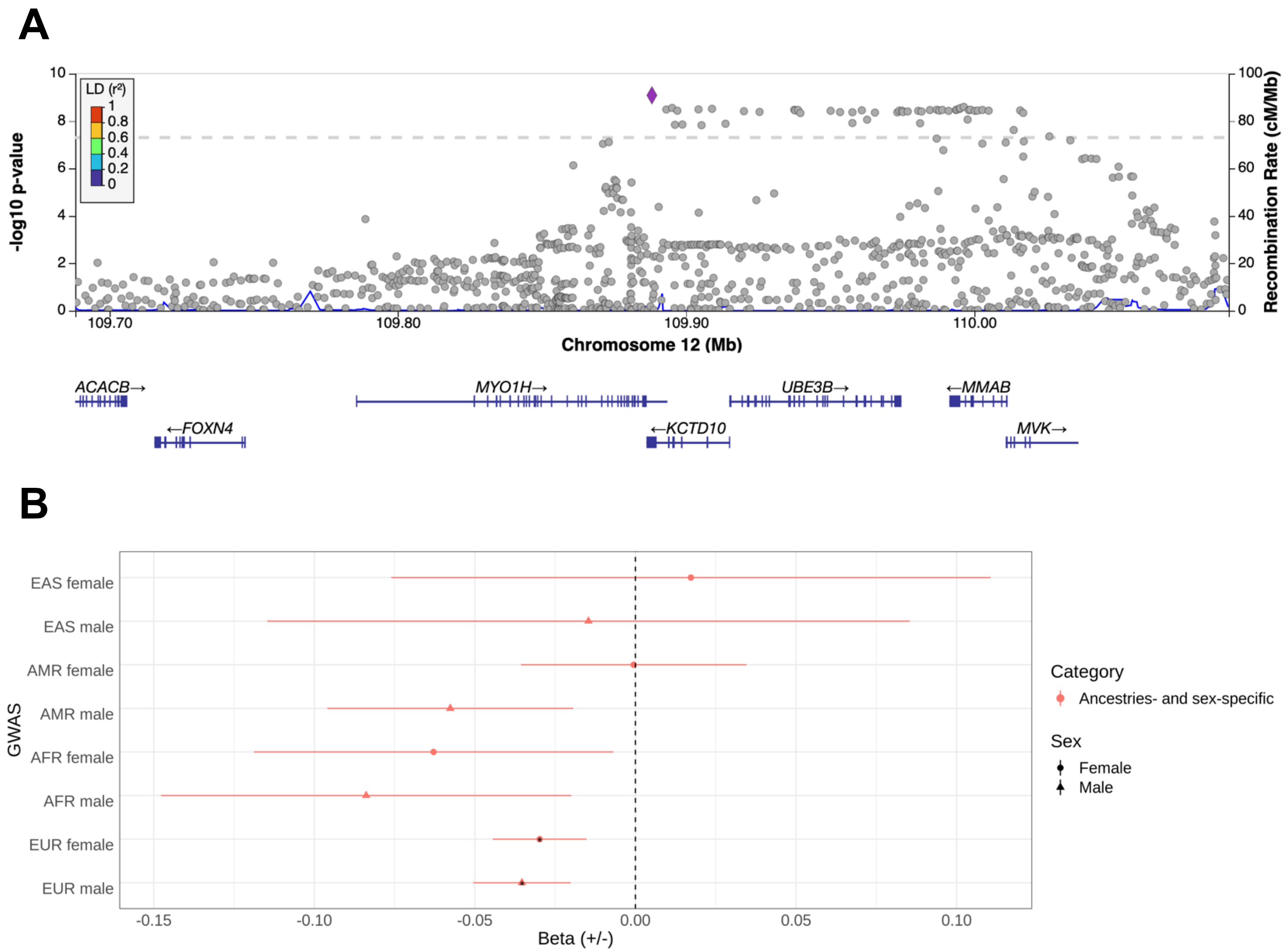

Supplementary Fig 73. Locus zoom and variant effect forest plot of rs3217162. A) Locus zoom plot of trans-ancestries sex-combined meta-analysis stuttering associations, where the sentinel variant is denoted in purple and surrounding variants are color coded by r2 bin using linkage disequilibrium (LD) generated from 1000 Genomes ALL reference. The x axis represents chromosome position (hg37) with annotated genes found within the region, the y axis represents $\log_{10}$ (p-value) of the association between the genetic variant and stuttering. Sentinel variant is a non-coding transcript within *KCTD10*. B) Variant effect forest plots of rs3217162 found within the genetic ancestries of European male (EUR male), European female (EUR female), African male (AFR male), African female (AFR female), Latino/Admixed American male (AMR male), Latino/Admixed American female (AMR female), East Asian male (EAS male), and East Asian female (EAS female). Male variant effects are designated by triangles, and female variant effects are designed by circles. Line length indicates standard error for the betas found in the respective GWAS. Variants reaching replicative significance, *p*-value < 8.77×10^{-4} (.05/57 unique loci) are indicated by asterisks.

A

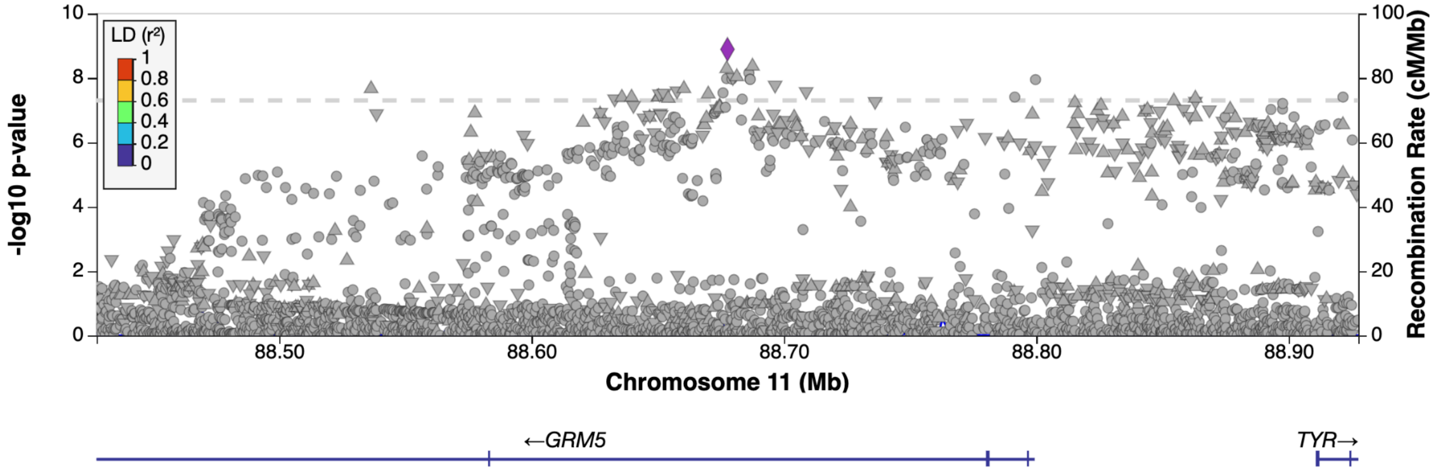

B

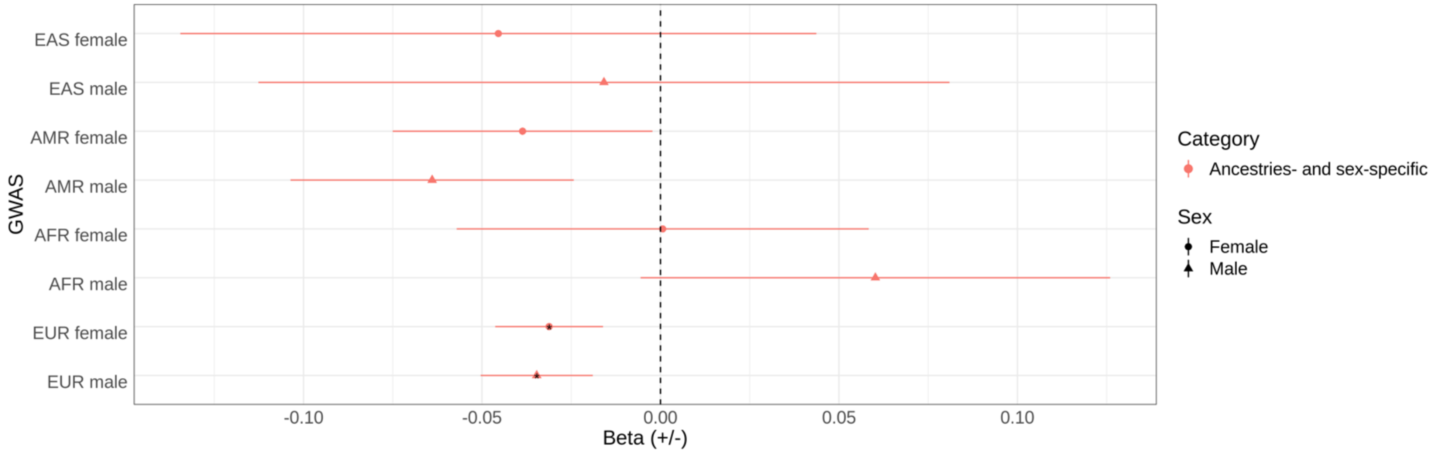

Supplementary Fig 74. Locus zoom and variant effect forest plot of rs669480. A) Locus zoom plot of trans-ancestries sex-combined meta-analysis stuttering associations, where the sentinel variant is denoted in purple and surrounding variants are color coded by r2 bin using linkage disequilibrium (LD) generated from 1000 Genomes ALL reference. The x axis represents chromosome position (hg37) with annotated genes found within the region, the y axis represents $\log_{10}$ (*p*-value) of the association between the genetic variant and stuttering. Sentinel variant is an intronic variant within *GRM5*. B) Variant effect forest plots of rs669480 found within the genetic ancestries of European male (EUR male), European female (EUR female), African male (AFR male), African female (AFR female), Latino/Admixed American male (AMR male), Latino/Admixed American female (AMR female), East Asian male (EAS male), and East Asian female (EAS female). Male variant effects are designated by triangles, and female variant effects are designed by circles. Line length indicates standard error for the betas found in the respective GWAS. Variants reaching replicative significance, *p*-value < 8.77×10^{-4} (.05/57 unique loci) are indicated by asterisks.

A

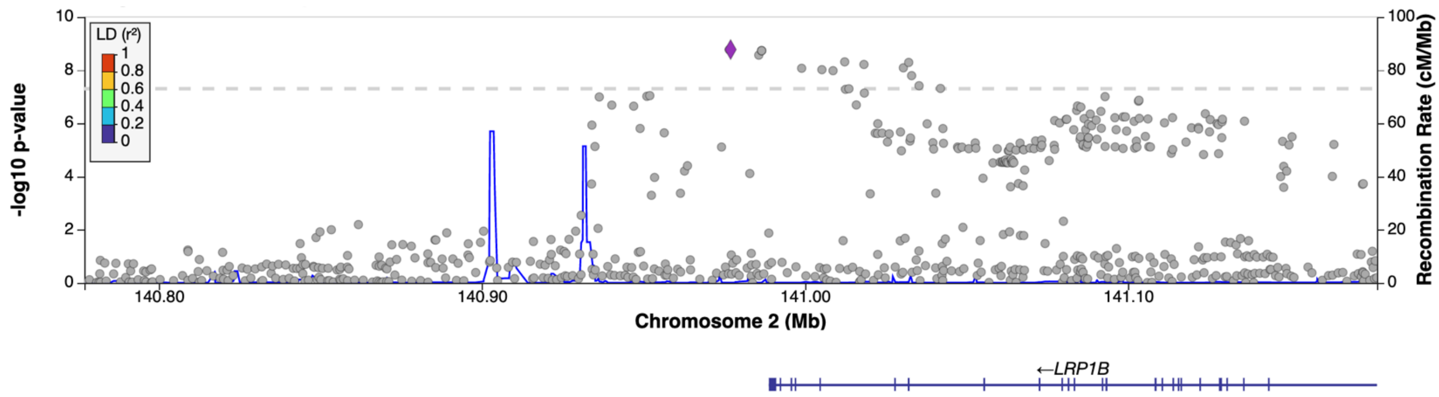

B

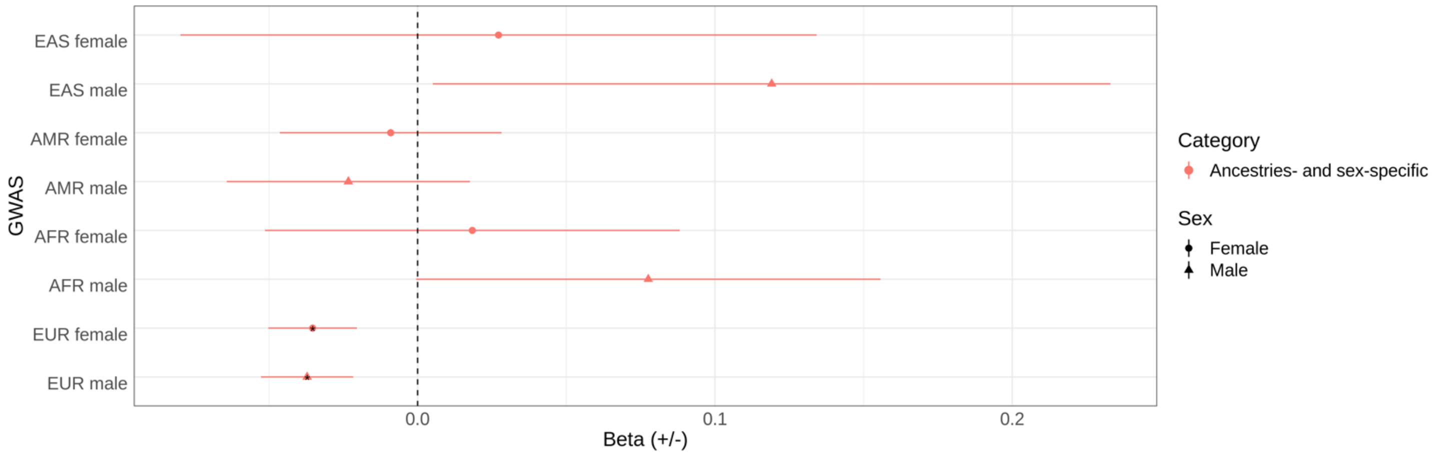

Supplementary Fig 75. Locus zoom and variant effect forest plot of rs4624326. A) Locus zoom plot of trans-ancestries sex-combined meta-analysis stuttering associations, where the sentinel variant is denoted in purple and surrounding variants are color coded by r2 bin using linkage disequilibrium (LD) generated from 1000 Genomes ALL reference. The x axis represents chromosome position (hg37) with annotated genes found within the region, the y axis represents $\log_{10}$ (*p*-value) of the association between the genetic variant and stuttering. Sentinel variant is upstream of *LRP1B*. B) Variant effect forest plots of rs4624326 found within the genetic ancestries of European male (EUR male), European female (EUR female), African male (AFR male), African female (AFR female), Latino/Admixed American male (AMR male), Latino/Admixed American female (AMR female), East Asian male (EAS male), and East Asian female (EAS female). Male variant effects are designated by triangles, and female variant effects are designed by circles. Line length indicates standard error for the betas found in the respective GWAS. Variants reaching replicative significance, *p*-value < 8.77×10^{-4} (.05/57 unique loci) are indicated by asterisks.

A

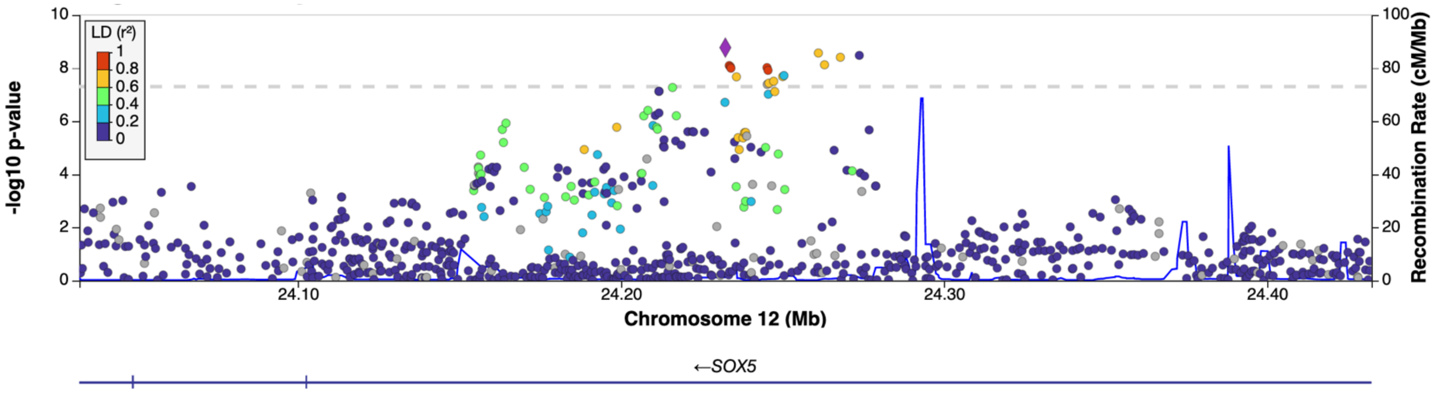

B

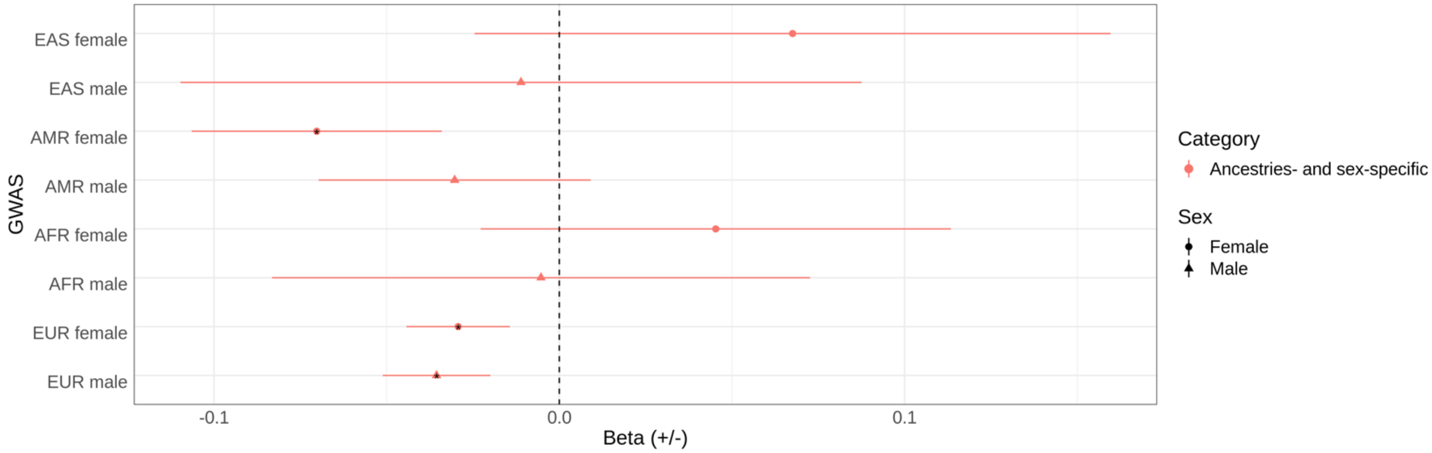

Supplementary Fig 76. Locus zoom and variant effect forest plot of rs10842270. A) Locus zoom plot of trans-ancestries sex-combined meta-analysis stuttering associations, where the sentinel variant is denoted in purple and surrounding variants are color coded by r2 bin using linkage disequilibrium (LD) generated from 1000 Genomes ALL reference. The x axis represents chromosome position (hg37) with annotated genes found within the region, the y axis represents $\log_{10}$ (*p*-value) of the association between the genetic variant and stuttering. Sentinel variant is a genetic upstream transcript or intronic variant within *SOX5*. B) Variant effect forest plots of rs10842270 found within the genetic ancestries of European male (EUR male), European female (EUR female), African male (AFR male), African female (AFR female), Latino/Admixed American male (AMR male), Latino/Admixed American female (AMR female), East Asian male (EAS male), and East Asian female (EAS female). Male variant effects are designated by triangles, and female variant effects are designed by circles. Line length indicates standard error for the betas found in the respective GWAS. Variants reaching replicative significance, *p*-value < 8.77×10^{-4} (.05/57 unique loci) are indicated by asterisks.

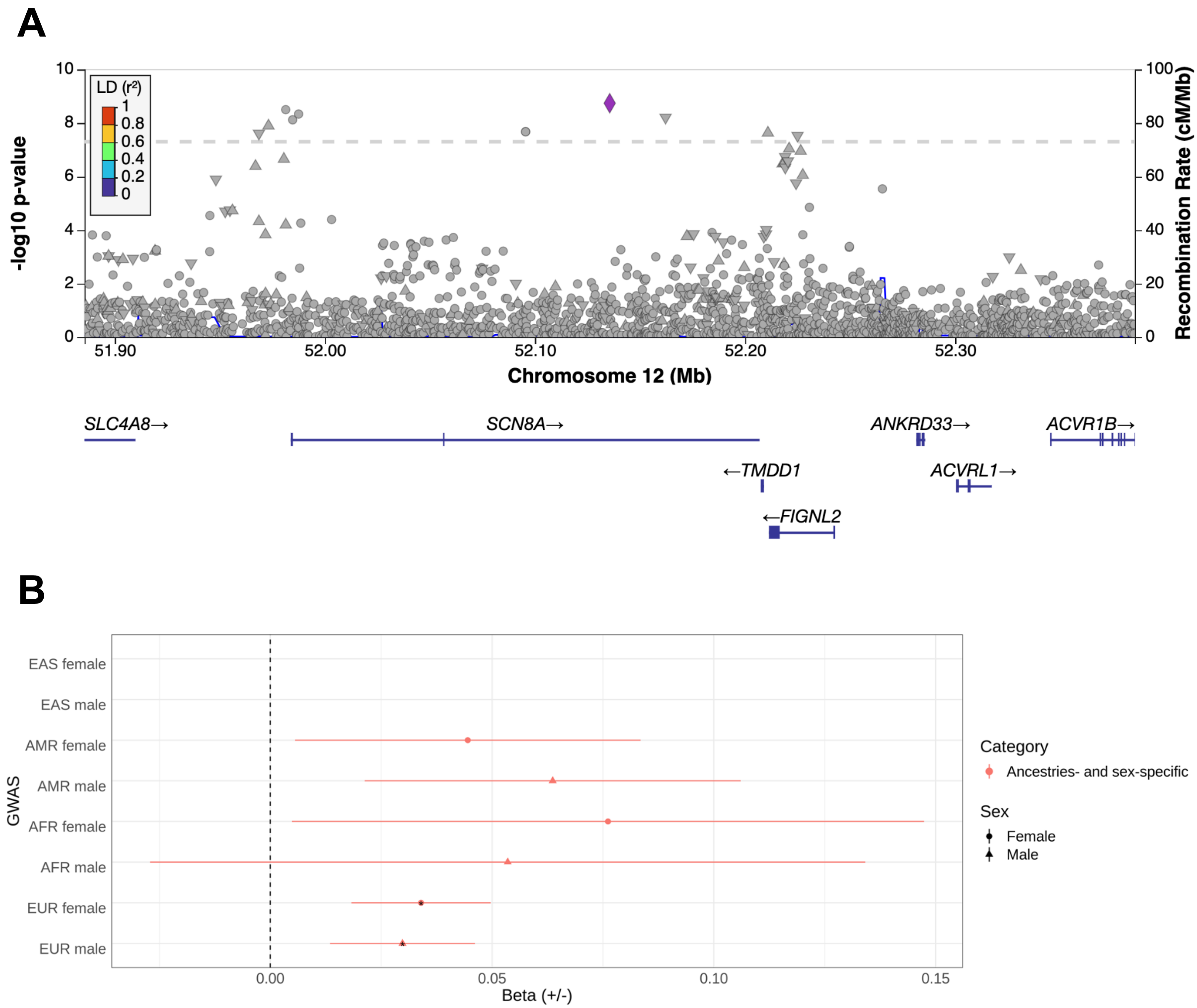

Supplementary Fig 77. Locus zoom and variant effect forest plot of rs11495450. A) Locus zoom plot of trans-ancestries sex-combined meta-analysis stuttering associations, where the sentinel variant is denoted in purple and surrounding variants are color coded by r2 bin using linkage disequilibrium (LD) generated from 1000 Genomes ALL reference. The x axis represents chromosome position (hg37) with annotated genes found within the region, the y axis represents $\log_{10}$ (*p*-value) of the association between the genetic variant and stuttering. Sentinel variant is an intronic variant within *SCN8A*. B) Variant effect forest plots of rs11495450 found within the genetic ancestries of European male (EUR male), European female (EUR female), African male (AFR male), African female (AFR female), Latino/Admixed American male (AMR male), and Latino/Admixed American female (AMR female). Variant not found in East Asian male (EAS male), and East Asian female (EAS female). Male variant effects are designated

by triangles, and female variant effects are designed by circles. Line length indicates standard error for the betas found in the respective GWAS. Variants reaching replicative significance, *p*-value < 8.77×10^{-4} (.05/57 unique loci) are indicated by asterisks.

A

B

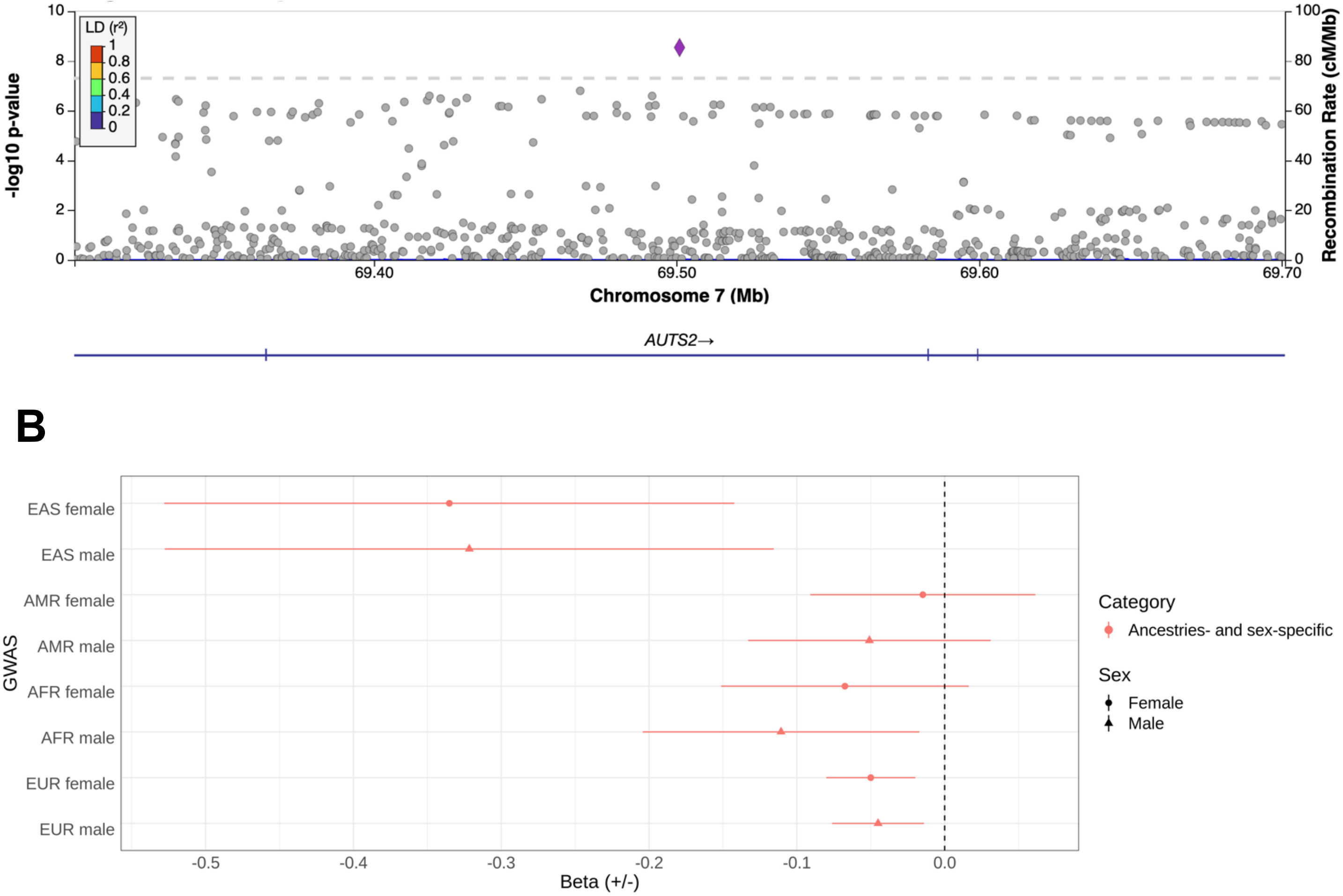

Supplementary Fig 78. Locus zoom and variant effect forest plot of rs7806413. A) Locus zoom plot of trans-ancestries sex-combined meta-analysis stuttering associations, where the sentinel variant is denoted in purple and surrounding variants are color coded by r2 bin using linkage disequilibrium (LD) generated from 1000 Genomes ALL reference. The x axis represents chromosome position (hg37) with annotated genes found within the region, the y axis represents $\log_{10}$ (*p*-value) of the association between the genetic variant and stuttering. SSentinel variant is a genetic upstream transcript or intronic variant within *AUTS2*. B) Variant effect forest plots of rs7806413 found within the genetic ancestries of European male (EUR male), European female (EUR female), African male (AFR male), African female (AFR female), Latino/Admixed American male (AMR male), Latino/Admixed American female (AMR female), East Asian male (EAS male), and East Asian female (EAS female). Male variant effects are designated by triangles, and female variant effects are designed by circles. Line length indicates standard error for the betas found in the respective GWAS. Variants reaching replicative significance, *p*-value < 8.77×10^{-4} (.05/57 unique loci) are indicated by asterisks.

A

B

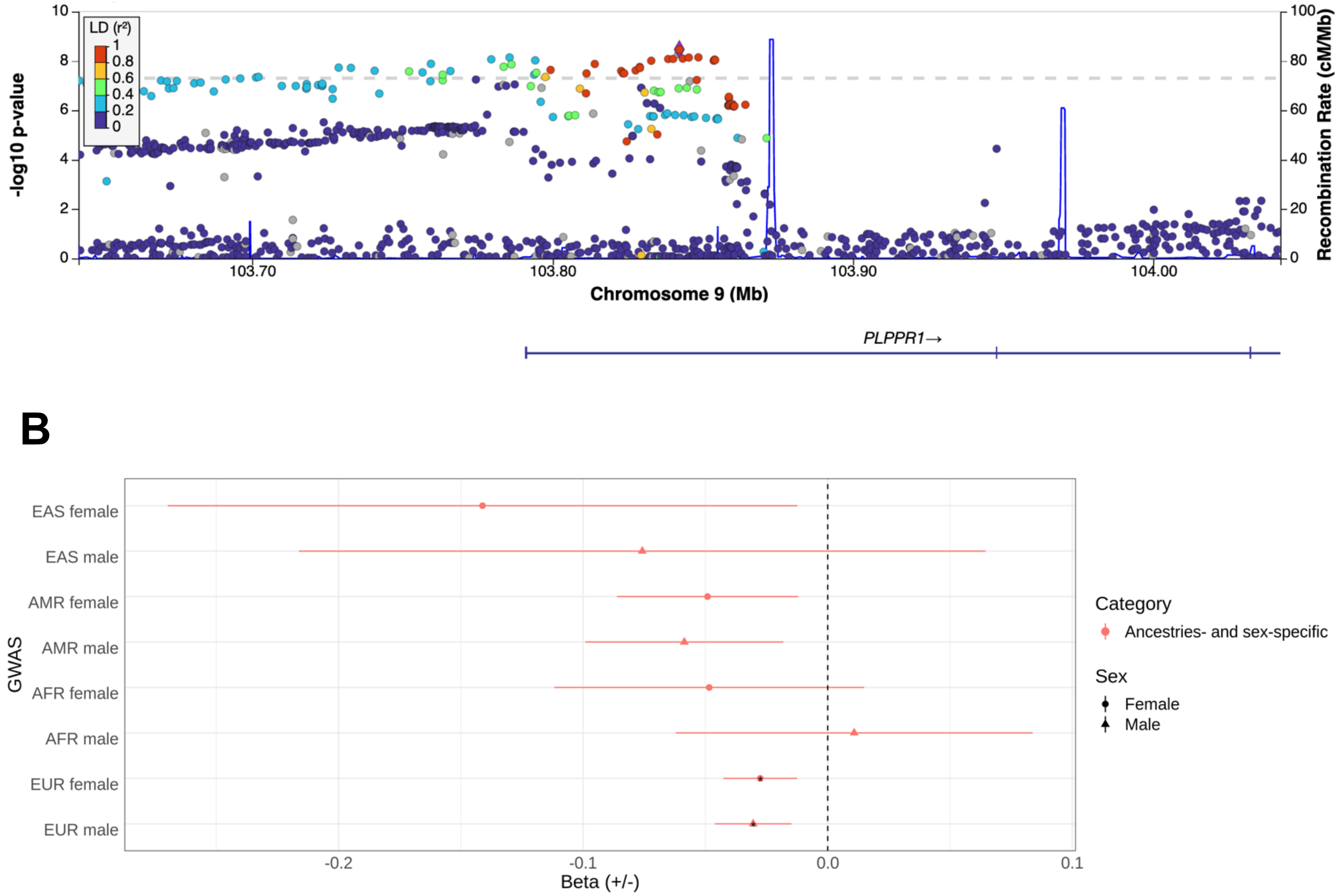

Supplementary Fig 79. Locus zoom and variant effect forest plot of rs650891. A) Locus zoom plot of trans-ancestries sex-combined meta-analysis stuttering associations, where the sentinel variant is denoted in purple and surrounding variants are color coded by r2 bin using linkage disequilibrium (LD) generated from 1000 Genomes ALL reference. The x axis represents chromosome position (hg37) with annotated genes found within the region, the y axis represents $\log_{10}$ (*p*-value) of the association between the genetic variant and stuttering. Sentinel variant is a genetic upstream transcript or intronic variant within *PLPPR1*. B) Variant effect forest plots of rs650891 found within the genetic ancestries of European male (EUR male), European female (EUR female), African male (AFR male), African female (AFR female), Latino/Admixed American male (AMR male), Latino/Admixed American female (AMR female), East Asian male (EAS male), and East Asian female (EAS female). Male variant effects are designated by triangles, and female variant effects are designed by circles. Line length indicates standard error for the betas found in the respective GWAS. Variants reaching replicative significance, *p*-value < 8.77×10^{-4} (.05/57 unique loci) are indicated by asterisks.

A

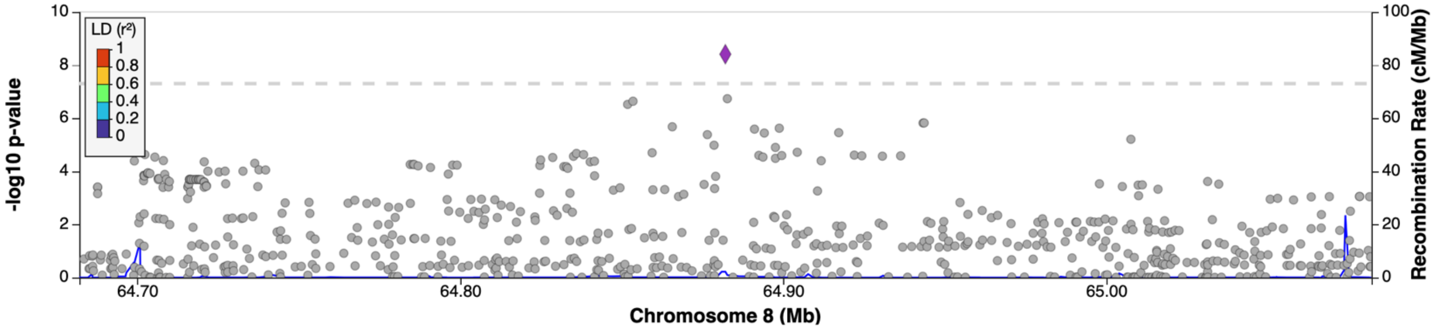

B

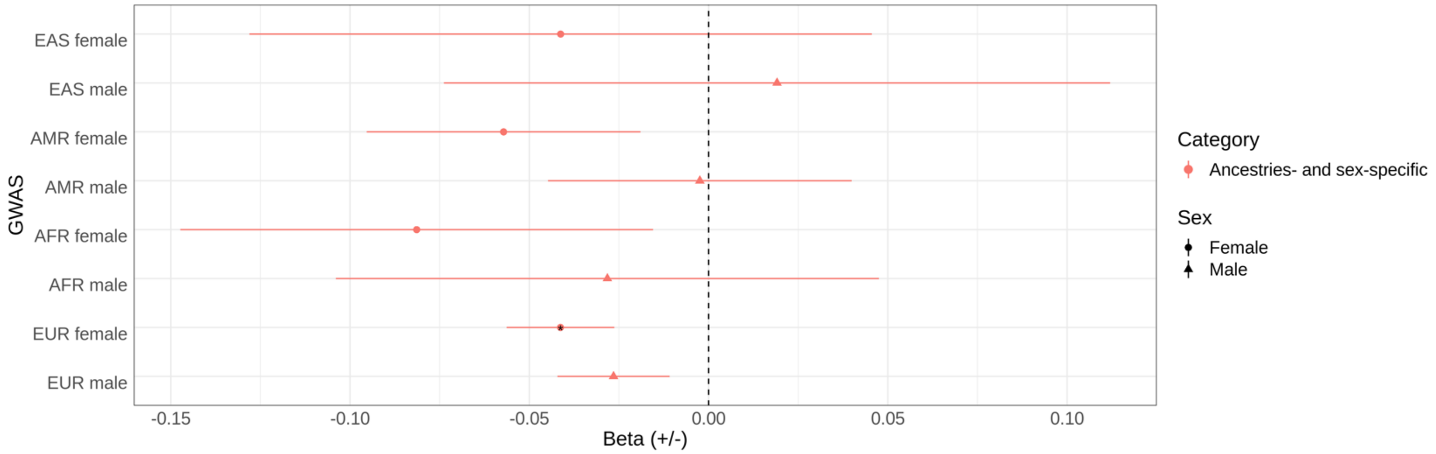

Supplementary Fig 80. Locus zoom and variant effect forest plot of rs10109510. A) Locus zoom plot of trans-ancestries sex-combined meta-analysis stuttering associations, where the sentinel variant is denoted in purple and surrounding variants are color coded by r2 bin using linkage disequilibrium (LD) generated from 1000 Genomes ALL reference. The x axis represents chromosome position (hg37) with annotated genes found within the region, the y axis represents $\log_{10}$ (*p*-value) of the association between the genetic variant and stuttering. Sentinel variant is located more than 500kb (upstream or downstream) from a protein-coding gene. B) Variant effect forest plots of rs10109510 found within the genetic ancestries of European male (EUR male), European female (EUR female), African male (AFR male), African female (AFR female), Latino/Admixed American male (AMR male), Latino/Admixed American female (AMR female), East Asian male (EAS male), and East Asian female (EAS female). Male variant effects are designated by triangles, and female variant effects are designed by circles. Line length indicates standard error for the betas found in the respective GWAS. Variants reaching replicative significance, *p*-value < 8.77×10^{-4} (.05/57 unique loci) are indicated by asterisks.

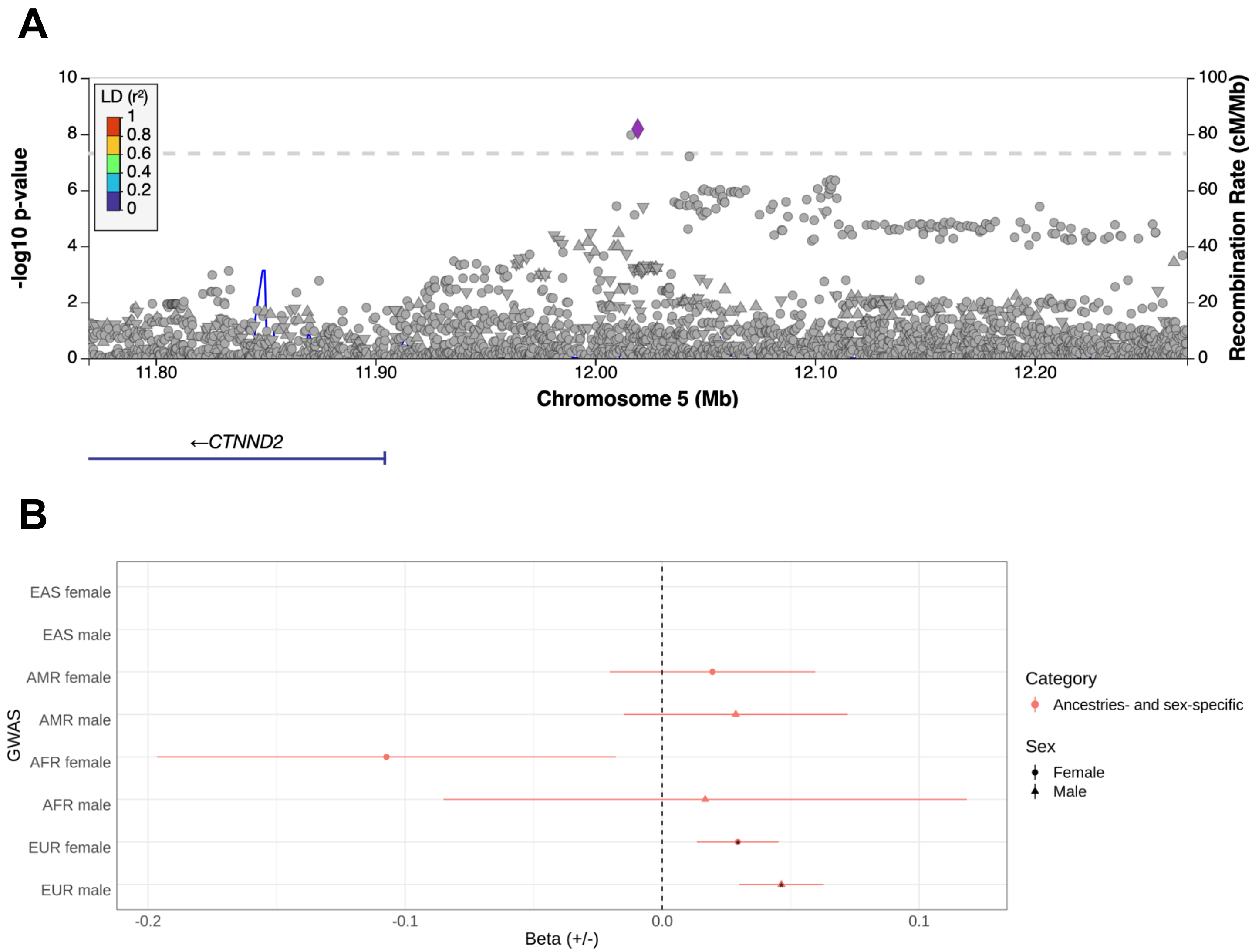

Supplementary Fig 81. Locus zoom and variant effect forest plot of rs4331897. A) Locus zoom plot of trans-ancestries sex-combined meta-analysis stuttering associations, where the sentinel variant is denoted in purple and surrounding variants are color coded by r2 bin using linkage disequilibrium (LD) generated from 1000 Genomes ALL reference. The x axis represents chromosome position (hg37) with annotated genes found within the region, the y axis represents $\log_{10}$ (*p*-value) of the association between the genetic variant and stuttering. Sentinel variant is upstream of *CTNND2*. B) Variant effect forest plots of rs4331897 found within the genetic ancestries of European male (EUR male), European female (EUR female), African male (AFR male), African female (AFR female), Latino/Admixed American male (AMR male), and Latino/Admixed American female (AMR female). Variant not found in East Asian male (EAS male), and East Asian female (EAS female). Male variant effects are designated by triangles, and female variant effects are designed by circles. Line length indicates standard error for the betas found in the respective GWAS. Variants reaching replicative significance, *p*-value <

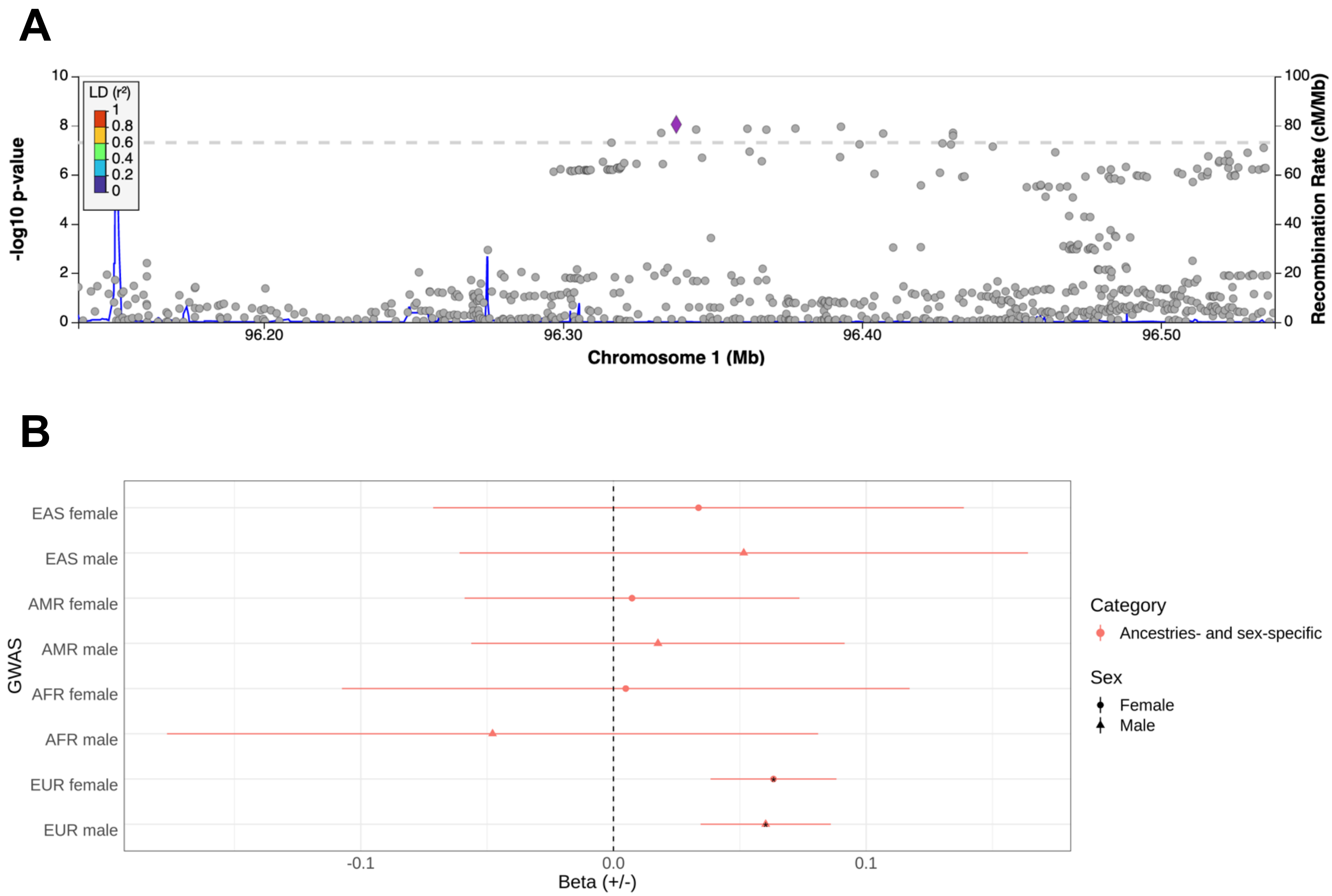

Supplementary Fig 82. Locus zoom and variant effect forest plot of rs12040559. Locus zoom plot of trans-ancestries sex-combined meta-analysis stuttering associations, where the sentinel variant is denoted in purple and surrounding variants are color coded by r2 bin using linkage disequilibrium (LD) generated from 1000 Genomes ALL reference. The x axis represents chromosome position (hg37) with annotated genes found within the region, the y axis represents $\log_{10}$ (*p*-value) of the association between the genetic variant and stuttering. Sentinel variant is located more than 500kb (upstream or downstream) from a protein-coding gene. B) Variant effect forest plots of rs12040559 found within the genetic ancestries of European male (EUR male), European female (EUR female), African male (AFR male), African female (AFR female), Latino/Admixed American male (AMR male), Latino/Admixed American female (AMR female), East Asian male (EAS male), and East Asian female (EAS female). Male variant effects are designated by triangles, and female variant effects are designed by circles. Line length indicates standard error for the betas found in the respective GWAS. Variants reaching replicative significance, *p*-value < 8.77×10^{-4} (.05/57 unique loci) are indicated by asterisks.

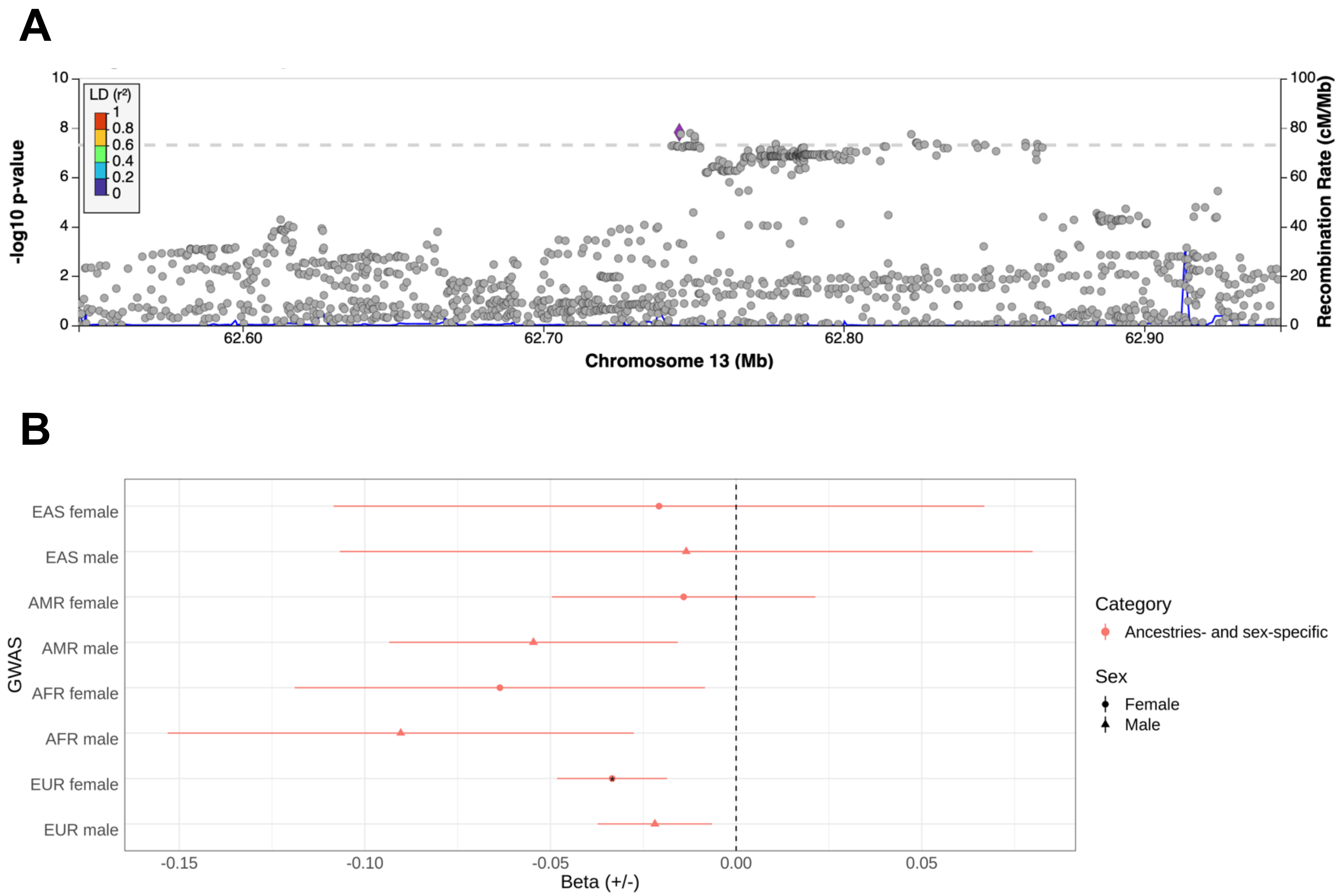

Supplementary Fig 83. Locus zoom and variant effect forest plot of rs56090583. A) Locus zoom plot of trans-ancestries sex-combined meta-analysis stuttering associations, where the sentinel variant is denoted in purple and surrounding variants are color coded by r2 bin using linkage disequilibrium (LD) generated from 1000 Genomes ALL reference. The x axis represents chromosome position (hg37) with annotated genes found within the region, the y axis represents $\log_{10}$ (*p*-value) of the association between the genetic variant and stuttering. Sentinel variant is located more than 500kb (upstream or downstream) from a protein-coding gene. B) Variant effect forest plots of rs56090583 found within the genetic ancestries of European male (EUR male), European female (EUR female), African male (AFR male), African female (AFR female), Latino/Admixed American male (AMR male), Latino/Admixed American female (AMR female), East Asian male (EAS male), and East Asian female (EAS female). Male variant effects are designated by triangles, and female variant effects are designed by circles. Line length indicates standard error for the betas found in the respective GWAS. Variants reaching replicative significance, *p*-value < 8.77×10^{-4} (.05/57 unique loci) are indicated by asterisks.

A

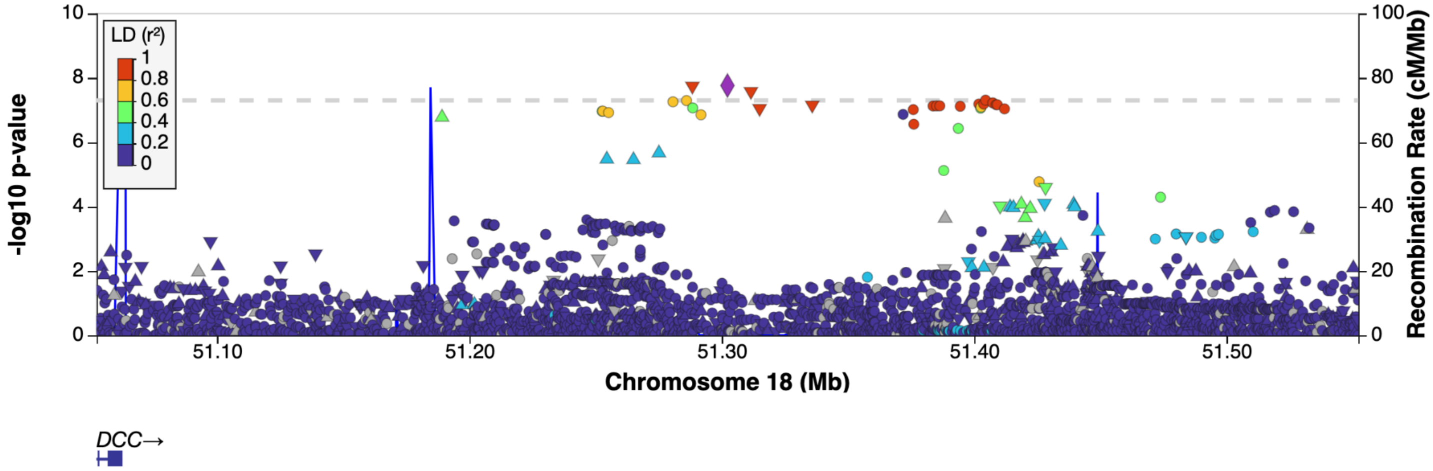

B

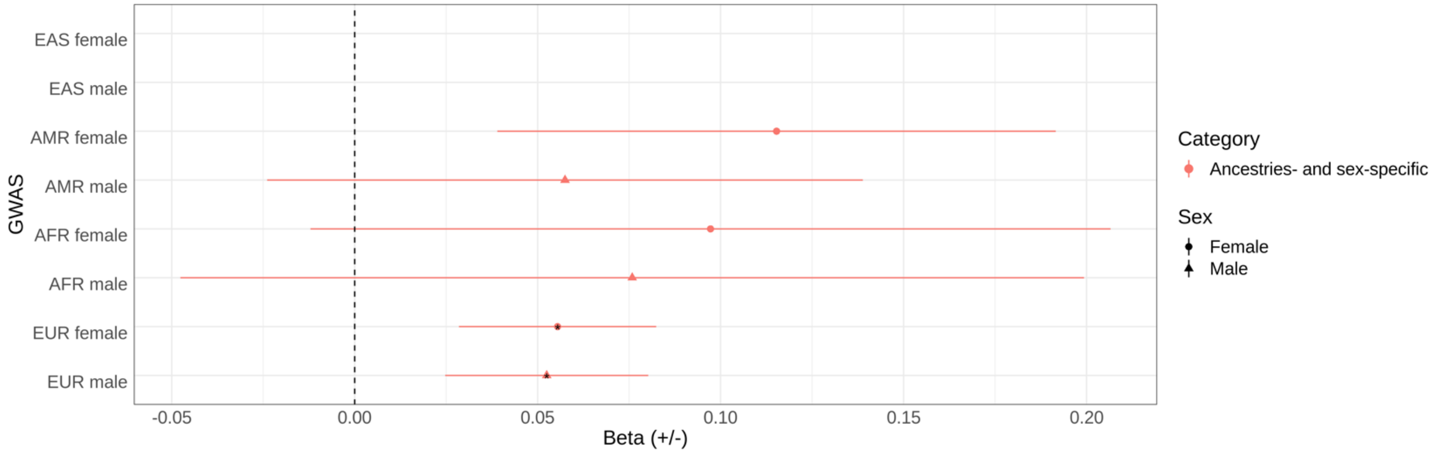

Supplementary Fig 84. Locus zoom and variant effect forest plot of rs7228924. A) Locus zoom plot of trans-ancestries sex-combined meta-analysis stuttering associations, where the sentinel variant is denoted in purple and surrounding variants are color coded by r2 bin using linkage disequilibrium (LD) generated from 1000 Genomes ALL reference. The x axis represents chromosome position (hg37) with annotated genes found within the region, the y axis represents $\log_{10}$ (*p*-value) of the association between the genetic variant and stuttering. Sentinel variant is located more than 500kb (upstream or downstream) from a protein-coding gene. B) Variant effect forest plots of rs7228924 found within the genetic ancestries of European male (EUR male), European female (EUR female), African male (AFR male), African female (AFR female), Latino/Admixed American male (AMR male), and Latino/Admixed American female (AMR female). Variant not found in East Asian male (EAS male), and East Asian female (EAS female). Male variant effects are designated by triangles, and female variant effects are designed by circles. Line length indicates standard error for the betas found in the

respective GWAS. Variants reaching replicative significance, *p*-value < 8.77 x 10^{-4} (.05/57 unique loci) are indicated by asterisks.

A

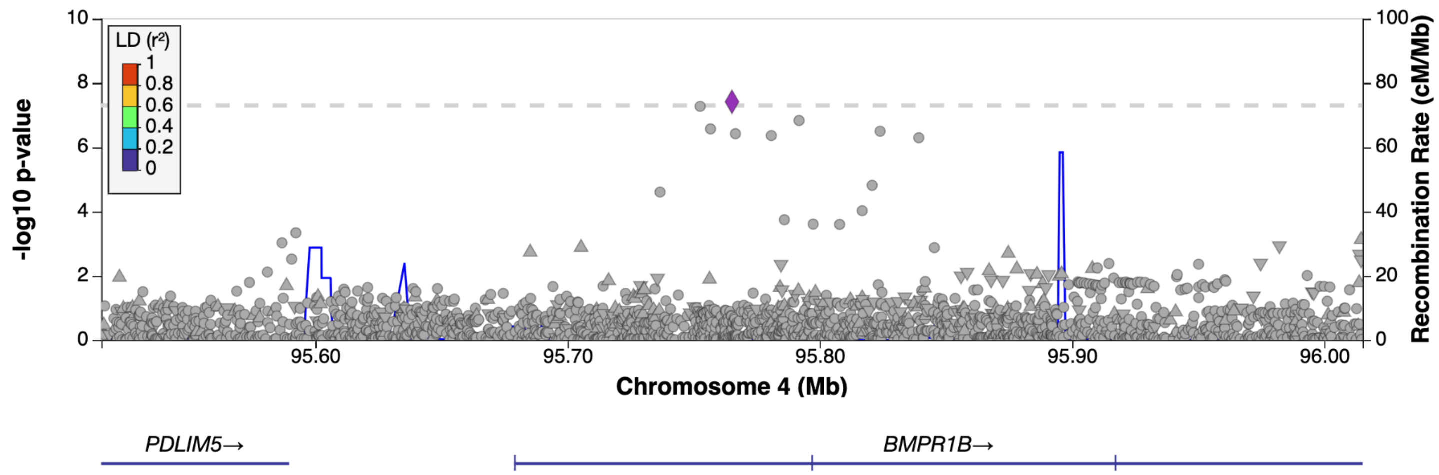

B

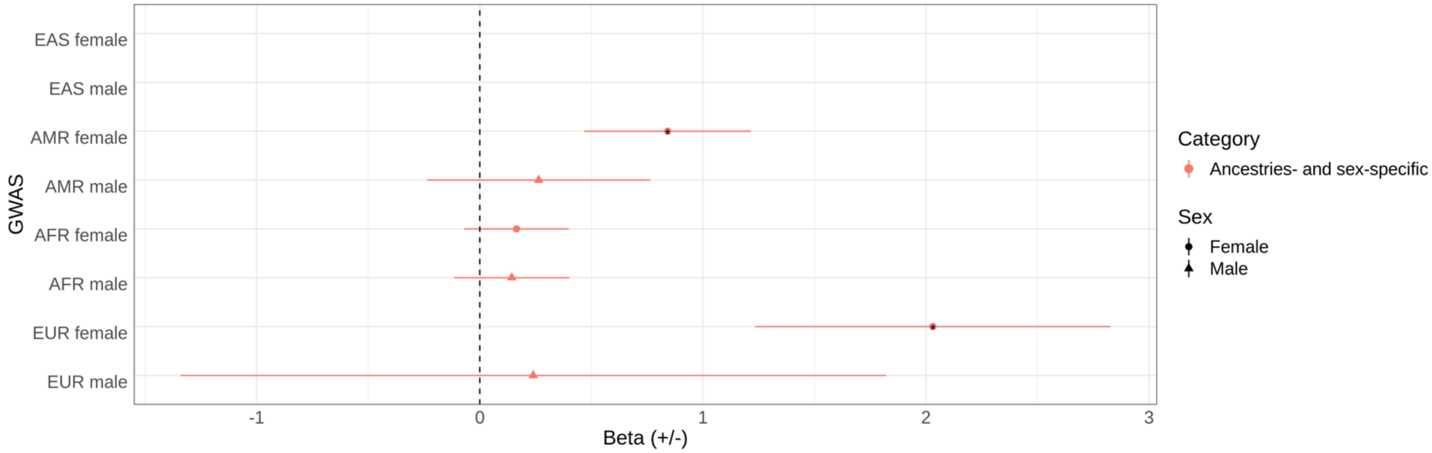

Supplementary Fig 85. Locus zoom and variant effect forest plot of rs143169712. A) Locus zoom plot of trans-ancestries sex-combined meta-analysis stuttering associations, where the sentinel variant is denoted in purple and surrounding variants are color coded by r2 bin using linkage disequilibrium (LD) generated from 1000 Genomes ALL reference. The x axis represents chromosome position (hg37) with annotated genes found within the region, the y axis represents $\log_{10}$ (*p*-value) of the association between the genetic variant and stuttering. Sentinel variant is a genetic upstream transcript or intronic variant within *BMPR1B*. B) Variant effect forest plots of rs143169712 found within the genetic ancestries of European male (EUR male), European female (EUR female), African male (AFR male), African female (AFR female), Latino/Admixed American male (AMR male), and Latino/Admixed American female (AMR female). Variant not found in East Asian male (EAS male), and East Asian female (EAS female). Male variant effects are designated by triangles, and female variant effects are designed by circles. Line length indicates standard error for the betas found in the respective GWAS.

Variants reaching replicative significance, *p*-value < 8.77×10^{-4} (.05/57 unique loci) are indicated by asterisks.

A

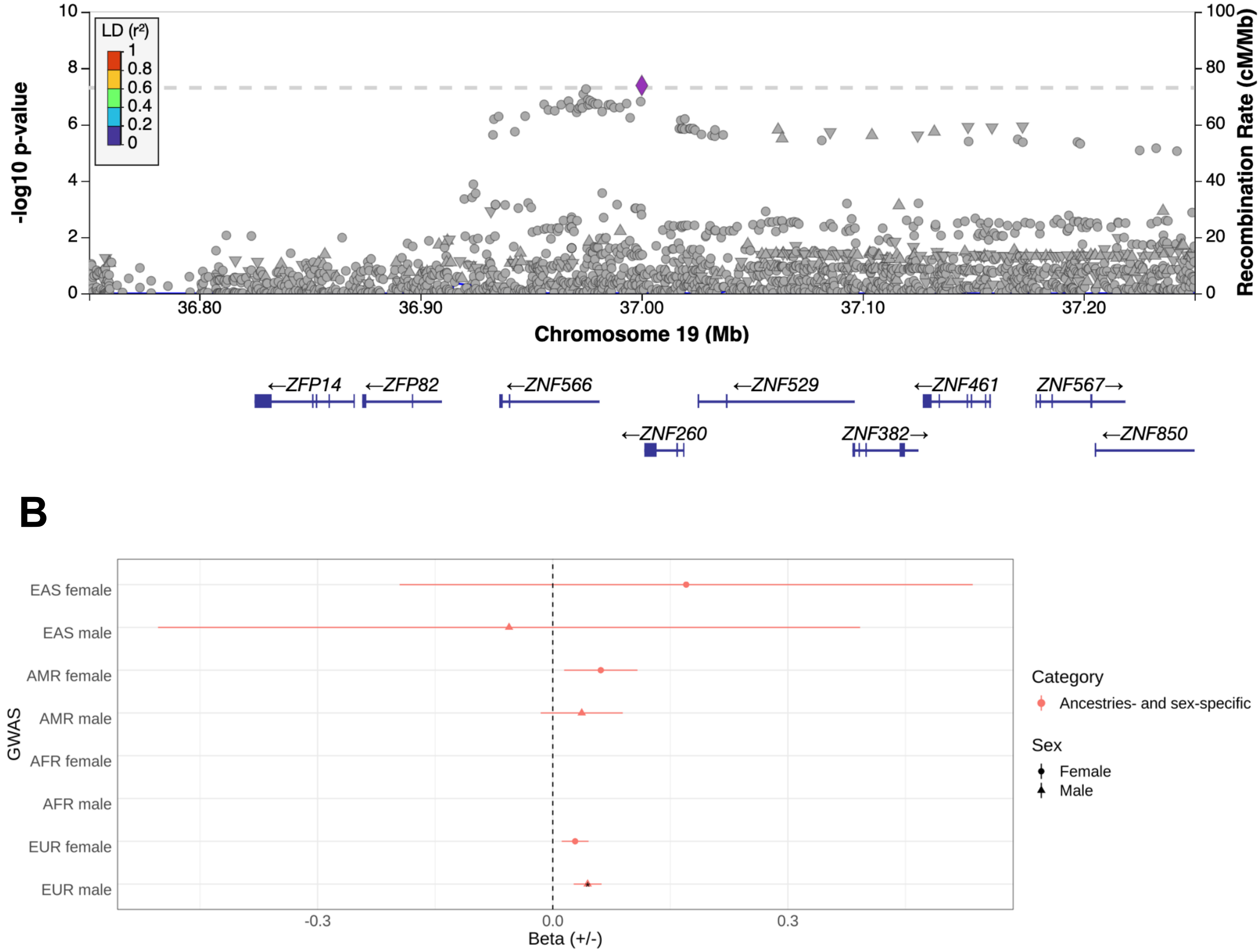

Supplementary Fig 86. Locus zoom and variant effect forest plot of rs73032465. A) Locus zoom plot of trans-ancestries sex-combined meta-analysis stuttering associations, where the sentinel variant is denoted in purple and surrounding variants are color coded by r2 bin using linkage disequilibrium (LD) generated from 1000 Genomes ALL reference. The x axis represents chromosome position (hg37) with annotated genes found within the region, the y axis represents $\log_{10}$ (*p*-value) of the association between the genetic variant and stuttering. Sentinel variant is between *ZNF566* and *ZNF567*. B) Variant effect forest plots of rs73032465 found within the genetic ancestries of European male (EUR male), European female (EUR female), Latino/Admixed American male (AMR male), Latino/Admixed American female (AMR female), East Asian male (EAS male), and East Asian female (EAS female). Variant not found in African male (AFR male), and African female (AFR female). Male variant effects are designated by triangles, and female variant effects are designed by circles. Line length indicates standard

error for the betas found in the respective GWAS. Variants reaching replicative significance, *p*-value < 8.77×10^{-4} (.05/57 unique loci) are indicated by asterisks.

A

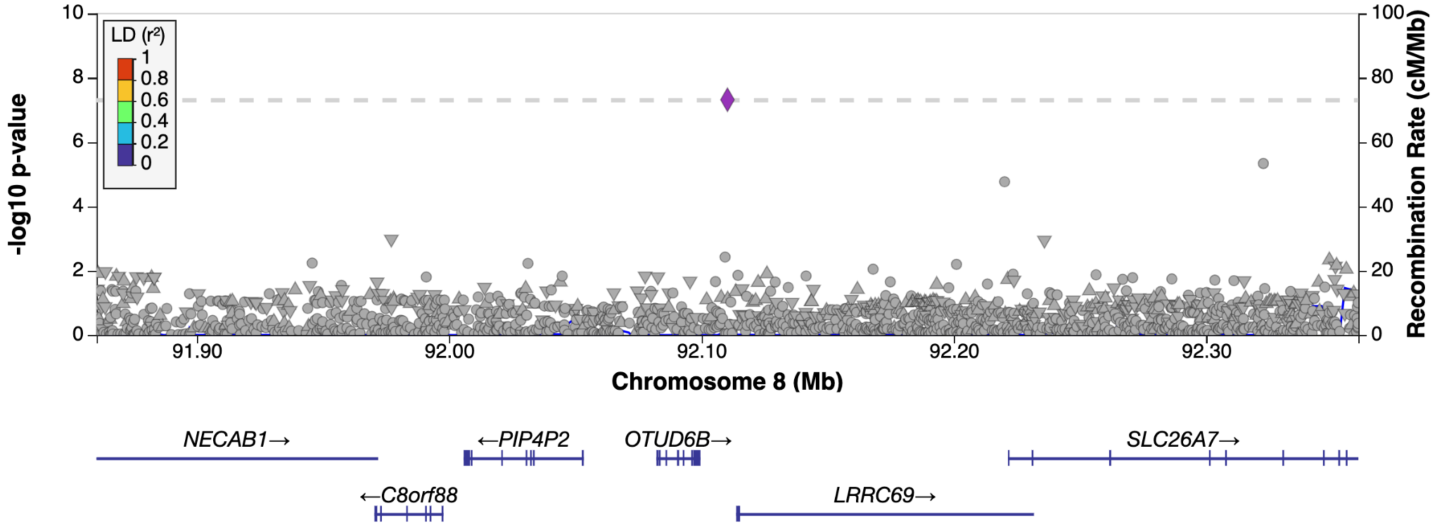

B

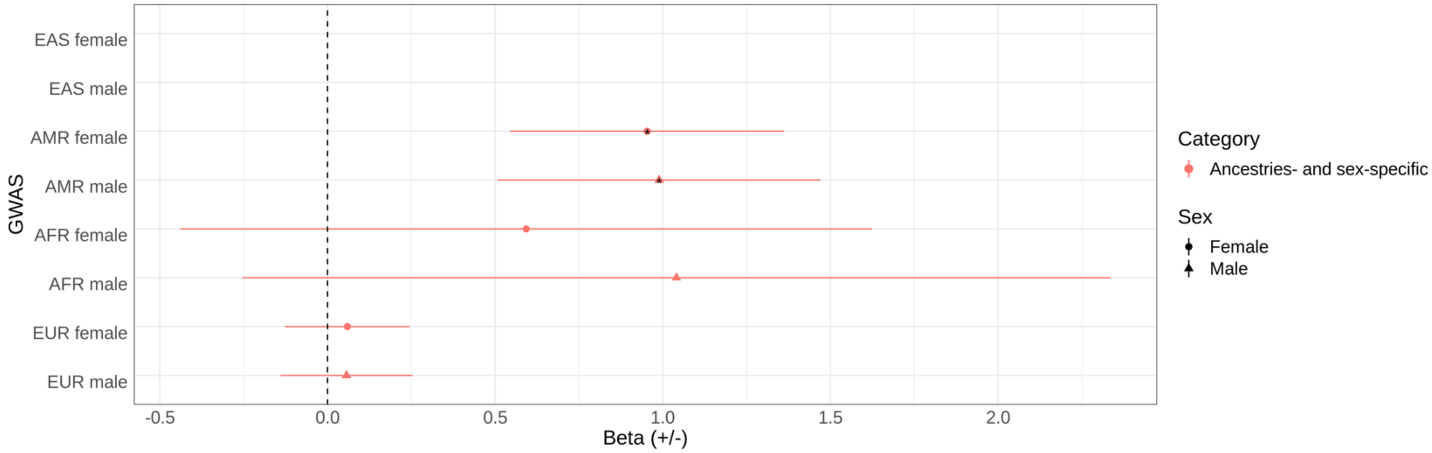

Supplementary Fig 87. Locus zoom and variant effect forest plot of rs141986240. A) Locus zoom plot of trans-ancestries sex-combined meta-analysis stuttering associations, where the sentinel variant is denoted in purple and surrounding variants are color coded by r2 bin using linkage disequilibrium (LD) generated from 1000 Genomes ALL reference. The x axis represents chromosome position (hg37) with annotated genes found within the region, the y axis represents $\log_{10}$ (*p*-value) of the association between the genetic variant and stuttering. Sentinel variant is between *OTUD68* and *LRRC69*. B) Variant effect forest plots of rs141986240 found within the genetic ancestries of European male (EUR male), European female (EUR female), African male (AFR male), African female (AFR female), Latino/Admixed American male (AMR male), and Latino/Admixed American female (AMR female). Variant not found in East Asian male (EAS male), and East Asian female (EAS female). Male variant effects are designated by triangles, and female variant effects are designed by circles. Line length indicates standard

error for the betas found in the respective GWAS. Variants reaching replicative significance, *p*-value < 8.77 x 10^{-4} (.05/57 unique loci) are indicated by asterisks.

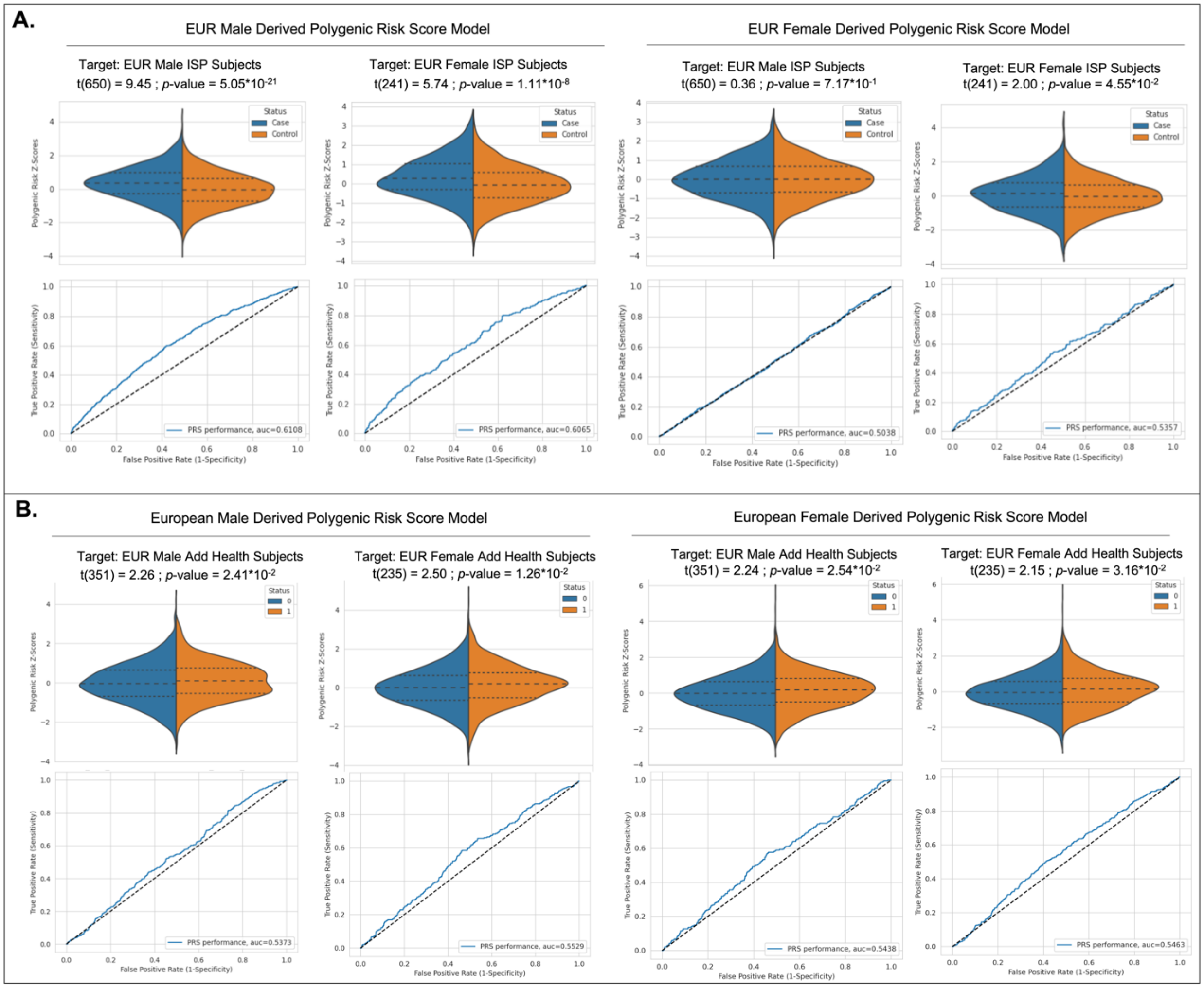

Supplementary Fig 88. Self-report stuttering polygenic risk-score model performance in European stuttering test sets. Polygenic risk scores developed using 23andMe GWAS results and applied to clinically validated International Stuttering Project and self-report Add Health subjects demonstrate increased stuttering liability within stuttering cases. The model was developed and trained using default auto-phi shrinkage parameter through PRScs. LD panels were constructed using 1KG phase 3 EUR reference. Polygenic risk scores were z-score normalized and case-control distributions were compared using Student's two-sample t-test (two-sided, α = 0.05). Cases colored in blue and controls in orange for both data sets. A) Represents AUC metrics and PRS scores in the ISP cohort. B) Represents AUC metrics and PRS Scores in Add Health subjects.

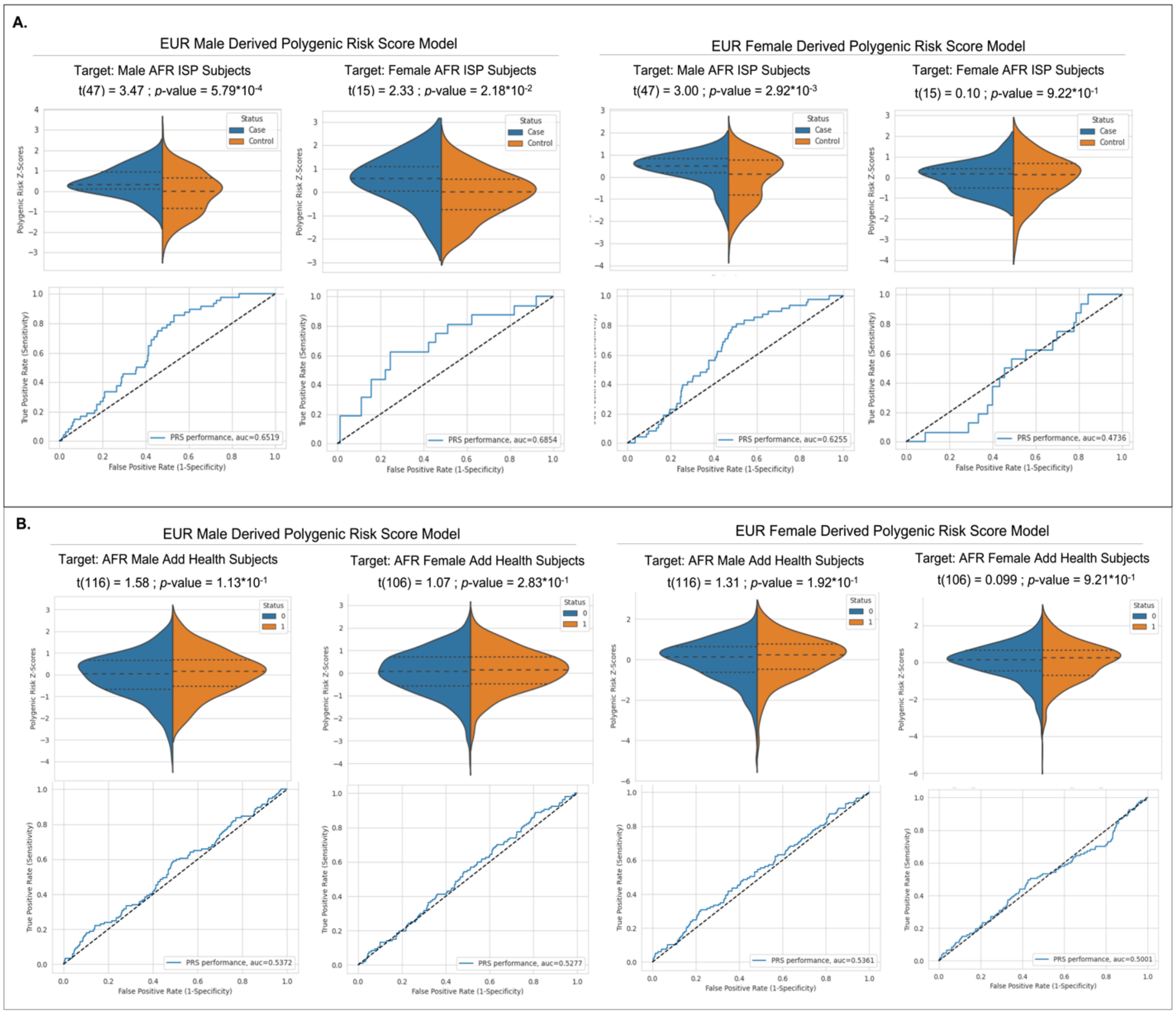

Supplementary Fig 89. Self-report stuttering polygenic risk-score model performance in AFR stuttering samples. Polygenic risk scores developed using 23andMe GWAS results and applied to clinically validated International Stuttering Project and Add Health subjects. The model was developed and trained using default auto-phi shrinkage parameter through PRScs. LD panels were constructed using 1KG phase 3 EUR reference. Polygenic risk scores were z-score normalized and case-control distributions were compared using Student's two-sample t-test (two-sided, α = 0.05). Cases colored in blue and controls in orange for both panels. A) Represents AUC metrics and PRS scores in the ISP cohort. B) Represents AUC metrics and PRS Scores in Add Health subjects.

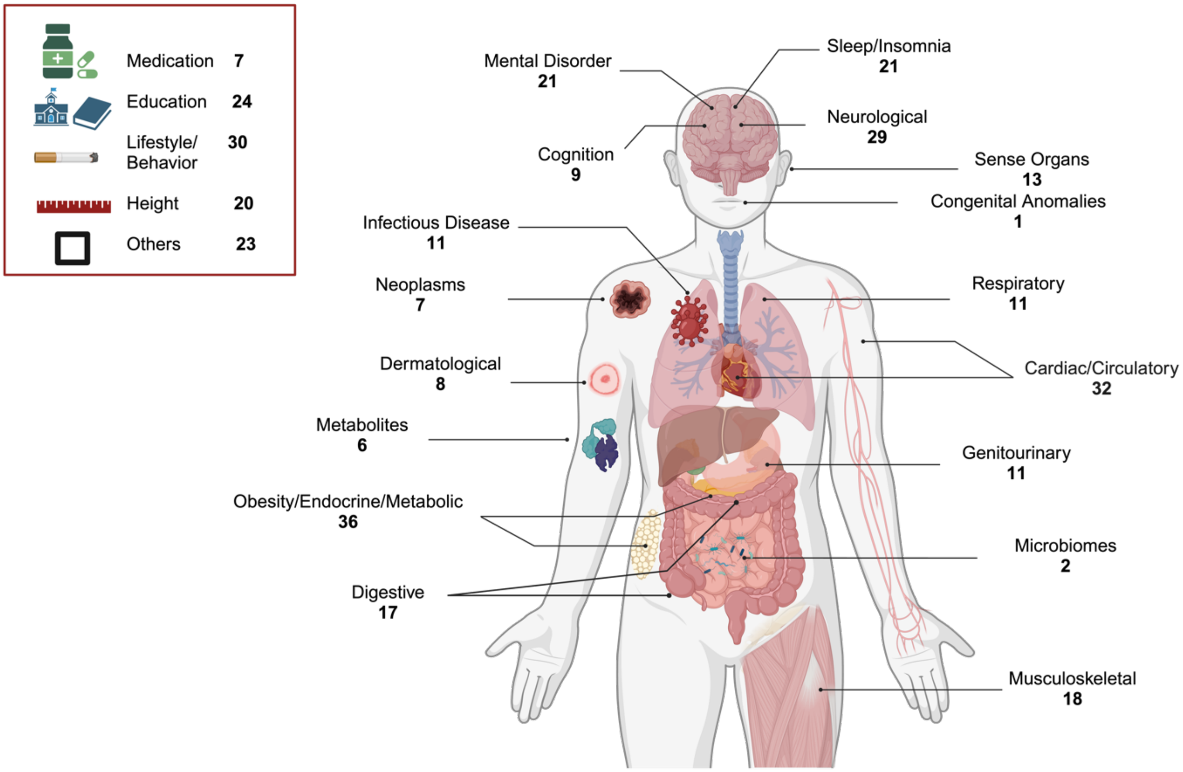

Supplementary Fig 90. **Number of unique stuttering risk genes by GWAS trait category.** Human body figure showing the number of unique identified stuttering genes associated with categorized traits in the GWAS Catalog (Release Date: 21-12-2022). Forty-eight unique genes, across ancestry- and sex-specific analyses, sex-combined, ancestry-specific meta-analyses, ancestry-combined, sex-specific meta-analyses, and ancestry- and sex-combined meta-analysis, were present in the GWAS Catalog search. GWAS Catalog traits and categories associated with our genome-wide significant hits are listed in Supplementary Table 19.
