# Supplementary material for: Large-scale genome-wide analyses of stuttering
Source: Nat Genet. 2025 Jul 28;57(8):1835–47. doi: 10.1038/s41588-025-02267-2 (PMC12339392; doi:10.1038/s41588-025-02267-2)
Supplement: Supplementary file 2 — Reporting Summary [file 41588_2025_2267_MOESM2_ESM.pdf]

nature portfolio

Corresponding author(s): Dr. Jennifer (Piper) Below
Last updated by author(s): 2/17/2025

Reporting Summary

Nature Portfolio wishes to improve the reproducibility of the work that we publish. This form provides structure for consistency and transparency in reporting. For further information on Nature Portfolio policies, see our Editorial Policies and the Editorial Policy Checklist.

Statistics

For all statistical analyses, confirm that the following items are present in the figure legend, table legend, main text, or Methods section.

n/a	Confirmed	
☐	☒	The exact sample size (*n*) for each experimental group/condition, given as a discrete number and unit of measurement
☐	☒	A statement on whether measurements were taken from distinct samples or whether the same sample was measured repeatedly
☐	☒	The statistical test(s) used AND whether they are one- or two-sided<br>*Only common tests should be described solely by name; describe more complex techniques in the Methods section.*
☐	☒	A description of all covariates tested
☐	☒	A description of any assumptions or corrections, such as tests of normality and adjustment for multiple comparisons
☐	☒	A full description of the statistical parameters including central tendency (e.g. means) or other basic estimates (e.g. regression coefficient) AND variation (e.g. standard deviation) or associated estimates of uncertainty (e.g. confidence intervals)
☐	☒	For null hypothesis testing, the test statistic (e.g. *F*, *t*, *r*) with confidence intervals, effect sizes, degrees of freedom and *P* value noted<br>*Give P values as exact values whenever suitable.*
☒	☐	For Bayesian analysis, information on the choice of priors and Markov chain Monte Carlo settings
☒	☐	For hierarchical and complex designs, identification of the appropriate level for tests and full reporting of outcomes
☐	☒	Estimates of effect sizes (e.g. Cohen's *d*, Pearson's *r*), indicating how they were calculated

Our web collection on statistics for biologists contains articles on many of the points above.

Software and code

Policy information about availability of computer code

Data collection	23andMe, Inc. collected data.
Data analysis	Open Targets Genetics (22.10), LDSC (LD SCore) v2.0.0, Python 3.9.7, R 4.0.0, Mendelian Randomization Package v.7.0, PRScs (last updated version: Oct 20, 2019), MR-MEGA ver. 0.2, METAL (version released on: 2011-03-25), PLINK v1.9. fastENLOC v1.0, eMAGMA (integrates MAGMA v1.07b), and concordance analyses (https://github.com/belowlab/Concordance-Analysis or https://doi.org/10.5281/zenodo.14884575).

For manuscripts utilizing custom algorithms or software that are central to the research but not yet described in published literature, software must be made available to editors and reviewers. We strongly encourage code deposition in a community repository (e.g. GitHub). See the Nature Portfolio guidelines for submitting code & software for further information.

Data

Policy information about availability of data

All manuscripts must include a data availability statement. This statement should provide the following information, where applicable:

- Accession codes, unique identifiers, or web links for publicly available datasets
- A description of any restrictions on data availability
- For clinical datasets or third party data, please ensure that the statement adheres to our policy

Ancestry- and sex-specific summary statistics of self-reported stuttering will be made available through the 23andMe website to qualified researchers under

agreement with 23andMe that protects the privacy of the 23andMe participants. Interested investigators should visit the 23andMe Publication Dataset Access Program at https://research.23andme.com/dataset-access/. The top 10,000 SNPs for all primary analyses are provided as an Extended Data file.

Research involving human participants, their data, or biological material

Policy information about studies with human participants or human data. See also policy information about sex, gender (identity/presentation), and sexual orientation and race, ethnicity and racism.

Reporting on sex and gender	We analyzed data stratified by sex, as well as downstream meta-analyses results.
Reporting on race, ethnicity, or other socially relevant groupings	We analyzed data stratified by genetic ancestries, as well as meta-analyzed primary results.
Population characteristics	See below.
Recruitment	Participants are customers of 23andMe, Inc., and are invited to participate in general research, our study uses data from online questions. There is under-representation of low socio-economic status.
Ethics oversight	Vanderbilt University

Note that full information on the approval of the study protocol must also be provided in the manuscript.

Field-specific reporting

Please select the one below that is the best fit for your research. If you are not sure, read the appropriate sections before making your selection.

☐ Life sciences ☒ Behavioural & social sciences ☐ Ecological, evolutionary & environmental sciences

For a reference copy of the document with all sections, see nature.com/documents/nr-reporting-summary-flat.pdf

Behavioural & social sciences study design

All studies must disclose on these points even when the disclosure is negative.

Study description	This is a quantitative study that relies on self-report data. Replication analyses draw on confirmed stuttering status by a speech language pathologist, and self-reported parent perception of child stuttering.
Research sample	The research sample are customers of 23andMe, Inc., a consumer genetics company, who have agreed to participate in research. Demographic details (see Table 1). They are slightly selected in that there is over-representation of higher socio-economic participants. The replication sample were recruited from speech and language clinics, and controls were drawn from the general population.
Sampling strategy	The sample were volunteer customers of 23andMe, Inc., who consented to the use of their DNA and survey results (voluntary response sampling). The largest sample size available at the time of study was used.
Data collection	Data collection for the main analysis was online survey collection. For replication samples, it was testing from a speech language pathologist, and also self-reported parent perception of child stuttering.
Timing	The main sample data include customers who consented to participate up until June 2020. Both replication datasets varied in collection times. The National Longitudinal Study of Adolescent to Adult Health began data collection around 1994, and the International Stuttering Project began data collection around 2011. Both replication dataset sample collections stopped around 2021, with data collection in these cohorts currently ongoing.
Data exclusions	Participants who had marked "not sure" were excluded from data analyses. Exclusion criteria was pre-established.
Non-participation	This is not a longitudinal study. Hence, there are no sample drop outs to report.
Randomization	There was no randomization, but we controlled for age, and stratified by genetic sex and ancestries in analyses.

Reporting for specific materials, systems and methods

We require information from authors about some types of materials, experimental systems and methods used in many studies. Here, indicate whether each material, system or method listed is relevant to your study. If you are not sure if a list item applies to your research, read the appropriate section before selecting a response.

Materials & experimental systems

n/a	Involved in the study
☒	☐ Antibodies
☒	☐ Eukaryotic cell lines
☒	☐ Palaeontology and archaeology
☒	☐ Animals and other organisms
☒	☐ Clinical data
☒	☐ Dual use research of concern
☒	☐ Plants

Methods

n/a	Involved in the study
☒	☐ ChIP-seq
☒	☐ Flow cytometry
☒	☐ MRI-based neuroimaging
